# Bifunctional Catalysis of Aldol Reactions by Foldamer Dihydrazides: Assessment of Conformational Preorganization

**DOI:** 10.1021/jacs.5c10914

**Published:** 2025-09-04

**Authors:** Philip P. Lampkin, Kyana M. Sanders, Leah C. Garman, Ilia A. Guzei, Samuel H. Gellman

**Affiliations:** Department of Chemistry, 5228 University of Wisconsin−Madison, 1101 University Avenue, Madison, Wisconsin 53706, United States

## Abstract

We previously reported that molecules containing two cyclic hydrazide units connected by a polymethylene linker could catalyze aldol condensations via a bifunctional mechanism. One hydrazide apparently provides nucleophilic activation, via enamine formation, while the other provides electrophilic activation, via iminium formation. Here, we ask whether catalytic efficacy can be enhanced by using a conformationally preorganized linker to connect the hydrazide units. In the new catalyst series, the linkers are α/β-peptides (oligomers containing α- and β-amino acid residues). The α/β-peptide scaffold features a 1:2 α:β backbone repeat and forms a helix with approximately three residues per turn. When α residues with hydrazide-containing side chains have an *i*,​​*i*+3 sequence relationship, helical folding induces alignment of the hydrazides. Incremental variation of side chain length allowed us to identify an optimal spacing between the hydrazide units, comprising 16 atoms. Catalytic efficacy, as judged by relative initial rates of an aldol condensation, was ∼3.5-fold greater for this α/β-peptide relative to the dihydrazide with a flexible 16-atom spacer from the previous series. Single-crystal X-ray crystallographic analyses of three α/β-peptide catalysts provide insight into modes of conformational flexibility available to these foldamers.

## Graphical abstract

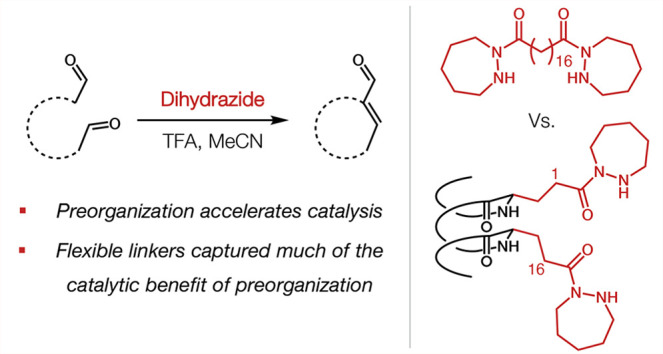

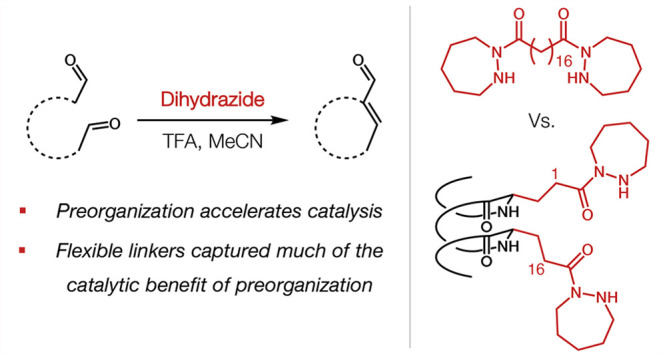

## Introduction

Enzyme-mediated increases in reaction rates are thought to arise from diverse features of active sites, including precise orientation of sets of reactive groups presented by the protein,^1^ arrangement of substrates relative to one another,^2^ substrate desolvation,^3,4^ tailoring of the local electrostatic environment,^5^ and coupling of enzyme dynamics to reaction coordinates.^6–8^ Many efforts to create synthetic catalysts have involved attempts to endow unnatural molecular frameworks with at least one enzyme-inspired feature.^9–23^ Perhaps the most common approach has been to arrange two or more reactive groups on a small-molecule scaffold to enable coordinated engagement of substrate(s).^24–35^

We previously reported that the aldol condensation shown in Figure 1A is catalyzed by a simple seven-membered ring hydrazide, and that the initial rate of product formation displays an apparent second-order dependence on hydrazide concentration.^36^ This observation is consistent with simultaneous electrophilic substrate activation via iminium formation and nucleophilic substrate activation via enamine formation. According to this hypothesis, two hydrazide-derived units would be present in the transition state for carbon–carbon bond formation. This mechanistic understanding led to evaluation of a series of polymethylene-linked dihydrazides for bifunctional catalysis of the aldol condensation. The dihydrazide with a 10-methylene linker displayed the greatest catalytic activity, i.e., the highest initial rate of product formation under dilute conditions.^36^

**Figure 1:**
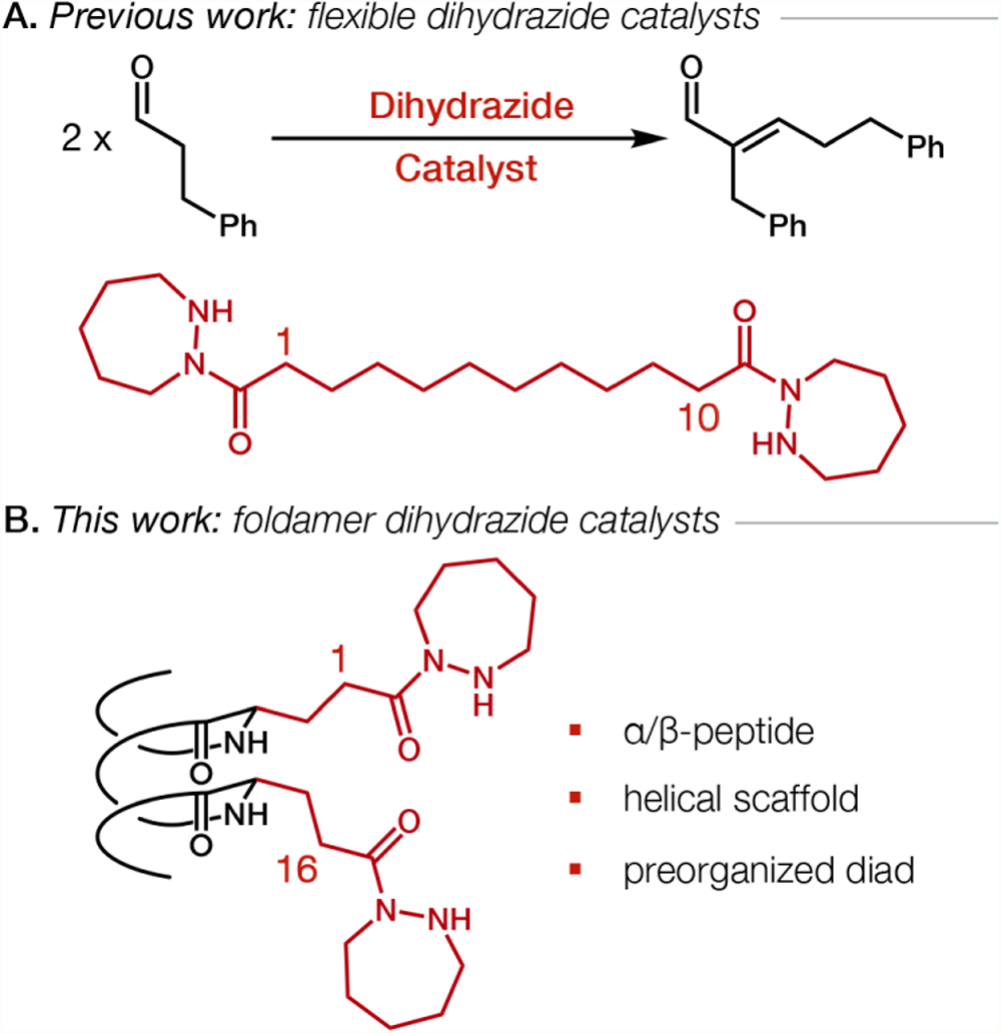
(A) Bifunctional catalysis of a homoaldol condensation by a flexible dihydrazide. (B) Cartoon of a helical α/β-peptide with a preorganized hydrazide diad.

The present study asks whether bifunctional catalysis can be enhanced by replacing the flexible linking segment with a conformationally defined linker that restrains the orientation of the two hydrazide units (Figure 1B). Peptides have been widely employed as scaffolds to link reactive groups with the goal of enhancing catalytic outcomes.^37–40^ Many of these efforts have featured oligomers of α-amino acid residues; impressive levels of stereoselectivity have been achieved with these chiral catalysts.^41–47^ The intrinsic modularity of the peptide backbone facilitates structural variation among catalyst candidates, and the benefits of modularity are retained when building blocks such as β-, γ- or δ-amino acids are employed or nonpeptide oligomeric scaffolds are examined.^48–54^

We have identified a peptide backbone containing α- and β-amino acid residues that is particularly favorable for orienting a pair of reactive groups.^55–57^ This α/β-peptide family features a 1:2 α:β repeat and favors a helical conformation with three residues per turn. This scaffold allows one to harness residue-based strategies for constraining conformational flexibility that are available for β but not α residues: helix stability is enhanced with β residues that feature a five-membered ring constraint.^58^ The folding of these α/β-peptides is expected to align positions of successive α residues (*i*,*i*+3 spacing) along one side of the helix. Reactive side chains presented by these α residues can act on substrates in a coordinated fashion. We have shown that α/β-peptides with amine diads catalyze aldol-based macrocyclization reactions.^59,60^

The work described here evaluates aldol catalysis by α/β-peptides with a conformationally preorganized hydrazide diad. The α/β-peptide data provide a basis for comparisons with the catalytic activities observed for dihydrazides containing flexible polymethylene linkers. These comparisons reveal a 2.5- to 3.5-fold boost in catalytic activity from the α/β-peptide scaffold relative to the linkers lacking preorganization. Compared to the remarkable catalytic activities of enzymes that appear to be enabled, at least in part, by reactive group preorganization, we interpret rate enhancement due to preorganization to be surprisingly modest in our system. Our findings include the first crystal structures of catalytically active α/β-peptide foldamers. These structures show how α/β-peptides can preorganize a catalytic diad and offer glimpses of conformational flexibility that is likely to be manifested in solution.

## Results and Discussion

### Reactivity

We focused on the homoaldol condensation of hydrocinnamaldehyde for assessing catalytic efficacy of α/β-peptide foldamers, as in our previous study of flexible dihydrazides.^36^ This work was not intended to establish a new preparative method. Instead, we used an easily monitored homoaldol reaction as the basis for comparing catalytic efficacy among molecules containing a hydrazide diad. Reactivity was evaluated by measuring product yields at a defined time point (1 h) and initial rates of product formation using ^1^H NMR and an internal standard. Initial rates were determined by measuring product concentration at several time points at low reaction completion for each catalyst.

To facilitate comparisons, we present relative initial rate (ν_*REL*_) values for dihydrazides, which are initial rates normalized to the initial rate for the simple hydrazide **1**. Because the aldol condensation rate showed an approximately second order dependence on the concentration of monohydrazide **1** but an approximately first order dependence on the concentrations of dihyrazides,^36^ the ν_*REL*_ values are derived from reactions in which the concentration of the monohydrazide is twice the concentration of the dihydrazide, which means that the total concentration of hydrazide units is the same in the two reaction solutions. The absolute hydrazide concentration was held constant across all hydrocinnamaldehyde reactions we compared, 0.5 mM for dihydrazides vs 1 mM for **1** and other monohydrazides. This consistency is important because ν_*REL*_ values vary as a function of catalyst concentration.

Figure 2A provides ν_*REL*_ values for a set of α/β-peptides that contain at least one cyclic hydrazide unit in a side chain. The hydrazide was incorporated into the α/β-peptides via a glutamic acid-derived residue (Glu­(Hy)), an aspartic acid-derived residue (Asp­(Hy)) or a residue bearing a more extended side chain derived from diamino propionic acid and succinic acid (Dap­(SuccHy)) (Figure 2B). All α/β-peptides had a triphenylpropionyl group (TPP) at the N-terminus and a *N*-methylamide group at the C-terminus to promote solubility in acetonitrile.

**Figure 2:**
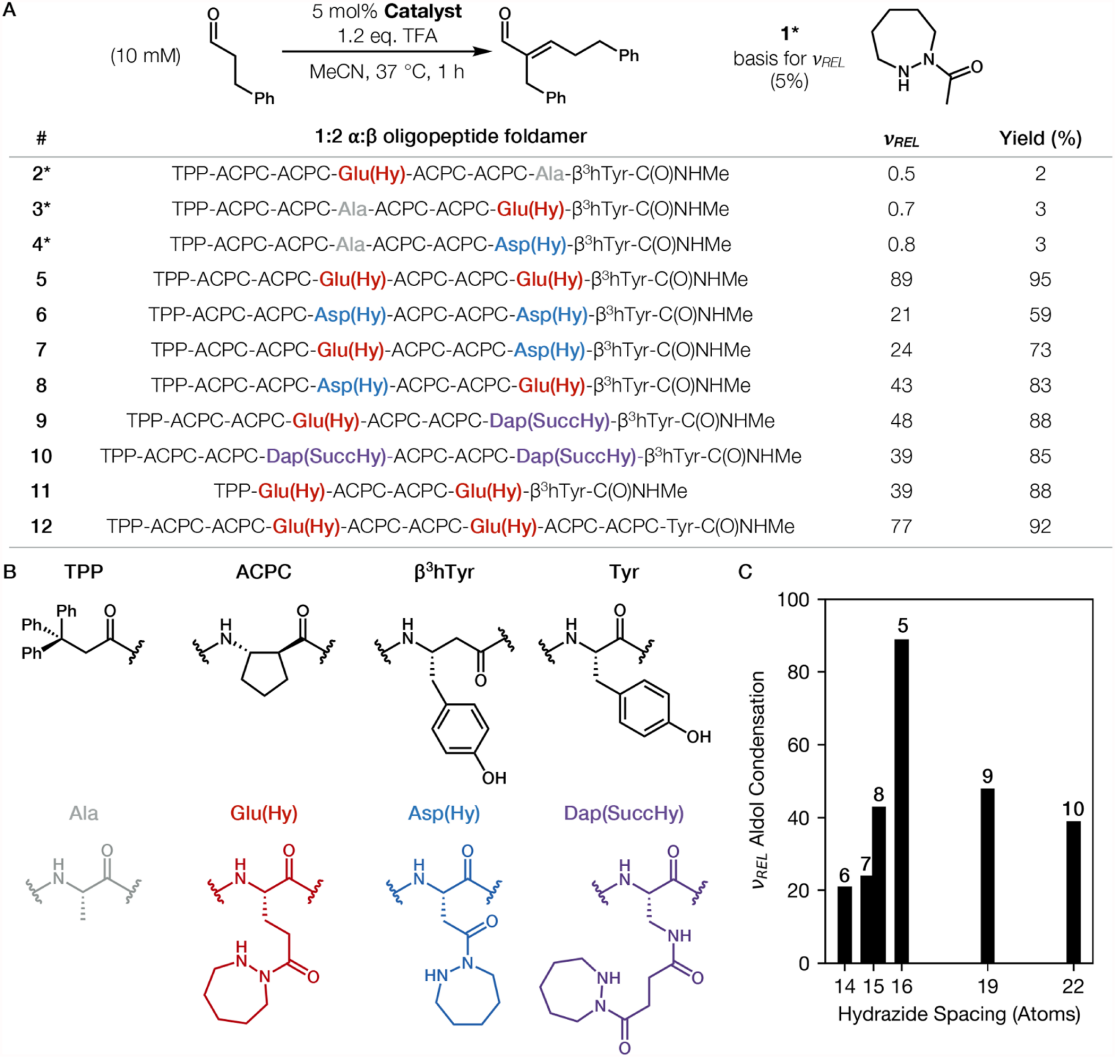
(A) Product yields after 1 h and relative initial rates of product formation (ν_*REL*_) determined by ^1^H NMR using an internal standard of aldol condensations catalyzed by monohydrazide **1** and 1:2 α:β oligopeptide foldamers **2**–**12** under the conditions shown. Key α-residues in the *i*,*i*+3 spacing are highlighted in each sequence. (B) Structures for α- and β-amino acid residues used in this study. (C) Summary of ν_*REL*_ as a function of the number of atoms in the spacer between hydrazide units. Labels for α/β-peptides **5**–**10** are above the corresponding bars in the graph. (*) = 10 mol % catalyst used.

When 10 mM aldehyde substrate and 1.2 equiv. trifluoroacetic acid (TFA) in acetonitrile at 37 °C were combined with 1 mM (10 mol %) hydrazide **1**, the aldol condensation yield was 5% after 1 h. Three α/β-peptides, **2**, **3** and **4**, were evaluated to determine the effect on catalytic activity of incorporating a single hydrazide via a Glu­(Hy) or Asp­(Hy) residue into an α/β-peptide. The α/β-peptide isomer of **4** with Asp­(Hy) and Ala positions swapped could not be evaluated because it was not soluble in acetonitrile. In each case, incorporation of the hydrazide unit into a side chain slightly diminished catalytic activity relative to simple hydrazide **1** (ν_*REL*_ values of 0.5–0.8). These observations may indicate that the peptide scaffold slightly hinders approach of substrates to a hydrazide moiety in a side chain, relative to unencumbered hydrazide **1**. Alternatively, the peptide bulk may slightly hinder the approach of a peptide-bound enamine to a peptide-bound iminium. These very small effects were indistinguishable for the Glu­(Hy) and Asp­(Hy) residues.

α/β-Peptide **5** contains two hydrazide units that should be aligned in the helical conformation; this bifunctional catalyst was highly active (ν_*REL*_ = 89). While monohydrazides **1**-**4** provided very small product yields after 1 h under these conditions (2–5%), a nearly quantitative product yield (95%) was observed for bifunctional catalyst **5** after 1 h. The initial rate displayed an approximately first order dependence on α/β-peptide **5**, which is consistent with a mechanism involving bifunctional catalysis (Figure S11). We note that if α/β-peptide **2**, **3** or **4** rather than **1** were used for normalization, ν_*REL*_ for **5** would be 178, 127, or 111. Thus, relative to the peptide monohydrazides, the bifunctional α/β-peptide displayed very high catalytic activity. Additionally, **5** was significantly more catalytically active than α/β-peptides with amine–amine or hydrazide-amine reactive diads (Table S9).

We compared **5** with α/β-peptides containing Asp­(Hy) or Dap­(SuccHy) residues in place of Glu­(Hy) to determine how catalytic activity is influenced by varying the length of the side chains bearing the reactive hydrazides, which varies the total number of atoms between the hydrazide units. Increasing side chain length in this series necessarily increases the flexibility of the linking segment, because the number of single bonds in the linker is increased. The results show that **5** is the most effective catalyst among the set of α/β-peptides we examined. Decreasing side chain length from Glu­(Hy) to Asp­(Hy), at both positions (**6**) or just one (**7** or **8**), caused a significant decrease in ν_*REL*_ (Figure 2A). There were corresponding declines in aldol condensation product yield after 1 h for **6**–**8** relative to **5**. We hypothesize that the shorter side chains of the Asp­(Hy) residues, relative to Glu­(Hy), result in a less favorable intramolecular approach of the enamine and iminium species in **6–8** relative to **5**, which disfavors carbon–carbon bond formation for **6–8** relative to **5**. In our previous study of dihydrazides containing flexible linkers, we observed a comparable decline in catalytic activity when the 10-methylene linker was replaced with a shorter linker.^36^

Increasing side chain length, by replacing one or both Glu­(Hy) with Dap­(SuccHy), caused a decline in catalytic activity relative to **5**: ν_*REL*_ = 48 for **9**, with one Dap­(SuccHy) residue, and ν_*REL*_ = 39 for **10**, with two Dap­(SuccHy) residues. Our data indicate that there is an optimal spacing between the hydrazide units (Figure 2C), at or near the linker length of **5**. As the number of atoms between the two hydrazides increases beyond this optimum, the decline in ν_*REL*_ presumably reflects an increasing entropic cost to adoption of conformations that bring the hydrazide groups into proximity. We previously observed an optimal spacing between the reactive groups among dihydrazides with polymethylene linkers (10 methylenes in that system).^36^

To probe the contribution of the helical backbone conformation to catalytic efficacy, we examined analogues of α/β-peptide **5** that contained fewer or more residues, **11** and **12**, while retaining the Glu­(Hy) diad with *i*,*i*+3 spacing. Helix formation is subject to length-dependent cooperativity among conventional peptides (entirely α residues)^61^ and peptides containing β residues.^62,63^ Thus, for peptides with similar compositions, increasing peptide length should increase the extent of helix population. α/β-Peptide **11** contains two fewer cyclic β residues than does **5**, and **11** was a less active catalyst relative to **5** (ν_*REL*_ = 39 vs 89). These data are consistent with the expectation that population of the helical conformation is lower for the shorter peptide; most nonhelical conformations presumably do not orient the two hydrazide units in a manner that promotes the aldol condensation. α/β-Peptide **12** contains two more cyclic β residues relative to **5**; however, this increase in peptide length had relatively little effect on catalytic activity (ν_*REL*_ = 77 vs 89). This observation could indicate that population of the helical conformation is very high for **5** in acetonitrile, and that increasing α/β-peptide length therefore cannot significantly enhance the extent of helix formation.

As a complementary approach to assessing the role of conformational preorganization provided by the α/β-peptide backbone in terms of reactivity, we compared the most effective α/β-peptide catalyst, **5**, with previously described simple dihydrazide catalysts (Figure 3). The conditions employed in the previous study were slightly different from those used here for the α/β-peptides, and selected simple dihydrazides were therefore reevaluated under the current conditions.^64^ As with the data in Figure 2, ν_*REL*_ is based on normalization to the initial rate observed with monohydrazide **1**. Data for only two polymethylene-linked dihydrazides are shown. Compound **13**, with a 10-methylene linker, was the most active catalyst among the set we previously evaluated.^36^ Compound **14**, with a 16-methylene linker, has the same number of atoms (and therefore bonds) between the two hydrazide moieties as are found in peptide **5**.

**Figure 3:**
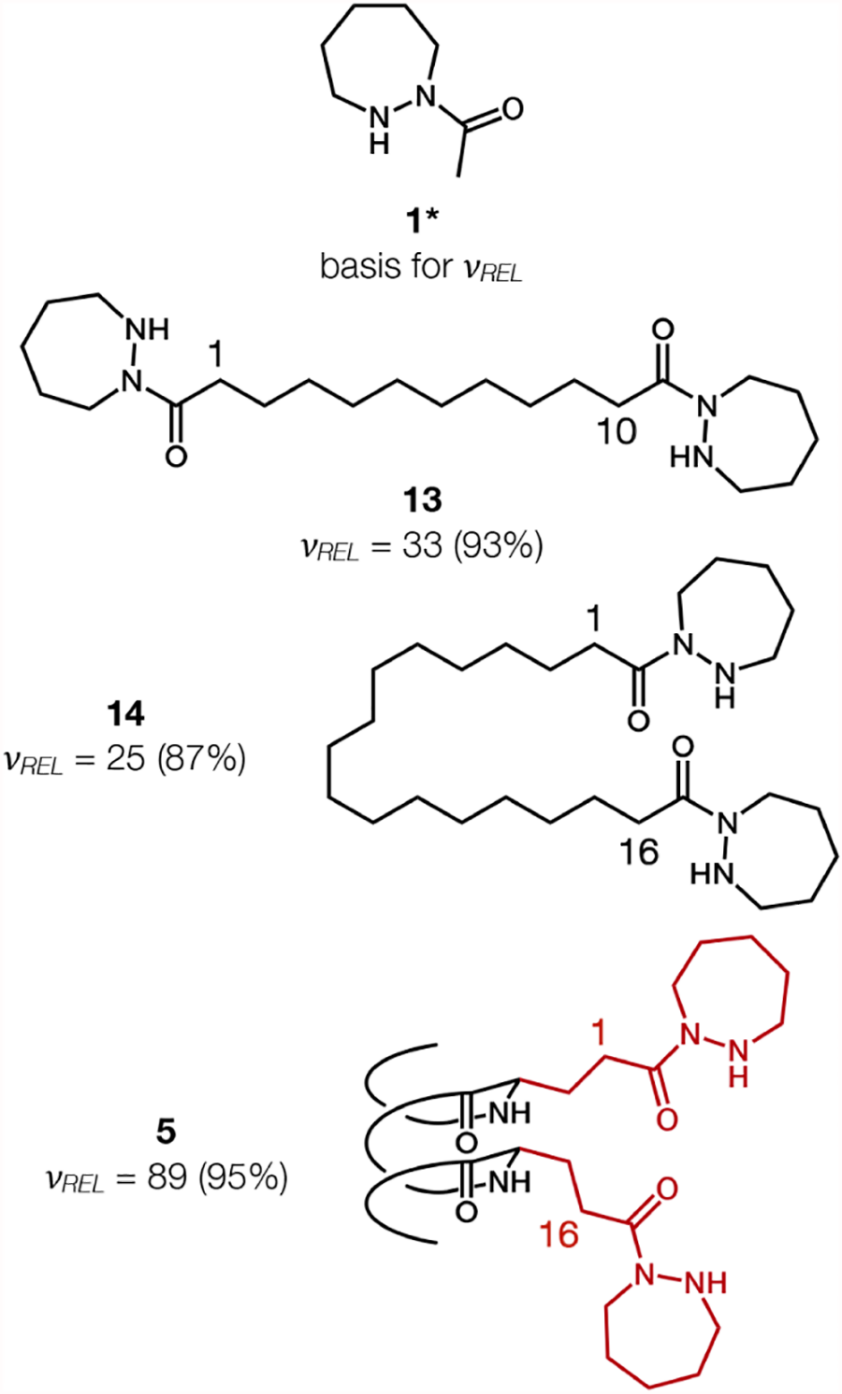
Product yields after 1 h and relative initial rates of product formation (ν_*REL*_) determined by ^1^H NMR using an internal standard for aldol condensations catalyzed by **1**, **13**, **14**, or **5** in the presence of 1.2 equiv of TFA. (*) = 10 mol % catalyst used.

These comparisons show that the helical α/β-peptide scaffold enhances catalytic activity relative to more flexible linkers, although this enhancement is modest. Relative to the most active among the simple dihydrazides (**13**), α/β-peptide **5** displays a ∼2.5-fold improvement in ν_*REL*_. Relative to a simple dihydrazide with a linker containing the same number of atoms (**14**), α/β-peptide **5** displays a ∼3.5-fold improvement in ν_*REL*_. The data in Figure 4 show that the modest advantage of α/β-peptide **5** relative to simple dihydrazide **13** for catalysis of the intermolecular aldol condensation is maintained for catalysis of an intramolecular aldol condensation that leads to formation of a 15-membered ring.

**Figure 4:**
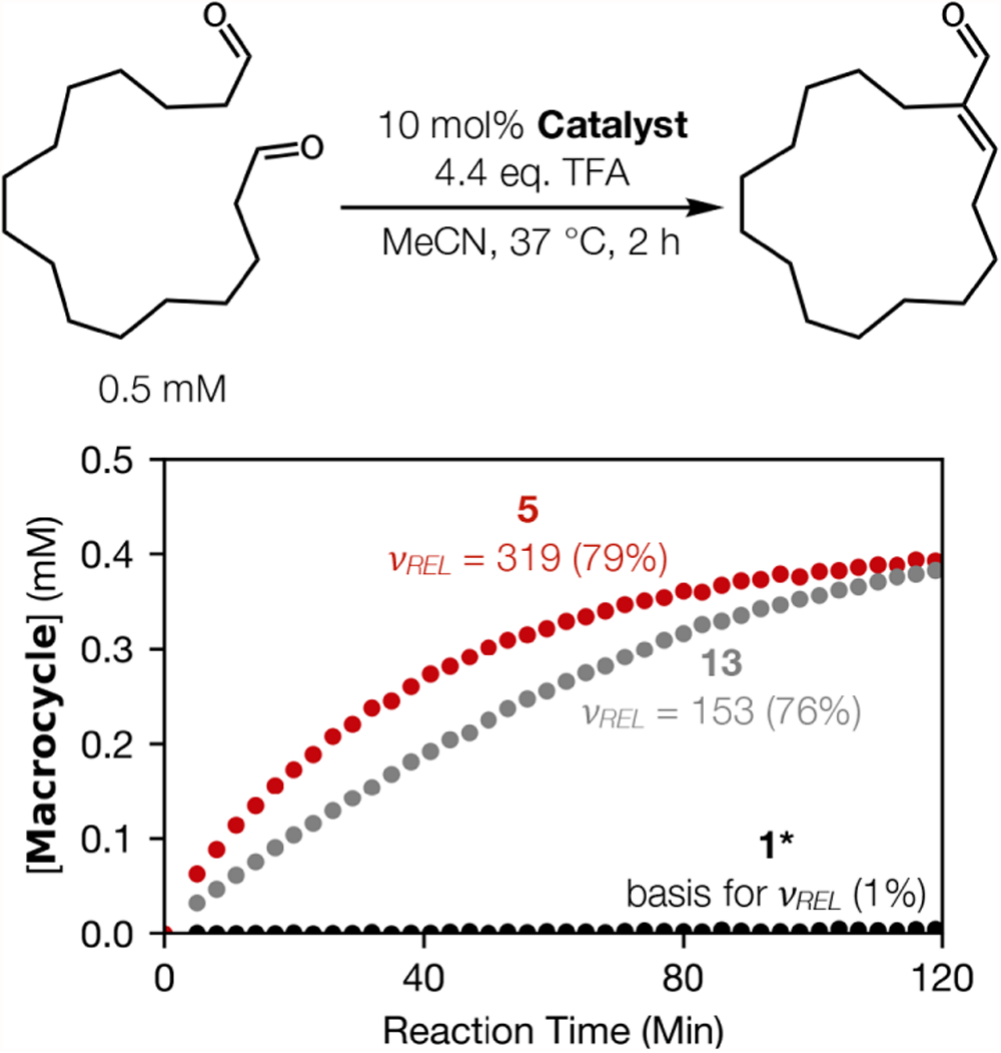
Reaction profiles showing product concentration as a function of time, product yields after 2 h and relative initial rates of product formation (ν_*REL*_) measured by ^1^H NMR using an internal standard for macrocyclization reactions catalyzed by **1**, **5**, or **13** under the conditions shown. (*) = 20 mol % catalyst used.

Short α/β-peptide **11** is slightly more effective as a catalyst than simple dihydrazide **14**, which has the same number of bonds between the hydrazide groups (ν_*REL*_ 39 vs 25). This similarity in reactivity supports the conclusion that the short peptide is probably not highly folded in acetonitrile, which would mean that the peptidic backbone of **11** does little to preorganize the reactive diad beyond covalently connecting the two hydrazides.

### Structural Analysis

Our interest in peptides with the 1:2 α:β pattern was motivated by previously reported crystal structures of α/β-peptides containing α-amino acid residues without functionalized side chains (mostly alanine and amino-isobutyric acid).^55,56,65,66^ To gain further insight on the contributions of the peptide scaffold to the catalytic properties of α/β-peptides with a hydrazide diad, we crystallized and structurally characterized peptide catalysts **5**, **11**, and **12**.

Treatment of the TFA salt of α/β-peptide **5**, **11**, or **12**, which had been isolated via HPLC, with saturated aqueous sodium bicarbonate yielded free amine forms of these peptides. Preparation of a saturated solution of free amine peptide **5** or **12** in acetonitrile at ∼60 °C followed by slow cooling to room temperature yielded crystals of each compound or a solvate. Single crystals of **11** for X-ray analysis were grown by slow diffusion of diethyl ether into a saturated solution of free amine **11** in dichloromethane.

Selected crystallographic parameters for α/β-peptides **5**, **11**, and **12** are presented in Table 1.^67^ The crystal structures for the α/β-peptide catalysts are of lower quality than is typical for small-molecule single-crystal X-ray analysis. The Supporting Information contains the relevant structure refinement details.

**Table 1:** Selected Crystal and Diffraction Parameters for α/β-Peptides **5**, **11**, and **12** ^a^

α/β-peptide	**5**	**11**	**12**
Empirical Formula	C_76_H_102_N_12_O_11_·solvent	C_64_H_84_N_10_O_9_·solvent	C_87_H_118_N_14_O_13_·solvent
M (g/mol)	1359.69	1137.41	1570.95
Crystal System	triclinic	orthorhombic	orthorhombic
Space Group	P1	*P*2_1_2_1_2	*P*2_1_2_1_2_1_
*a* (Å)	13.831(2)	27.316(9)	24.9460(6)
*b* (Å)	14.780(2)	27.870(7)	28.2854(6)
*c* (Å)	50.433(10)	9.881(3)	36.9128(9)
α (°)	90.670(10)	90	90
β (°)	97.195(10)	90	90
γ (°)	111.175(14)	90	90
*V* (Å^3^)	9519(3)	7522(4)	26046(10)
Z	5	4	12
μ(Cu Kα) (mm^–1^)	0.647	0.546	0.660
*Dcalc* (g/cm^3^)	1.186	1.004	1.202
2Θ range for data collection	5.308° to 158.948°	7.208° to 89.18°	3.936° to 100.86°
Reflections (measured)	366837	39713	205089
Reflections (unique)	77295 (R_int_ = 0.0680, R_sigma_ = 0.0511)	591 (R_int_ = 0.0881, R_sigma_ = 0.0511)	27098 (R_int_ = 0.0655, R_sigma_ = 0.0455)
R_1_ (I > 2σ(I))	0.0767	0.1029	0.1221
wR_2_ (all data)	0.2111	0.3319	0.3465

a Data collection *T* = 100(1) K and λ = 1.54178 for all peptides.

Extrapolation from crystal structures to solution behavior for inherently flexible molecules must be guided by caution. Nevertheless, this set of structures is helpful in framing our design hypothesis that the 1:2 α:β backbone favors a helix that aligns hydrazide side chains with *i*,*i*+3 spacing in the sequence. In addition, these structures collectively offer a perspective on the conformational flexibility of these α/β-peptides that may be manifested in solution.

In the crystal structure of **5**·solvent, Figure 5, there are five symmetry-independent molecules of α/β-peptide heptamer **5** in the asymmetric unit. Each molecule adopts a helical conformation that aligns the two Glu­(Hy) residues along one side of the helix. Four of the five conformations contain the maximum number of six C=O (*i*)···H—N (*i*+3) hydrogen bonds that is possible for the fully helical α/β-peptide heptamer. The remaining conformation has one fewer C=O (*i*)···H—N (*i*+3) hydrogen bond because of a deviation from helicity near the C-terminus. For the geometry of **5** depicted in the upper inset of Figure 5B, the α carbons of the Glu­(Hy) residues are separated by 5.916(10) Å. In the five independent molecules of **5** the distances between the α-carbons of Glu­(hy) residues ranged between 5.776(9)–6.193(9) Å. Thus, the helical α/β-peptide scaffold aligns the *i*,*i*+3-spaced α residues despite small conformational variations in the peptide backbone.

**Figure 5:**
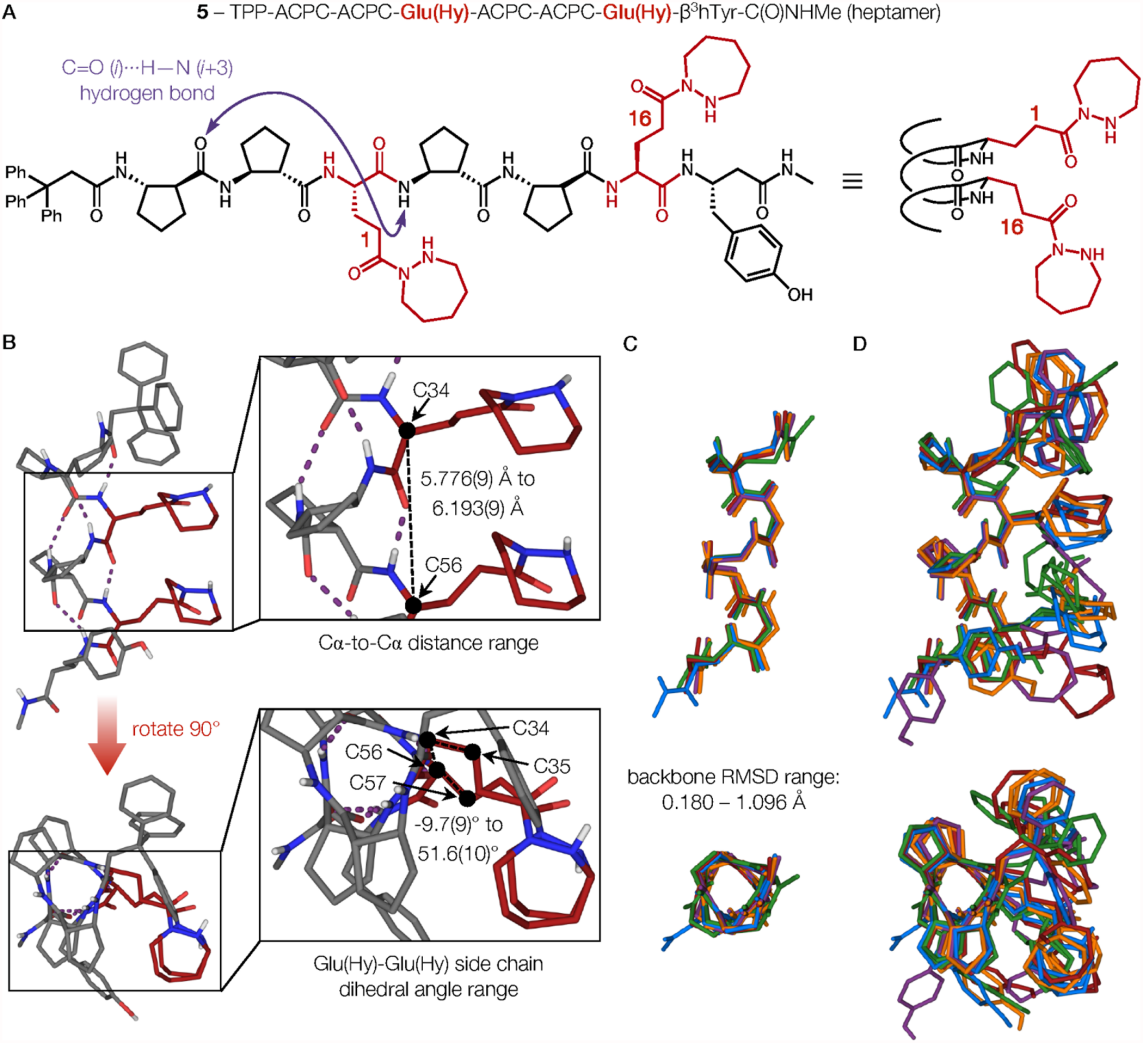
(A) Chemical structure drawing of heptamer **5**. The purple arrow indicates the C=O (*i*)···H—N (*i*+3) hydrogen bond interaction. (B) Crystal structure of a representative conformer of **5** (**5-C**) depicting the observed ranges for distances between α-carbons (Cα) C34–C56 and dihedral angles between Glu­(Hy) side chain atoms C57–C56–C34–C35. Glu­(Hy) residues are highlighted in red. Dashed lines (purple) indicate C=O (*i*)···H—N (*i*+3) hydrogen bonds. (C) Superposition of the five experimental geometries of conformer **5** with side chains, N-terminal trityl group, and H atoms omitted. (D) Superimposed structures of the five conformers of heptamer **5**.

An additional perspective on backbone-level variation among the five independent conformations of α/β-peptide **5** is provided by Figure 5C. The backbones of the five conformers are superimposed, highlighting the similarity among the five molecules at the backbone level. Quantitative pairwise comparisons among the structures are provided in the Supporting Information. The RMSD values derived from each possible pairwise backbone comparison range between 0.180 to 1.096 Å, which indicates that the conformations are similar but distinct.

In contrast to the backbone consistency among the five conformations in the crystal of **5**, the Glu­(Hy) side chains displayed considerable orientational variety among the symmetry-independent α/β-peptide molecules. We characterized this variation by using the bond between the α-carbon and the β-carbon (first carbon in the side chain) of each Glu­(Hy) residue to define a vector. For each independent conformation of **5**, the angle between the two Cα-Cβ vectors, when the structure was viewed down the helical axis, was defined as the “side chain dihedral angle” (Figure 5B, lower inset). These dihedral angles, which ranged from −9.7(9)° to 51.6(10)°, collectively highlight the diversity of hydrazide diad geometries that can be achieved without large variations in the α/β-peptide helix backbone. Further insight on the variation among Glu­(Hy) side chain positions across the five conformations in the crystals of **5** was provided by superimposing of all symmetry-independent molecules, including the side chains (Figure 5D).

The crystal structure of α/β-peptide pentamer **11** is shown in Figure 6. Reactivity data presented above showed that pentamer **11** is somewhat less catalytically effective relative to heptamer **5**, and we speculated that the pentamer might be less conformationally stable (less helical) relative to the heptamer. The crystal structure of pentamer **11** contained only one symmetry-independent conformer, which featured a helical backbone but exhibited only two of the four possible C=O (*i*)···H—N (*i*+3) hydrogen bonding interactions. An additional hydrogen bonding interaction between a hydrazide unit and the backbone was present. The distance between Glu­(Hy) residue α-carbons was 5.684(19) Å, shorter than any of the distances measured among the five conformers in the crystal of **5**. The side chain dihedral angle was 97.7(12)° for the lone conformer of **11**, which is well outside the range of values observed among the conformers of **5**. The structural comparison between **11** and **5** supports the conclusion we drew based on differences in catalytic activity between these two α/β-peptides, i.e., that the pentapeptide backbone may be too short to provide significant conformational preorganization of the hydrazide diad.

**Figure 6:**
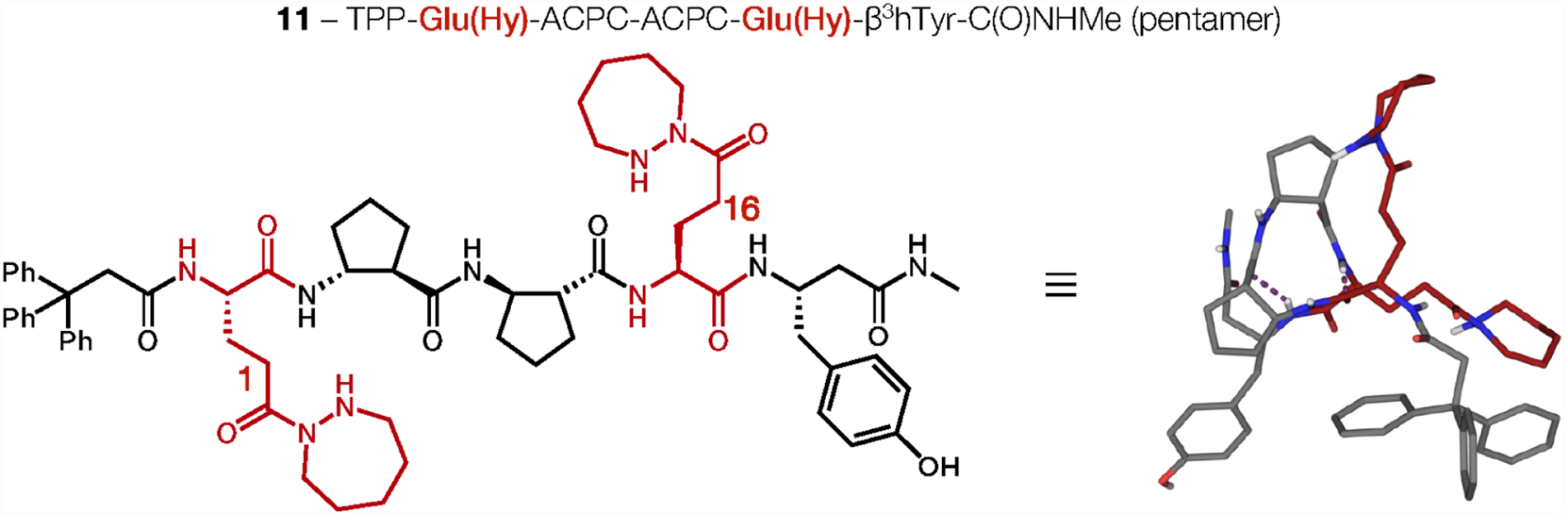
Chemical structure drawing and crystal structure viewed along the helical axis of pentamer **11**. Glu­(Hy) residues are highlighted in red. Dashed lines (purple) indicate C=O (*i*)···H—N (*i*+3) hydrogen bonds.

Conformers of α/β-peptide nonamer **12** obtained by single-crystal X-ray crystallographic analysis are presented in Figure 7. The asymmetric unit contains three symmetry-independent molecules of **12**. Each conformer is helical with the Glu­(Hy) diad located on one side of the helix. Two of the three conformers contained eight C=O (*i*)···H—N (*i*+3) hydrogen bonding interactions, the maximum number possible for the nonamer. The third conformer had the C=O (*i*)···H—N (*i*+3) hydrogen bonding pattern interrupted by a Glu­(Hy) C=O (hydrazide)···H—N (backbone) hydrogen bond. Distances between the Glu­(Hy) α-carbons in each conformer were between 5.32(2) and 6.01(2) Å, a larger range than was observed for heptamer **5**. Variation among the three α/β-peptide backbones in the crystal of **12** was larger than among the five backbones in the crystal of **5**. For nonamer **12**, pairwise RMSD values for the backbones ranged from 0.925 to 4.619 Å (Figure 7C). The larger backbone variation evident in the crystal of **12** relative to the crystal of **5** was paralleled by a larger variation in arrangement of the Glu­(Hy) side chains in the crystal of **12**. The side chain dihedral angles among the three conformers of **12** ranged from −54.0 (17)° to 35.6(13)°. Superimposition of three of the solid-state conformers of **12** (Figure 7D) provides a visual perspective on the side chain variations.

**Figure 7:**
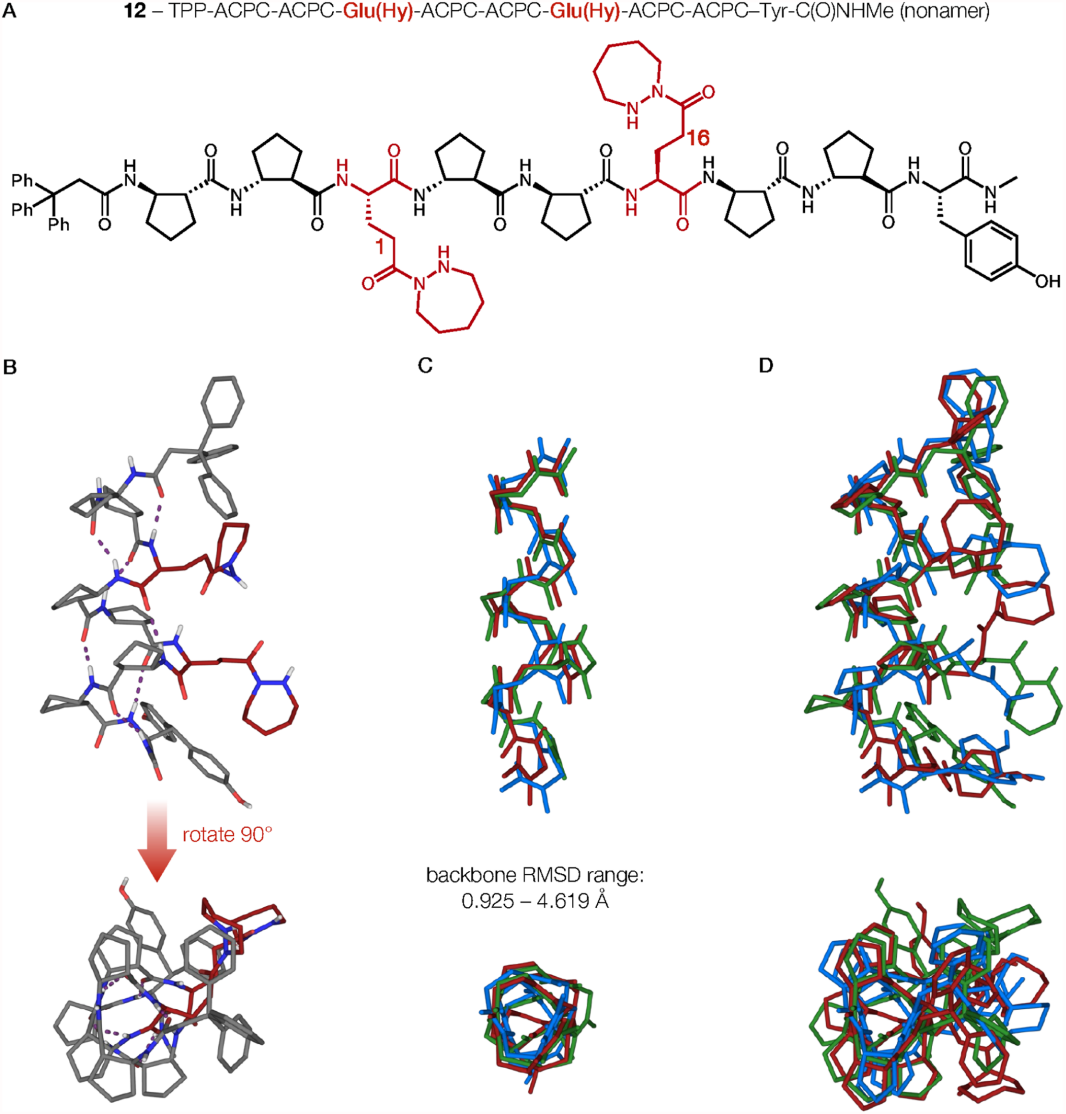
(A) Chemical structure drawing of nonamer **12**. (B) Crystal structure of one conformer of **12** (**12–1**). Glu­(Hy) residues are highlighted in red. Dashed lines (purple) indicate C=O (*i*)···H—N (*i*+3) hydrogen bonds. (C) Superposition of the three experimental geometries of conformer **12** with side chains, N-terminal trityl group, and H atoms omitted. (D) Superimposed structures of the three conformers of nonamer **12**.

The reactivity data in Figures 2 and 3 show that α/β-peptides **5**, **11**, and **12** were comparable or superior to flexible dihydrazide **13** as catalysts of the aldol condensation. We used the set of crystallographically independent conformers (nine altogether) observed in the three α/β-peptide structures to assess the range of hydrazide diad juxtapositions accessible to the common backbone. For this comparison, we used the distances between the carbonyl carbons of the two hydrazide groups (Figure 8A). These distances varied between 5.19(3) and 7.840(16) Å.

**Figure 8:**
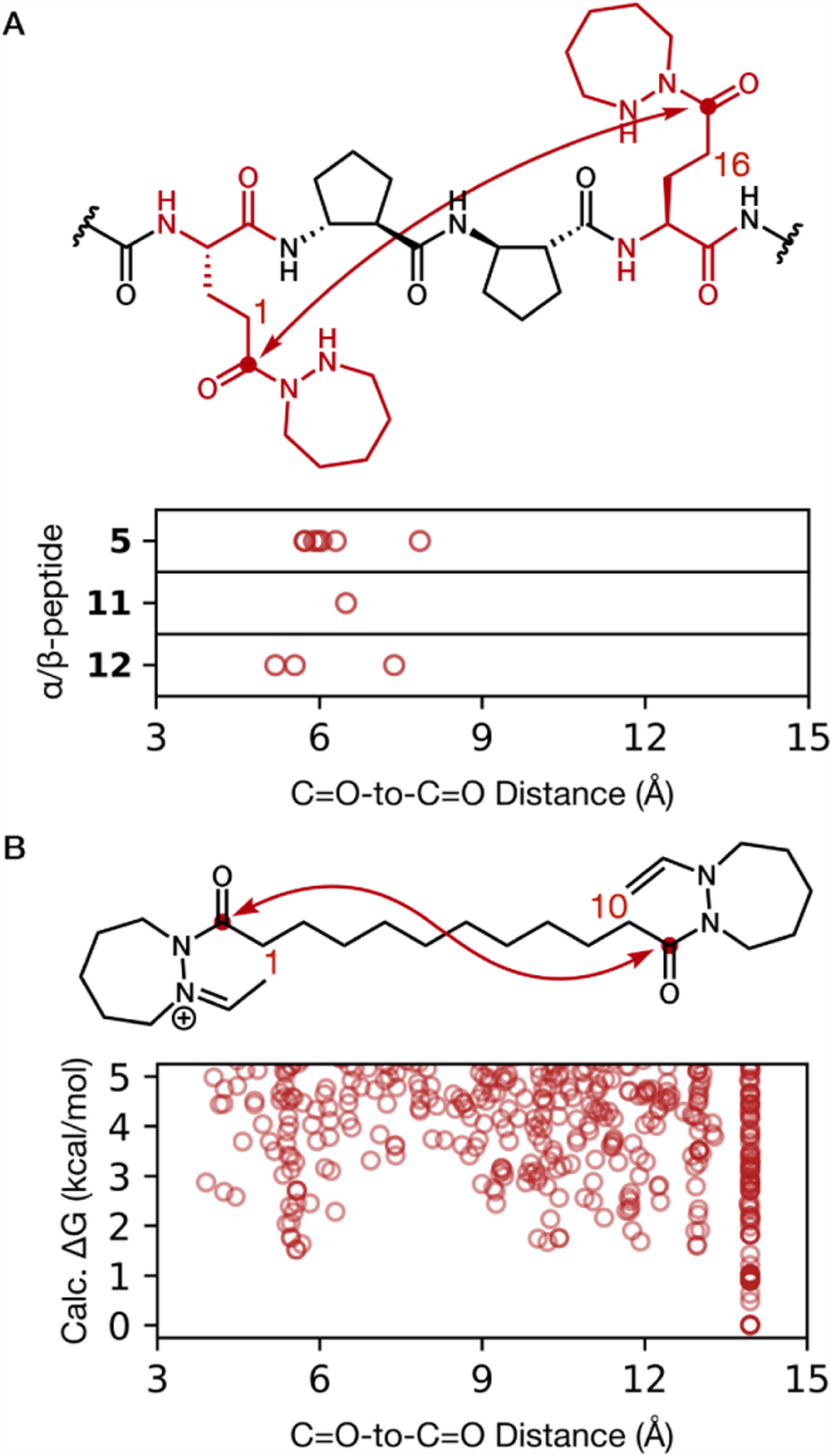
(A) Range of C=O-to-C=O distances observed in the crystal structures for α/β-peptides **5**, **11**, and **12**. (B) Calculated relative free energies and C=O-to-C=O distances for conformers of simple dihydrazide derivative **13’**.

This analysis provided a basis for comparison with flexible dihydrazide **13**. In this case, we turned to a computational approach. This effort employed a derivative of **13** (designated **13’**) in which one hydrazide has formed an enamine with acetaldehyde and the other hydrazide has formed an iminium with acetaldehyde. We assume that a comparable intermediate, derived from hydrocinnamaldehyde, is involved in bifunctional catalysis of the aldol condensation by α/β-peptide **5** or flexible dihydrazide **13**.

A large set of conformers of **13’** was generated using RDkit^68^ and optimized at the M06-2X/def2-TZVP/CPCM = MeCN level. Conformers that displayed negative vibrational modes were removed from the ensemble. Full computational methods are provided in the Supporting Information. For each conformer, we measured the distance between the carbonyl carbons to compare with crystal structures of **5**, **11**, and **12**. The computational results suggested a small energetic preference (∼1.5 kcal/mol) for conformers of **13’** with extended polymethylene tethers (C=O-to-C=O distance ≈ 14 Å) relative to conformers with C=O-to-C=O distances in the range (5.19(3)–7.840(16) Å) observed for α/β-peptides **5**, **11**, and **12** (Figure 8B). The conformer with the lowest free energy had a fully extended polymethylene linker with all linker CC–CC torsion angles ∼180° and a C=O-to-C=O distance of 13.95 Å. A similar energetic preference for extended alkane conformations was observed when conformers were evaluated at the B3YLP/6-31G­(d,p)/CPCM = MeCN level of theory. These findings are consistent with previous high-level computational investigations of medium-length *n*-alkane conformational preferences.^69–71^ Thus, unlike **5**, **11**, and **12**, compound **13’** is not predicted to have a propensity to adopt conformations that would bring the iminium and enamine moieties together.

Comparing C=O-to-C=O distances obtained from crystallographic data for α/β-peptides **5**, **11**, and **12** with analogous distances derived from computational analysis of **13’** does not allow us to draw firm conclusions about the conformational properties of these molecules in solution. Nevertheless, this comparison offers evidence that the α/β-peptides are predisposed to bring the two hydrazide units near one another, as envisioned in their design, but that significant latitude remains in terms of the juxtaposition of the two hydrazide units of the α/β-peptides in solution. In contrast, **13’** is not predisposed to bring the two hydrazides into proximity, although proximity is possible.

## Conclusions

Our previous report showed that simple, flexible dihydrazides, such as **13** or **14**, could be substantially more effective than a monohydrazide for catalysis of the aldol condensation.^36^ These findings suggested that a bifunctional catalytic mechanism could be significantly enhanced simply by covalent linkage of the two reactive sites, without any effort to favor spatial proximity of those reactive sites via conformational preorganization of the linker. Relatively long linkers were necessary for optimal catalytic activity; smaller homologues of **13** were poorer catalysts.

The present study has built on this precedent to assess linkers that are conformationally predisposed to bring two hydrazides into proximity. Preorganization was implemented by using an α/β-peptide to link the hydrazide units. The 1:2 α:β backbone repeat favors a specific helical conformation, and proper hydrazide placement within the sequence caused alignment of the two groups upon helix formation. The α/β-peptide scaffold provided a modest catalytic enhancement relative to a nonpreorganized linker. Under the conditions we used for our comparisons, α/β-peptide dihydrazide **5** displayed a ∼3.5-fold improvement in ν_*REL*_ when compared with flexible dihydrazide **14**; each has 16 atoms between the two hydrazide units.

Catalyst **5** appears to represent the optimal scaffold for the hydrazide diad in this α/β-peptide series. Catalytic activity declined with shorter side chains linking the hydrazides to the peptide backbone (peptides **6**–**8**) and with longer side chains (peptides **9**–**10**). An optimum in linker length was previously observed among the simple dihydrazides: increasing or decreasing the linker relative to **13** caused a decline in ν_*REL*_.^36^ These trends may suggest that a minimum covalent separation between the hydrazide units is necessary to achieve the optimal geometry for carbon–carbon bond formation between the enamine and iminium moieties, and that elongation beyond that minimum results in a larger entropic barrier to the bond-forming step. Incorporation of a helical turn into the linker, by progressing from the simple dihydrazides to the α/β-peptide dihydrazides, caused an increase in the number of atoms in the optimal linker (16 for the α/β-peptide series introduced here vs 10 for the simpler dihydrazides studied previously). However, the more highly engineered α/β-peptide linkers provided an enhancement in catalytic activity of only ∼2.5-fold in terms of ν_*REL*_ under the conditions employed in our study, based on the comparison α/β-peptide **5** vs flexible dihydrazide **13**. We subjectively characterize this enhancement as modest because the difference between α/β-peptide **5** and flexible dihydrazide **13** is an order of magnitude smaller than the difference between flexible dihydrazide and a simple monohydrazide.

Diverse efforts have been pursued to coordinate the actions of two or more reactive groups via a covalent linker.^32,44,53,57,72–79^ Few of these studies have evaluated the contribution of linker preorganization to catalytic efficacy.

The previous study of simple dihydrazide catalysts^36^ and the current study of α/β-peptide dihydrazides, together, raise the question: why does a conformationally preorganized linker not provide a larger boost to catalytic activity? One possibility is that more substantial increases in activity would require greater linker preorganization, i.e., a more rigid linker that enforces a juxtaposition of hydrazide units optimized for the rate-determining step in the aldol condensation. We speculate, however, that additional rigidity would not provide significant further catalytic benefit. The aldol condensation involves many fundamental steps,^80^ and even if it were possible to rigidify a bifunctional catalyst for one step (e.g., carbon–carbon bond formation), the resulting conformational restrictions could hinder other steps.

It is noteworthy that enzymes are inherently flexible molecules, but they can nevertheless achieve very large rate accelerations.^81^ Spatial organization of reactive groups appears to be one source of enzymatic catalysis, but our dihydrazide findings raise the possibility that simple covalent connection between reactive groups provides much of the benefit accessible for a bifunctional catalyst. Perhaps the benefit of a preorganized scaffold would be larger if the number of catalyst–substrate contacts required to facilitate the reaction were increased. This possibility could be explored with trifunctional catalysts. Perhaps more effective catalysis requires desolvation in addition to reactive group preorganization. The results shared here provide a basis for future studies that explore these possibilities.

## Supplementary Material



## Notes

Synthetic methods, experimental procedures, kinetics data, crystallography information, computational methods and characterization data (PDF)
