# Supplementary material for: Bifunctional Catalysis of Aldol Reactions by Foldamer Dihydrazides: Assessment of Conformational Preorganization
Source: J Am Chem Soc. 2025 Sep 4;147(37):33932–43. doi: 10.1021/jacs.5c10914 (PMC12503358; doi:10.1021/jacs.5c10914)
Supplement: Supplementary file 1 [file ja5c10914_si_001.pdf]

Bifunctional Catalysis of Aldol Reactions by Foldamer Dihydrazides: Assessment of Conformational Preorganization

Philip P. Lampkin, Kyana M. Sanders, Leah C. Garman, Ilia A. Guzei and Samuel H. Gellman*

Department of Chemistry, University of Wisconsin–Madison,
1101 University Avenue, Madison, WI 53706, United States

Email: gellman@chem.wisc.edu

Supporting information

Table of Contents

1. **General Information** **S3**
2. **Synthetic Information** **S4**
   - **2.1** - Fmoc-L-Glu(HyBoc)-OH (**S5**)
   - **2.2** - Fmoc-L-Asp(HyBoc)-OH (**S7**)
   - **2.3** - HO-Succ(HyBoc) (**S10**)
   - **2.4** - α/β-Peptide Foldamers **2**-**12** and **15**-**27**
3. **Experimental Information** **S13**
   - **3.1** - Homoaldol Condensation Reaction Monitoring
   - **3.2** - Macrocyclization Reaction Monitoring
   - **3.3** - Other Experiments
     - **3.3.1** - Dependence of Initial Rate on TFA Loading
     - **3.3.2** - Dependence of Initial Rate on H_2O Loading
     - **3.3.3** - Dependence of Initial Rate on [**5**]
     - **3.3.4** - Dependence of Initial Rate on Dihydrazide α/β-Peptides Sequence Order
     - **3.3.5** - Reactivities of α/β-Peptides Containing 1° and 2° Amines
     - **3.3.6** - Insoluble and Gel-Forming α/β-Peptides
4. **Reaction Profiles and Initial Rates Data** **S31**
   - **4.1** - Relative Rates and 1 Hour Yields Summary – Homoaldol
   - **4.2** - Reaction Profiles and Initial Rates for **1**-**21** – Homoaldol
   - **4.3** - Relative Rates and 2 Hour Yields Summary – Macrocyclization
   - **4.4** - Reaction Profiles for **1, 5** and **13** – Macrocyclization
5. **Crystallography Information** **S57**
   - **5.1** - Crystal Growth Method for **S10** (Slow Evaporation)
   - **5.2** - Crystal Growth Method for **5** and **12** (Slow Cooling)
   - **5.3** - Crystal Growth Method for **11** (Liquid-Liquid Diffusion)
   - **5.4** - Data Collection, structural solution and refinement for **S10**
   - **5.5** - Data Collection, structural solution and refinement for **5**
   - **5.6** - Data Collection, structural solution and refinement for **11**
   - **5.7** - Data Collection, structural solution and refinement for **12**
   - **5.8** - Crystal Structure-derived Geometric Parameters for **5**
   - **5.9** - Crystal Structure-derived Geometric Parameters for **11**
   - **5.10** - Crystal Structure-derived Geometric Parameters for **12**
   - **5.11** - Comparison of Geometric Parameters for **5**, **11** and **12**
6. **Computational Information** **S82**
   - **6.1** - DFT-derived Geometric Parameters for **13'** – B3LYP/6-31G(d,p)
   - **6.2** - DFT-derived Geometric Parameters for **13'** – M06-2X/def2-TZVP
7. **NMR Spectra, MS and UPLC Data for New Compounds** **S111**
8. **References** **S135**
9. **Instrumentation Funding Acknowledgements** **S138**

1. General Information

Reagents, amino acid derivatives and solvents were purchased from Ambeed, Chem-Impex International or Sigma-Aldrich and used as received unless noted. Solvents were HPLC-grade unless specified. All reactions and manipulations were carried out without any effort to exclude air or moisture unless stated. Glassware, reagents and solvents were stored on the benchtop unless otherwise stated. Thin-layer chromatography was carried out using SiliCycle SiliaPlate 250 µm extra hard layer 60 Å plates, and compounds were visualized using UV light or an iodine-silica chamber. Flash column chromatography was conducted using Silicycle SiliaFlash P60, 40-63 µm 60 Å silica. High-performance liquid chromatography (HPLC) was carried out using an Agilent 1260 Infinity II preparative LC UV-MSD system equipped with a 1290 Infinity II Preparative Open-Bed Sampler/Collector module and preparative Waters XSelect CSH C4 OBD column (130 Å, 5 µm, 19 mm X 250 mm). Analytical ultra performance liquid chromatography (UPLC) was carried out using a Waters Acquity H-Class UPLC equipped with an analytical Waters UPLC Protein BEH C4 column (300 Å, 1.7 µm, 2.1 mm X 100 mm). Solid-phase peptide synthesis was carried out using a CEM Mars II microwave reactor.

All ^{1}H and ^{13}C NMR spectra were measured using a Bruker NEO-500 NMR spectrometer equipped with a 5mm Prodigy-BBO probe. Kinetics experiments were conducted at 37°C inside the spectrometer. Probe temperature was calibrated prior to experiments using a neat ethylene glycol sample and the Bruker TopSpin "CalcTemp" program. Chemical shifts (δ) are reported in parts per million (ppm). All ^{1}H and ^{13}C NMR spectra are internally referenced to tetramethylsilane (δ(^{1}H) = 0.00 ppm, δ(^{13}C) = 0.00 ppm). Data for ^{1}H and ^{13}C NMR signal multiplicity are reported as s (singlet), d (doublet), t (triplet), q (quartet) or m (multiplet) and coupling constants (*J*) are reported in Hz. High resolution mass spectra were obtained using a Bruker IMPACT II MS.

Chemical Abbreviations:
Trifluoroacetic acid – TFA
Dimethylformamide – DMF
Hexafluorophosphate Azabenzotriazole Tetramethyl Uronium – HATU
N,N-Diisopropylethylamine – DIPEA
Ethyl Acetate – EtOAc
Methanol – MeOH
9-Fluorenylmethyloxycarbonyl – Fmoc
tert-Butyloxycarbonyl – Boc
Allyloxycarbonyl – Alloc
Dichloromethane – DCM
Acetonitrile – MeCN
1,2-Dichloroethane – DCE
Tetramethylsilane – TMS

2. Synthetic Information

Compounds **1**, **13**, **14** and 1,16-hexadecanedial were prepared as previously described.[1,2] Analytical data agreed with literature values. *tert*-Butyl 1,2-diazepane-1-carboxylate (**S1**) used in the synthesis of Fmoc-L-Glu(HyBoc)-OH (**S5**), Fmoc-L-Asp(HyBoc)-OH (**S7**) and HO-Succ(HyBoc) (**S10**) was purchased from Ambeed. Analytical data for **S1** sourced from Ambeed matched those of **S1** synthesized in-house.[1]

2.1 Fmoc-L-Glu(HyBoc)-OH (S5)

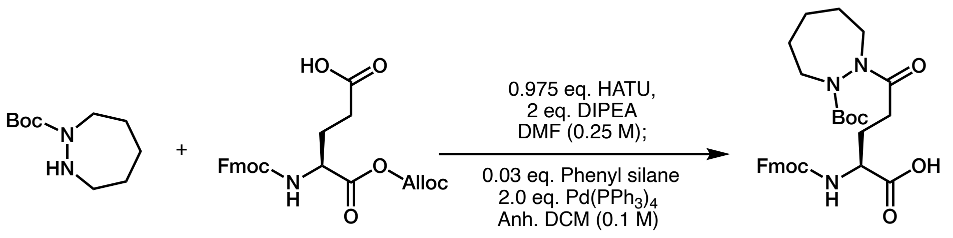

To a 250 mL round bottom flask containing a stir bar were added Fmoc-L-glutamic acid 1-allyl ester (**S4**) (2.9 g, 7.3 mmol), HATU (2.7 g, 7.12 mmol, 0.975 equiv.), biotech-grade DMF (29.0 mL [concentration of the reaction solution is 0.25 M relative to the amount of **S4**]), and DIPEA (2.5 mL, 14.6 mmol, 2 equiv.). The mixture was stirred vigorously for until all solids were dissolved, and then *tert*-Butyl 1,2-diazepane-1-carboxylate (**S1**) (1.5 g, 7.3 mmol, 1.0 equiv.) was added. The mixture was stirred for 24 h after, which it was diluted with EtOAc (150 mL), and the resulting solution was washed with brine (3 x 150 mL). The organic layer was separated and dried with anhydrous Na_2SO_4. The organic layer was then filtered into a 500 mL round bottom flask and concentrated *in vacuo*. The resulting crude residue was placed under a high vacuum for 24 h to remove residual moisture. The dried crude residue was then dissolved in anhydrous DCM (73 mL [concentration of the reaction solution is 0.1 M relative to the starting amount of **S4**]). To the resulting solution was added phenylsilane (1.8 mL, 14.6 mmol, 2.0 equiv.) followed by slow portionwise addition of tetrakis(triphenylphosphine)palladium(0) (0.25 g, 0.22 mmol, 0.03 equiv.). The solution was then placed under nitrogen atmosphere and left to stir. After 4 h, the resulting solution is concentrated *in vacuo* until a thick black residue was obtained. This residue was then dissolved in DCM (100 mL), and the solution was washed with brine (3 x 150 mL). The organic and aqueous layers were separated, and the organic layer was dried over anhydrous Na_2SO_4, filtered and concentrated *in vacuo*. The crude product was purified by flash column chromatography (0.0:1.0 EtOAc:pentanes to 1.0:0.0 EtOAc:pentanes to 0.75:0.25 EtOAc:MeOH mobile phase gradient) and dried under a high vacuum, yielding Fmoc-L-Glu(HyBoc)-OH (**S5**) as a clear foam (2.5 g, 4.5 mmol, 62% isolated yield).

Additional Synthetic Notes: Deprotection of the Alloc group generates significant CO_2 that will cause the reaction mixture to violently bump if tetrakis(triphenylphosphine)palladium(0) is added too rapidly. Trace tetrakis(triphenylphosphine)palladium(0) impurities in the product can be removed by dissolving the product in EtOAc, vigorously stirring with decolorizing carbon for 2 minutes, filtering the resulting mixture through a thick celite pad and washing the pad with EtOAc. Trace Pd impurities in the product were not observed to diminish peptide synthesis yield. The product can be obtained as a powder by dissolving the

product cleaned with decolorizing carbon in minimal EtOAc and precipitating it via dropwise addition of the solution to a beaker containing a rapidly stirring solution of pentanes followed by vacuum filtration.

Note on characterization of S5 by NMR: Amino acid **S5** exists as a mixture of several rotamers that slowly interconvert on NMR timescales. Only the expected mass of **S5** was observed by mass spectrometry. α/β-Peptides prepared with **S5** exhibited masses corresponding to incorporation of **S5**. Crystal structures of α/β-Peptides incorporating **S5** had residues the matched the proposed structure of Glu(Hy), a derivative of **S5**. Full NMR analysis of related monohydrazide and dihydrazide species are reported in reference 1. Due to the presence of several rotamers significantly hindering analysis of **S7** by NMR, only the shifts of ^{1}H and ^{13}C NMR resonances for **S5** are summarized below. ^{1}H and ^{13}C NMR spectra for **S5** are provided in section 7.

Fmoc-L-Glu(HyBoc)-OH (S5): ^{1}H NMR (500 MHz, $CDCl_3$): δ = 7.80-7.70 (m), 7.65-7.50 (m), 7.45-7.33 (m), 7.32-7.26 (m), 6.30-5.90 (m), 4.55-3.87 (m), 3.48-3.35 (m), 3.31-3.15 (m), 3.06-2.85 (m), 2.75-2.35 (m), 2.33-2.20 (m), 2.15-1.92 (m), 1.88-1.30 (m) ppm; ^{13}C NMR (125.725 MHz, $CDCl_3$): δ = 176.21, 175.74, 175.62, 174.63, 174.25, 174.11, 173.90, 156.31, 156.20, 154.44, 154.30, 154.16, 154.02, 143.92, 143.88, 143.72, 143.70, 141.25, 141.22, 127.69, 127.08, 125.24, 125.16, 119.94, 82.35, 82.03, 67.28, 53.89, 53.73, 53.66, 52.00, 50.43, 48.75, 48.45, 48.29, 47.04, 28.95, 28.20, 28.17, 26.58, 25.58 ppm; HR-ESI-MS: *m/z:* 550.2565 ($[M\text{-}H]^-$ calculated for $C_{30}H_{36}N_3O_7^-$: 550.2558).

2.2 Fmoc-L-Asp(HyBoc)-OH (**S7**)

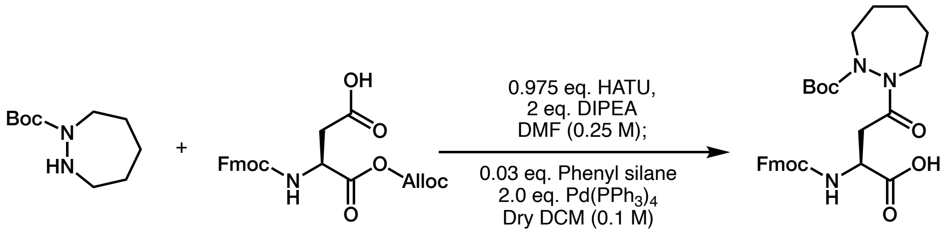

1 eq. **S1** **S6** **S7** (46%)

Fmoc-L-aspartic acid 1-allyl ester (**S6**) (3.6 g, 9.00 mmol) was converted into Fmoc-L-Asp(HyBoc)-OH (**S7**) according to the procedure given in section 2.1, yielding a white foam (2.2 g, 4.1 mmol, 46% isolated yield).

Note on characterization of S7 by NMR: Amino acid **S7** exists as a mixture of several rotamers that interconvert slowly on the NMR timescale. Only the expected mass of **S7** was observed by mass spectrometry. α/β-Peptides prepared with **S7** exhibited masses corresponding to incorporation of **S7**. Full NMR analysis of related monohydrazide and dihydrazide species are reported in reference 1. Due to the presence of several rotamers significantly hindering analysis of **S7** by NMR, only the shifts of ^{1}H and ^{13}C NMR resonances for **S7** are summarized below. ^{1}H and ^{13}C NMR spectra for **S7** are provided in section 7.

Fmoc-L-Glu(HyBoc)-OH (S7):

^{1}H NMR (500 MHz, $CDCl_3$): δ = 7.80-7.75 (m), 7.65-7.58 (m), 7.45-7.33 (m), 7.32-7.26 (m), 6.30-5.90 (m), 4.75-4.55 (m), 4.5-4.39 (m), 4.35-4.20 (m), 4.19-4.09 (m), 4.05-3.90 (m), 3.52-2.6 (m), 1.95-0.7 (m) ppm; ^{13}C NMR (125.725 MHz, $CDCl_3$): δ = 174.91, 174.47, 174.21, 173.57, 173.42, 156.00, 154.16, 153.91, 153.78, 153.63, 143.90, 143.73, 143.64, 127.76, 127.73, 127.12, 127.10, 127.08, 125.26, 125.15, 120.00, 82.56, 82.38, 82.26, 67.37, 67.32, 51.94, 50.36, 50.19, 50.03, 48.89, 47.07, 35.51, 35.00, 34.64, 29.72, 28.22, 28.10, 26.70, 26.57, 25.55, 25.46 ppm; HR-ESI-MS: *m/z:* 536.2405 ([*M*-H]$^{-}$ calculated for $C_{29}H_{34}N_3O_7^{-}$: 536.2402).

2.3 HO-Succ(HyBoc) (**S10**)

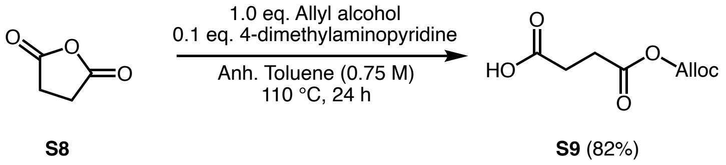

Succinic anhydride (**S8**, 6.0 g, 60 mmol) was converted to 1-(2-propen-1-yl)butanedioate (**S9**) according to a literature procedure,[3] yielding a clear oil (7.8 g, 49.2 mmol, 82% yield). Analytical data agreed with literature values.[3]

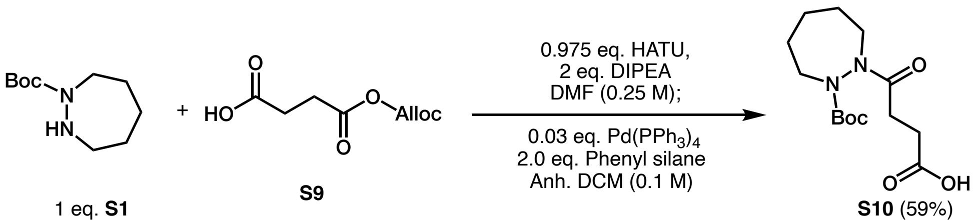

1-(2-Propen-1-yl) butanedioate (**S9**) (3.0 g, 14.8 mmol) was converted into HO-Succ(HyBoc) (**S10**) according to the procedure given in section 2.1, yielding a clear foam (2.6 g, 8.7 mmol, 59% isolated yield).

Note on characterization of S10 by NMR: Compound **S10** exists as a mixture of several rotamers that interconvert slowly on the NMR timescale. α/β-Peptides prepared with **S10** exhibited masses corresponding to incorporation of **S10**. A crystal structure of **S10** is described in section 5.4. Full NMR analysis of related monohydrazide and dihydrazide species are reported in reference 1. Due to the presence of several rotamers significantly hindering analysis of **S10** by NMR, only the shifts of ^{1}H and ^{13}C NMR resonances for **S10** are summarized below. ^{1}H and ^{13}C NMR spectra for **S10** are provided in section 7.

HO-Succ(HyBoc) (S10): ^{1}H NMR (500 MHz, $CDCl_3$): δ = 4.16-4.06 (m), 4.04-3.90 (m), 3.39-3.27 (m), 3.25-3.12 (m), 3.10-2.93 (m), 2.85-2.41 (m), 1.9-1.3 (m) ppm; ^{13}C NMR (125.725 MHz, $CDCl_3$): δ = 177.82, 173.98, 173.73, 154.25, 132.17, 132.13, 132.11, 132.09, 128.60, 128.51, 81.95, 81.79, 51.95, 50.27, 48.37, 48.02, 28.85, 28.74, 28.30, 28.20, 27.48, 27.07, 26.75, 26.64, 26.58, 25.75, 25.68 ppm; HR-ESI-MS: *m/z:* 299.1612 ([M-H]–) calculated for $C_{29}H_{34}N_3O_7^-$: 299.1612.

2.4 α/β-Peptide Foldamers **2-12** and **15-27**

Solid-phase Peptide Synthesis Preparation

Fmoc-protected α- and β-amino acids for each coupling step (100 µmol, 4.0 equiv. amino acid per coupling relative to loading of resin of 25 µmol) were weighed into individual 14 mL plastic centrifuge tubes. HATU coupling agent was weighed into a 50 mL plastic centrifuge tube, diluted with Biotech-grade DMF and shaken vigorously until all solids were dissolved to make a 0.09875 M solution. The HATU coupling agent solution was prepared the same day it was used. Fmoc-protected NovaBioChem methyl indole AM resin (25 µmol by amine loading, 0.67 mmol/g loading, 37.3 mg) was added to a 5 mL Torviq solid-phase peptide synthesis vessel equipped with a stir bar. The resin was swelled with ~3 mL DCM for 30 min. The DCM was aspirated from the vessel and the swelled resin was rinsed with 3 x 4 mL of Biotech-grade DMF. The resin was dried by aspiration for 2.5 minutes, after which ~3 mL of 20% v/v piperidine in ACS-grade DMF was added to the reaction vessel. The vessel was placed in a microwave reactor, and a temperature probe was placed in the reaction vessel solution. The microwave Fmoc group deprotection program was initiated (ramp to 80°C over 2 min, hold at 80°C for 4 min). After program completion, the vessel was removed from the microwave, the solution was aspirated from the vessel, the deprotected resin was washed five times with 4 mL ACS-grade DMF and the resin was dried by aspiration for ~2.5 min.

Coupling of Amino Acids

Powdered Fmoc-protected amino acids were activated for coupling steps by adding 1 mL of the HATU coupling agent stock solution (3.95 equiv., 0.099 mmol HATU) and DIPEA (8 equiv., 0.035 mL, 0.2 mmol) to the falcon tube containing the Fmoc-protected amino acid and vortexing until all solids were dissolved (~2.5 minutes). The activated amino acid solution (1 mL) was then added to the reaction vessel containing the unprotected resin. The vessel containing the solution was transferred to the microwave reactor, and the temperature probe was placed in the reaction vessel. The microwave amino acid coupling program was initiated (ramp to 70 °C over 2 min, hold at 70 °C for 13 min). After program completion, the vessel as removed from the microwave reactor, the coupling solution was aspirated from the vessel, the resin was washed 5 x 4 mL ACS-grade DMF and the resin was allowed to dry via aspiration for ~2.5 min This procedure was repeated for each coupling step of solid-phase peptide synthesis.

Deprotection of Fmoc-protected Amino Acids

A solution for Fmoc-group deprotection was prepared by adding 200 mL of ACS-grade piperidine to 800 mL of ACS-grade DMF (20% v/v piperidine in DMF). The Fmoc group deprotection solution is stable, and excess solution was saved for future syntheses. Fmoc deprotection solution (~3 mL) was added to the reaction vessel containing the Fmoc-protected resin-bound peptide, the vessel was transferred to the microwave reactor, and the temperature probe was placed in the reaction vessel solution. The microwave Fmoc group deprotection program was initiated (ramp to 80°C over 2 min, hold at 80°C for 4 min). After program completion, the vessel was removed from the microwave reactor, the deprotection solution was aspirated from the vessel, the resin was washed with 5 x 4 mL ACS-grade DMF, and the resin was dried via aspiration for ~2.5 min. This procedure was repeated for each Fmoc group deprotection step of solid-phase peptide synthesis.

Deprotection of Alloc-protected Amino Acids

A solution for Alloc-group deprotection steps was prepared by adding $Pd(PPh_3)_4$ (0.5 equiv. relative to amine loading of resin, 14.4 mg, 125 μmol), $PhSiH_3$ (23.0 equiv. relative to amine loading of resin, 0.071 mL, 0.58 mmol) to DCE (1 mL). The Alloc deprotection solution was prepared immediately prior to use. Alloc deprotection solution (~1 mL) was added to the reaction vessel containing the dried Fmoc-protected resin-bound peptide, the vessel containing the solution was transferred to the microwave reactor, and the temperature probe was placed in the reaction vessel solution. The microwave Alloc deprotection program was initiated (ramp to 35°C over 2 min, hold at 35°C for 12 min). After program completion, the vessel was removed from the microwave reactor, the Alloc deprotection solution was aspirated from the vessel, the resin was washed with 5 x 4 mL ACS-grade DMF, and the resin was dried via aspiration for ~2.5 min. This procedure was repeated twice for each Alloc group deprotection step of solid-phase peptide synthesis.

Cleavage of peptides from resin

A cleavage solution for removing all acid-labile protecting groups and freeing peptides from the resin was prepared by dissolving 2.5% triispropylsilane and 2.5% H_2O in 95% TFA (v:v:v). The cleavage solution was prepared immediately prior to use. Cleavage solution (~4 mL) was added to the reaction vessel containing the completed resin-bound peptide. The vessel was transferred to the microwave reactor, and the temperature probe was placed in the reaction vessel. The microwave cleavage program was initiated (ramp to 40°C over 2 min, hold at 40°C for 30 min). After program completion, the vessel was removed from the microwave reactor, and the solution was stirred at room temp for 10 min. The crude peptide cleavage solution was then expunged into a 50 mL centrifuge tube, and the resin was washed 3x with 100% TFA, which was drained into the same tube. The TFA solution in the centrifuge tube was concentrated under a stream of N_2 until a thick gel remained. To the tube was added ~40 mL of cold diethyl ether. The resulting precipitate was isolated by centrifugation of the mixture at 4500 RPM for 10 minutes to produce a pellet of the crude peptide. The supernatant was decanted, and the tube containing the crude peptide solid was left partially capped on the benchtop under ambient conditions for 12 h to dry the pellet.

HPLC Purification and Isolation of Peptides

Samples of crude peptide for HPLC purification were prepared by dissolving the dried solid in ~2 mL of 1:1 H_2O:MeCN and filtering the resulting solution through a 0.45 μm filter into 1 mL high recovery disposable HPLC vials. Crude peptides were purified by HPLC using a preparative column (Waters XSelect CSH C4 OBD 130 Å, 5 μm, 19 mm X 250 mm) and a flow rate of 20 mL per min. A gradient of 70% A (H_2O + 0.1% TFA), 30% B (MeCN + 0.1% TFA) ramped to 45% A, 55% B over 10 min was used. The final 45% A, 55% B composition was held for 3 min before equilibrating over 2 min back to the starting conditions. The same method was used to purify all peptides. UV (220 nm channel) and MS (SIM mode) analysis were used to monitor for product elution. An autosampler was used to collect all fractions containing the pure peptide.

HPLC fractions containing the pure peptide were combined into a 50 mL centrifuge tube and concentrated under a stream of N_2 to remove all MeCN. The concentrated solution was then frozen using dry ice and lyophilized, yielding a fluffy white powder of pure peptide. The pure peptide was dissolved in 80:20 HPLC-grade MeCN:nanopure H_2O and transferred into a small, pre-weighed vial. The peptide

solution in the vial was concentrated to dryness under a stream of N_2, and the vial was placed under a high vacuum for 48 h to remove any residual moisture. The vial containing the dried, purified peptide was weighed to determine the final isolated mass of the peptide.

Additional notes on preparation of catalyst stocks: DCM could be used to dissolve the purified peptides and transfer to the final vial. However, HPLC-grade DCM was observed to leave a residue after concentration that was insoluble in MeCN. Catalyst stock solutions prepared from peptides transferred using DCM were found to behave the same as stock solutions prepared from peptides transferred using 80:20 HPLC-grade MeCN:nanopure water, despite the presence of insoluble particulate matter in the former. All kinetics and 1 h reactions for yield determination used catalyst stocks prepared from peptides transferred using 80:20 HPLC-grade MeCN:nanopure water. Peptides isolated by HPLC were TFA salts. All peptide mass and stock solution preparation calculations accounted for this. Some peptide TFA salts were only slightly soluble in MeCN. Additional TFA (1 to 3 equivalents relative to the peptide) was added to the stock solutions for these peptides to promote solubility. All reactions conducted using stock solutions with TFA added to promote peptide solubility accounted for the extra TFA (total amount of TFA in all reaction was always 1.2 equivalents relative to hydrocinnamaldehyde unless otherwise stated).

UPLC and HR-ESI-MS Analysis of Peptide Purity

To analyze peptide purity, 2.0 µL of a 0.5 mM solution of the peptide (0.000125 mmol peptide in 0.25 mL 80:20 MeCN:nanopure H_2O) was injected onto an UPLC equipped with an analytical Waters UPLC Protein BEH C4 column (300 Å, 1.7 µm, 2.1 mm X 100 mm) and UV-Vis detector. A gradient of 90% A (nanopure H_2O + 0.1% TFA), 10% B (MeCN + 0.1% TFA) ramped to 10% A, 90% B over 7 min was used. The final 10% A, 90% B composition was held for 1 min before equilibrating over 2 min back to the starting conditions. UV (220 nm channel) analysis was used to monitor for elution of the peptide. Peptide purity was determined using the Waters “ApexTrack” integration algorithm on UPLC chromatograms obtained using the 220 nm channel. Peptide mass was confirmed by HR-ESI-MS mass spectrometry.

Note on peptide stability: The TFA salts of hydrazide-containing peptides **2**-**12** and **15**-**18** slowly degraded when stored as stocks in MeCN under ambient conditions. Degradation could be slowed by storing peptides under N_2 at -20 °C. Degraded peptide stocks could be recovered via repurification by HPLC. No measurable degradation was observed of TFA salts of diamine peptides **19**-**21** in MeCN over a period of several months.

Expected masses, observed masses and purities for all peptides are provided in section 7. The expected masses were observed for all peptides. Purities of all peptides are summarized in Table S1.

Table S1. Sequences of α/β-peptide foldamers produced for this work and their purities

#	α/β-Peptide Foldamer Sequence	Purity (%)
**2**	TPP-ACPC-ACPC-**Glu(Hy)**-ACPC-ACPC-Ala-β^3HTyr-C(O)NHMe	98
**3**	TPP-ACPC-ACPC-Ala-ACPC-ACPC-**Glu(Hy)**-β^3HTyr-C(O)NHMe	98
**4**	TPP-ACPC-ACPC-Ala-ACPC-ACPC-**Asp(Hy)**-β^3HTyr-C(O)NHMe	97
**5**	TPP-ACPC-ACPC-**Glu(Hy)**-ACPC-ACPC-**Glu(Hy)**-β^3HTyr-C(O)NHMe	98
**6**	TPP-ACPC-ACPC-**Asp(Hy)**-ACPC-ACPC-**Asp(Hy)**-β^3HTyr-C(O)NHMe	96
**7**	TPP-ACPC-ACPC-**Glu(Hy)**-ACPC-ACPC-**Asp(Hy)**-β^3HTyr-C(O)NHMe	89
**8**	TPP-ACPC-ACPC-**Asp(Hy)**-ACPC-ACPC-**Glu(Hy)**-β^3HTyr-C(O)NHMe	87
**9**	TPP-ACPC-ACPC-**Glu(Hy)**-ACPC-ACPC-**Dap(SuccHy)**--β^3HTyr-C(O)NHMe	97
**10**	TPP-ACPC-ACPC-**Dap(SuccHy)**-ACPC-ACPC-**Dap(SuccHy)**-β^3HTyr-C(O)NHMe	95
**11**	TPP-**Glu(Hy)**-ACPC-ACPC-**Glu(Hy)**- β^3HTyr-C(O)NHMe	92
**12**	TPP-ACPC-ACPC-**Glu(Hy)**-ACPC-ACPC-**Glu(Hy)**-ACPC-ACPC-Tyr-C(O)NHMe	93
**15**	TPP-ACPC-**Glu(Hy)**-ACPC-ACPC-**Glu(Hy)**-ACPC-β^3HTyr-C(O)NHMe	91
**16**	TPP-ACPC-ACPC-**Glu(Hy)**-ACPC-ACPC-**Aze**-β^3HTyr-C(O)NHMe	97
**17**	TPP-ACPC-ACPC-**Glu(Hy)**-ACPC-ACPC-**Dab**-β^3HTyr-C(O)NHMe	98
**18**	TPP-ACPC-ACPC-**Glu(Hy)**-ACPC-ACPC-**Lys**-β^3HTyr-C(O)NHMe	93
**19**	TPP-ACPC-ACPC-**Aze**-ACPC-ACPC-**Aze**-β^3HTyr-C(O)NHMe	97
**20**	TPP-ACPC-ACPC-**Dab**-ACPC-ACPC-**Dap**-β^3HTyr-C(O)NHMe	100
**21**	TPP-ACPC-ACPC-**Lys**-ACPC-ACPC-**Lys**-β^3HTyr-C(O)NHMe	97
**22***	TPP-**Glu(Hy)**-ACPC-ACPC-**Glu(Hy)**-ACPC-ACPC-Tyr-C(O)NHMe	N/A
**23***	TPP-ACPC-ACPC-**Asp(Hy)**-ACPC-ACPC-Ala-β^3HTyr-C(O)NHMe	N/A
**24***	TPP-βAla-βAla-**Glu(Hy)**-βAla-βAla-**Glu(Hy)**- β^3HTyr-C(O)NHMe	N/A
**25***	TPP-β^3HAla-β^3HAla-**Glu(Hy)**-β^3HAla-β^3HAla-**Glu(Hy)**- β^3HTyr-C(O)NHMe	N/A
**26***	TPP-ACPC-ACPC-**An**-ACPC-ACPC-**An**-β^3HTyr-C(O)NHMe	N/A
**27***	TPP-AIB-APC-ACPC-AIB-APC-ACPC-Tyr-C(O)NHMe	N/A

(*) = gels or insoluble in pure MeCN; could not purify or conduct kinetics experiments with these peptides.

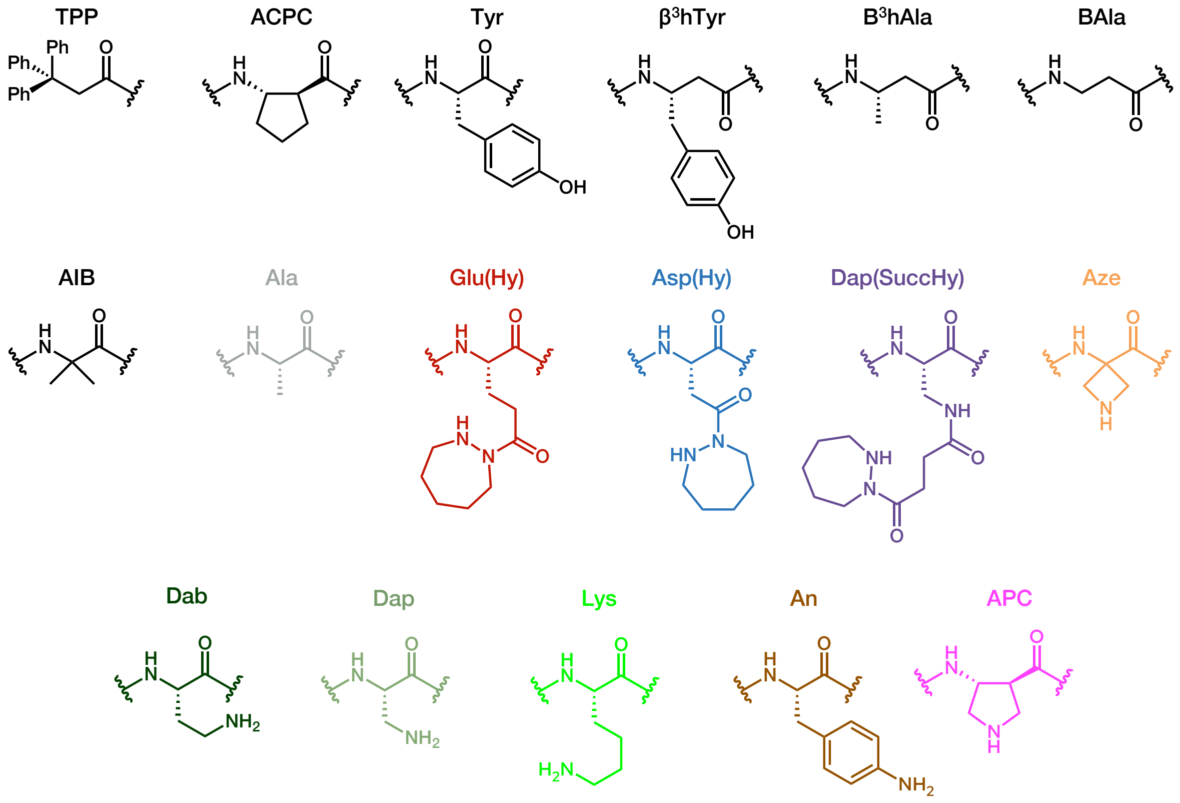

Figure S1. Structures of all α- and β-amino acids in α/β-peptides **2-12** and **15-27**

3. Experimental Information

Reactant stock solutions were prepared immediately prior to use using freshly opened (< 2 weeks old) bottles of Sigma-Aldrich brand MeCN-D_3 containing 1% TMS and were disposed of after 1 week of use. Hydrocinnamaldehyde was distilled prior to use. Freshly prepared catalyst stock solutions were allowed to sit for 24 h prior to use. α/β-Peptide catalyst stock solutions were stable when stored in a freezer for a several months, but some degradation was observed after ~1 year of storage. Reactions conducted in NMR tubes were not stirred. NMR probe temperature was calibrated prior to use. All kinetics and reaction profile experiments were conducted at least twice. Mesitylene was used as an internal standard in all reactions. Reported yields for the homoaldol product take into account that the maximum theoretical yield corresponds to half of the starting amount of hydrocinnamaldehyde.

3.1 Homoaldol Condensation Reaction Monitoring

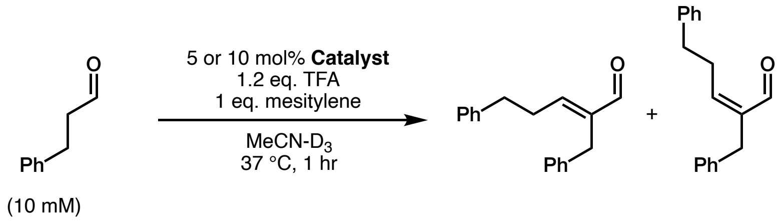

Homoaldol Condensation reaction monitoring and kinetics experiments were carried out using a modified literature procedure.[1] To a 5 x 178 mm NMR sample tube were added a 1:1 mix of hydrocinnamaldehyde and mesitylene (20 µL of a 0.25 M solution in MeCN-D_3, 0.005 mmol of hydrocinnamaldehyde, and 0.005 mmol mesitylene), and TFA (9.35 µL of a 0.588 M solution in MeCN-D_3, 0.0055 mmol, 1.1 equiv. for catalysts **1-4** or 8.5 µL of a 0.588 M solution in MeCN-D_3, 0.005 mmol, 1.0 equiv. for catalysts **5-21**). The reaction mixture was diluted with MeCN-D_3 (~434 µL, to a final aldehyde concentration of 0.01 M), and the NMR tube was capped. The sample was then inserted into a 500 MHz NMR spectrometer pre-heated to 37 °C, and the spectrometer sample was locked, tuned, and shimmed, and gain was adjusted. A ^{1}H NMR spectrum of the sample prior to addition of catalyst was acquired. The sample was then removed from the spectrometer and catalyst (20 µL of a 0.020 M stock in MeCN-D_3, 0.0005 mmol, 0.1 equiv. for the TFA salts of catalysts **1**-**4** or for or 10 µL of a 0.025 M stock in MeCN-D_3, 0.00025 mmol, 0.05 equiv. for the TFA salts of catalysts **5**-**21**) was quickly added. The NMR tube was briefly shaken to mix the sample and then immediately reinserted into the NMR spectrometer. Subsequent procedural steps and key NMR parameters varied in accordance with catalyst identity.

For initial rates experiments and 1 h reactions conducted with catalysts **1**-**4** and **16**-**21**, The spectrometer was again locked and shimmed. Exactly 5 min after addition of the catalyst a second ^{1}H NMR spectrum of the sample was acquired, followed by acquisition of an additional ^{1}H NMR spectrum every 3 min for 1 h. Key NMR spectrometer parameters for both initial rates and 1 h experiments are summarized in Table S2.

Table S2. Key NMR Parameters – Homoaldol Condensation – Slow Kinetics

Parameter	Description	Value
aq	Acquisition time (s)	1
ns	Number of scans	4
d1	Delay between scans (s)	25
d20	Delay between acquisitions (s)	180

For initial rates experiments conducted with catalysts **5-15** the spectrometer was not locked or shimmed upon reinsertion of the sample. Exactly 43 seconds after addition of the catalyst a second ^{1}H NMR spectrum of the sample was acquired, using the NMR parameters given in Table S3, followed by acquisition of an additional ^{1}H NMR spectrum every ~10 seconds for 2.5 minutes.

Table S3. Key NMR Parameters – Homoaldol Condensation – Very Fast Kinetics.

Parameter	Description	Value
aq	Acquisition time (s)	1
ns	Number of scans	1
d1	Delay between scans (s)	1
d20	Delay between acquisitions (s)	10

For 1 h reactions conducted with catalysts **5-15** the spectrometer was not locked or shimmed upon reinsertion of the sample. Exactly 43 seconds after addition of the catalyst a second ^{1}H NMR spectrum of the sample was acquired, using the key NMR parameters summarized in Table S4, followed by acquisition of an additional ^{1}H NMR spectrum every ~30 seconds for 1 h.

Table S4. Key NMR Parameters – Homoaldol Condensation –Fast Kinetics

Parameter	Description	Value
aq	Acquisition time (s)	1
ns	Number of scans	4
d1	Delay between scans (s)	1
d20	Delay between acquisitions (s)	30

Initial rates and 1 h reaction profiles for homoaldol reactions catalyzed by **1-21** are provided in section 4.2.

3.2 Macrocyclization Reaction Monitoring

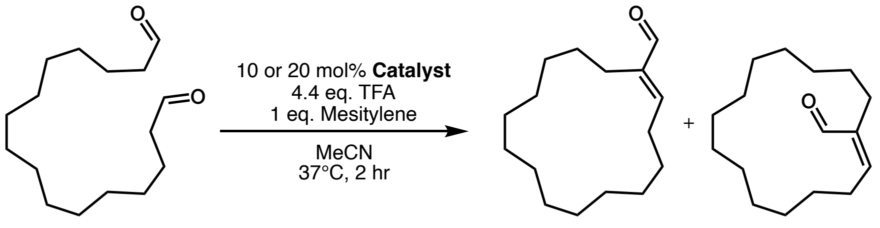

Macrocyclization reaction monitoring and kinetics experiments were carried out using a literature procedure and 20 mol% of catalyst **1** or 10 mol% of catalyst **5** or **13**.[1] Key NMR parameters for both initial rates and 2 h experiments are summarized in the table S5.

Table S5. Key NMR Parameters – Macrocyclization

Parameter	Description	Value
aq	Acquisition time (s)	1
ns	Number of scans	42
d1	Delay between scans (s)	1
d20	Delay between acquisitions (s)	180

Initial rates and 2 h reaction profiles for the **1-**, **5-** and **13**-catalyzed macrocyclization reactions are provided in section 4.4.

3.3 Other Experiments

3.3.1 Dependence of Initial Rate on TFA Loading

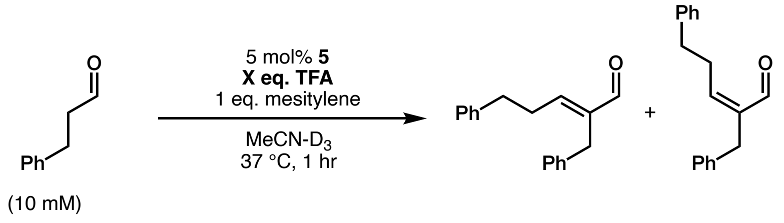

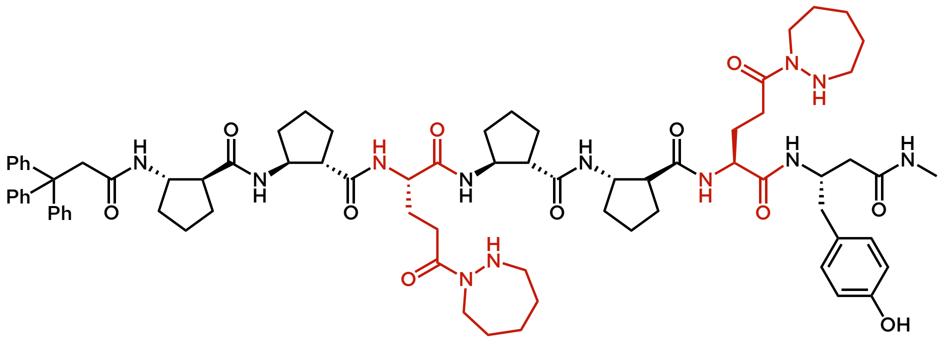

TPP-ACPC-ACPC-Glu(Hy)-ACPC-ACPC-Glu(Hy)-B[3]HTyr-C(O)NHMe (5)

Homoaldol condensations of hydrocinnamaldehyde with loadings of 0.2, 0.3, 0.6, 0.7, 1.2 and 1.5 equivalents TFA were carried out according to the procedure detailed in section 3.1 using catalyst **5**. Relative initial rates (v_{REL}) were determined by ^{1}H NMR analysis using mesitylene as an internal standard (Table S6). Plots depicting the initial rates under each reaction condition are provided below (Figures S2 to S7). The initial reaction rate of **5** under the 0.2 equivalents TFA conditions was used as the basis for calculation of v_{REL} for this comparison.

Table S6. Relative initial rates of homoaldol condensation catalyzed by 5 mol% **5** conducted with 0.2, 0.3, 0.6, 0.7, 1.2 and 1.5 equivalents of TFA.

X (equiv.)	v_{REL}
0.2	1
0.3	1.4
0.6	1.8
0.7	1.7
1.2	2.1
1.5	1.7

The observed dependence of reaction rate on TFA loading differed for foldamer **5** and simple dihydrazide **13**. Simple dihydrazide **13** displayed a maximum activity at ~0.6 equivalents of TFA.[1] At higher TFA loadings, **13** was slightly less active. All reactions reported herein were conducted with 1.2 equivalents of TFA unless otherwise stated.

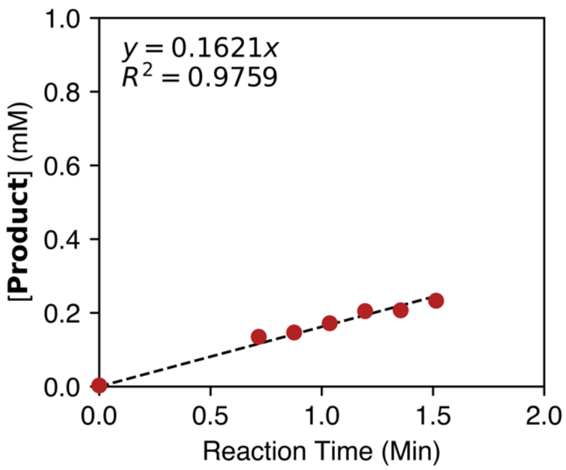

Figure S2. Initial rate for hydrocinnamaldehyde homoaldol reaction catalyzed by 5 mol% **5** with 0.2 equiv. TFA.

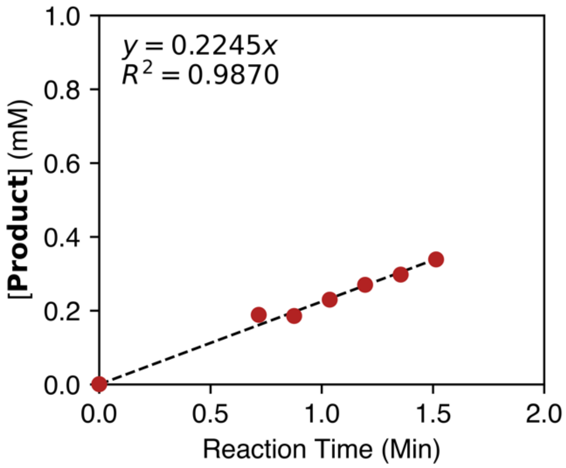

Figure S3. Initial rate for hydrocinnamaldehyde homoaldol reaction catalyzed by 5 mol% **5** with 0.3 equiv. TFA.

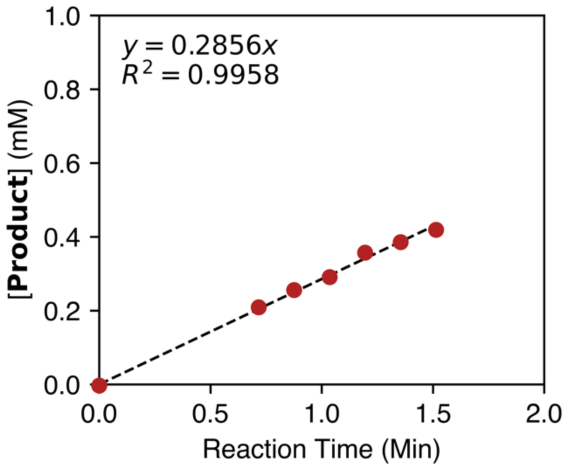

Figure S4. Initial rate for hydrocinnamaldehyde homoaldol reaction catalyzed by 5 mol% **5** with 0.6 equiv. TFA.

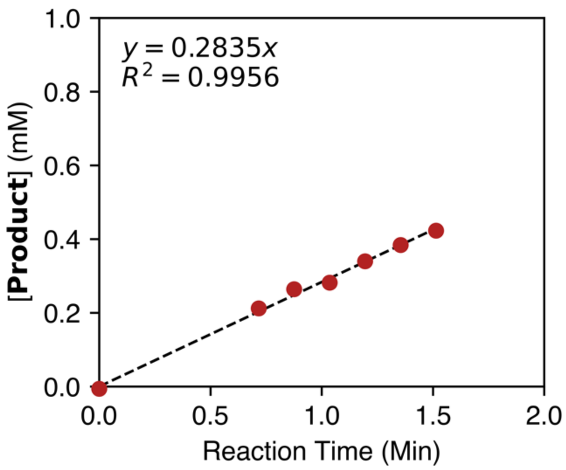

Figure S5. Initial rate for hydrocinnamaldehyde homoaldol reaction catalyzed by 5 mol% **5** with 0.7 equiv. TFA.

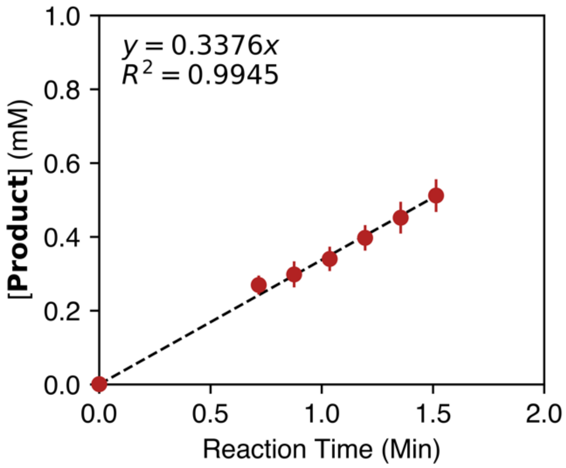

Figure S6. Initial rate for hydrocinnamaldehyde homoaldol reaction catalyzed by 5 mol% **5** with 1.2 equiv. TFA.

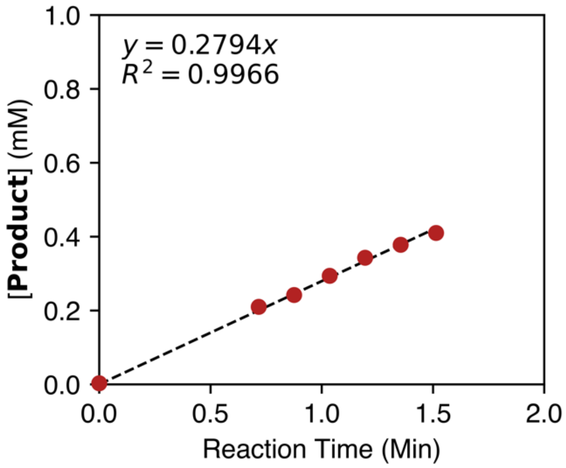

Figure S7. Initial rate for hydrocinnamaldehyde homoaldol reaction catalyzed by 5 mol% **5** with 1.5 equiv. TFA.

3.3.2 Dependence of Initial Rate on H_2O Loading

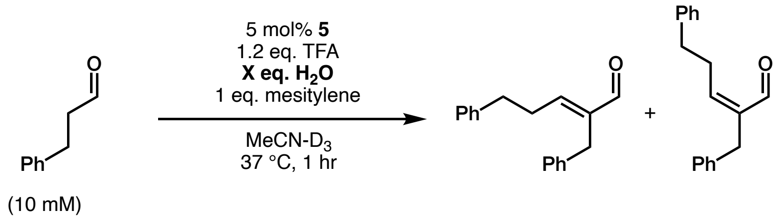

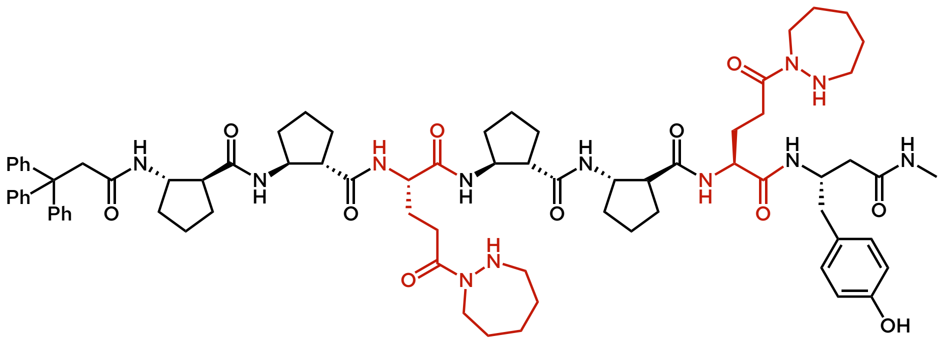

TPP-ACPC-ACPC-Glu(Hy)-ACPC-ACPC-Glu(Hy)-B³HTyr-C(O)NHMe (5)

Homoaldol condensations of hydrocinnamaldehyde in the presence of 1.0 and 5.0 equivalents H_2O were carried out according to the procedure detailed in section 3.1 using catalyst **5**. Relative initial rates (v_{REL}) were determined by ^{1}H NMR analysis using mesitylene as an internal standard (Table S7). Plots depicting the initial rates under each reaction condition are provided below (Figures S8 to S10). The initial reaction rate of **5** with no added H_2O was used as the basis for calculation of v_{REL} for this comparison. The initial rate of aldol condensation catalyzed by **5** was highly sensitive to the presence of water. Excess water significantly slowed the homoaldol reaction.

Table S7. Relative initial rates of homoaldol condensation catalyzed by 5 mol% **5** conducted in the presence of 0, 1 and 5 equivalents of H_2O.

X (equiv.)	v_{REL}
0.0	1.0
1.0	0.8
5.0	0.2

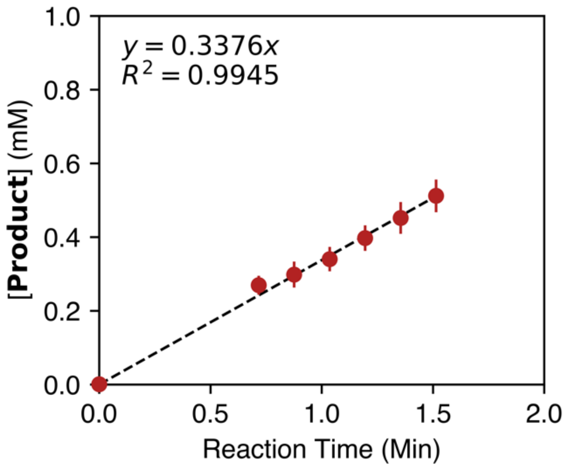

Figure S8. Initial rate for hydrocinnamaldehyde homoaldol reaction catalyzed by 5 mol% **5** and no added H_2O.

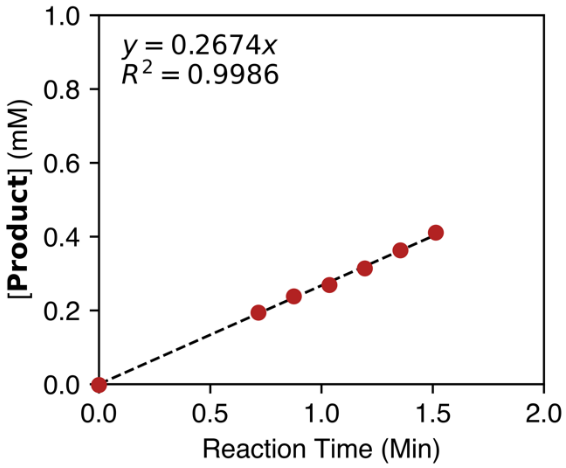

Figure S9. Initial rate for hydrocinnamaldehyde homoaldol reaction catalyzed by 5 mol% **5** and 1 equivalent of H_2O.

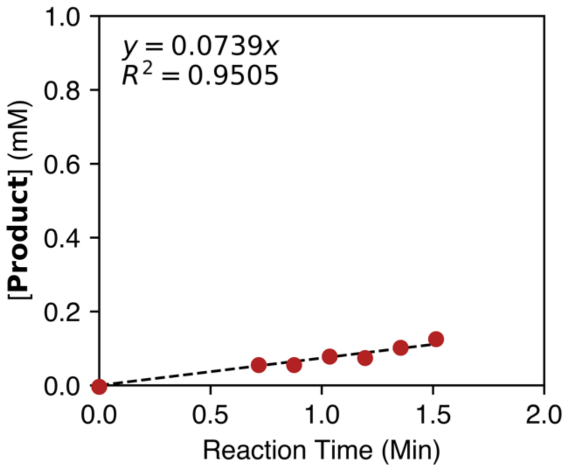

Figure S10. Initial rate for hydrocinnamaldehyde homoaldol reaction catalyzed by 5 mol% **5** and 5 equivalents of H_2O.

3.3.3 Dependence of Initial Rate on [**5**]

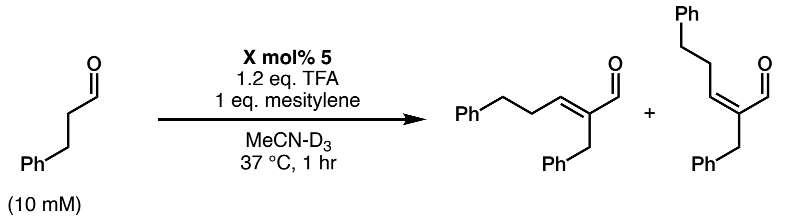

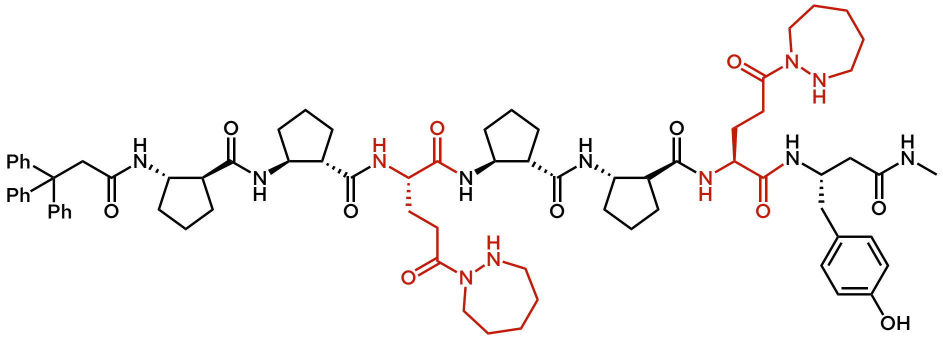

TPP-ACPC-ACPC-Glu(Hy)-ACPC-ACPC-Glu(Hy)-B^{3}HTyr-C(O)NHMe (5)

Homoaldol condensations of hydrocinnamaldehyde with loadings of 1.25, 2.50, 3.75, 5.0 and 6.25 mol% **5** were carried out according to the procedure detailed in section 3.1. Initial reaction rates (v_{INT}) were determined by ^{1}H NMR analysis using mesitylene as an internal standard. Plots depicting the initial rates under each reaction condition are provided below (Figures S12 to S16).

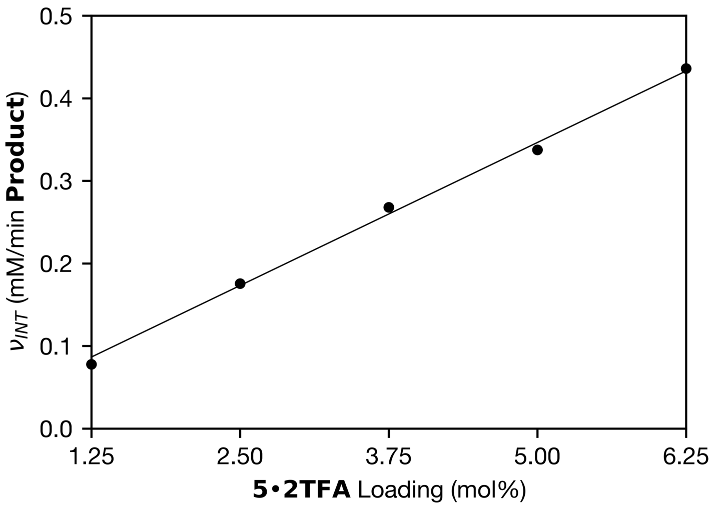

Figure S11. Initial rates of homoaldol reaction catalyzed with 1.25, 2.5, 3.75, 5.0 or 6.25 mol% **5**.

An approximately first-order dependence of v_{INT} on [**5**] was observed, suggesting that **5** serves as a bifunctional catalyst that simultaneously activates two equivalents of hydrocinnamaldehyde via enamine and iminium activation (Figure S11).

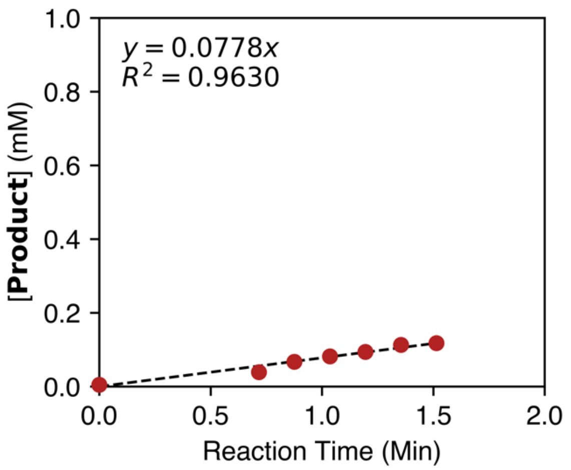

Figure S12. Initial rate for hydrocinnamaldehyde homoaldol reaction catalyzed by 1.25 mol% **5**.

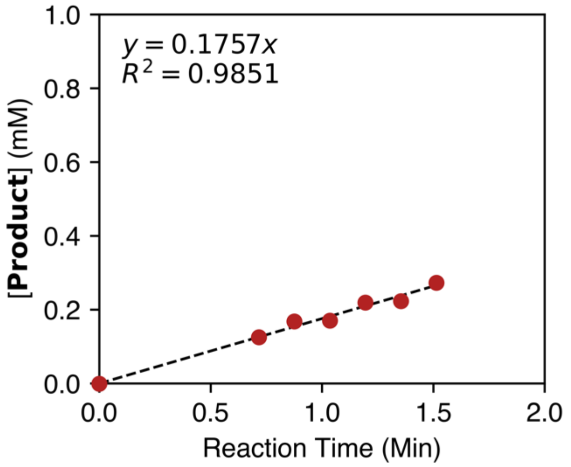

Figure S13. Initial rate for hydrocinnamaldehyde homoaldol reaction catalyzed by 2.50 mol% **5**.

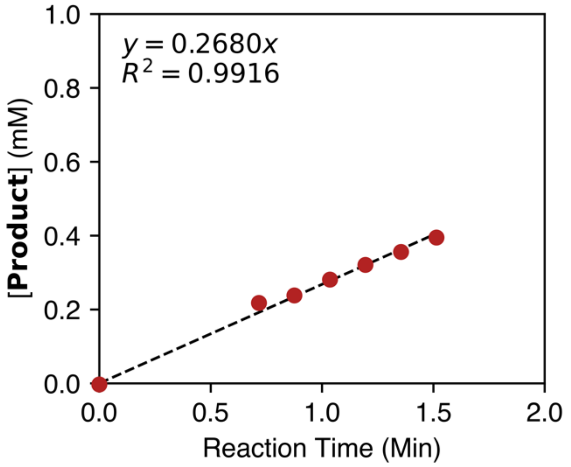

Figure S14. Initial rate for hydrocinnamaldehyde homoaldol reaction catalyzed by 3.75 mol% **5**.

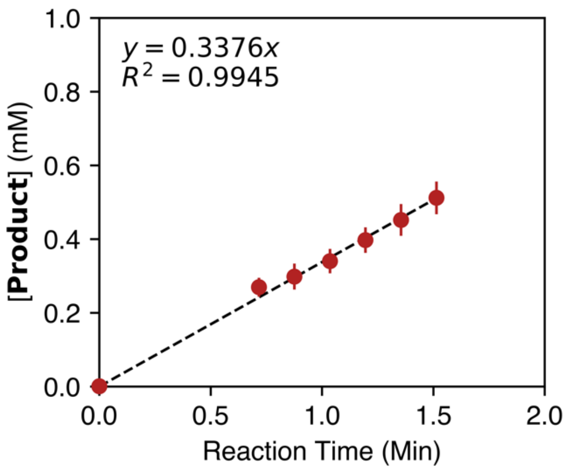

Figure S15. Initial rate for hydrocinnamaldehyde homoaldol reaction catalyzed by 5.00 mol% **5**.

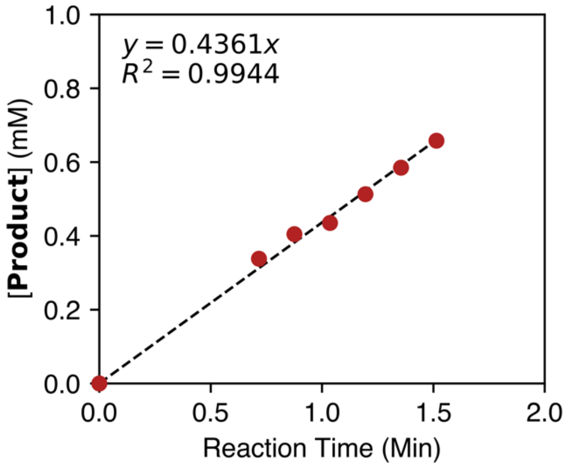

Figure S16. Initial rate for hydrocinnamaldehyde homoaldol reaction catalyzed by 6.25 mol% **5**.

3.3.4 Dependence of Initial Rate on Dihydrazide α/β-Peptides Sequence Order

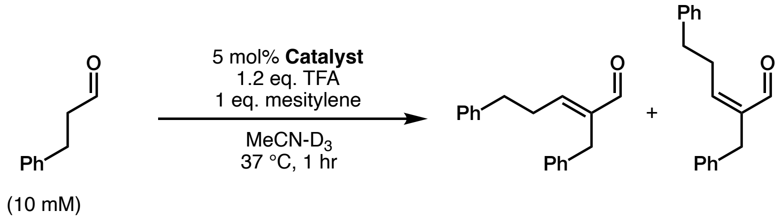

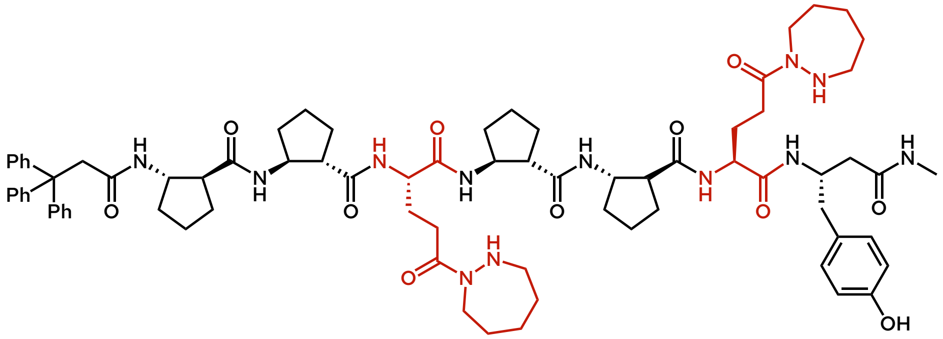

TPP-ACPC-ACPC-Glu(Hy)-ACPC-ACPC-Glu(Hy)-B³HTyr-C(O)NHMe (5)

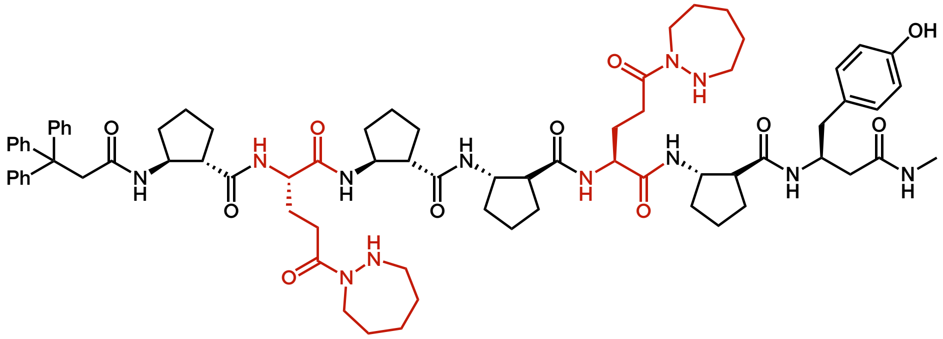

TPP-ACPC-Glu(Hy)-ACPC-ACPC-Glu(Hy)-ACPC-β³HTyr-C(O)NHMe (15)

Homoaldol condensations of hydrocinnamaldehyde with catalysts **5** and **15** were carried out according to the procedure detailed in section 3.1. Relative rates (v_{REL}) and 1 h reaction yields were determined by ^{1}H NMR analysis using mesitylene as an internal standard. The initial reaction rate of **1** was used as the basis for calculation of v_{REL} for this comparison. Plots depicting the reaction profiles and initial rates for each catalyst are provided in section 4.2. Relative rates and 1 h reaction yields for both catalysts are summarized in Table S8. Another foldamer (**22**) containing 2 Glu(Hy) residues in the *i*, *i*+3 arrangement was synthesized, but proved insoluble in MeCN, preventing testing (see section 3.3.6).

Table S8. Relative initial rates of homoaldol condensation catalyzed by small molecule monohydrazide 1 or dihydrazide foldamers **5** or **15** with varied peptide sequence.

Catalyst	v_{REL}	1 h yield (%)
**1***	1	5
**5**	89	95
**15**	93	98

(*) = 10 mol% catalyst used.

The amino acid sequence of **5** could be shifted by one residue (to the sequence of peptide **15**) while maintaining catalyst activity. Further shifting of the sequence yielded an insoluble peptide **22** that could not be tested.

3.3.5 Reactivities of α/β-Peptides Containing 1° and 2° Amines

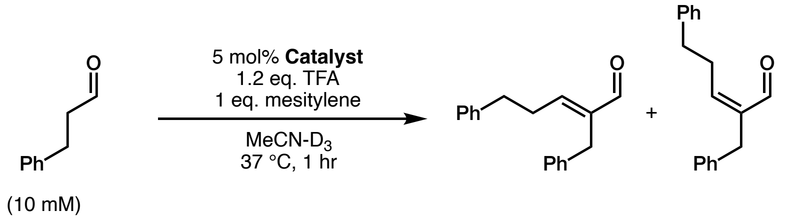

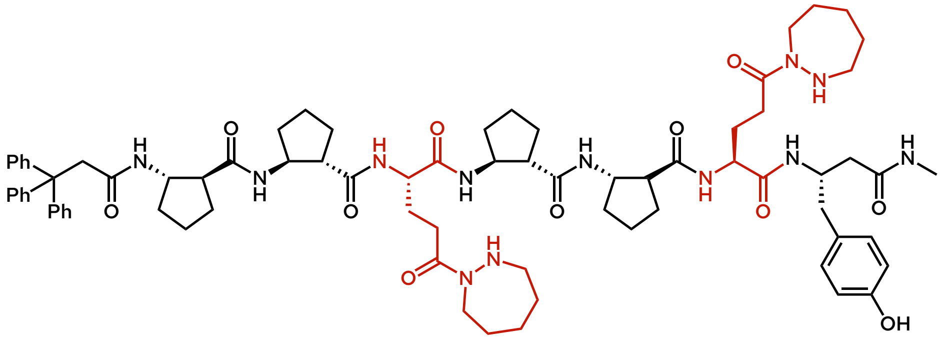

TPP-ACPC-ACPC-Glu(Hy)-ACPC-ACPC-Glu(Hy)-B^{3}HTyr-C(O)NHMe (5)

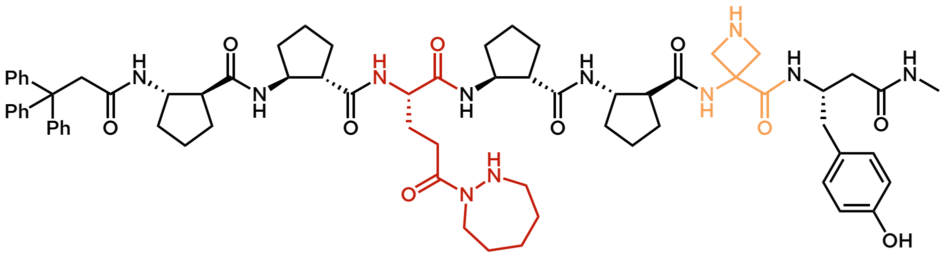

TPP-ACPC-ACPC-Glu(Hy)-ACPC-ACPC-Aze-B^{3}HTyr-C(O)NHMe (16)

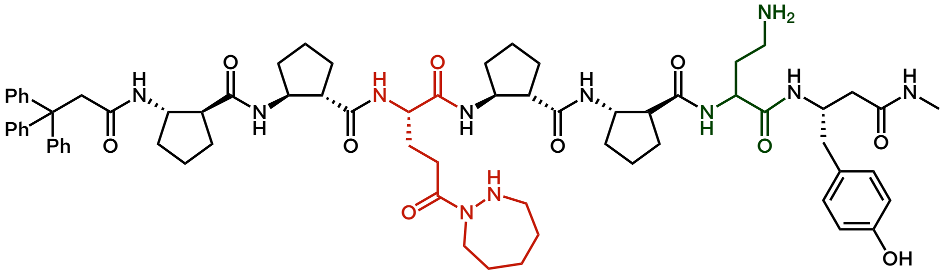

TPP-ACPC-ACPC-Glu(Hy)-ACPC-ACPC-Dab-β^{3}HTyr-C(O)NHMe (17)

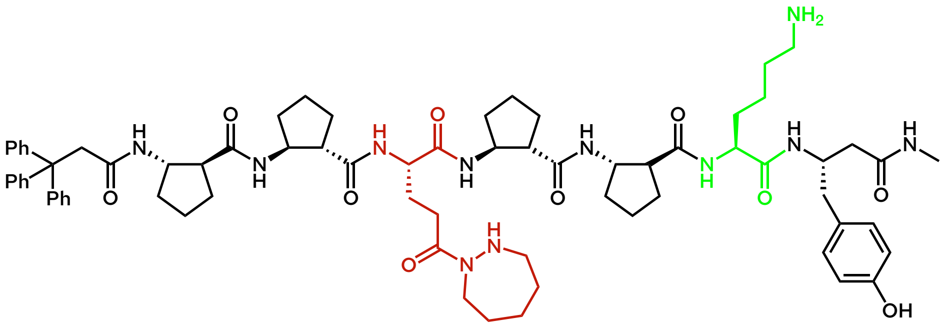

TPP-ACPC-ACPC-Glu(Hy)-ACPC-ACPC-Lys-β^3HTyr-C(O)NHMe (18)

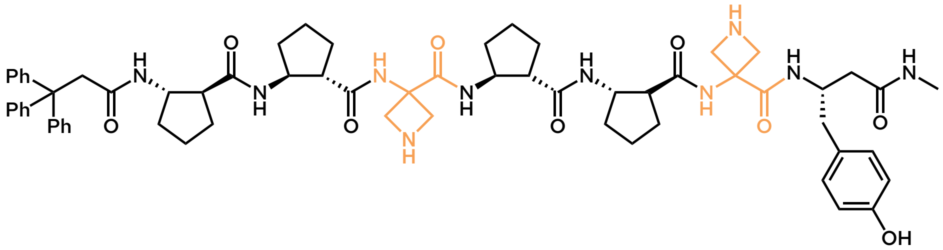

TPP-C(O)NH-ACPC-ACPC-Aze-ACPC-ACPC-Aze-B^{3}HTyr-C(O)NHMe (19)

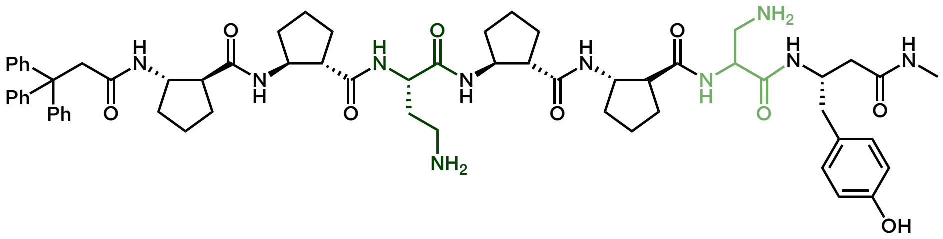

TPP- ACPC-ACPC-Dab-ACPC-ACPC-Dap-B^{3}HTyr-C(O)NHMe (20)

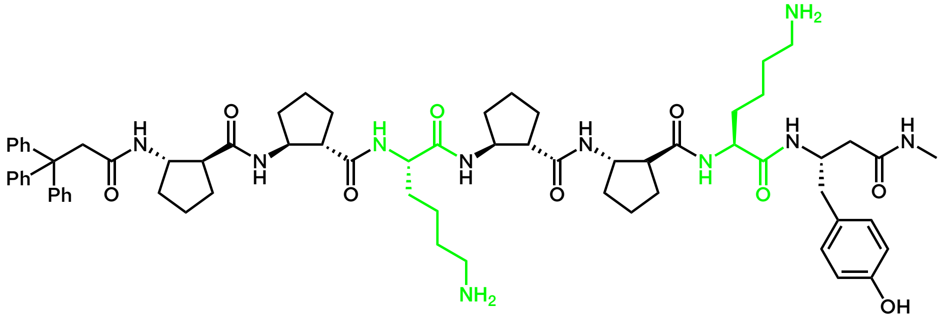

TPP-ACPC-ACPC-Lys-ACPC-ACPC-Lys-β^3HTyr-C(O)NHMe (21)

Homoaldol condensations of hydrocinnamaldehyde with catalysts **5** and **16**-**21** were carried out according to the procedure detailed in section 3.1. Relative initial rates (v_{REL}) and 1 h reaction yields were determined by ^{1}H NMR analysis using mesitylene as an internal standard. The initial reaction rate of **1** was used as the basis for calculation of v_{REL} for this comparison. Plots depicting the reaction profiles and initial rates for each catalyst are provided in section 4.2. Relative rates and 1 h reaction yields for all catalysts are summarized in Table S9.

Table S9. Relative initial rates of homoaldol condensation catalyzed by small molecule monohydrazide **1**, Glu(Hy)-Glu(Hy) dihydrazide foldamer **5** or **16**-**21** containing 1° and 2° amine reactive groups

Catalyst	v_{REL}	1 h yield (%)
**1***	1	5
**5**	89	95
**16**	5	20
**17**	2	4
**18**	2	5
**19**	0.3	2
**20**	0	0
**21**	0	0

(*) = 10 mol% catalyst used.

Hydrazide-amine peptides **16**-**18** exhibited only slightly increased activity (v_{REL} = 2-5) relative to small-molecule monohydrazide **1**. This suggests that two hydrazide units are necessary to achieve the high levels of rate enhancement exhibited by **5**. Amine-amine catalysts **19**-**21** are analogues of foldamer catalysts previously identified as competent bifunctional foldamer aminocatalysts;[2] these catalysts produced only trace product under the reaction conditions and were far less active than small-molecule monohydrazide **1**.

3.3.6 Insoluble and Gel-Forming α/β-Peptides

The following α/β-Peptides were insoluble in MeCN or formed gels in MeCN/H_2O, preventing testing or purification by HPLC.

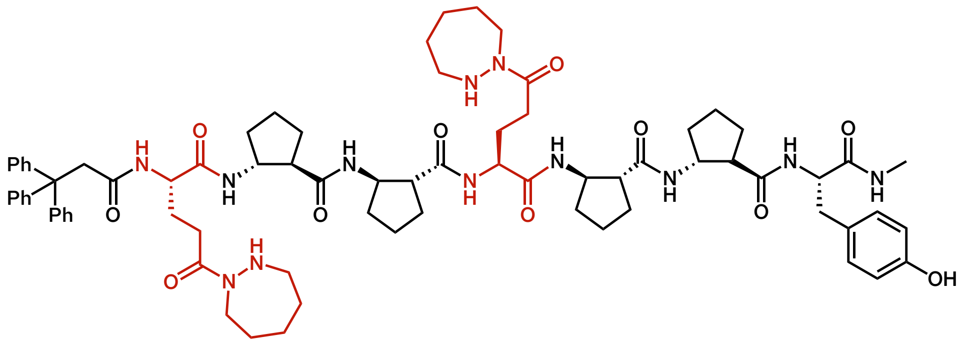

TPP-Glu(Hy)-ACPC-ACPC-Glu(Hy)- ACPC-ACPC-Tyr-C(O)NHMe (22)

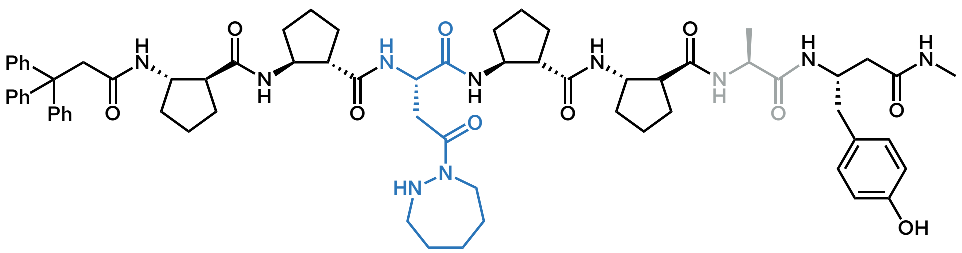

TPP-ACPC-ACPC-Asp(Hy)-ACPC-ACPC-Ala-β^3HTyr-C(O)NHMe (23)

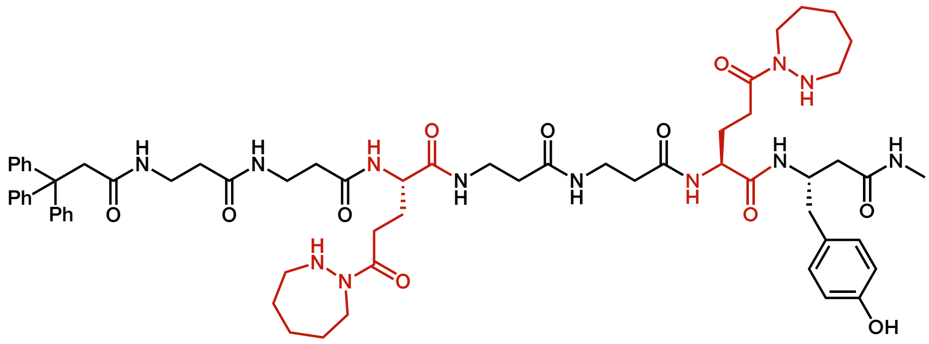

TPP-βAla-βAla-Glu(Hy)-βAla-βAla-Glu(Hy)- β^3HTyr-C(O)NHMe (24)

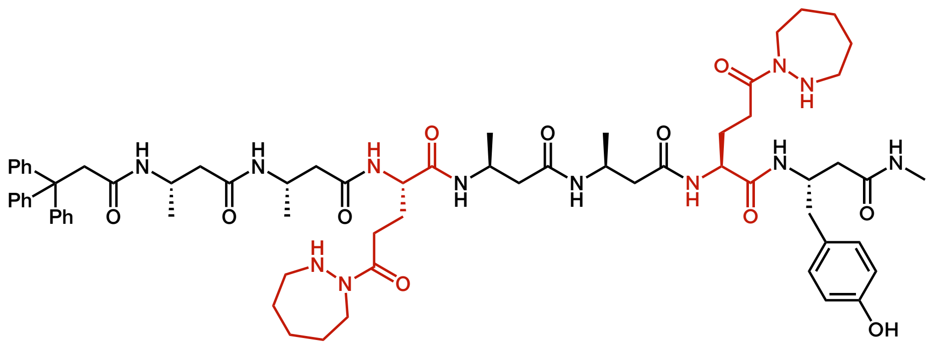

TPP-β^3HAla-β^3HAla-Glu(Hy)-β^3HAla-β^3HAla-Glu(Hy)- β^3HTyr-C(O)NHMe (25)

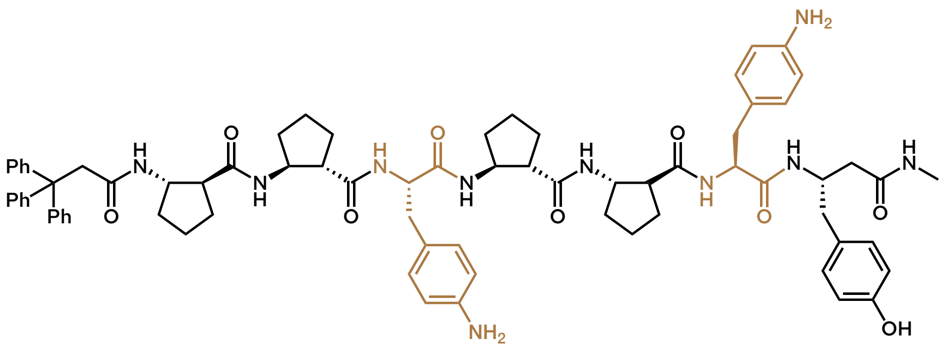

TPP-ACPC-ACPC-An-ACPC-ACPC-An-β^{3}HTyr-C(O)NHMe (26)

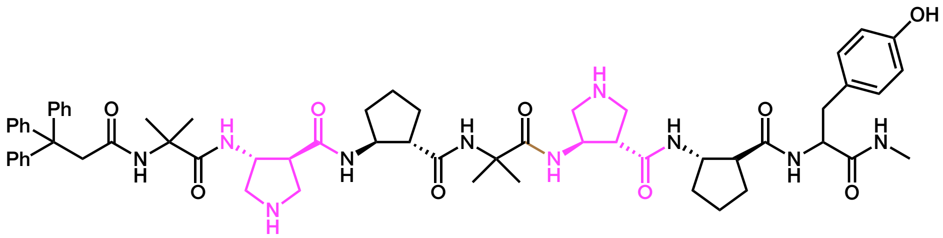

TPP-AIB-APC-ACPC-AIB-APC-ACPC-Tyr-C(O)NHMe (27)

4. Reaction Profiles and Initial Rates Data

Initial rates and 1 h reaction yields for α/β-peptides **22-27** could not be obtained as these foldamers formed gels under the purification conditions or were insoluble under the reaction conditions.

4.1 Relative Rates and 1 Hour Yields Summary – Homoaldol

Table S10. Summary of relative initial rates and 1 h reaction yields for catalysts of hydrocinnamaldehyde homoaldol condensation.

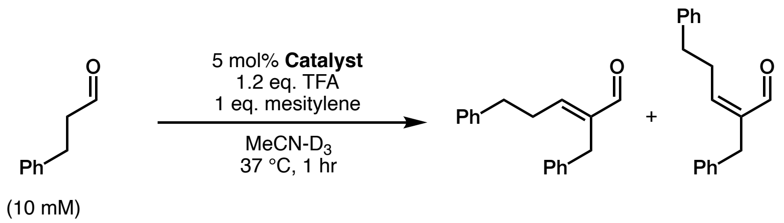

#	Catalyst	v_{rel}	Yield (%)
**1***	1-(1,2-Diazepan-1-yl)ethan-1-one 1	1	5
**2***	TPP-ACPC-ACPC-**Glu(Hy)**-ACPC-ACPC-Ala-β³HTyr-C(O)NHMe	0.5	2
**3***	TPP-ACPC-ACPC-Ala-ACPC-ACPC-**Glu(Hy)**-β³HTyr-C(O)NHMe	0.7	3
**4**	TPP-ACPC-ACPC-Ala-ACPC-ACPC- **Asp(Hy)**-β³HTyr-C(O)NHMe	0.8	3
**5**	TPP-ACPC-ACPC-**Glu(Hy)**-ACPC-ACPC-**Glu(Hy)**-β³HTyr-C(O)NHMe	89	95
**6**	TPP-ACPC-ACPC-**Asp(Hy)**-ACPC-ACPC-**Asp(Hy)**-β³HTyr-C(O)NHMe	21	59
**7**	TPP-ACPC-ACPC-**Glu(Hy)**-ACPC-ACPC-**Asp(Hy)**-β³HTyr-C(O)NHMe	24	73
**8**	TPP-ACPC-ACPC-**Asp(Hy)**-ACPC-ACPC-**Glu(Hy)**-β³HTyr-C(O)NHMe	43	83
**9**	TPP-ACPC-ACPC-**Glu(Hy)**-ACPC-ACPC-**Dap(SuccHy)**--β³HTyr-C(O)NHMe	48	88
**10**	TPP-ACPC-ACPC-**Dap(SuccHy)**-ACPC-ACPC-**Dap(SuccHy)**-β³HTyr-C(O)NHMe	39	85
**11**	TPP-**Glu(Hy)**-ACPC-ACPC-**Glu(Hy)**- β³HTyr-C(O)NHMe	39	88
**12**	TPP-ACPC-ACPC-**Glu(Hy)**-ACPC-ACPC-**Glu(Hy)**-ACPC-ACPC-Tyr-C(O)NHMe	77	92
**13**	1,12-Di(1,2-diazepan-1-yl)dodecane-1,12-dione	33	93
**14**	1,16-Di(1,2-diazepan-1-yl)hexadecane-1,16-dione	25	87
**15**	TPP-ACPC-**Glu(Hy)**-ACPC-ACPC-**Glu(Hy)**-ACPC-β³HTyr-C(O)NHMe	93	98
**16**	TPP-ACPC-ACPC-**Glu(Hy)**-ACPC-ACPC-**Aze**-β³HTyr-C(O)NHMe	5	20
**17**	TPP-ACPC-ACPC-**Glu(Hy)**-ACPC-ACPC-**Dab**-β³HTyr-C(O)NHMe	2	4
**18**	TPP-ACPC-ACPC-**Glu(Hy)**-ACPC-ACPC-**Lys**-β³HTyr-C(O)NHMe	2	5
**19**	TPP-ACPC-ACPC-**Aze**-ACPC-ACPC-**Aze**-β³HTyr-C(O)NHMe	0.3	2
**20**	TPP-ACPC-ACPC-**Dab**-ACPC-ACPC-**Dap**-β³HTyr-C(O)NHMe	0	0
**21**	TPP-ACPC-ACPC-**Lys**-ACPC-ACPC-**Lys**-β³HTyr-C(O)NHMe	0	0

(*) = 10 mol% catalyst used.

Comparisons between the v_{rel} and 1 h yields for sets of peptides not discussed in the main text are provided in sections 3.3.4 and 3.3.5

4.2 Reaction Profiles and Initial Rates for **1-21** – Homoaldol

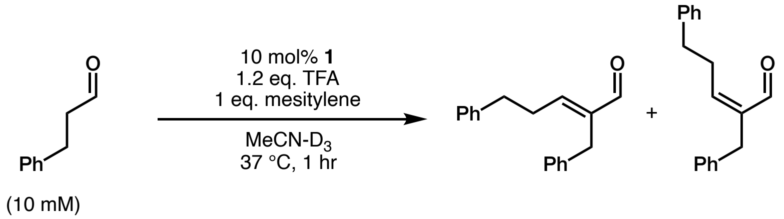

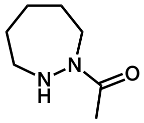

1-(1,2-Diazepan-1-yl)ethan-1-one (1)

v_{rel} = 1

1 h yield = 5%

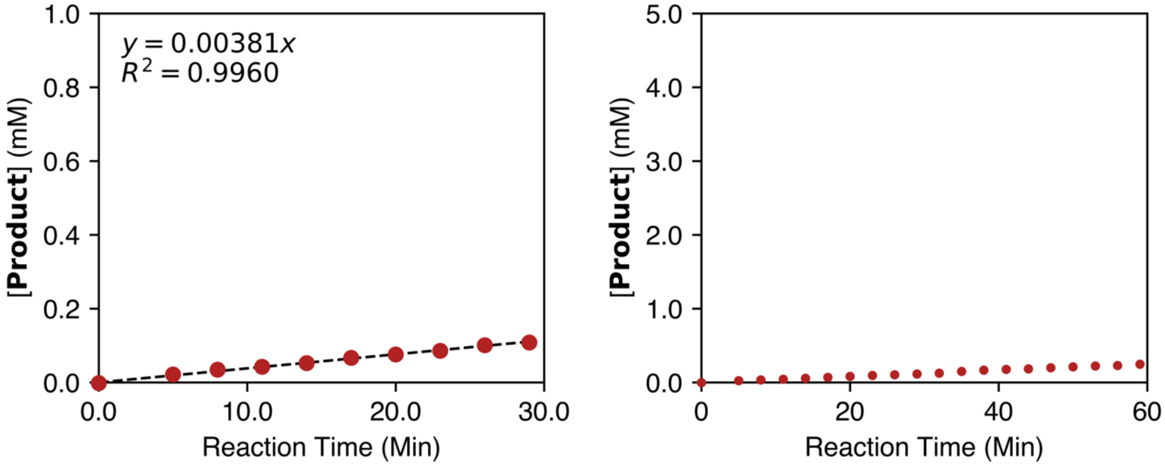

Figure S17. Initial rate and 1 h reaction profile for hydrocinnamaldehyde homoaldol reaction catalyzed by 10 mol% **1**.

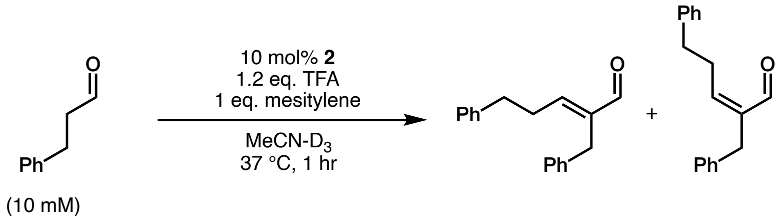

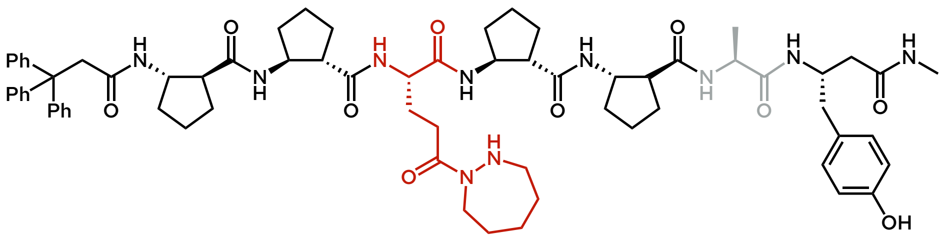

TPP-ACPC-ACPC-Glu(Hy)-ACPC-ACPC-Ala-B[3]HTyr-C(O)NHMe (2)

v_{rel} = 0.5

1 h yield = 2%

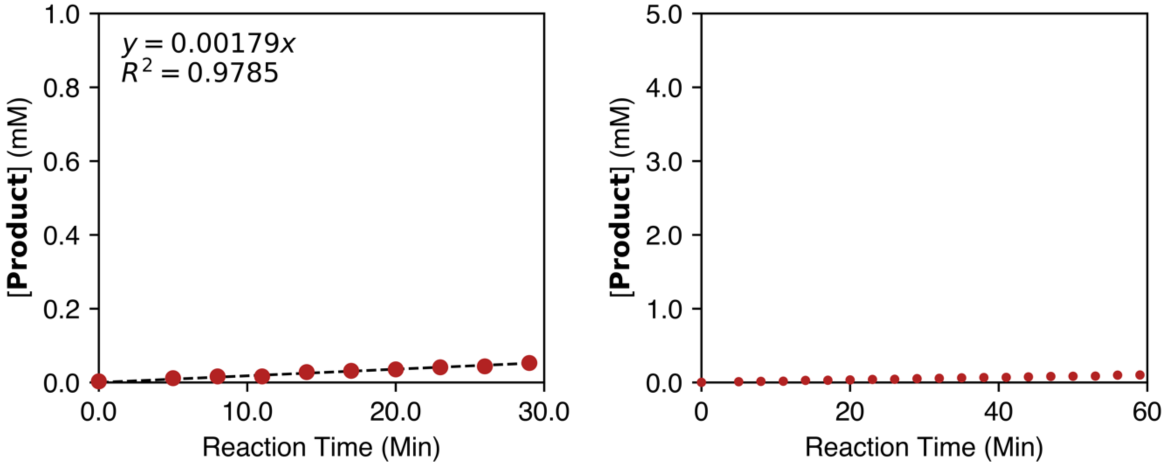

Figure S18. Initial rate and 1 h reaction profile for hydrocinnamaldehyde homoaldol reaction catalyzed by 10 mol% **2**.

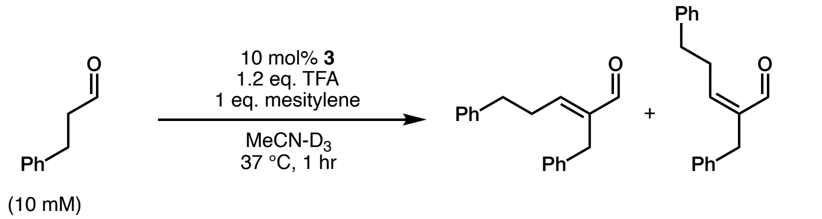

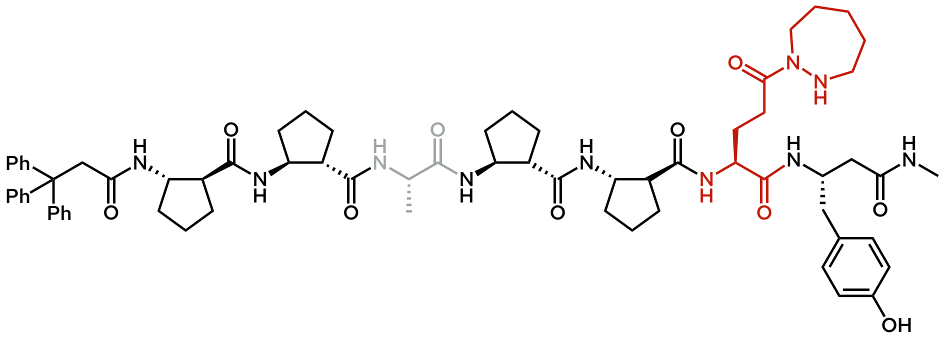

TPP-ACPC-ACPC-Ala-ACPC-ACPC-Glu(Hy)-B^{3}HTyr-C(O)NHMe (3)

v_{rel} = 0.7

1 h yield = 3%

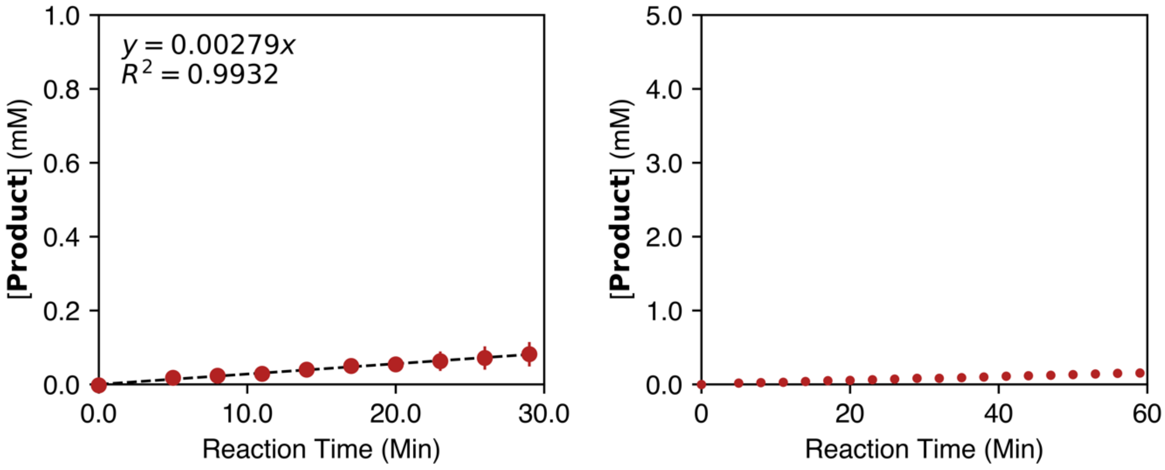

Figure S19. Initial rate and 1 h reaction profile for hydrocinnamaldehyde homoaldol reaction catalyzed by 10 mol% **3**.

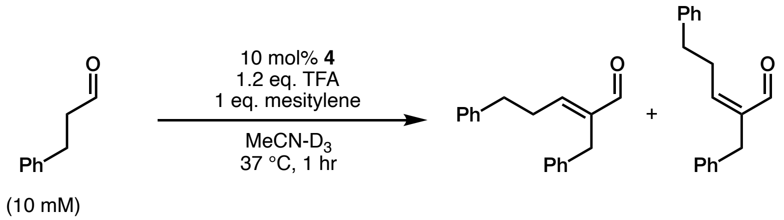

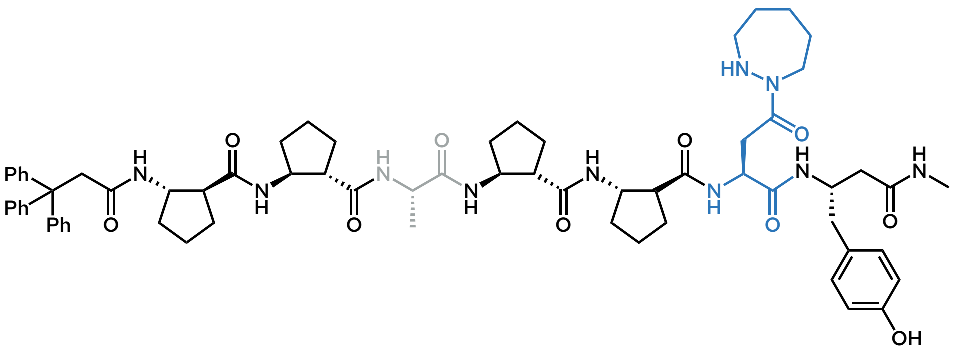

TPP-ACPC-ACPC-Ala-ACPC-ACPC-Asp(Hy)-β[3]HTyr-C(O)NHMe (4)

v_{rel} = 0.8

1 h yield = 3%

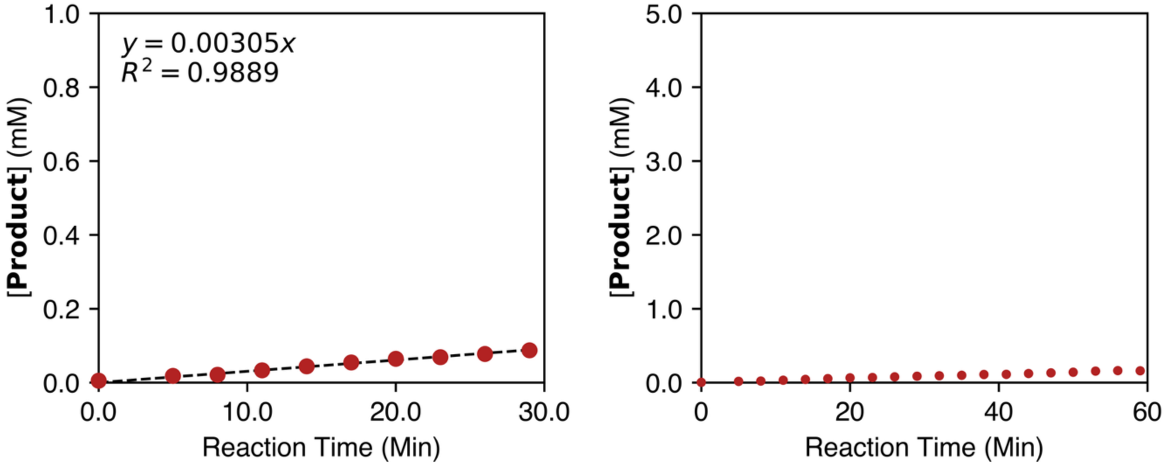

Figure S20. Initial rate and 1 h reaction profile for hydrocinnamaldehyde homoaldol reaction catalyzed by 10 mol% **4**.

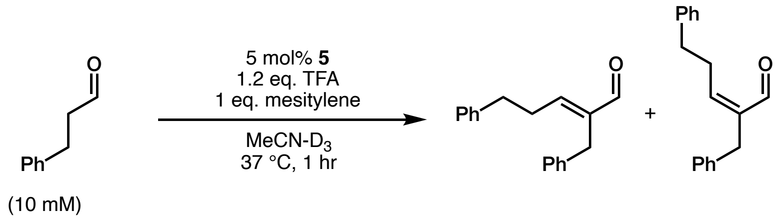

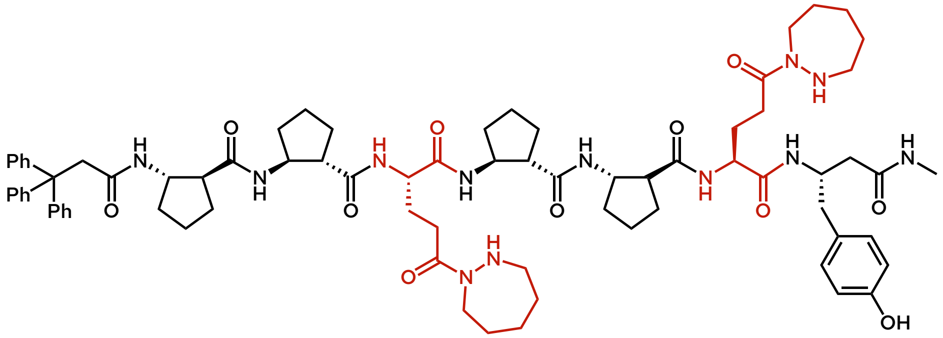

TPP-ACPC-ACPC-Glu(Hy)-ACPC-ACPC-Glu(Hy)-B³HTyr-C(O)NHMe (5)

$v_{rel} = 89$

1 h yield = 95%

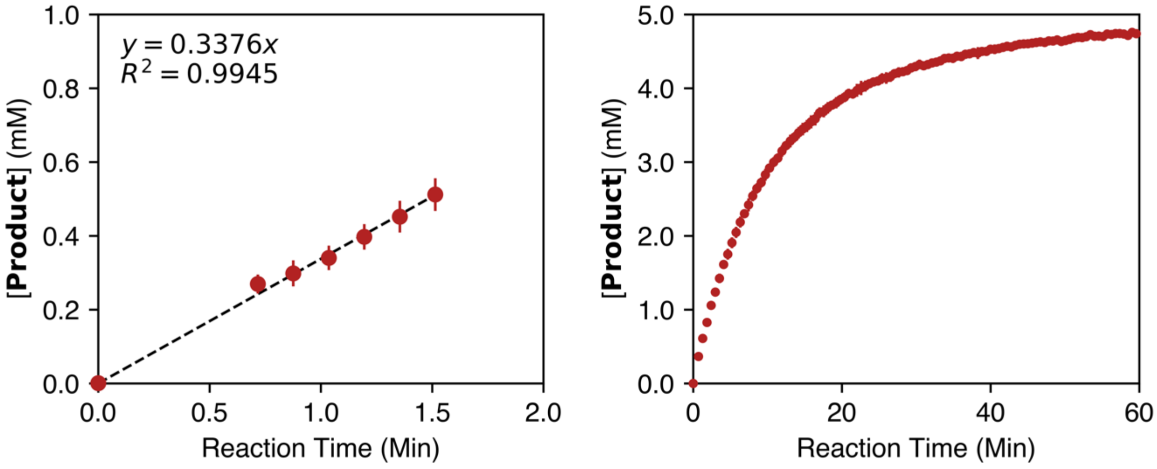

Figure S21. Initial rate and 1 h reaction profile for hydrocinnamaldehyde homoaldol reaction catalyzed by 5 mol% **5**.

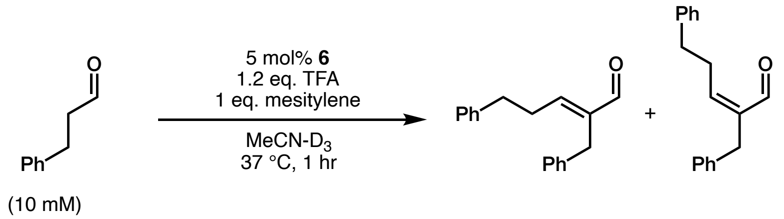

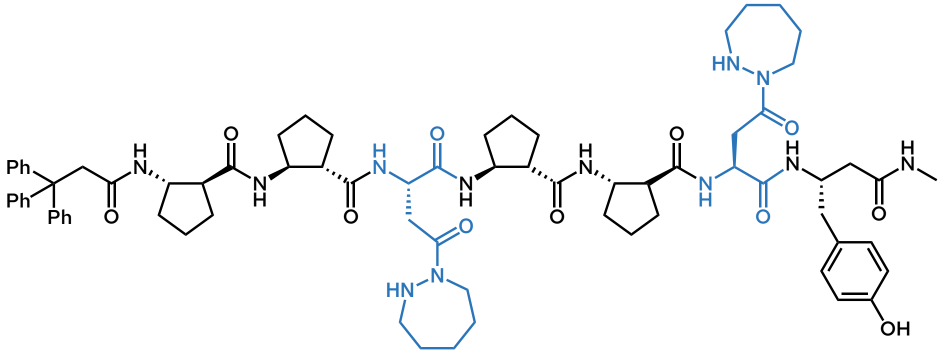

TPP- ACPC-ACPC-Asp(Hy)-ACPC-ACPC-Asp(Hy)-B^{3}HTyr-C(O)NHMe (6)

v_{rel} = 21

1 h yield = 59%

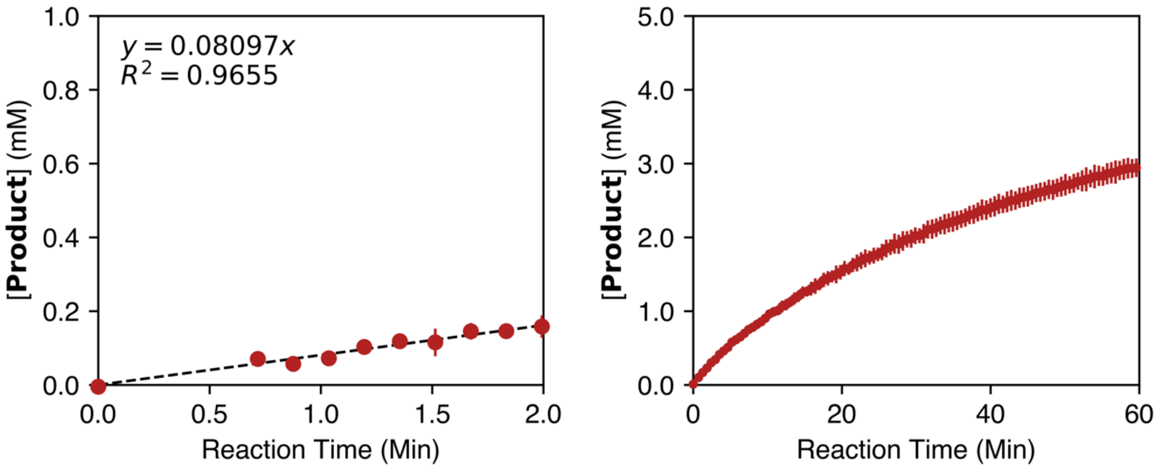

Figure S22. Initial rate and 1 h reaction profile for hydrocinnamaldehyde homoaldol reaction catalyzed by 5 mol% **6**.

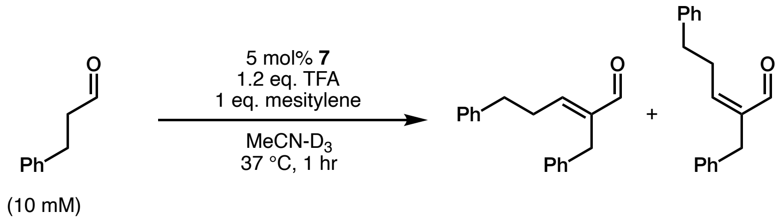

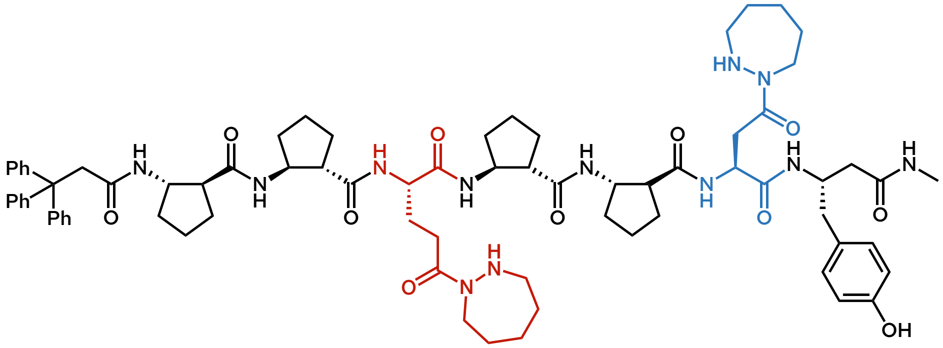

TPP-ACPC-ACPC-Glu(Hy)-ACPC-ACPC-Asp(Hy)-β^{3}HTyr-C(O)NHMe (7)

v_{rel} = 24

1 h yield = 73%

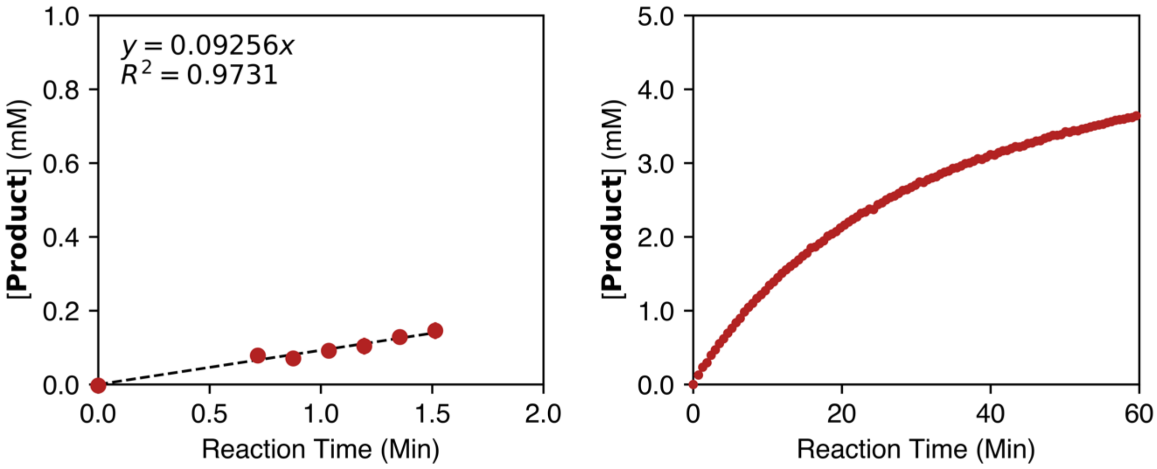

Figure S23. Initial rate and 1 h reaction profile for hydrocinnamaldehyde homoaldol reaction catalyzed by 5 mol% **7**.

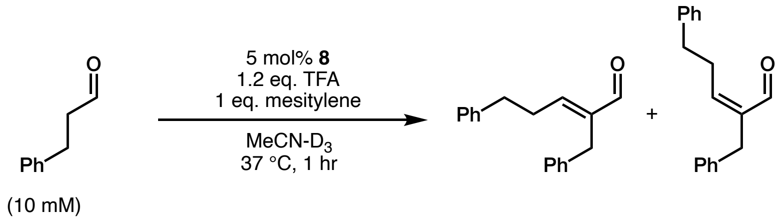

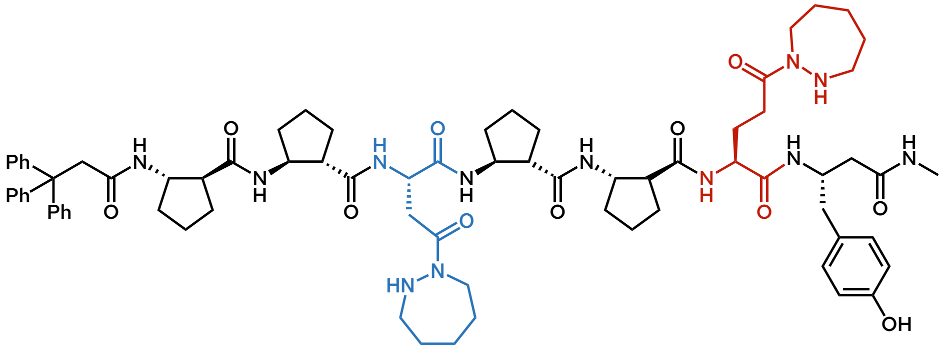

TPP-ACPC-ACPC-Asp(Hy)-ACPC-ACPC-Glu(Hy)-β³HTyr-C(O)NHMe (8)

$v_{\text{rel}} = 43$

1 h yield = 83%

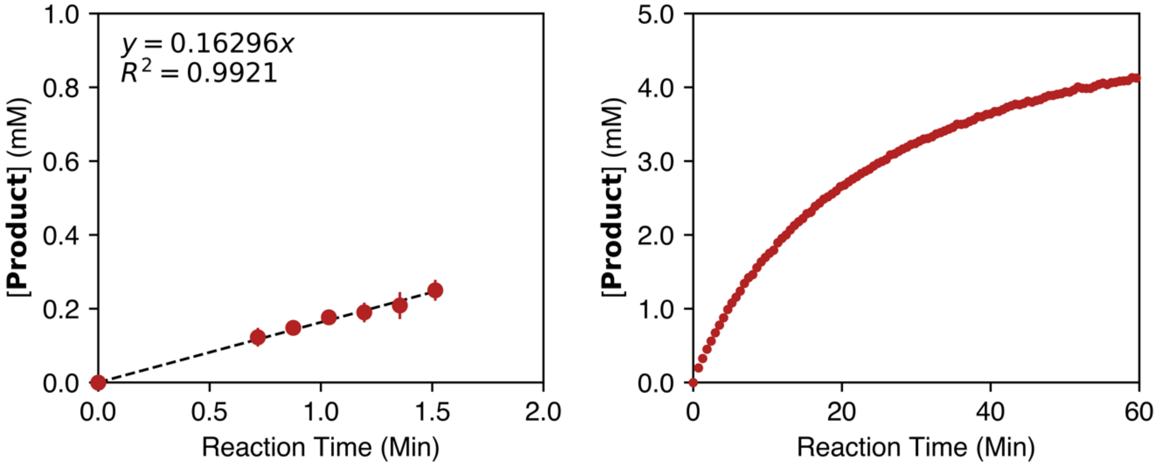

Figure S24. Initial rate and 1 h reaction profile for hydrocinnamaldehyde homoaldol reaction catalyzed by 5 mol% **8**.

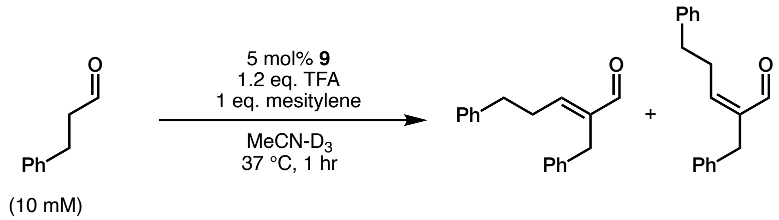

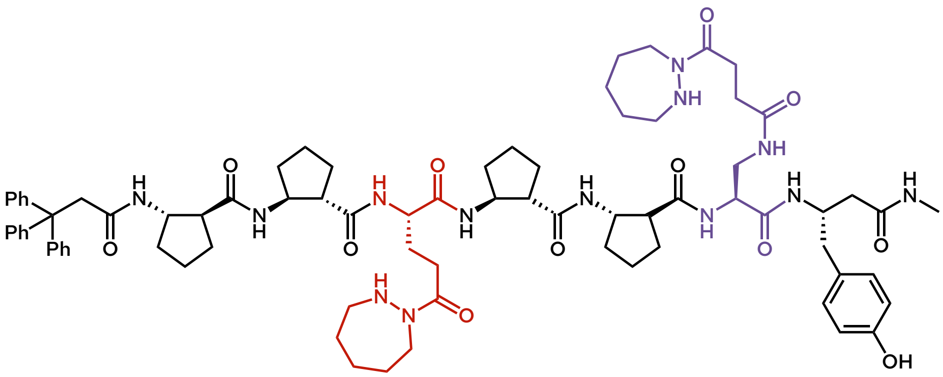

TPP-ACPC-ACPC-Glu(Hy)-ACPC-ACPC-Dap(SuccHy)-β^{3}HTyr-C(O)NHMe (9)

v_{rel} = 48

1 h yield = 88%

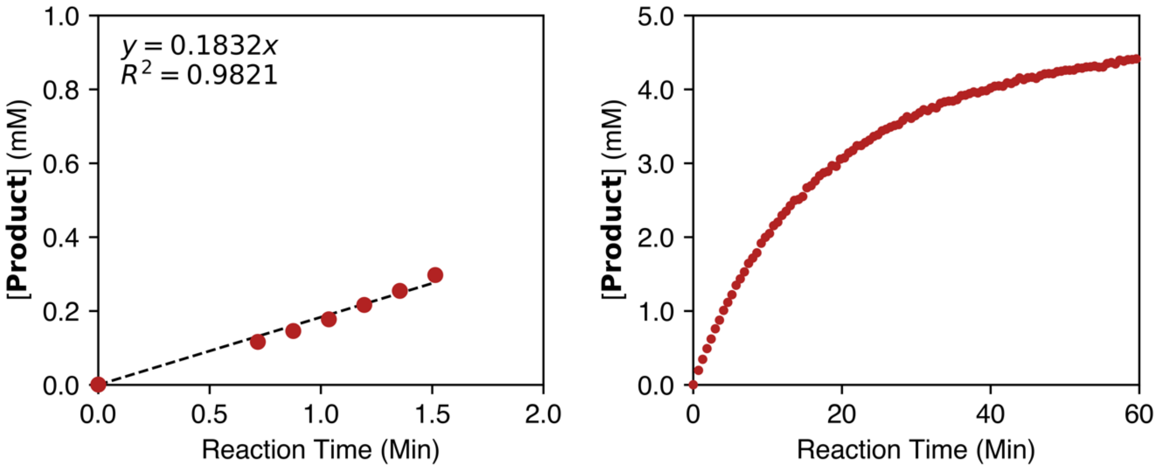

Figure S25. Initial rate and 1 h reaction profile for hydrocinnamaldehyde homoaldol reaction catalyzed by 5 mol% **9**.

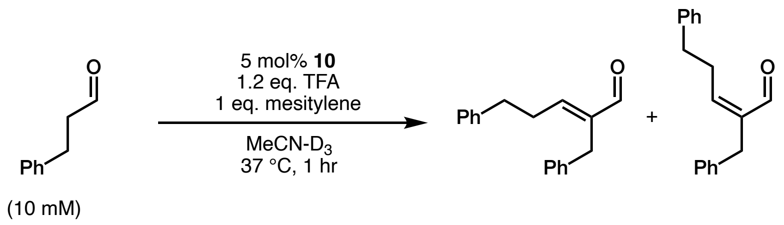

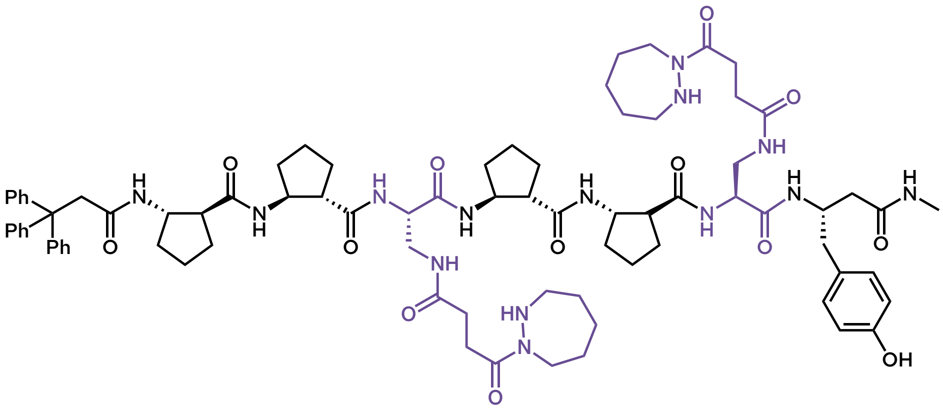

TPP-ACPC-ACPC-Dap(SuccHy)-ACPC-ACPC-Dap(SuccHy)-B^{3}HTyr-C(O)NHMe (10)

v_{rel} = 39

1 h yield = 85%

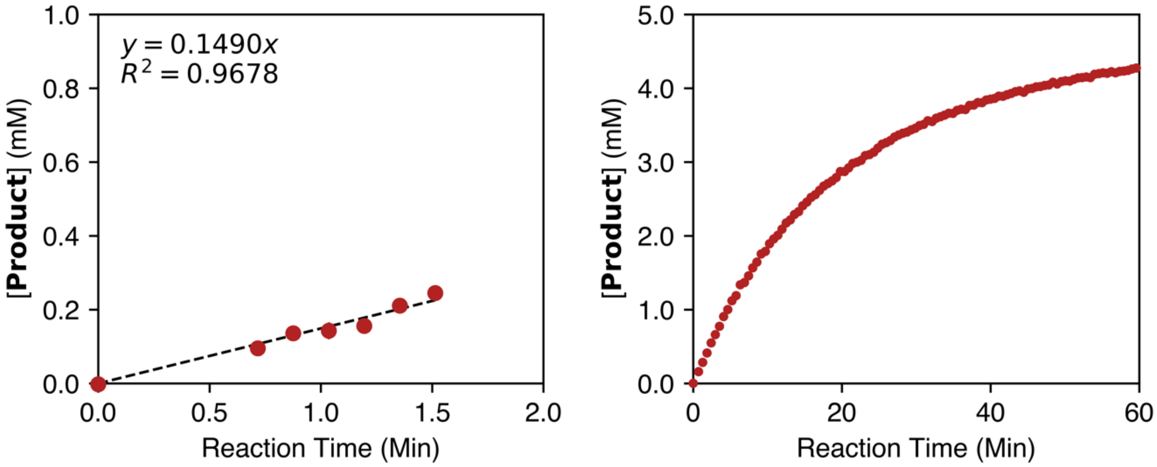

Figure S26. Initial rate and 1 h reaction profile for hydrocinnamaldehyde homoaldol reaction catalyzed by 5 mol% **10**.

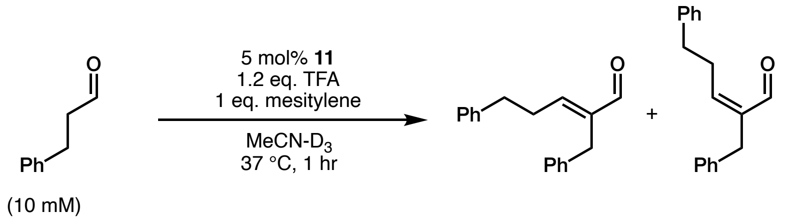

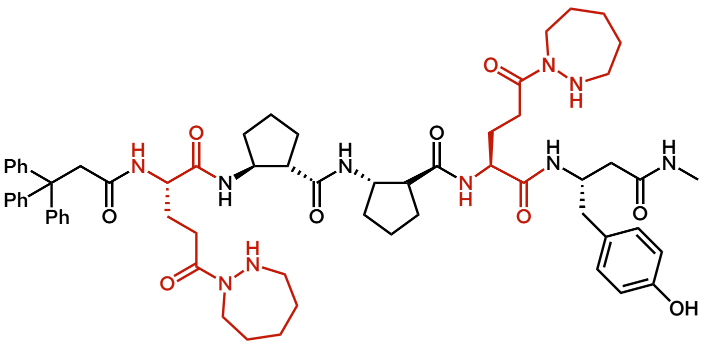

TPP-Glu(Hy)-ACPC-ACPC-Glu(Hy)-B[3]HTyr-C(O)NHMe (11)

v_{rel} = 39

1 h yield = 88%

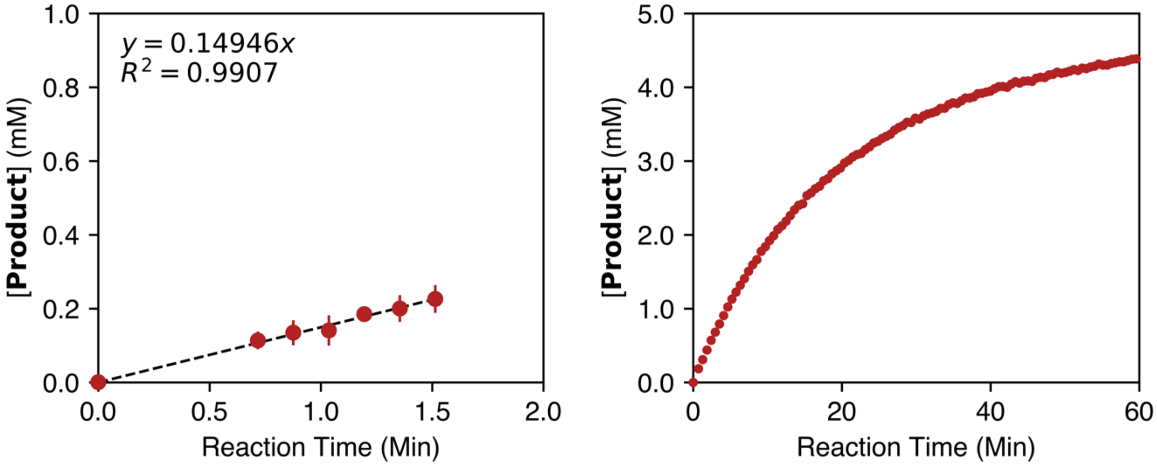

Figure S27. Initial rate and 1 h reaction profile for hydrocinnamaldehyde homoaldol reaction catalyzed by 5 mol% **11**.

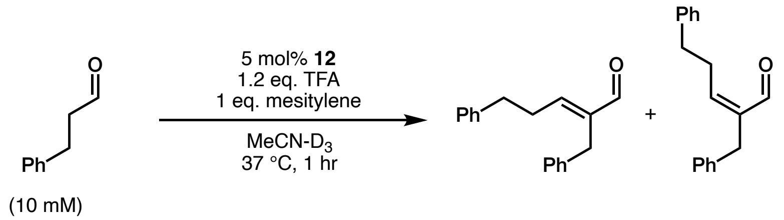

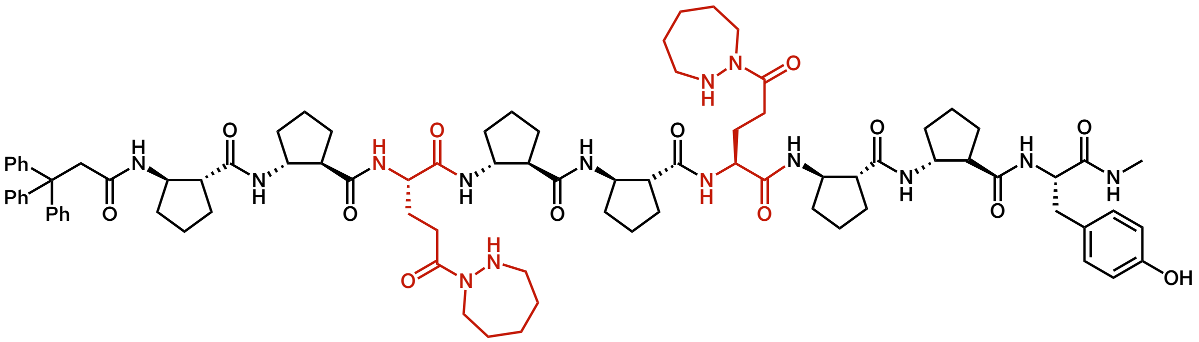

TPP-ACPC-ACPC-Glu(Hy)-ACPC-ACPC-Glu(Hy)-ACPC-ACPC-Tyr-C(O)NHMe (12)

v_{rel} = 77

1 h yield = 92%

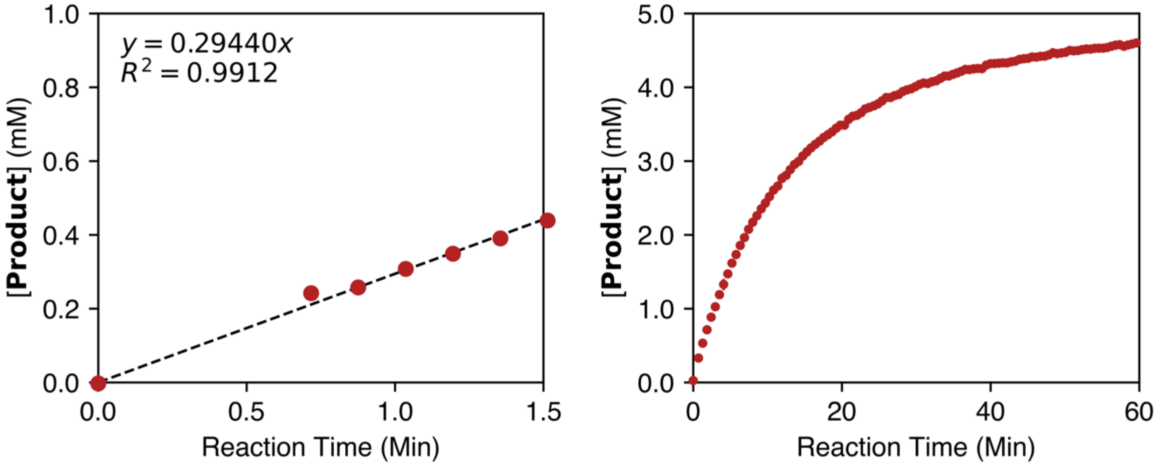

Figure S28. Initial rate and 1 h reaction profile for hydrocinnamaldehyde homoaldol reaction catalyzed by 5 mol% **12**.

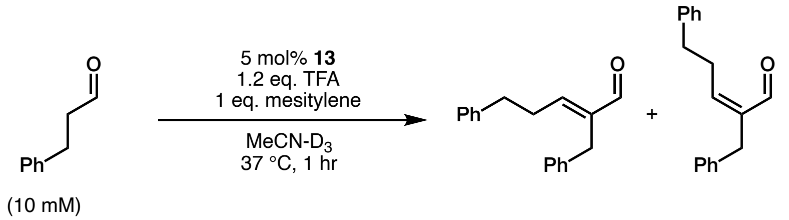

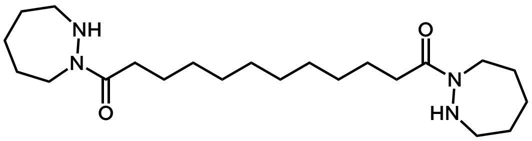

1,12-Di(1,2-diazepan-1-yl)dodecane-1,12-dione (13)

v_{rel} = 33

1 h yield = 93%

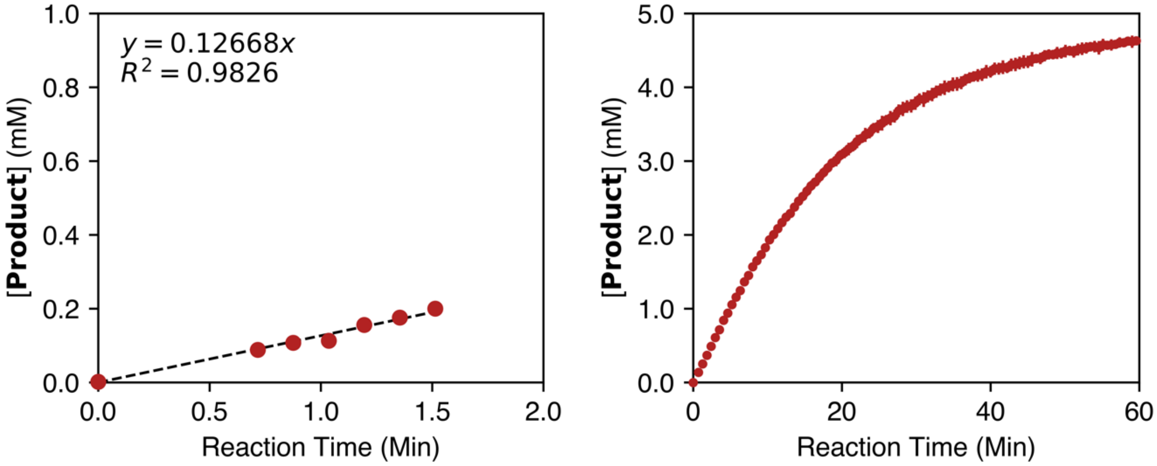

Figure S29. Initial rate and 1 h reaction profile for hydrocinnamaldehyde homoaldol reaction catalyzed by 5 mol% **13**.

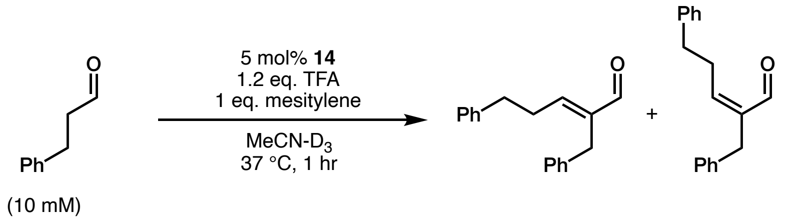

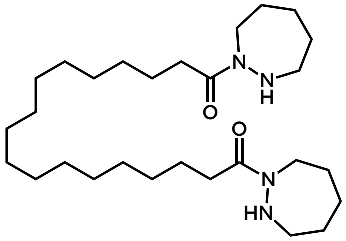

1,16-Di(1,2-diazepan-1-yl)hexadecane-1,16-dione (14)

$v_{rel} = 25$

1 h yield = 87%

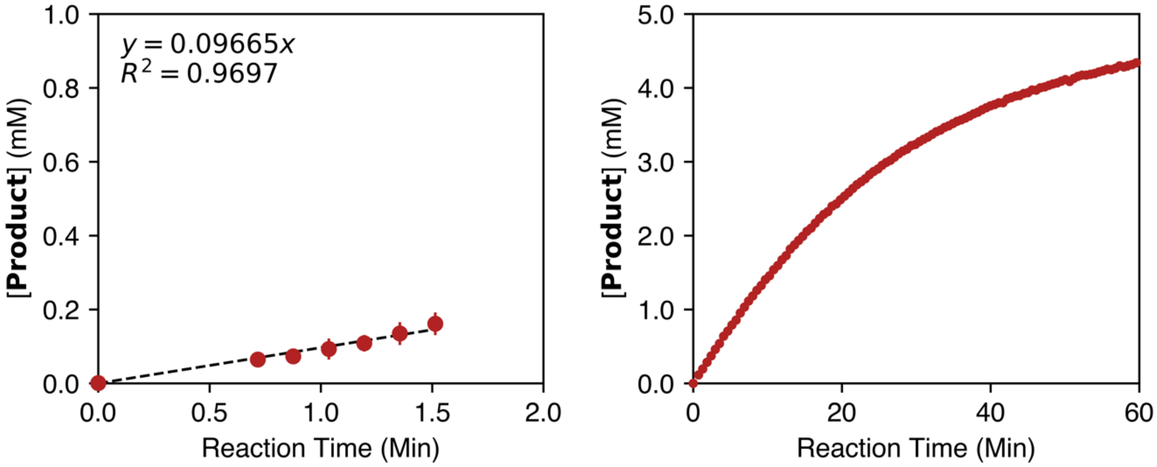

Figure S30. Initial rate and 1 h reaction profile for hydrocinnamaldehyde homoaldol reaction catalyzed by 5 mol% **14**.

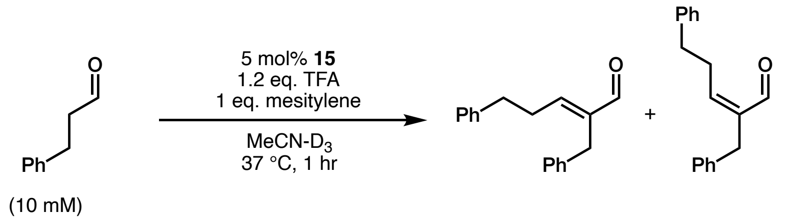

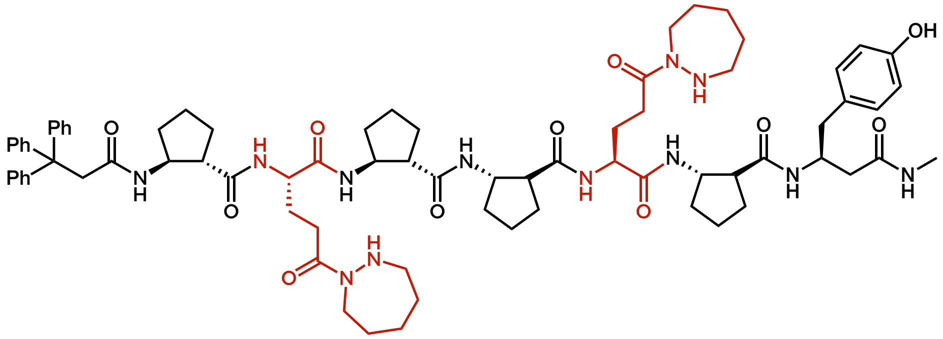

TPP-ACPC-Glu(Hy)-ACPC-ACPC-Glu(Hy)-ACPC-β³HTyr-C(O)NHMe (15)

v_{rel} = 93

1 h yield = 98%

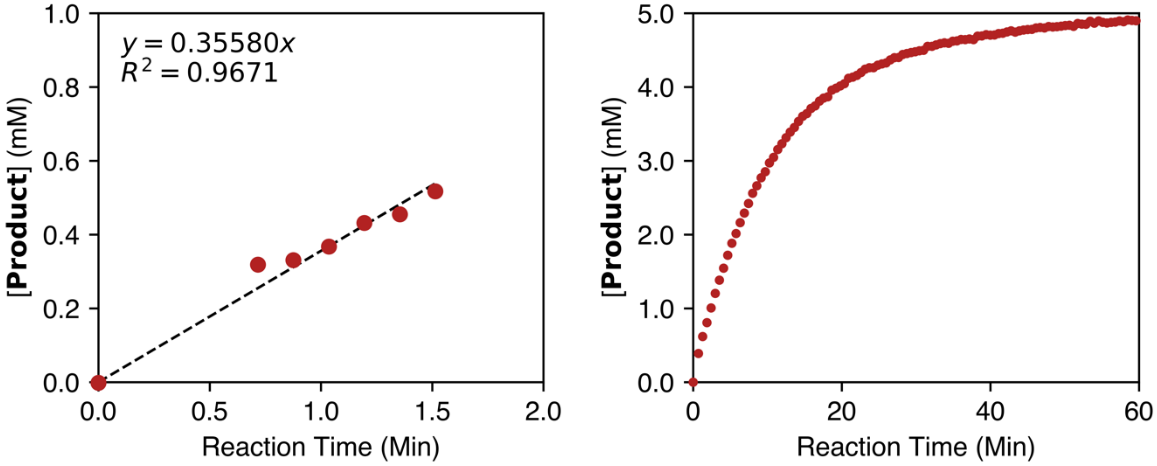

Figure S31. Initial rate and 1 h reaction profile for hydrocinnamaldehyde homoaldol reaction catalyzed by 5 mol% **15**.

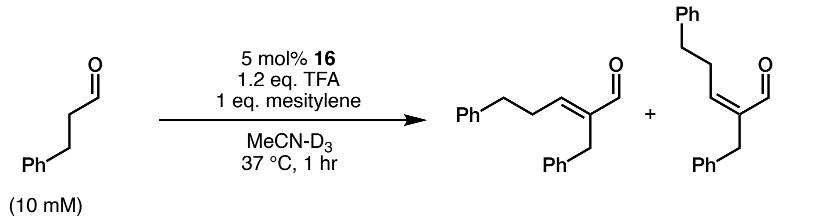

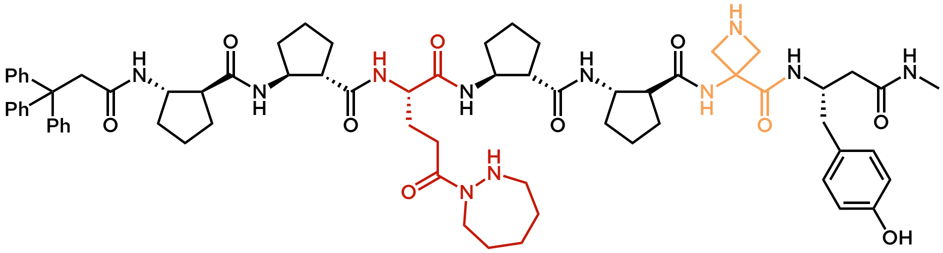

TPP-ACPC-ACPC-Glu(Hy)-ACPC-ACPC-Aze-B^{3}HTyr-C(O)NHMe (16)

$v_{rel} = 5$

1 h yield = 20%

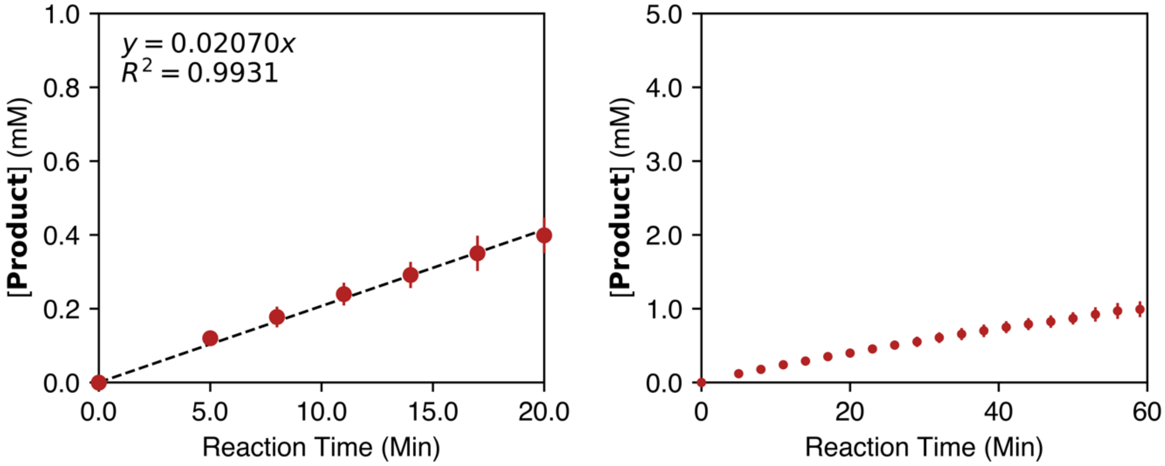

Figure S32. Initial rate and 1 h reaction profile for hydrocinnamaldehyde homoaldol reaction catalyzed by 5 mol% **16**.

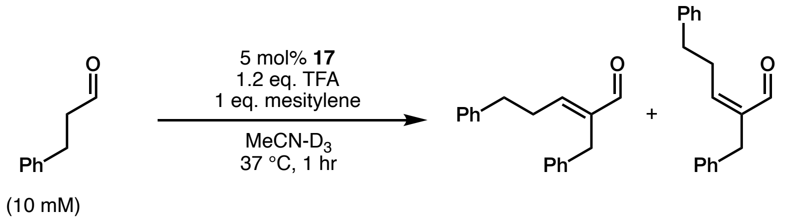

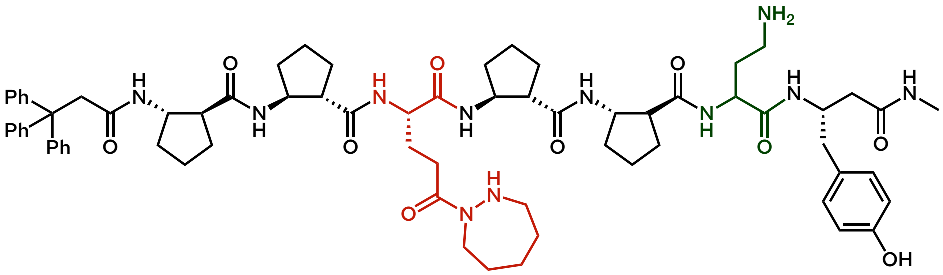

TPP-ACPC-ACPC-Glu(Hy)-ACPC-ACPC-Dab-β[3]HTyr-C(O)NHMe (17)

$v_{rel} = 2$

1 h yield = 4%

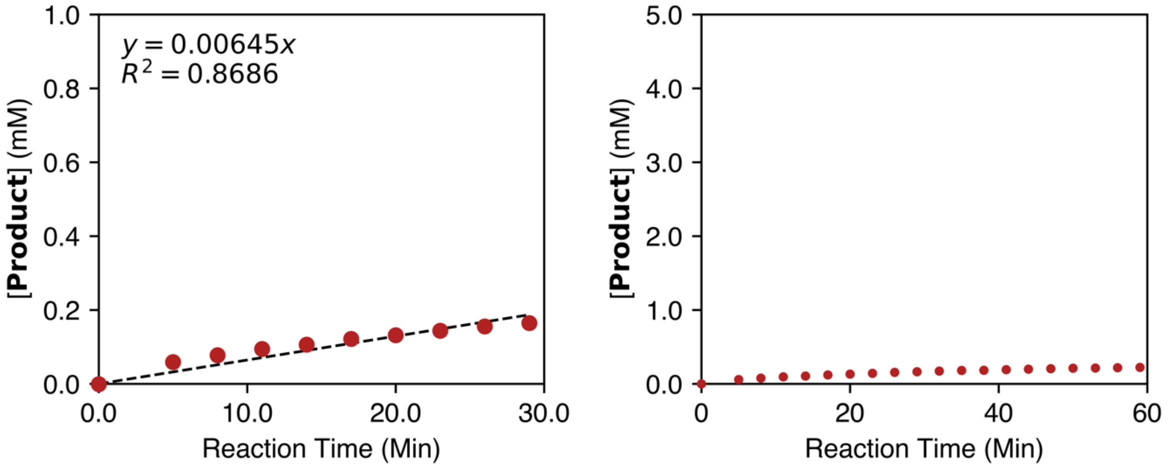

Figure S33. Initial rate and 1 h reaction profile for hydrocinnamaldehyde homoaldol reaction catalyzed by 5 mol% **17**.

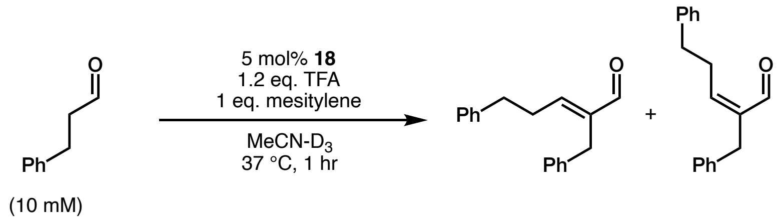

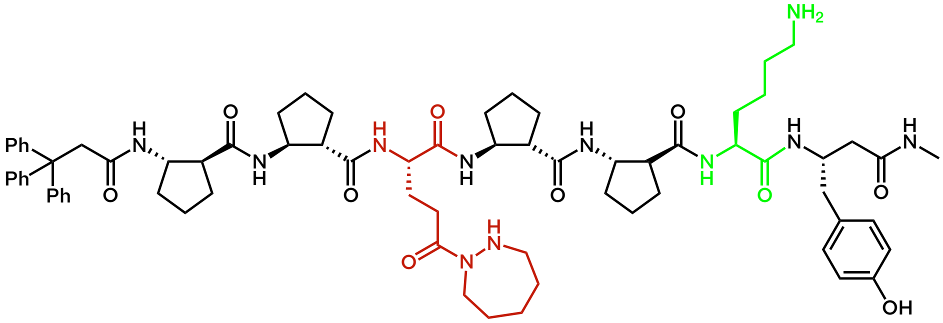

TPP-ACPC-ACPC-Glu(Hy)-ACPC-ACPC-Lys-β³HTyr-C(O)NHMe (18)

$v_{rel} = 2$

1 h yield = 5%

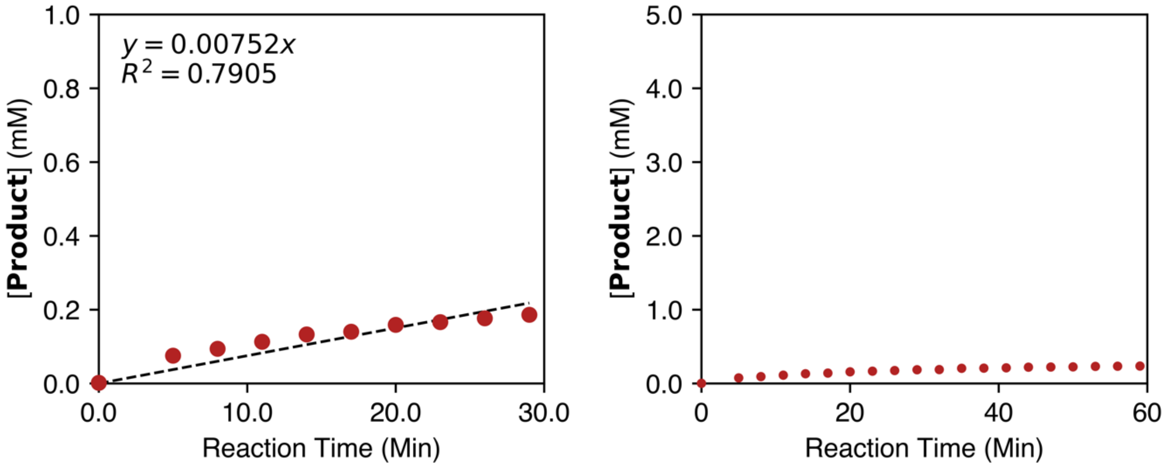

Figure S34. Initial rate and 1 h reaction profile for hydrocinnamaldehyde homoaldol reaction catalyzed by 5 mol% **18**.

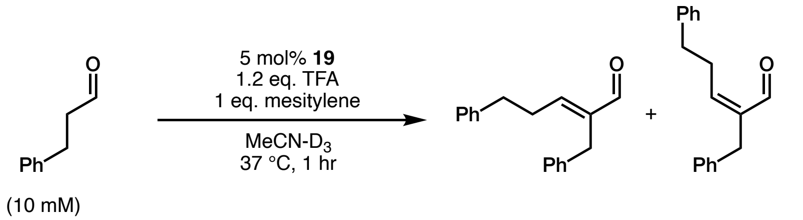

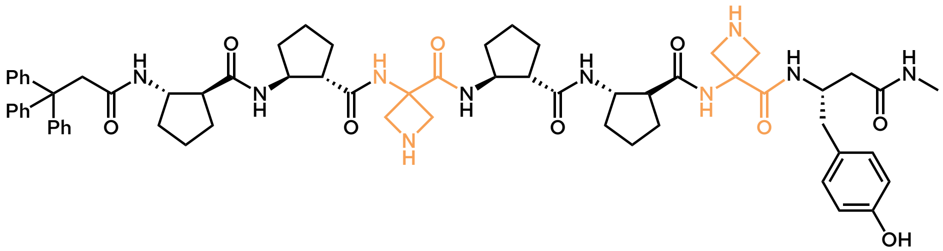

TPP-C(O)NH-ACPC-ACPC-Aze-ACPC-ACPC-Aze-B^{3}HTyr-C(O)NHMe (19)

v_{rel} = 0.3

1 h yield = 2%

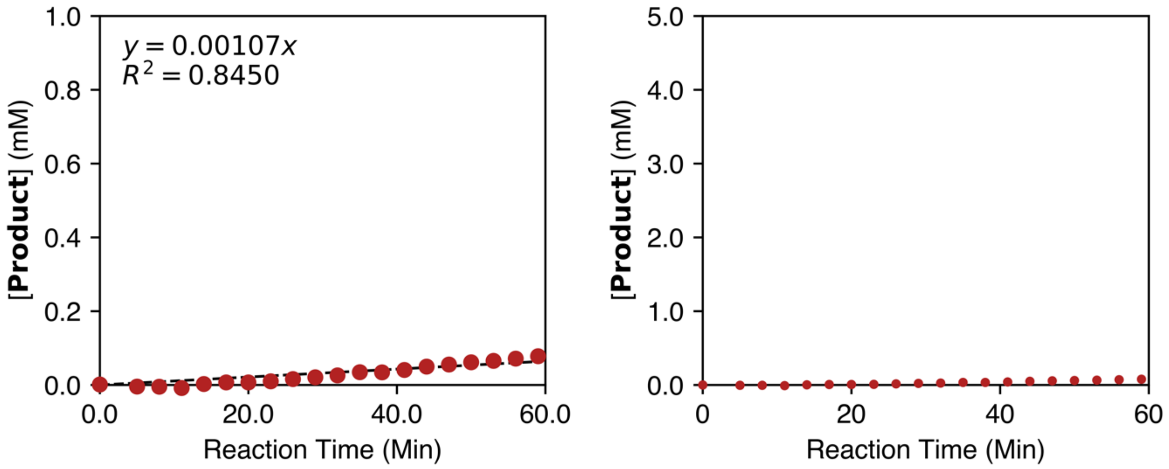

Figure S35. Initial rate and 1 h reaction profile for hydrocinnamaldehyde homoaldol reaction catalyzed by 5 mol% **19**.

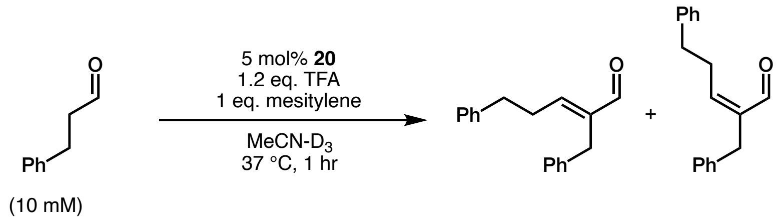

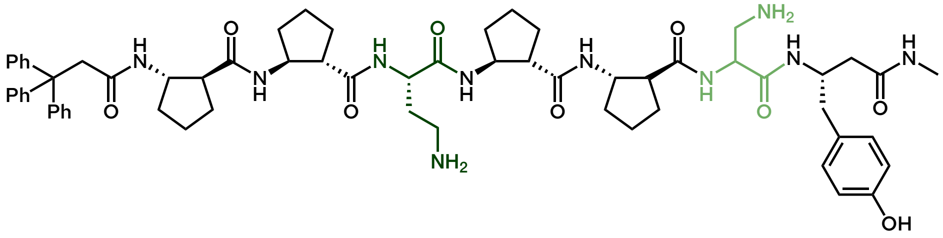

TPP- ACPC-ACPC-Dab-ACPC-ACPC-Dap-B³HTyr-C(O)NHMe (20)

v_{rel} = 0.0

1 h yield = 0%

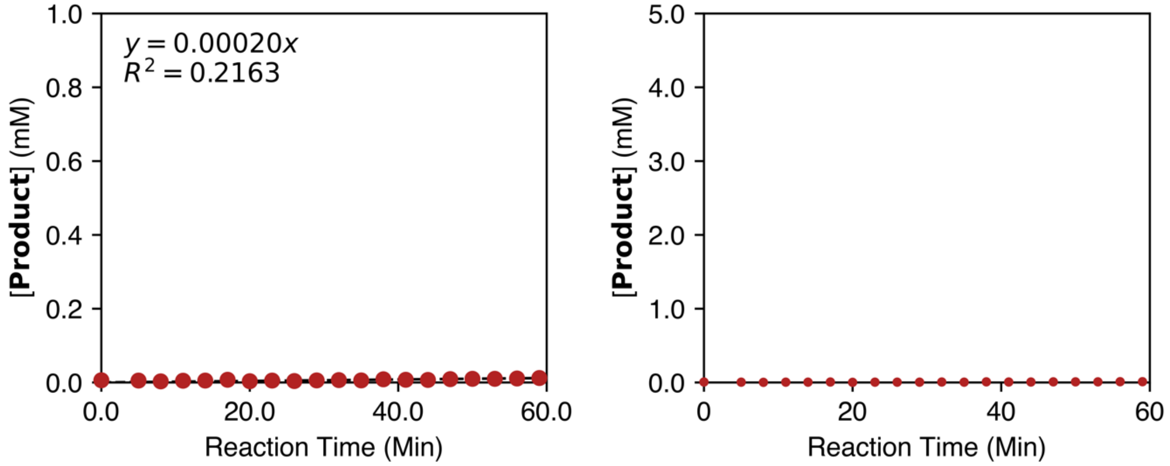

Figure S36. Initial rate and 1 h reaction profile for hydrocinnamaldehyde homoaldol reaction catalyzed by 5 mol% **20**.

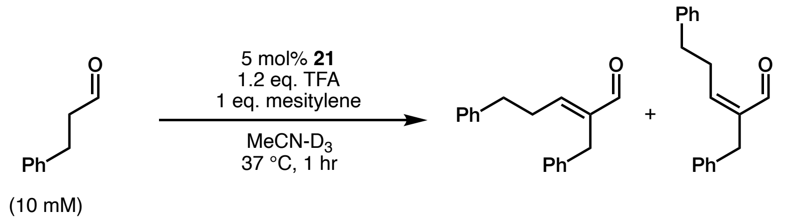

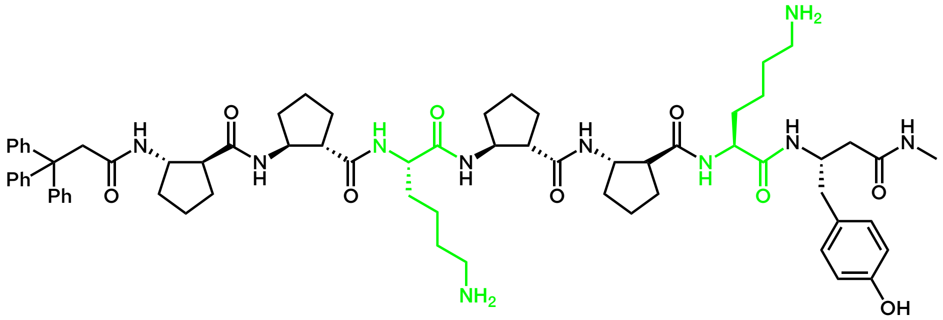

TPP-ACPC-ACPC-Lys-ACPC-ACPC-Lys-β³HTyr-C(O)NHMe (21)

$v_{rel} = 0.0$

1 h yield = 0%

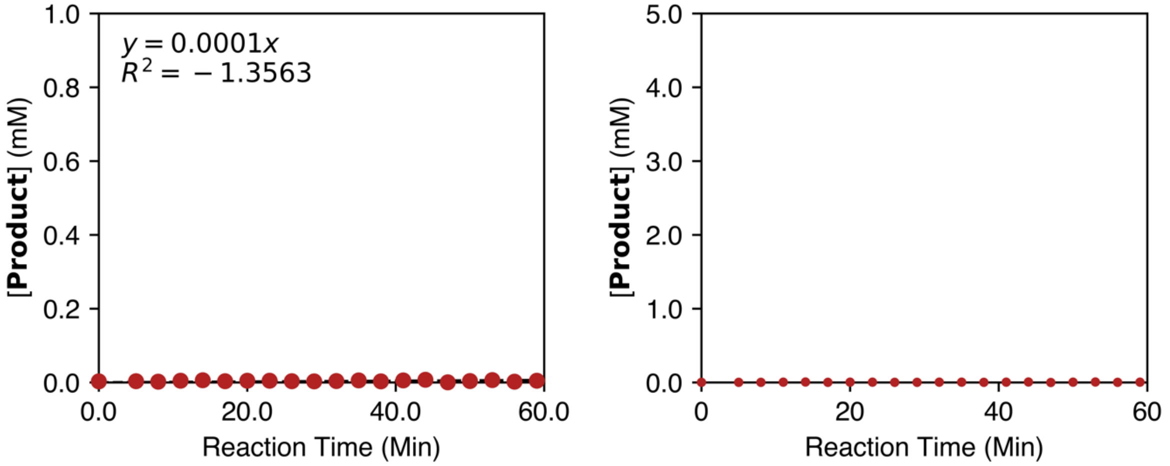

Figure S37. Initial rate and 1 h reaction profile for hydrocinnamaldehyde homoaldol reaction catalyzed by 5 mol% **21**.

4.3 Relative Rates and 2 h Yields Summary – Macrocyclization

Table S11. Summary of relative initial rates and 2 h reaction yields for catalysts of dialdehyde macrocyclization.

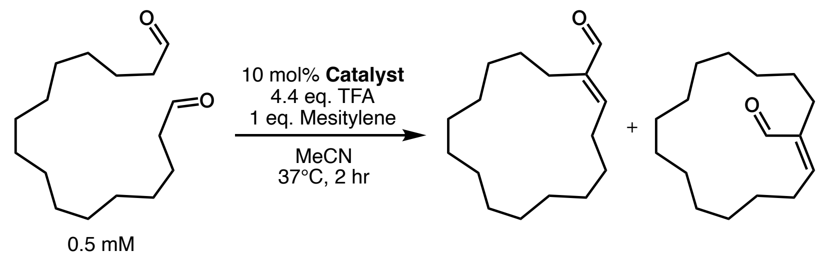

#	Catalyst	v_{rel}	Yield (%)
**1***	1-(1,2-Diazepan-1-yl)ethan-1-one	1	1
**5**	TPP-ACPC-ACPC-**Glu(Hy)**-ACPC-ACPC-**Glu(Hy)**-β^{3}HTyr-C(O)NHMe	319	79
**13**	1,12-Di(1,2-diazepan-1-yl)dodecane-1,12-dione	153	76

(*) = 10 mol% catalyst used.

4.4 Reaction Profiles and Initial Rates – Macrocyclization

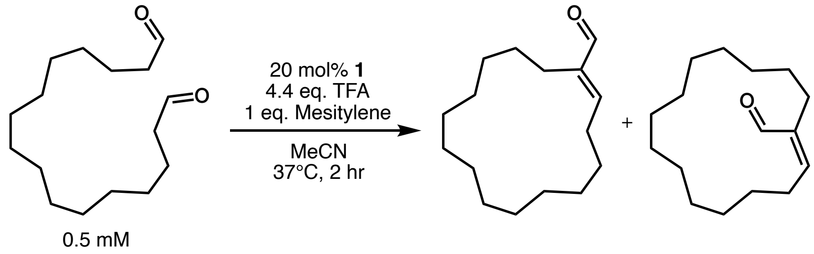

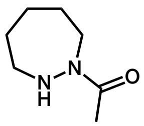

1-(1,2-Diazepan-1-yl)ethan-1-one (1)

$v_{rel} = 1$

1 h yield = 1%

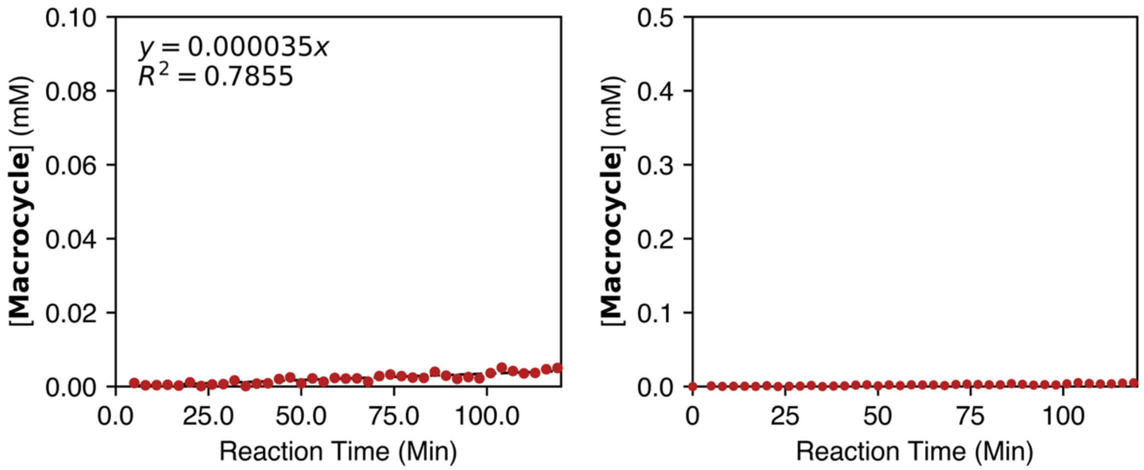

Figure S38. Initial rate and 2 h reaction profile for dialdehyde macrocyclization reaction catalyzed by 20 mol% **1**.

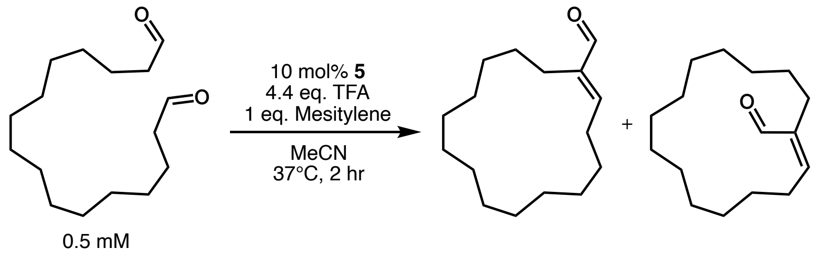

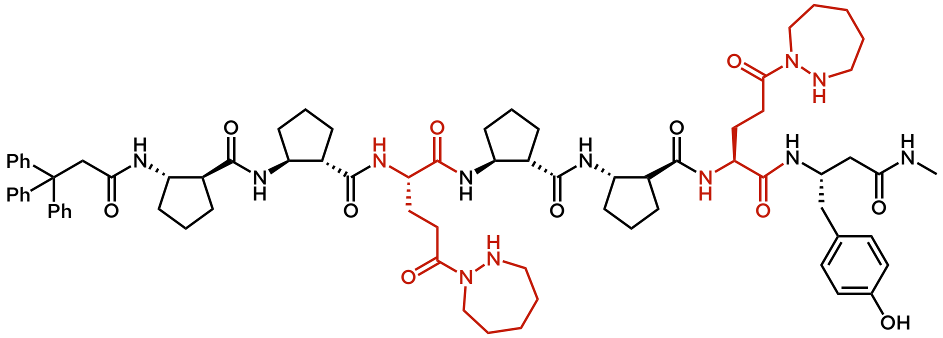

TPP-ACPC-ACPC-Glu(Hy)-ACPC-ACPC-Glu(Hy)-B³HTyr-C(O)NHMe (5)

v_{rel} = 319

1 h yield = 79%

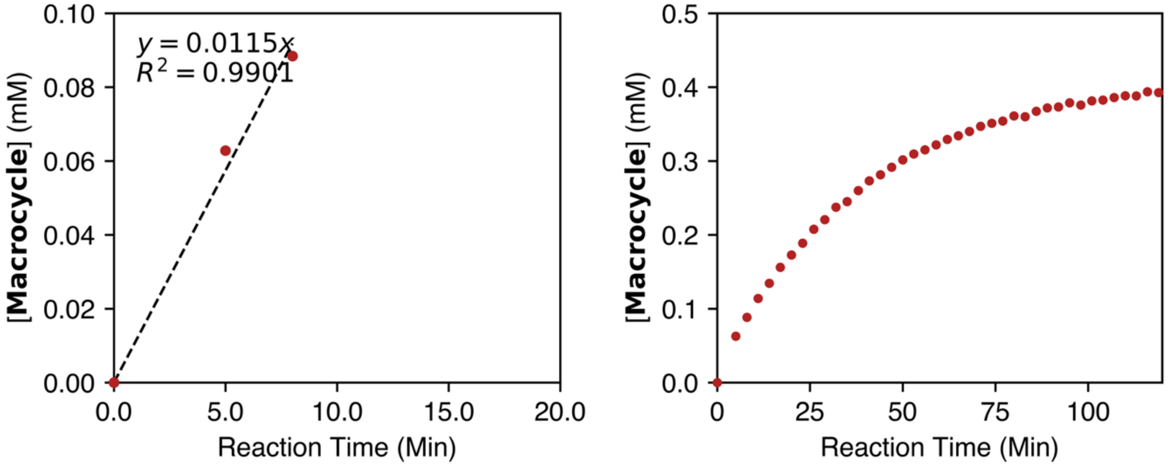

Figure S39. Initial rate and 2 h reaction profile for dialdehyde macrocyclization reaction catalyzed by 10 mol% **5**.

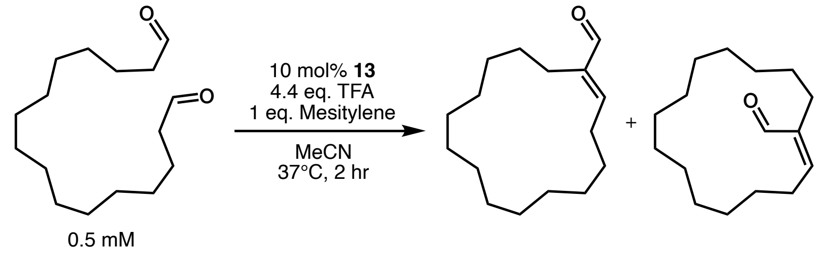

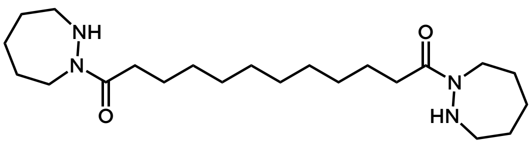

1,12-Di(1,2-diazepan-1-yl)dodecane-1,12-dione (13)

v_{rel} = 153

1 h yield = 76%

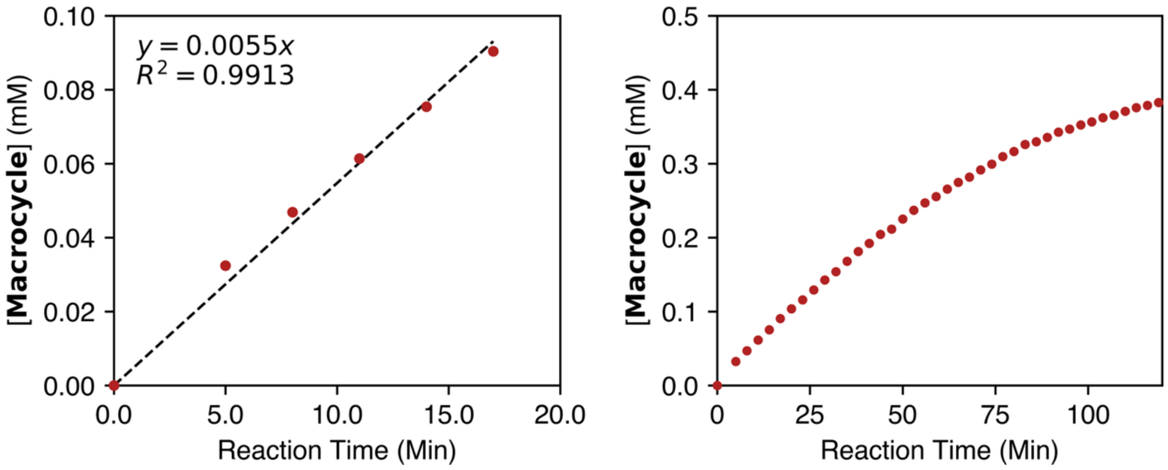

Figure S40. Initial rate and 2 h reaction profile for dialdehyde macrocyclization reaction catalyzed by 10 mol% **13**.

5. Crystallography Information

All crystal evaluation and data collection were performed on a Bruker D8 VENTURE PhotonIII four-circle diffractometer using MiTeGen MicroMounts. Olex2 was used to solve and refine all structures.

5.1 Crystal Growth Method for **S10** (Slow Evaporation)

To a glass vial containing ~400 mg of **S10** was added ~10 mL of $CHCl_3$ at 24 °C. The resulting solution was slowly concentrated under a gentle stream of N_2 until colorless crystals suitable for single crystal X-ray crystallography grew on the sides of the vial.

5.2 Crystal Growth Method for **5** and **12** (Slow Cooling)

Free amine foldamers **5** and **12** were prepared by dissolving ~10 mg of **5•2TFA** or **12•2TFA** in ~3 mL of LCMS-grade DCM and washing with 3 x 1 mL of saturated aqueous $NaHCO_3$. The DCM layer containing free amine foldamer was dried with anhydrous Na_2SO_4 and then dispensed into a clean 4 mL glass vial through a 0.45 µm syringe filter. The DCM was blown off under a stream of N_2, and the vial containing the free amine foldamer was left to sit in a vacuum desiccator for 72 h to remove any residual solvent.

To the glass vial containing the vacuum-dried free amine foldamer **5** or **12** was added ~0.5 mL of LCMS-grade acetonitrile at 24 °C. The vial was sealed with a Teflon cap and placed in a ~50 °C oven for ~5 minutes or until all solids had fully dissolved. The vial was then removed from the oven, placed in a thick styrofoam block with a 4 mL vial-sized cutout and allowed to slowly cool back to 24 °C. After ~24 h, colorless crystals of free amine foldamer **5** or **12** suitable for single crystal X-ray crystallography were obtained. Crystals of **5** and **12** could be handled outside the mother liquor without immediate degradation.

5.3 Crystal Growth Method for **11** (Liquid-Liquid Diffusion)

Free amine foldamer **11** (~10 mg) was prepared according to the procedure given in section 5.2.

Vacuum-dried free amine foldamer **11** was dissolved in ~0.3 mL of LCMS-grade DCM. The DCM solution was transferred to a 5 x 178 mm NMR sample tube via micropipette. To the NMR tube was then added ~2 mL of diethyl ether. The diethyl ether was slowly dispensed down the wall of the NMR tube using a syringe and needle to form a diethyl ether layer above the DCM solution containing free amine foldamer **11**. The NMR tube was carefully sealed with an air-tight plastic cap and left to stand upright on a vibration-free shelf at 24 °C. After ~4 weeks, several colorless crystals of free amine foldamer **11** suitable for single crystal X-ray crystallography were obtained. Crystals of **11** immediately degraded when handled outside the mother liquor.

5.4 Data Collection, Structural Solution and Refinement for **S10**

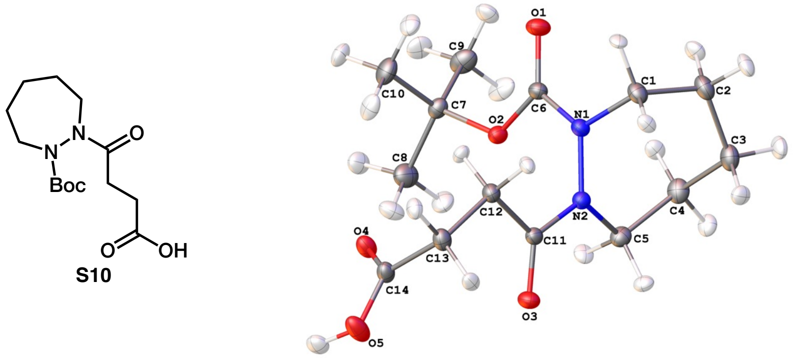

Figure S41. Chemical structure drawing and crystal structure of **S10** shown with 50% probability ellipsoids and all H atom labels omitted.

Data Collection

A colorless crystal with approximate dimensions 0.118 x 0.083 x 0.036 mm^3 was selected under oil under ambient conditions and attached to the tip of a MiTeGen MicroMount©. The crystal was mounted in a stream of cold nitrogen at 100(1) K and centered in the X-ray beam by using a video camera.

The crystal evaluation and data collection were performed on a Bruker D8 VENTURE PhotonIII four-circle diffractometer with Cu Kα (λ = 1.54178 Å) radiation and the detector to crystal distance of 5.0 cm.[4]

The initial cell constants were obtained from a 180° φ scan conducted at a 2θ = 50° angle with the exposure time of 1 second per frame. The reflections were successfully indexed by an automated indexing routine built in the APEX6 program. The final cell constants were calculated from a set of 9889 strong reflections from the actual data collection.

The data were collected by using the half sphere data collection routine to survey the reciprocal space to the extent of a half sphere to a resolution of 0.78 Å. A total of 18529 data were harvested by collecting 13 sets of frames with 0.9° scans in ω and φ with an exposure time 1–22 sec per frame. These highly redundant datasets were corrected for Lorentz and polarization effects. The absorption correction was based on fitting a function to the empirical transmission surface as sampled by multiple equivalent measurements.[5]

Structure Solution and Refinement

The systematic absences in the diffraction data were uniquely consistent for the space group $P2_1/c$ that yielded chemically reasonable and computationally stable results of refinement.[6-10]

A successful solution by intrinsic phasing provided most non-hydrogen atoms from the *E*-map. The remaining non-hydrogen atoms were located with an alternating series of least-squares cycles and difference Fourier maps. The atomic structure factors were determined by DFT calculations, using the r2SCAN hybrid functional and the cc-pVTZ basis set, in the NoSpherA2 extension of the olex2.refine program.[12-15] All atoms were refined with anisotropic atomic displacement coefficients.

The asymmetric unit contains one $C_{14}H_{24}N_2O_5$ molecule.

To yield chemically reasonable bond distances, the C-H distances for the C2–C4 atoms are restrained to have similar atomic distances. In addition, the C-H distances of the C8–C10 atoms are restrained to have similar atomic distances.

The final least squares refinement of 406 parameters against 3426 data resulted in residuals R (based on F^2 for $I \geq 2\sigma$) and wR (based on F^2 for all data) of 0.0140 and 0.0316, respectively. The final difference Fourier map was featureless.

Summary

Crystal Data for $C_{14}H_{24}N_2O_5$ (M =300.357 g/mol): monoclinic, space group $P2_1/c$ (no. 14), a = 14.509(6) Å, b = 10.541(2) Å, c = 11.268(3) Å, β = 112.524(16)°, V = 1591.9(9) Å^3, Z = 4, T = 100.00 K, μ(Cu Kα) = 0.791 mm^{-1}, $Dcalc$ = 1.253 g/cm^3, 17881 reflections measured (6.6° ≤ 2Θ ≤ 159.12°), 3426 unique (R_{int} = 0.0340, R_{sigma} = 0.0239) which were used in all calculations. The final R_1 was 0.0140 (I>=2u(I)) and wR_2 was 0.0316 (all data).

Table S12. Overview of crystallography-related parameters for **S10**.

Deposition number	2466655
Empirical formula	$C_{14}H_{24}N_2O_5$
Formula weight	300.357
Temperature/K	100.00
Crystal system	monoclinic
Space group	$P2_1/c$
a/Å	14.509(6)
b/Å	10.541(2)
c/Å	11.268(3)
α/°	90
β/°	112.524(16)
γ/°	90
Volume/Å^3	1591.9(9)
Z	4
ρ_{calc}g/cm^3	1.253
μ/mm^{-1}	0.791
F(000)	650.4
Crystal size/mm^3	0.118 × 0.083 × 0.036
Radiation	Cu Kα (λ = 1.54178)
2Θ range for data collection/°	6.6 to 159.12
Index ranges	$-17 \leq h \leq 18$, $-12 \leq k \leq 13$, $-14 \leq l \leq 14$
Reflections collected	17881
Independent reflections	3426 [R_{int} = 0.0340, R_{sigma} = 0.0239]
Data/restraints/parameters	3426/15/406
Goodness-of-fit on F^2	1.131
Final R indexes [I>=2σ (I)]	R_1 = 0.0140, wR_2 = 0.0308
Final R indexes [all data]	R_1 = 0.0162, wR_2 = 0.0316
Largest diff. peak/hole / e Å^{-3}	0.12/-0.10

5.5 Data Collection, Structural Solution and Refinement for **5**

5 – TPP-ACPC-ACPC-**Glu(Hy)**-ACPC-ACPC-**Glu(Hy)**-β^{3}hTyr-C(O)NHMe (heptamer)

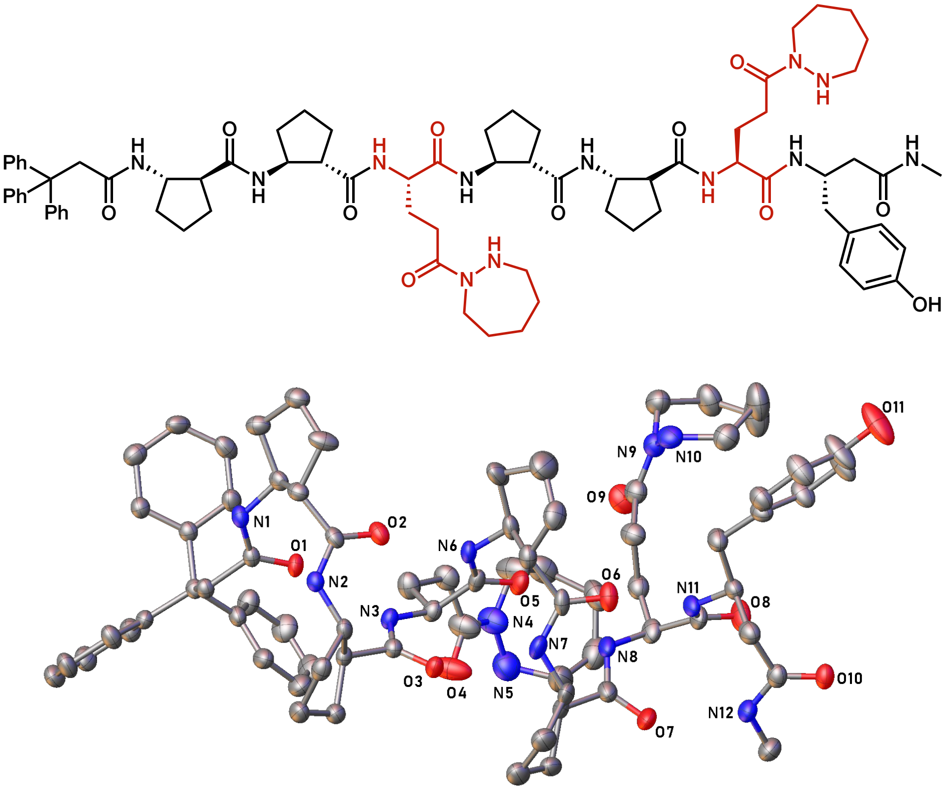

Figure S42. Chemical structure drawing and crystal structure for the "no suffix" molecule of **5** shown with 50% probability ellipsoids. All H atoms and minor disorder components are omitted.

Data Collection

A colorless crystal with approximate dimensions 0.2 × 0.05 × 0.05 mm^3 was selected under oil under ambient conditions and attached to the tip of a MiTeGen MicroMount©. The crystal was mounted in a stream of cold nitrogen at 100(1) K and centered in the X-ray beam by using a video camera.

The crystal evaluation and data collection were performed on a Bruker D8 VENTURE PhotonIII four-circle diffractometer with Cu Kα (λ = 1.54178 Å) radiation and the detector to crystal distance of 5.0 cm.[4]

The initial cell constants were obtained from a 180° ϕ scan conducted at a 2θ = 50° angle with the exposure time of 1 second per frame. The reflections were successfully indexed by an automated indexing routine built in the APEX3 program. The final cell constants were calculated from a set of 9832 strong reflections from the actual data collection.

The data were collected by using the full sphere data collection routine to survey the reciprocal space to the extent of a full sphere to a resolution of 0.80 Å. A total of 366837 data were harvested by collecting 48 sets of frames with 0.8° scans in ω and ϕ with an exposure time 2–16 sec per frame. These redundant datasets were corrected for Lorentz and polarization effects. The absorption correction was based on fitting a function to the empirical transmission surface as sampled by multiple equivalent measurements.[5]

Structure Solution and Refinement

The systematic absences in the diffraction data were consistent for the space groups $P\bar{1}$ and $P1$. The E-statistics strongly suggested the non-centrosymmetric space group $P1$ that yielded chemically reasonable and computationally stable results of refinement.[6-11]

A successful solution by intrinsic phasing provided most non-hydrogen atoms from the E-map. The remaining non-hydrogen atoms were located in an alternating series of least-squares cycles and difference Fourier maps. All non-hydrogen atoms were refined with anisotropic displacement coefficients unless specified otherwise. All hydrogen atoms were included in the structure factor calculation at idealized positions and were allowed to ride on the neighboring atoms with relative isotropic displacement coefficients.

The absolute structure was unequivocally established by resonant scattering effects. All chiral centers are *S*.

The asymmetric unit contains five symmetry-independent foldamers and several (probably eight) molecules of solvent MeCN.

All molecules were labeled in a similar fashion. No information is provided for the solvent molecules.

There is positional disorder in every molecule.

First molecule (labels without a suffix): the five-membered ring between N7 and C55 is disordered over two positions with the major component contribution of 0.65(3). The rings were refined with geometrical restraints and the atoms with atomic displacement parameter restraints.

Molecule A (labels with suffix A): a) the C8A Ph ring is disordered over two positions with the major component contribution of 0.86(3). The minor disorder component was refined isotropically with an idealized geometry and fixed atomic displacement parameters; b) the atom sequence between N7a and N11 is disordered over two positions with the major component contribution of 0.739(5). The disorder was refined with geometrical restraints. The major component was refined with atomic displacement parameter restraints, whereas the minor disorder component with fixed atomic displacement parameters.

Molecule B: a) the C14B Ph ring is disordered over two positions with the major component contribution of 0.73(3). Both disorder components were refined with an idealized geometry. The minor component was refined isotropically; b) atoms C45B and C46B are disordered over two positions with the major component contribution of 0.599(19). The fragments were refined with geometrical restraints and the atoms with atomic displacement parameter restraints; c) the C57B chain at atom C56B is disordered over two positions with the major component contribution of 0.577(8). The fragments were refined with geometrical restraints and the atoms with atomic displacement parameter restraints whereas selected atoms with atomic displacement parameter constraints. Also, the C1B ring was refined with an idealized geometry.

Molecule C: a) atom C30C is disordered over two positions with the major component contribution of 0.73(3). The fragment was refined with geometrical restraints and the atoms with atomic displacement parameter restraints; b) atoms C39C–C41C are disordered over two positions with the major component contribution of 0.837(14). The fragment was refined with geometrical restraints and the atoms with atomic displacement parameter restraints; c) the C57C chain at atom C56C is equally disordered over two positions. The fragments were refined with geometrical restraints and the atoms with atomic displacement parameter restraints.

Molecule D: atoms N10D and C61D are disordered over two positions with the major component contribution of 0.55(6). The fragments were refined with geometrical restraints and the atoms with atomic displacement parameter restraints.

There were several fully and partially occupied solvent molecules of MeCN present in the asymmetric unit. Three of them could be identified, but others could not. A significant amount of time was invested in identifying and refining the disordered molecules. Bond length restraints were applied to model the molecules but the resulting isotropic displacement coefficients suggested the molecules were mobile. In addition, the refinement was computationally unstable. Option Mask of program OLEX2[10] was used to correct the diffraction data for diffuse scattering effects and to calculate the number of solvent molecules. Note that all solvent molecules were excluded from the model. OLEX2 calculated the upper limit of volume that can be occupied by the solvent to be 855 $Å^3$, or ~9 % of the unit cell volume. The program calculated 179 electrons in the unit cell for the diffuse species. This approximately corresponds to eight molecules of MeCN in the asymmetric unit (176 electrons). It is very likely that many solvent molecules are disordered over several positions forming hydrogen bonds. Please note that all derived results in the following table are based on the known contents. No data are given for the diffusely scattering species.

The final least-squares refinement of 4784 parameters against 77295 data resulted in residuals R (based on F^2 for $I \geq 2\sigma$) and wR (based on F^2 for all data) of 0.0767 and 0.2111, respectively. The final difference Fourier map was featureless.

Summary

Crystal Data for $C_{76}H_{102}N_{12}O_{11}$ (M =1359.69 g/mol): triclinic, space group P1 (no. 1), a = 13.831(2) Å, b = 14.780(2) Å, c = 50.433(10) Å, α = 90.670(10)°, β = 97.195(10)°, γ = 111.175(14)°, V = 9519(3) $Å^3$, Z = 5, T = 100.00 K, μ(Cu Kα) = 0.647 mm^{-1}, $Dcalc$ = 1.186 g/cm^3, 366837 reflections measured (5.308° ≤ 2Θ ≤ 158.948°), 77295 unique (R_{int} = 0.0680, R_{sigma} = 0.0511) which were used in all calculations. The final R_1 was 0.0767 (I > 2σ(I)) and wR_2 was 0.2111 (all data).

Table S13. Overview of crystallography-related parameters for foldamer **5**.

Deposition number	2466658
Empirical formula	$C_{76}H_{102}N_{12}O_{11}$·solvent
Formula weight	1359.69
Temperature/K	100.00
Crystal system	triclinic
Space group	P1
a/Å	13.831(2)
b/Å	14.780(2)
c/Å	50.433(10)
α/°	90.670(10)
β/°	97.195(10)
γ/°	111.175(14)
Volume/Å^3	9519(3)
Z	5
ρ_{calc}g/cm^3	1.186
μ/mm^{-1}	0.647
F(000)	3650.0
Crystal size/mm^3	0.2 × 0.05 × 0.05
Radiation	Cu Kα (λ = 1.54178)
2Θ range for data collection/°	5.308 to 158.948
Index ranges	-17 ≤ h ≤ 17, -18 ≤ k ≤ 18, -63 ≤ l ≤ 64
Reflections collected	366837
Independent reflections	77295 [R_{int} = 0.0680, R_{sigma} = 0.0511]
Data/restraints/parameters	77295/1628/4784
Goodness-of-fit on F^2	1.024
Final R indexes [I>=2σ (I)]	R_1 = 0.0767, wR_2 = 0.2006
Final R indexes [all data]	R_1 = 0.0894, wR_2 = 0.2111
Largest diff. peak/hole / e Å^{-3}	0.66/-0.59
Flack parameter	0.15(10)

5.6 Data Collection, Structural Solution and Refinement for **11**

11 – TPP-**Glu(Hy)**-ACPC-ACPC-**Glu(Hy)**-β^3hTyr-C(O)NHMe (pentamer)

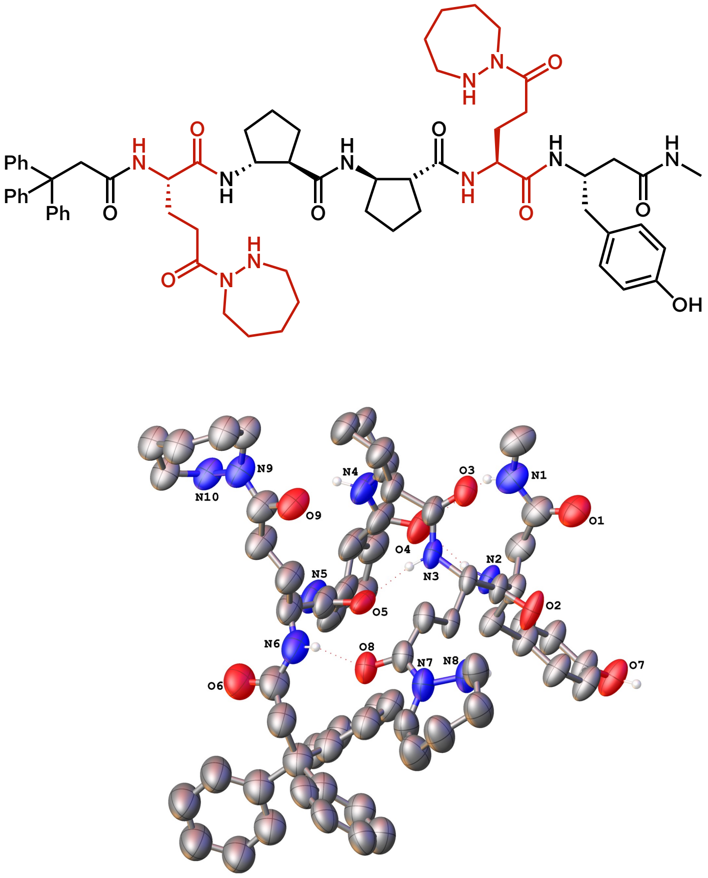

Figure S43. Chemical structure drawing and crystal structure for the entire complex in **11** shown with 30% probability ellipsoids. Only H atoms bound to hetero atoms are shown.

Data Collection

A colorless crystal with approximate dimensions 0.10 x 0.10 x 0.02 mm^3 was selected under oil under ambient conditions and attached to the tip of a MiTeGen MicroMount©. The crystal was mounted in a stream of cold nitrogen at 100(1) K and centered in the X-ray beam by using a video camera.

The crystal evaluation and data collection were performed on a Bruker D8 VENTURE PhotonIII four-circle diffractometer with Cu K_α (λ = 1.54178 Å) radiation and the detector to crystal distance of 5.0 cm.[4]

The initial cell constants were obtained from a 180° ϕ scan conducted at a 2θ = 50° angle with an exposure time of 1 second per frame. The reflections were successfully indexed by an automated indexing routine built into the APEX3 program. The final cell constants were calculated from a set of 9933 strong reflections from the actual data collection.

The data were collected by using a full sphere data collection routine to survey reciprocal space to the extent of a full sphere to a resolution of 1.10 Å. A total of 39865 data were harvested by collecting 8 sets of frames with 0.8° scans in ω and ϕ with exposure times of 3-90 sec per frame. These highly redundant datasets were corrected for Lorentz and polarization effects. The absorption correction was

based on fitting a function to the empirical transmission surface as sampled by multiple equivalent measurements.[5]

Structure Solution and Refinement

The systematic absences in the diffraction data were uniquely consistent for the chiral space group $P2_12_12$ that yielded chemically reasonable and computationally stable results of refinement.[6-11]

A successful solution by intrinsic phasing provided most non-hydrogen atoms from the *E*-map. The remaining non-hydrogen atoms were located with an alternating series of least-squares cycles and difference Fourier maps. All non-hydrogen atoms were refined with anisotropic displacement coefficients. All hydrogen atoms were included in the structure factor calculation at idealized positions and were allowed to ride on the neighboring atoms with relative isotropic displacement coefficients.

The configuration of the chiral atoms was assigned according to the synthesis: C4–*S*, C6–*S*, C8–*S*, C12–*S*, C14–*S*, C18–*S*, and C20–*S*.

The entire molecule was refined with a combination of geometric restraints and constraints as well as atomic displacement parameter restraints.

There are three solvent accessible voids containing diffuse species in the asymmetric unit. A significant amount of time was invested in identifying and refining the molecules. Idealized geometries[12] were applied to model the molecules, but the resulting isotropic displacement coefficients suggested the molecules were mobile. The masking feature in Olex2 was used to correct the diffraction data for diffuse scattering effects and to identify the solvate molecule. The calculated upper limits of volume that can be occupied by a solvent is 16, 196, and 332 Å^3, respectively. The program calculated 12, 48 and 86 electrons per void space for a total of 146 electrons in the asymmetric cell for the diffuse species. This approximately corresponds to three molecules of DCM and two molecules of water in the asymmetric unit (146 electrons). Please note that all derived results in the following table are based on the known contents. No data are given for the diffusely scattering species.

The final least-squares refinement of 702 parameters against 5913 data resulted in residuals R (based on F^2 for $I \geq 2\sigma$) and wR (based on F^2 for all data) of 0.1029 and 0.3319, respectively. The final difference Fourier map was featureless.

Summary

Crystal Data for $C_{64}H_{84}N_{10}O_9$ (M =1137.41 g/mol): orthorhombic, space group $P2_12_12$ (no. 18), a = 27.316(9) Å, b = 27.870(7) Å, c = 9.881(3) Å, V = 7522(4) Å^3, Z = 4, T = 100.00 K, μ(Cu Kα) = 0.546 mm^{-1}, $Dcalc$ = 1.004 g/cm^3, 39713 reflections measured (7.208° ≤ 2Θ ≤ 89.18°), 5913 unique (R_{int} = 0.0881, R_{sigma} = 0.0511) which were used in all calculations. The final R_1 was 0.1029 (I > 2σ(I)) and wR_2 was 0.3319 (all data).

Table S14. Overview of crystallography-related parameters for foldamer **11**.

Deposition number	2466656
Empirical formula	$C_{64}H_{84}N_{10}O_9$
Formula weight	1137.41
Temperature/K	100.00
Crystal system	orthorhombic
Space group	$P2_12_12$
a/Å	27.316(9)
b/Å	27.870(7)
c/Å	9.881(3)
α/°	90
β/°	90
γ/°	90
Volume/Å^3	7522(4)
Z	4
ρ_{calc}g/cm^3	1.004
μ/mm^{-1}	0.546
F(000)	2440.0
Crystal size/mm^3	0.1 × 0.1 × 0.02
Radiation	Cu Kα (λ = 1.54178)
2Θ range for data collection/°	7.208 to 89.18
Index ranges	-24 ≤ h ≤ 23, -25 ≤ k ≤ 25, -8 ≤ l ≤ 8
Reflections collected	39713
Independent reflections	5913 [R_{int} = 0.0881, R_{sigma} = 0.0511]
Data/restraints/parameters	5913/1514/702
Goodness-of-fit on F^2	1.351
Final R indexes [I>=2σ (I)]	R_1 = 0.1029, wR_2 = 0.3149
Final R indexes [all data]	R_1 = 0.1194, wR_2 = 0.3319
Largest diff. peak/hole / e Å^{-3}	0.31/-0.24
Flack parameter	2.1(2)

5.7 Data Collection, Structural Solution and Refinement for **12**

12 – TPP-ACPC-ACPC-Glu(Hy)-ACPC-ACPC-Glu(Hy)-ACPC-ACPC–Tyr-C(O)NHMe (nonamer)

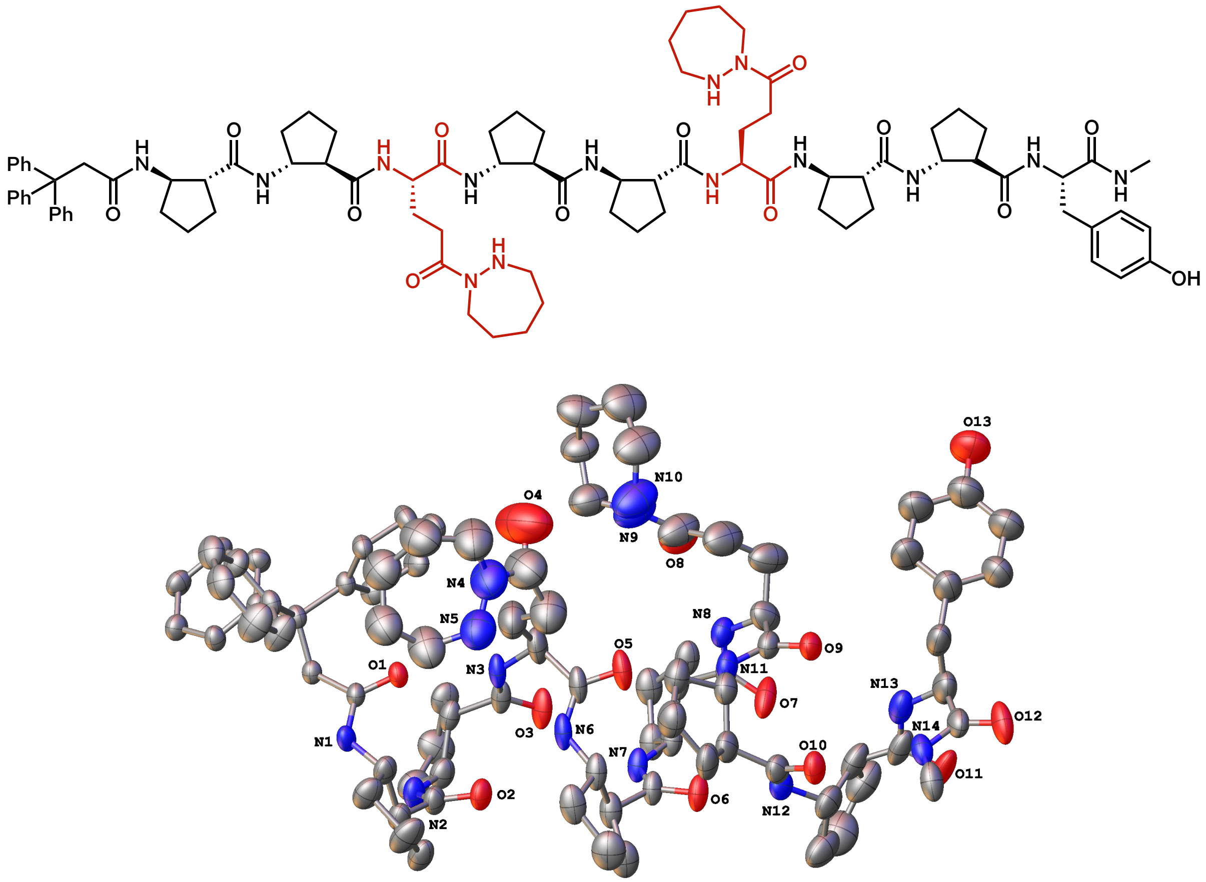

Figure S44. Chemical structure drawing and crystal structure for the “1” foldamer in **12** shown with 30 % probability ellipsoids. All H atoms and minor disorder components are omitted.

Data Collection

A colorless crystal with approximate dimensions 0.1 × 0.05 × 0.05 mm^3 was selected under oil under ambient conditions and attached to the tip of a MiTeGen MicroMount©. The crystal was mounted in a stream of cold nitrogen at 100(1) K and centered in the X-ray beam by using a video camera.

The crystal evaluation and data collection were performed on a Bruker D8 VENTURE diffractometer equipped with a Photon III detector with with Cu K_α (λ = 1.54178 Å) radiation and the diffractometer to crystal distance of 5.00 cm.[4]

The initial cell constants were obtained from one ϕ scan with short exposure times. The reflections were successfully indexed by an automated indexing routine built in the APEX5 program suite. The final cell constants were calculated from a set of 9753 strong reflections from the actual data collection.

The data were collected in a routine fashion to survey the reciprocal space to a resolution of 1 Å. A total of 205089 data were harvested by collecting 10 sets of frames with 1° scans in ω and ϕ with exposure times of 2–90 sec per frame. These highly redundant datasets were corrected for Lorentz and polarization effects. The absorption correction was based on fitting a function to the empirical transmission surface as sampled by multiple equivalent measurements.[5]

Structure Solution and Refinement

The systematic absences in the diffraction data were uniquely consistent for the space group $P2_12_12_1$ that yielded chemically reasonable and computationally stable results of refinement.[6-11]

A successful solution by the dual space recycling method provided most non-hydrogen atoms from the *E*-map. The remaining non-hydrogen atoms were located in an alternating series of least-squares cycles and difference Fourier maps. All non-hydrogen atoms were refined with anisotropic displacement coefficients unless specified otherwise. All hydrogen atoms were included in the structure factor calculation at idealized positions and were allowed to ride on the neighboring atoms with relative isotropic displacement coefficients, or refined with restraints and relative isotropic displacement coefficients.

The asymmetric unit content was assigned as 3 molecules of the foldamer and ½ of a water molecule: $(C_{87}H_{118}N_{14}O_{13})_3\cdot 1/2H_2O$. The foldamer molecules were refined with geometrical distance similarity restraints and constraints and atomic displacement parameter restraints and constraints. Many atoms exhibited large displacement parameters, especially in the 7-membered rings, but modeling the 7-membered rings as disordered was considered unproductive. The refinement of the four molecules is described below. The molecules were labelled in a consistent fashion offset by a 100 between the foldamers.

Foldamer C1. The *p*-cresolo group at C78 is disordered over two positions with the major component occupancy of 0.555(10). The benzene rings were refined with an idealized geometry.

Foldamer C101. The phenol ligand at C181 is disordered over two positions with the major component occupancy of 0.749(12). The benzene rings were refined with an idealized geometry.

Foldamer C201. The phenol ligand at C281 is disordered over two positions with the major component occupancy of 0.535(13). The benzene rings were refined with an idealized geometry. It was not possible to locate the 4-atom chain and diazepano group at atom C256. Attempts to conduct a refinement with a DFT-optimized rigid diazepano group were computationally unstable. The chain is only partially modelled with atoms for which peaks were present in the difference Fourier map as follows. Atom C257 – fully occupied, anisotropic. Atom C258/C58C indicate a disorder of the chain in a 0.555(10):0.445(10) ratio. Atoms C259, O208, N209, N210, and C264 were refined with a 0.445(10) occupancy. These disordered atoms were refined isotropically. This fragment was refined freely and its geometry is tentative.

Water molecule. An isolated peak of electron density located among the foldamers was assigned to be a half-occupied water molecule. No attempt to locate H atoms on it was made.

The overall formula of the compound reflects the composition $(C_{87}H_{118}N_{14}O_{13})_3\cdot 1/2H_2O$, whereas some atoms were not located and refined (the missing atoms in foldamer C201 and water molecule).

The absolute structure was established by resonant scattering effects. During the final stages of the refinement the outlier reflections were omitted and the Flack x parameter increased in value. The absolute structure assignment is consistent with the synthesis – all chiral centers are *S*.

The placement of the H atoms was optimized to form as many hydrogen-bonding interactions as possible. The final least-squares refinement of 3053 parameters against 27098 data with 13467 restraints resulted in residuals R (based on F^2 for $I \geq 2\sigma$) and wR (based on F^2 for all data) of 0.1221 and 0.3456, respectively. The final difference Fourier map was mostly featureless.

Summary

Crystal Data for $C_{87}H_{118.33}N_{14}O_{13.16}$ (M =1570.95 g/mol): orthorhombic, space group $P2_12_12_1$ (no. 19), a = 24.9460(6) Å, b = 28.2854(6) Å, c = 36.9128(9) Å, V = 26046.0(10) Å^3, Z = 12, T = 100.00 K, μ(Cu Kα) = 0.660 mm^{-1}, $Dcalc$ = 1.202 g/cm^3, 205089 reflections measured (3.936° ≤ 2Θ ≤ 100.86°), 27098 unique (R_{int} = 0.0655, R_{sigma} = 0.0455) which were used in all calculations. The final R_1 was 0.1221 (I > 2σ(I)) and wR_2 was 0.3465 (all data).

Table S15. Overview of crystallography-related parameters for foldamer **12**.

Deposition number	2466657
Empirical formula	$C_{87}H_{118}N_{14}O_{13}$·1/6 H_2O
Formula weight	1570.95
Temperature/K	100.00
Crystal system	orthorhombic
Space group	$P2_12_12_1$
a/Å	24.9460(6)
b/Å	28.2854(6)
c/Å	36.9128(9)
α/°	90
β/°	90
γ/°	90
Volume/Å^3	26046.0(10)
Z	12
ρ_{calc}g/cm^3	1.202
μ/mm^{-1}	0.660
F(000)	10124.0
Crystal size/mm^3	0.1 × 0.05 × 0.05
Radiation	Cu Kα (λ = 1.54178)
2Θ range for data collection/°	3.936 to 100.86
Index ranges	-24 ≤ h ≤ 24, -16 ≤ k ≤ 28, -33 ≤ l ≤ 36
Reflections collected	205089
Independent reflections	27098 [R_{int} = 0.0655, R_{sigma} = 0.0455]
Data/restraints/parameters	27098/13467/3053
Goodness-of-fit on F^2	1.361
Final R indexes [I>=2σ (I)]	R_1 = 0.1221, wR_2 = 0.3116
Final R indexes [all data]	R_1 = 0.1602, wR_2 = 0.3465
Largest diff. peak/hole / e Å^{-3}	0.71/-0.55
Flack parameter	0.16(5)

5.9 Crystal Structure-Derived Geometric Parameters for **5**

Five separate symmetry independent structures were observed in the asymmetric unit of foldamer **5**. Some structures displayed multiple distinct sidechain and peptide backbone conformations. Atom labels listed in the figures and tables presented below correspond with atom labels in the structural data for foldamer **5**.

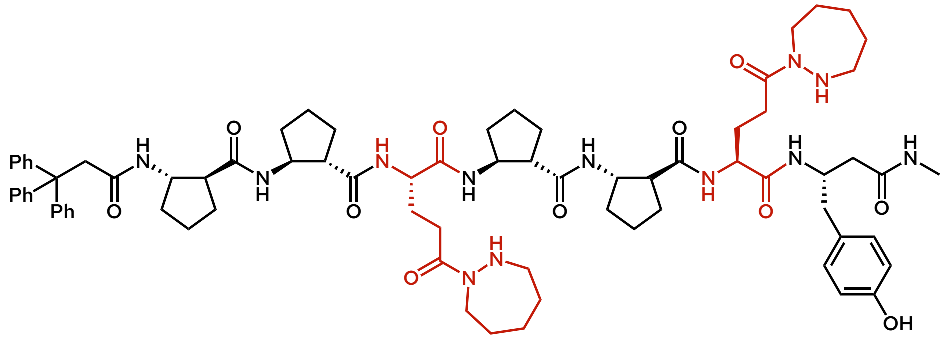

TPP-ACPC-ACPC-Glu(Hy)-ACPC-ACPC-Glu(Hy)-B^{3}HTyr-C(O)NHMe (5)

Table S16. Through space Cα-to-Cα distances for foldamer **5**

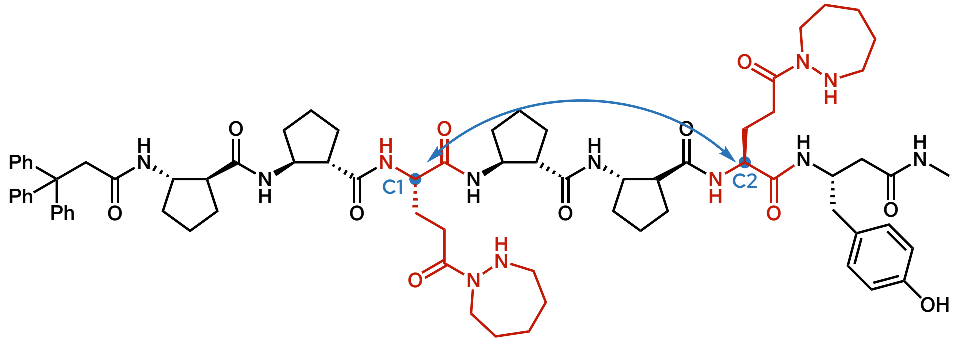

Atom Labels	Cα-to-Cα Distance (Å)
C34 - C56	5.776(9)
C34A - C56A	5.884(12)
C34A - C56E	5.88(3)
C34B - C56B	6.193(9)
C34C - C56C	5.916(10)
C34D - C56D	6.152(11)

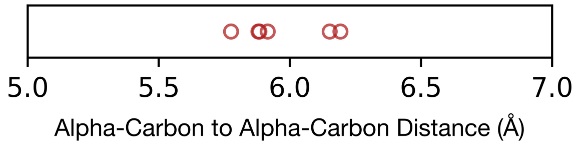

Figure S45. Plot of Cα-to-Cα distances for foldamer **5**

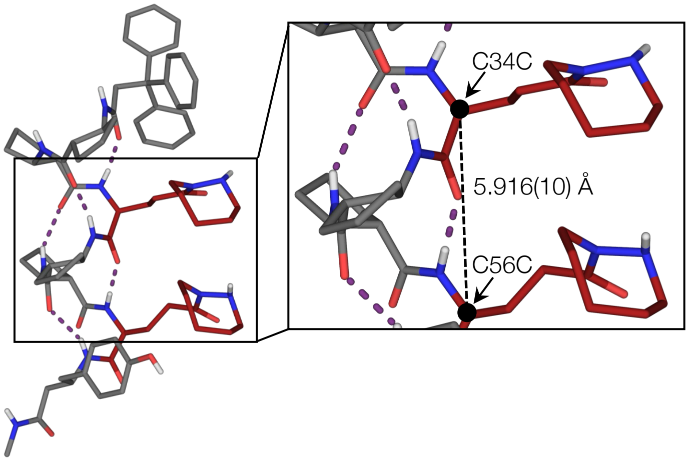

Figure S46. Depiction of Cα-to-Cα distance for foldamer labeled **5C**.

Table S17. Through space C=O-to-C=O distances for foldamer **5**

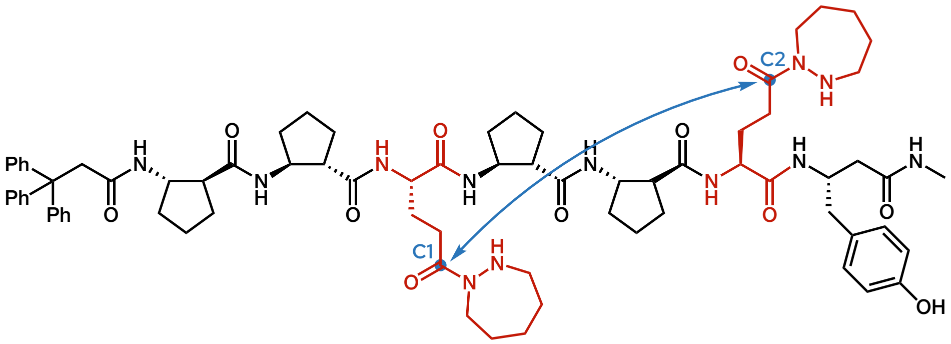

Atom Labels	C=O-to-C=O Distance (Å)
C37 - C59	5.721(12)
C37A - C59A	6.037(19)
C37A - C59E	5.97(3)
C37B - C59B	5.898(16)
C37B - C59F	6.30(2)
C37C - C59C	5.74(2)
C37D - C59D	7.840(16)

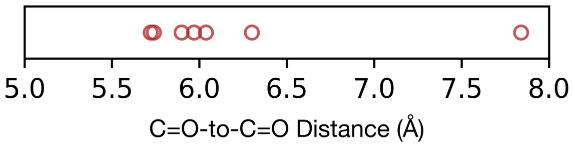

Figure S47. Plot of C=O-to-C=O distances for foldamer **5**

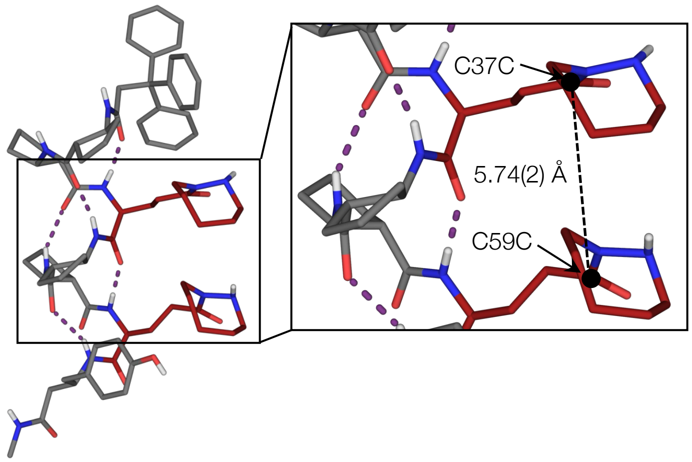

Figure S48. Depiction of C=O-to-C=O distance for foldamer labeled **5C**.

Table S18. Through space Glu(Hy)-Glu(Hy) sidechain dihedral angles for foldamer **5**

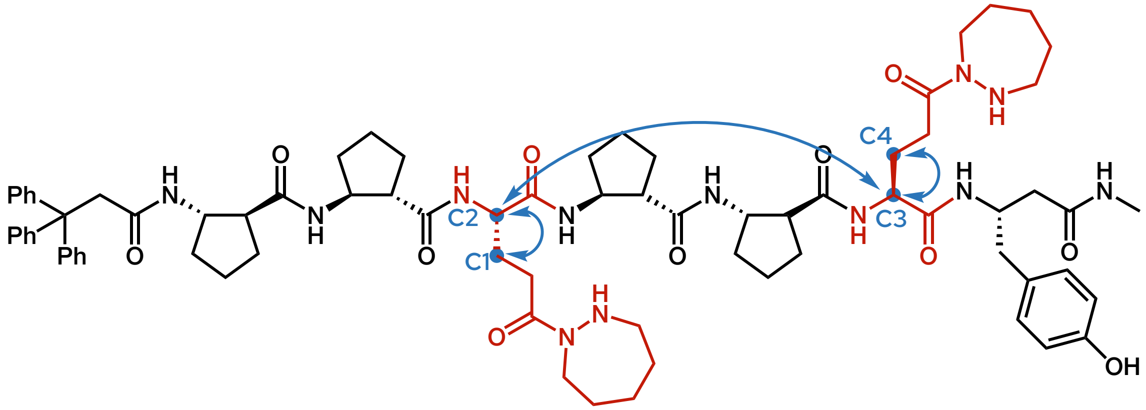

Atom Labels	Glu(Hy)-Glu(Hy) Dihedral Angle (°)
C35 – C34 – C56 – C57	24.5(6)
C35A – C34A – C56A – C57A	-7.7(9)
C35A – C34A – C56E – C57E	20.5(15)
C35B – C34B – C56B – C57B	47.3(8)
C35B – C34B – C56B – C57F	51.6(10)
C35C – C34C – C56C – C57C	31.8(8)
C35C – C34C – C56C – C57G	-9.7(9)
C35D – C34D – C56D – C57D	26.8(6)

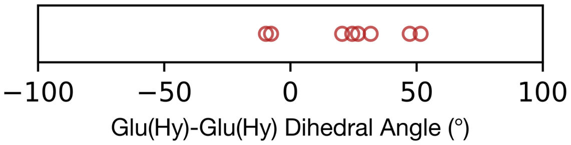

Figure S49. Plot of Glu(Hy)-Glu(Hy) sidechain dihedral angles for foldamer **5**

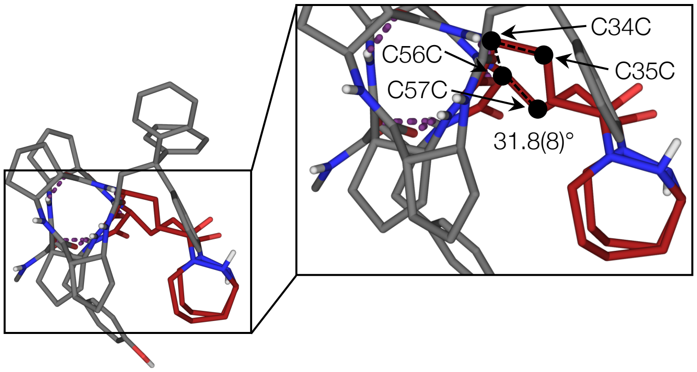

Figure S50. Depiction of Glu(Hy)-Glu(Hy) sidechain dihedral angle for foldamer labeled **5C**.

Table S19. Pairwise RMSD values for symmetry independent peptide backbones for foldamer **5**.

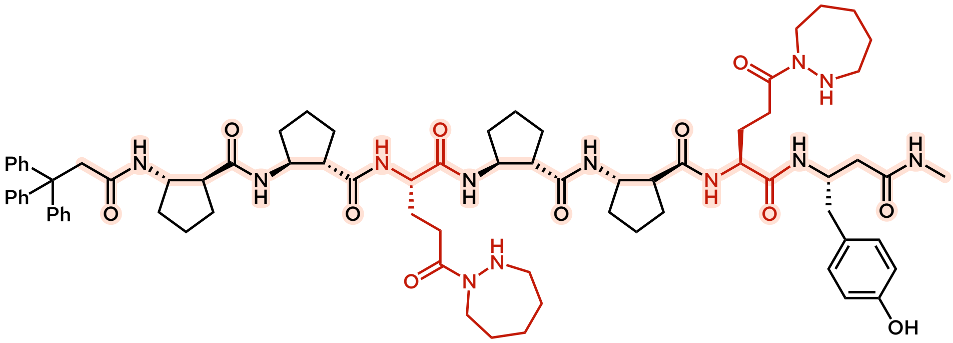

Backbones Compared	RMSD (Å)
No Suffix to A	0.180
No Suffix to B	0.462
No Suffix to C	1.093
No Suffix to D	0.299
A to B	0.546
A to C	1.095
A to D	0.352
B to C	1.096
B to D	0.358
C to D	1.019

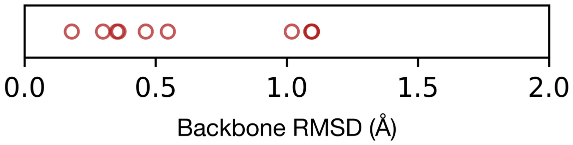

Figure S51. Plot of pairwise RMSD values for symmetry independent peptide backbones for foldamer **5**.

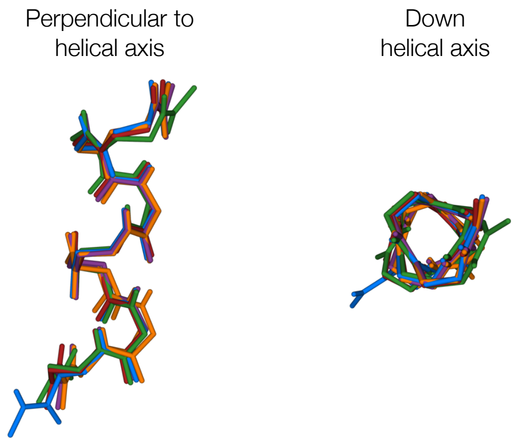

Figure S52. Superimposition of all symmetry independent peptide backbones of foldamer **5**.

5.10 Crystal Structure-Derived Geometric Parameters for **11**

One symmetry independent structure was observed in the asymmetric unit of foldamer **11**. Atom labels listed in the tables presented below correspond with atom labels in the structural data for foldamer **11**.

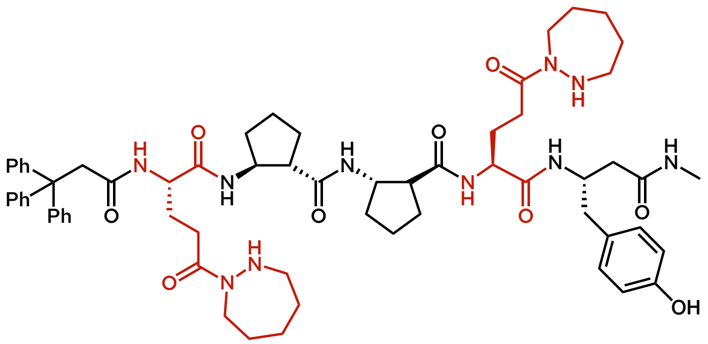

TPP-Glu(Hy)-ACPC-ACPC-Glu(Hy)-B³HTyr-C(O)NHMe (11)

Table S20. Through space Cα-to-Cα distances for foldamer **11**.

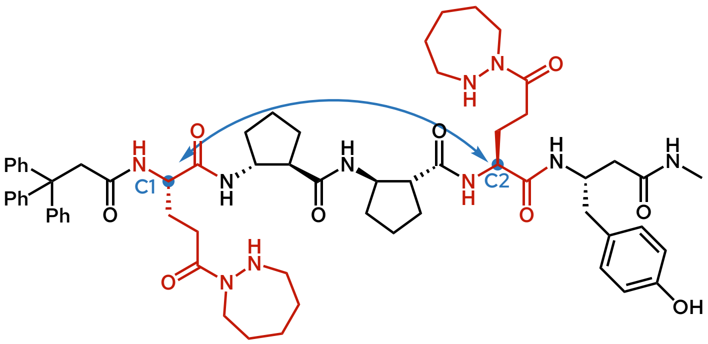

Atom Labels	Cα-to-Cα Distance (Å)
C6 – C20	5.684(19)

Table S21. Through space C=O-to-C=O distances for foldamer **11**.

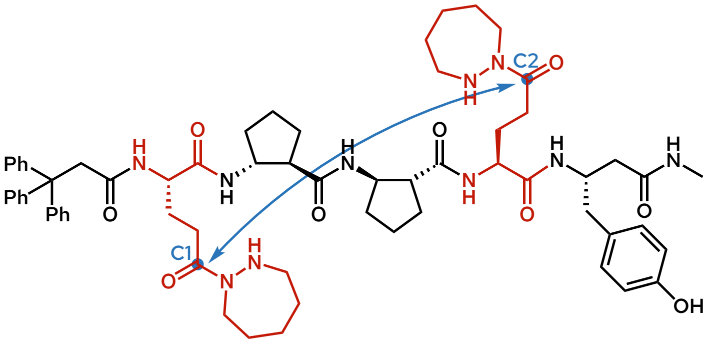

Atom Labels	C=O-to-C=O Distance (Å)
C51 – C59	6.498(2)

Table S22. Through space Glu(Hy)-Glu(Hy) sidechain dihedral angles for foldamer **11**.

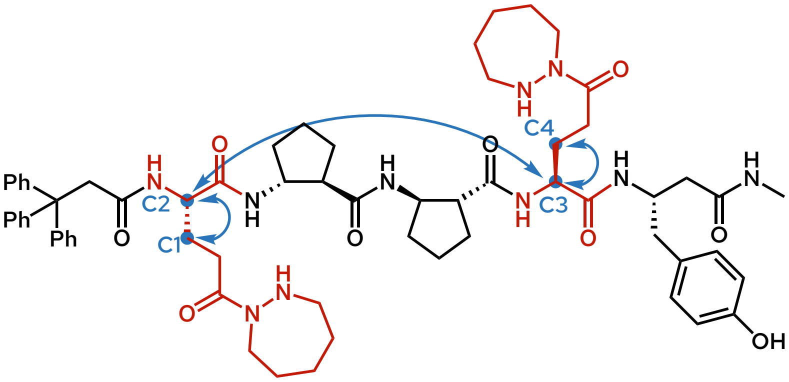

Atom Labels	Glu(Hy)-Glu(Hy) Dihedral Angle (°)
C57-C20-C6-C49	97.7(12)

5.11 Crystal Structure-Derived Geometric Parameters for **12**

Three separate symmetry independent structures were observed in the asymmetric unit of foldamer **12**. For one structure of **12**, a Glu(Hy) sidechain was not fully modeled due to significant disorder. Atom labels listed in the figures and tables presented below correspond with atom labels in the structural report for foldamer **12**.

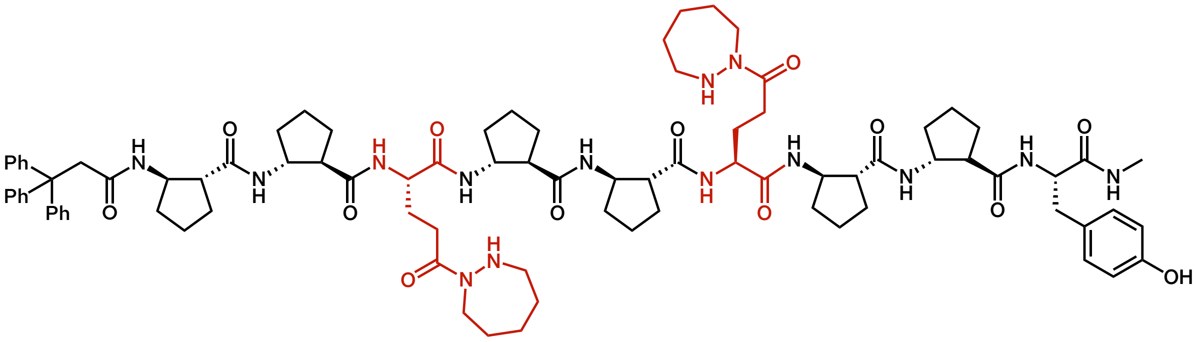

TPP-ACPC-ACPC-Glu(Hy)-ACPC-ACPC-Glu(Hy)-ACPC-ACPC-Tyr-C(O)NHMe (12)

Table S23. Through space Cα-to-Cα distances for foldamer **12**.

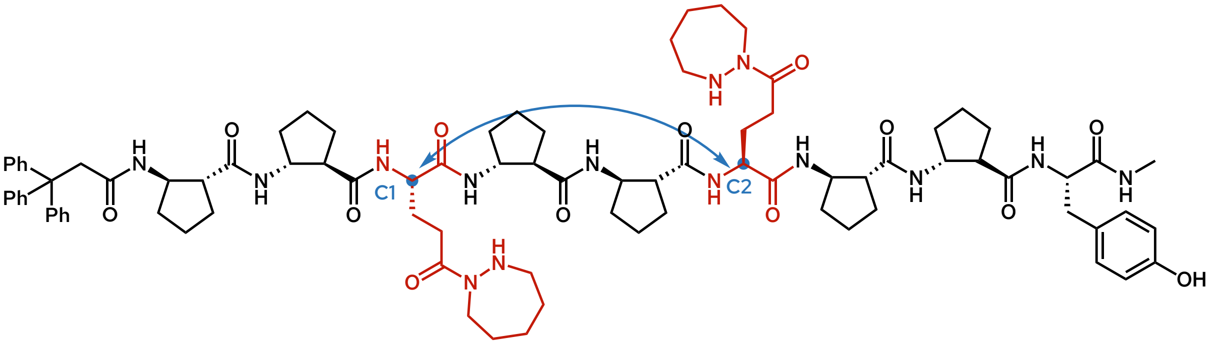

Atom Labels	Cα-to-Cα Distance (Å)
C34 - C56	6.01(2)
C134 - C156	5.32(2)
C234 - C256	5.766(18)

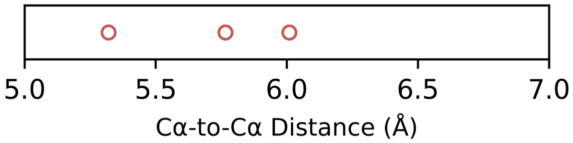

Figure S53. Plot of Cα-to-Cα distances for foldamer **12**.

Table S24. Through space C=O-to-C=O distances for foldamer **12**.

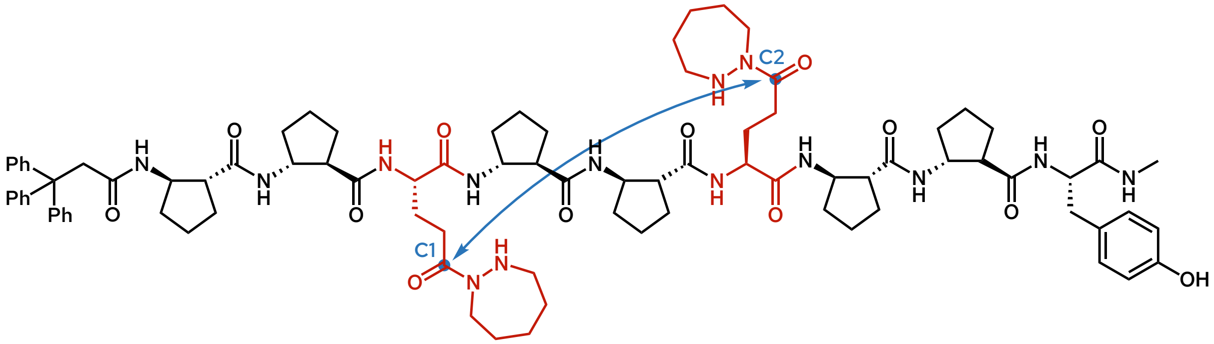

Atom Labels	C=O-to-C=O Distance (Å)
C37 - C59	5.19(3)
C137 - C159	5.53(3)
C237 - C259	7.33(6)

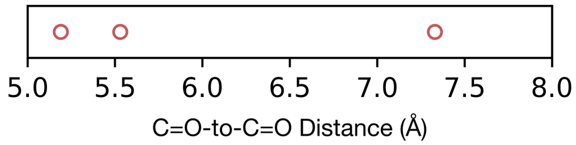

Figure S54. Plot of C=O-to-C=O distances for foldamer **12**.

Table S25. Through space Glu(Hy)-Glu(Hy) sidechain dihedral angles for foldamer **12**.

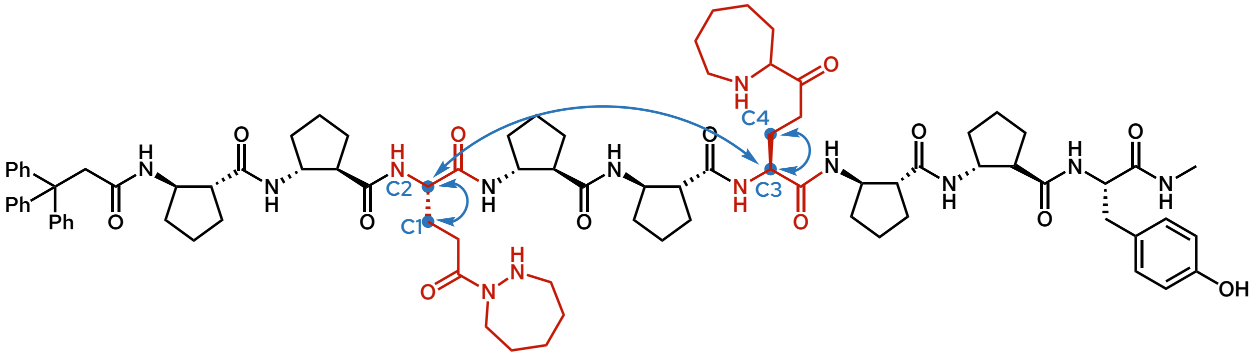

Atom Labels	Glu(Hy)-Glu(Hy) Dihedral Angle (°)
C35 – C34 – C56 – C57	-54.0(17)
C135 – C134 – C156 – C157	-46.6(13)
C235 – C234 – C256 – C257	35.6(13)

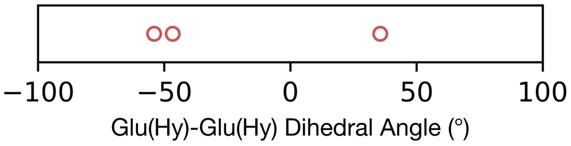

Figure S55. Plot of Glu(Hy)-Glu(Hy) sidechain dihedral angles for foldamer **12**.

Table S26. Pairwise RMSD values for symmetry independent peptide backbones for foldamer **12**.

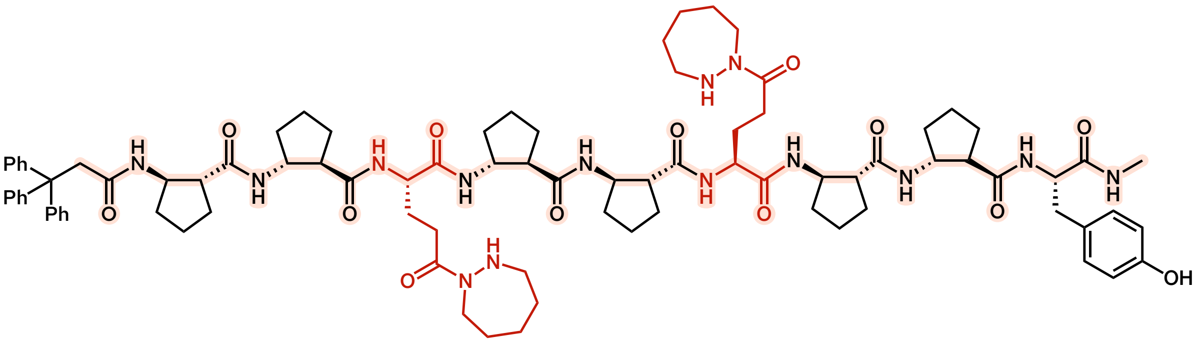

Backbones Compared	RMSD (Å)
No Suffix to 1	0.925
No Suffix to 2	1.284
1 to 2	1.711

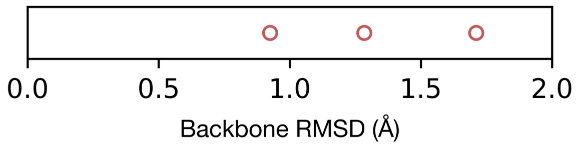

Figure S56. Plot of pairwise RMSD values for symmetry independent peptide backbones for foldamer **12.**

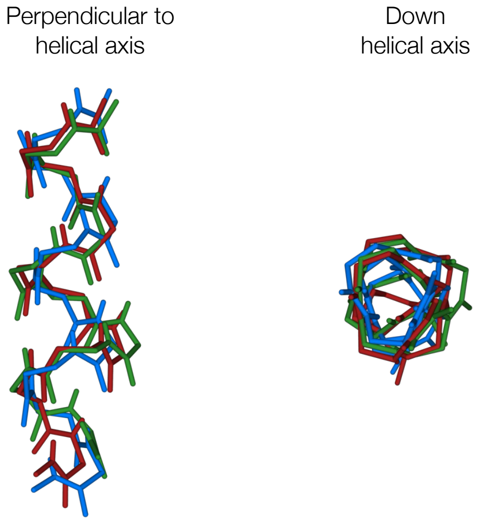

Figure S57. Superimposition of all symmetry independent peptide backbones of foldamer **12**.

5.12 Comparison of Geometric Parameters for **5**, **11**, and **12**

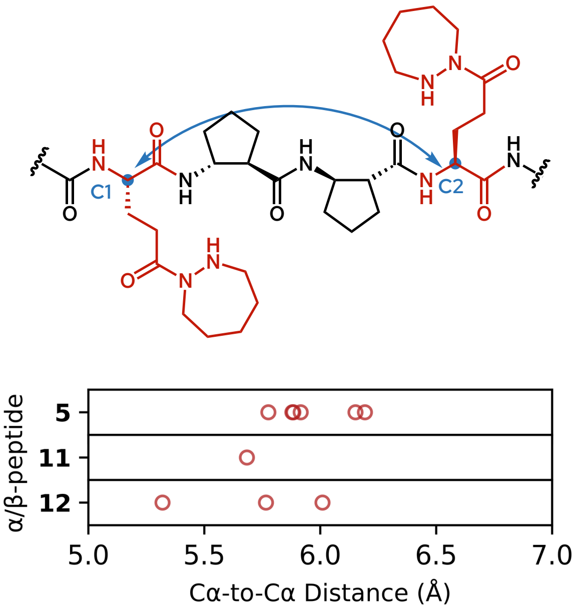

Figure S58. Comparison of Cα-to-Cα distances for foldamers **5**, **11** and **12**.

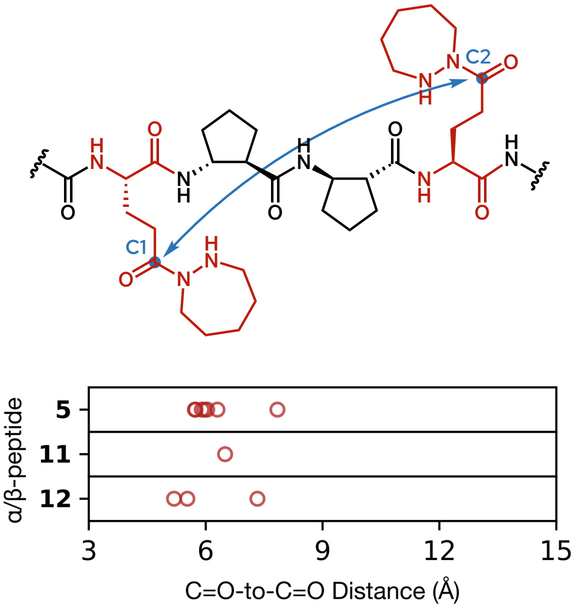

Figure S59. Comparison of C=O-to-C=O distances for foldamers **5**, **11** and **12**.

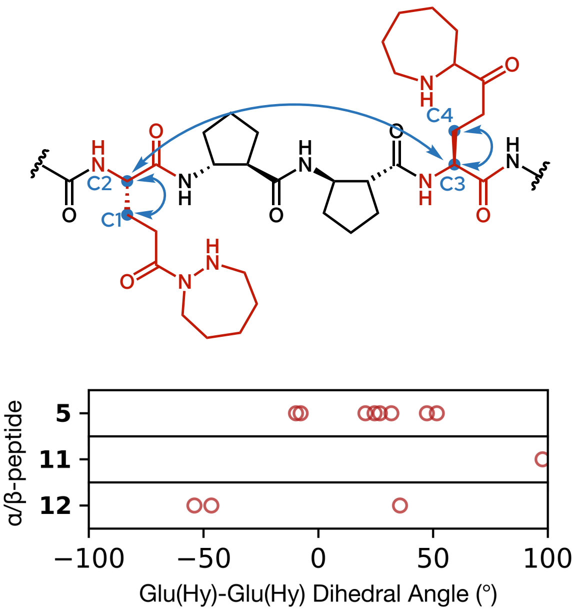

Figure S60. Comparison of Glu(Hy)-Glu(Hy) sidechain dihedral angles for foldamers **5**, **11** and **12**.

6. Computational Information

Initial conformer geometries were generated using RDKit.[16] All DFT calculations were carried out using Gaussian 16.[17] Geometry optimizations and vibrational frequency calculations were performed at the B3LYP/6-31G(d,p)/(CPCM=MeCN) and M06-2X/def2-TZVP/(CPCM=MeCN) levels.[18-22] Conformer energies obtained using the M06-2X method and a triplet zeta basis set are considered accurate for organic small-molecule species such as the dihydrazide studied here.[23] All reported optimized geometries possessed no negative vibrational modes. Structures were visualized using CYLview20.[24] Python code used to generate conformers is available on GitHub. Energies, free energies, geometric parameters and XYZ coordinates for all optimized species at both levels of theory are provided in a separate spreadsheet supplied in the GitHub repository for this work.

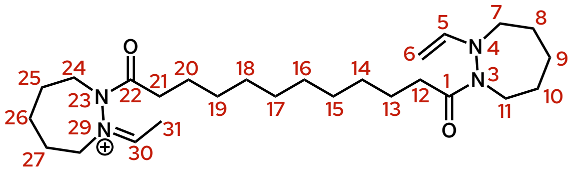

Figure S61. Atom numbering scheme for **13'**.

6.1 DFT-derived Geometric Parameters for **13'** – B3YLP/6-31G(d,p)

Using RDKit, 150,000 conformers (75,000 per iminium configurational isomer) of **13'** with acetaldehyde-derived iminium and enamine moieties on the scaffold were generated. To ensure the full conformation space of **13'** was sampled, additional conformers of **13'** with linear polymethylene tethers were generated. From this set, the relative free energies (ΔG) of ~2,500 conformers (~1,250 per iminium configurational isomer) representative of the conformational space accessible to **13'** were evaluated at the B3YLP/6-31G(d,p)/(CPCM=MeCN) level. An energetic preference for conformations with extended polymethylene tethers (all tether dihedrals in the *anti* configuration) was observed. This suggests close organization of iminium and enamine moieties on **13'** is not energetically favorable. This observation corresponds with previous high level computational investigations of long n-alkane conformational preferences.[25-27] Conformers of **13'** with the iminium in the *E* configuration were generally lower in relative free energy than similar conformers of **13'** with the iminium in the Z configuration. This observation agreed with the results of a previous computational investigation of 7-membered hydrazide configurational preferences.[1]

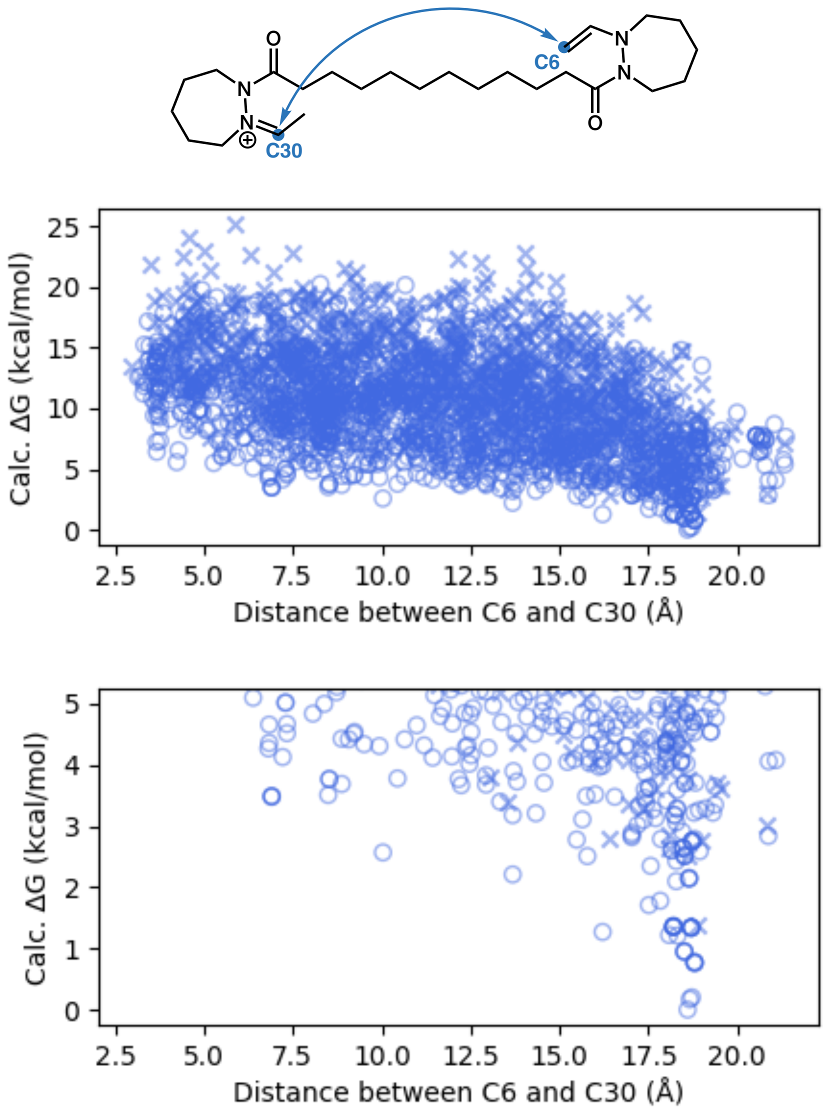

Figure S62. Distribution of the distance between C6 and C30 for conformers of **13'** optimized at the B3LYP/6-31G(d,p)/(CPCM=MeCN) level of theory. O = species with iminium in E configuration, X = species with iminium in Z configuration.

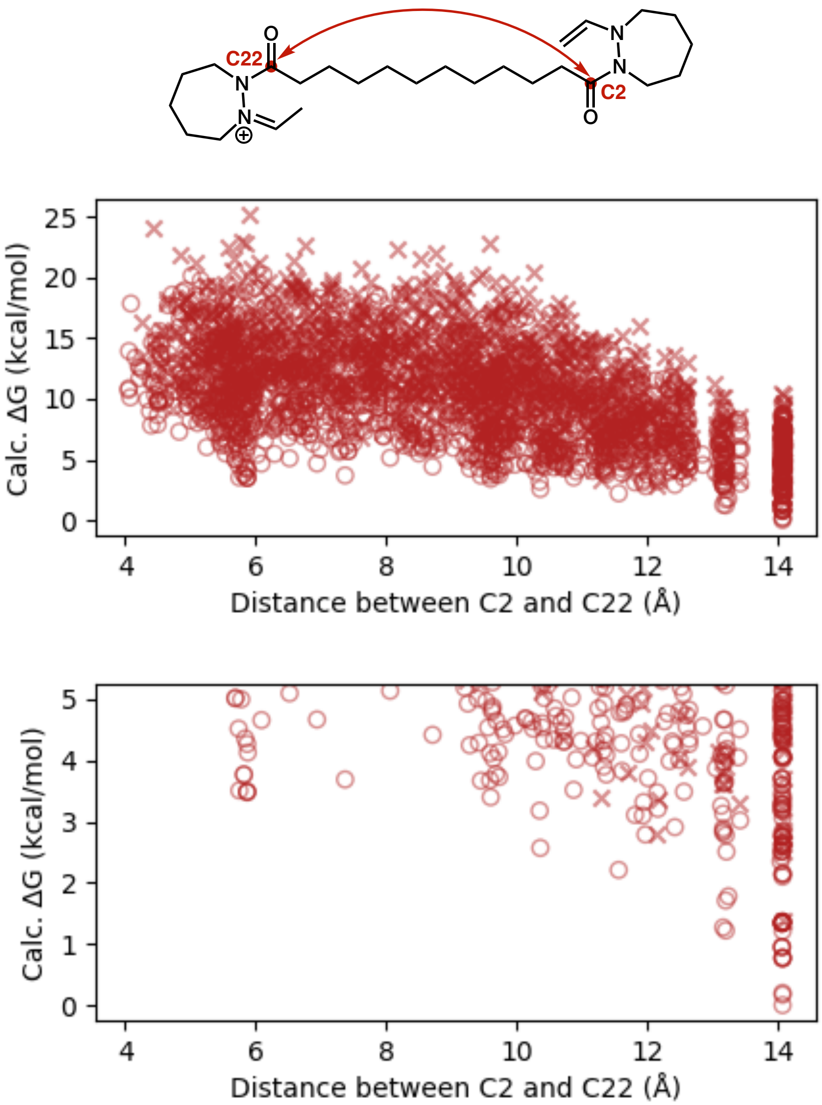

Figure S63. Distribution of the distance between C2 and C22 for conformers of **13'** optimized at the B3LYP/6-31G(d,p)/(CPCM=MeCN) level of theory. O = species with iminium in E configuration, X = species with iminium in Z configuration.

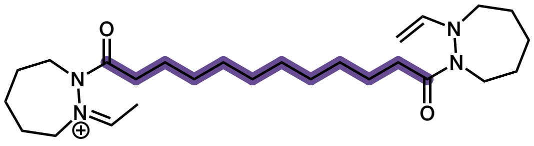

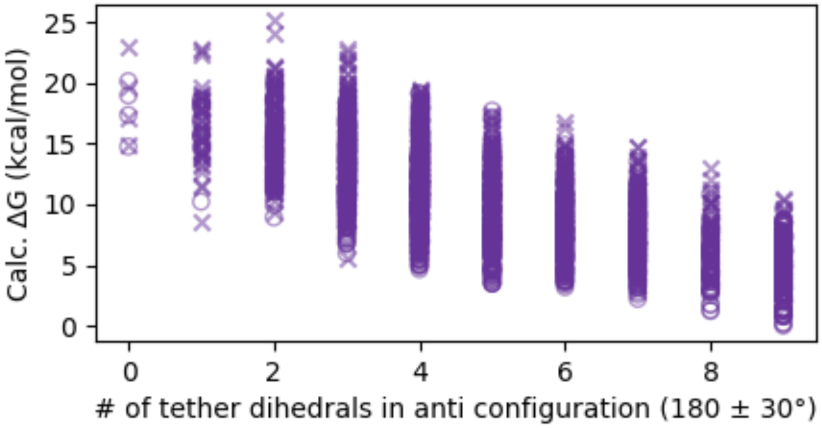

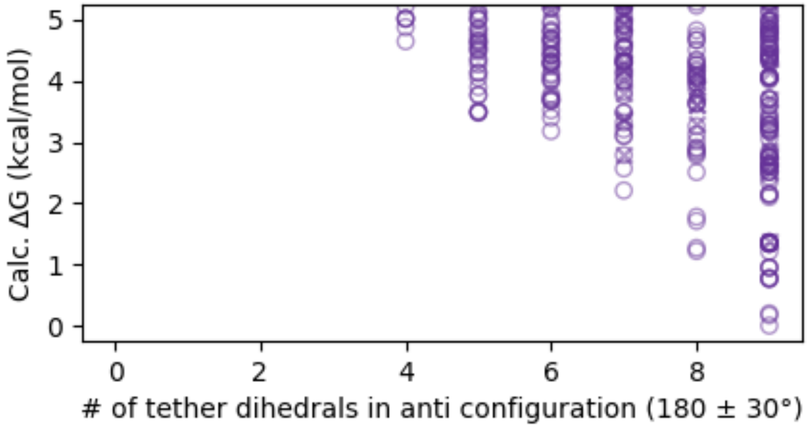

Figure S64. Distribution of the number of anti dihedral angles in the polymethylene tether for conformers of **13'** optimized at the B3LYP/6-31G(d,p)/(CPCM=MeCN) level of theory. O = species with iminium in E configuration, X = species with iminium in Z configuration.

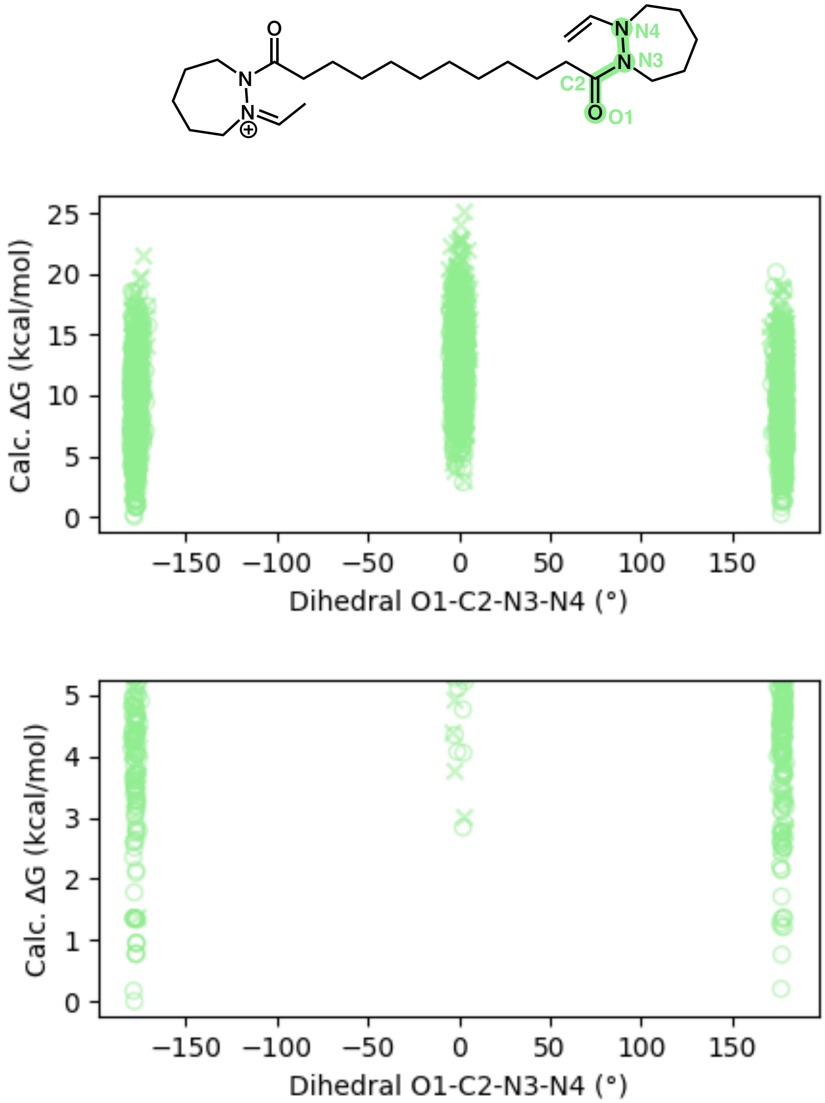

Figure S65. Distribution of O1-C2-N3-N4 dihedral angles for conformers of **13’** optimized at the B3LYP/6-31G(d,p)/(CPCM=MeCN) level of theory. O = species with iminium in E configuration, X = species with iminium in Z configuration.

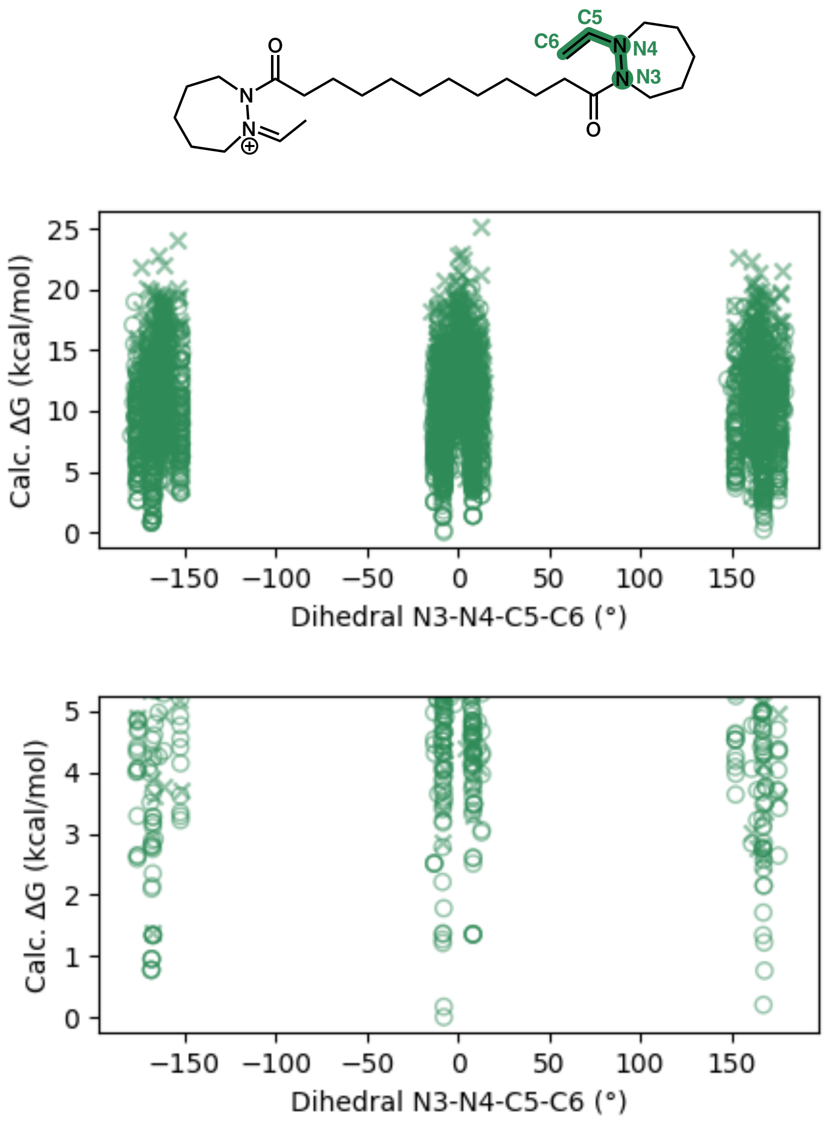

Figure S66. Distribution of N3-N4-C5-C6 dihedral angles for conformers of **13’** optimized at the B3LYP/6-31G(d,p)/(CPCM=MeCN) level of theory. O = species with iminium in E configuration, X = species with iminium in Z configuration.

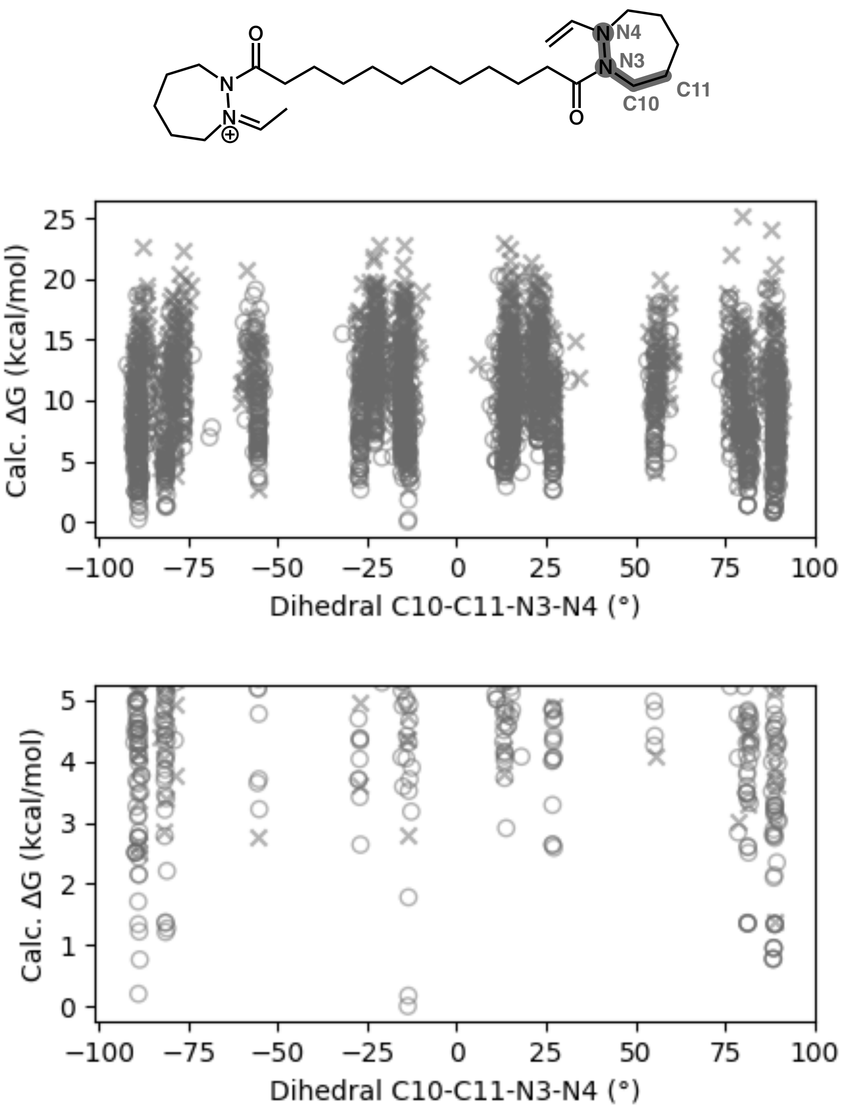

Figure S67. Distribution of C10-C11-N3-N4 dihedral angles for conformers of **13'** optimized at the B3LYP/6-31G(d,p)/(CPCM=MeCN) level of theory. O = species with iminium in E configuration, X = species with iminium in Z configuration.

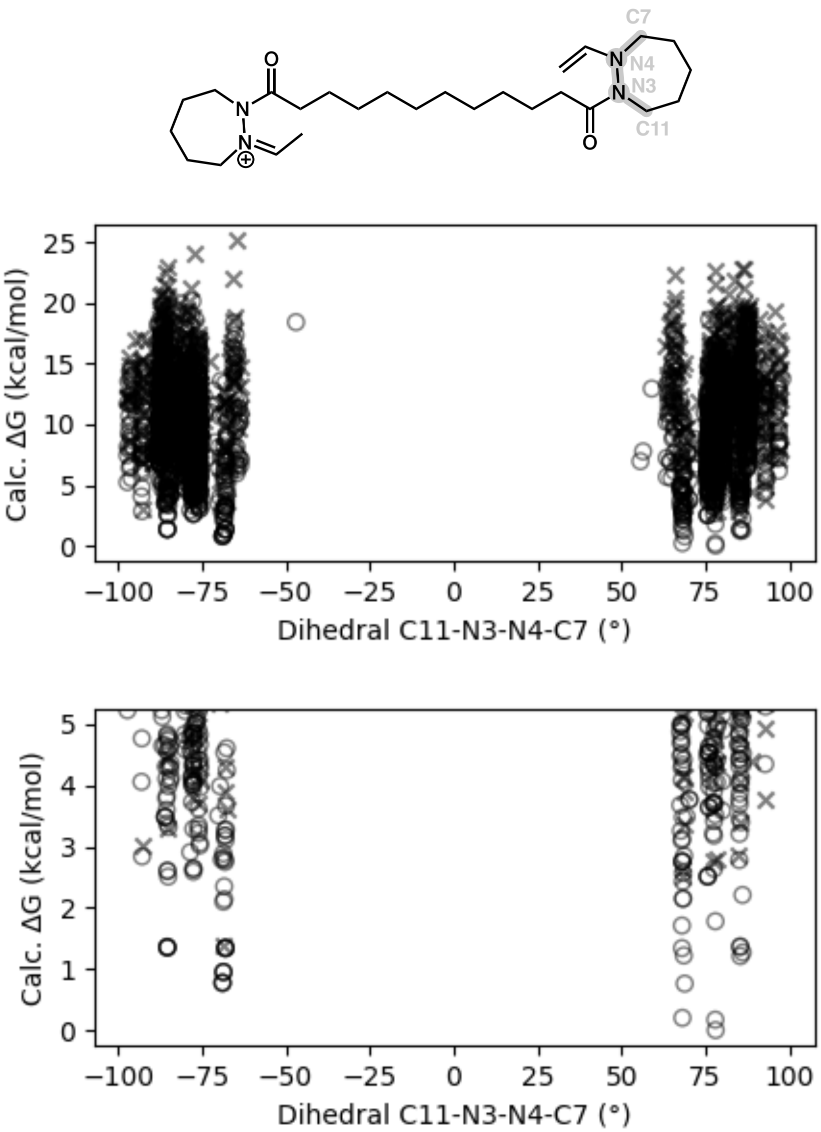

Figure S68. Distribution of C11-N3-N4-C7 dihedral angles for conformers of **13’** optimized at the B3LYP/6-31G(d,p)/(CPCM=MeCN) level of theory. O = species with iminium in E configuration, X = species with iminium in Z configuration.

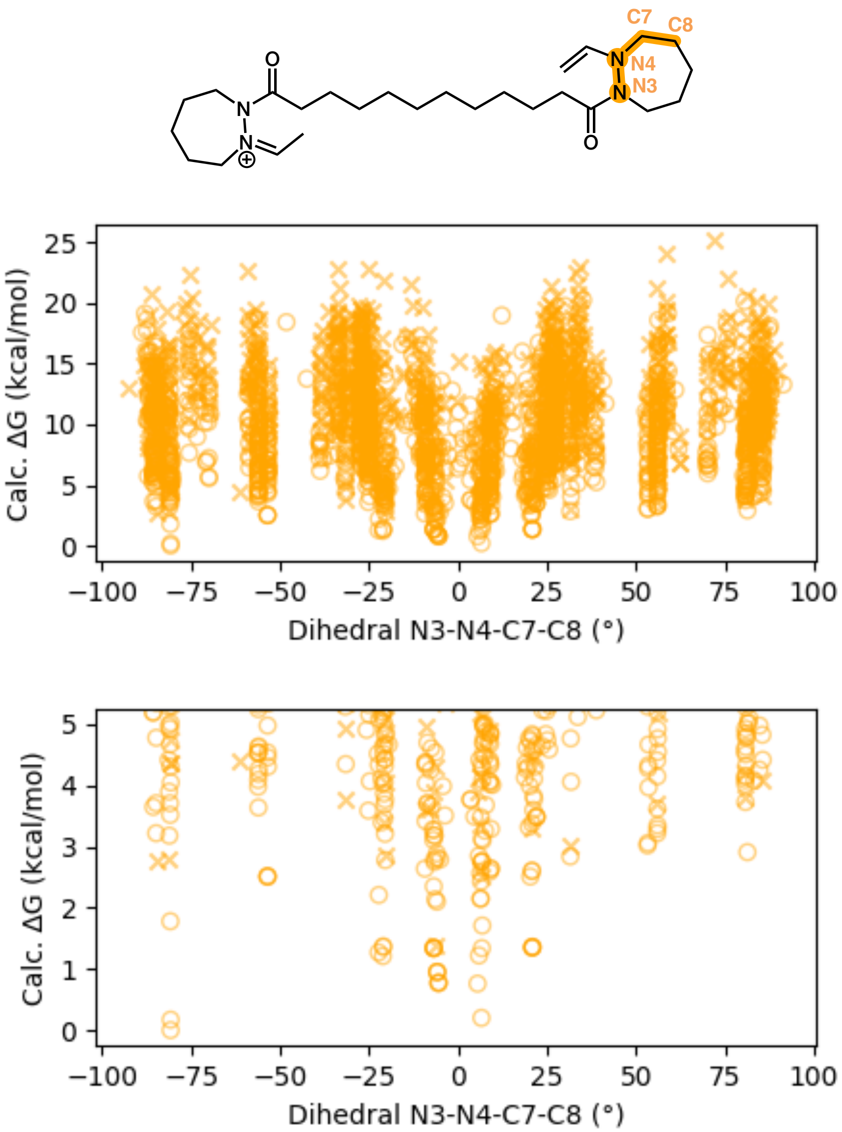

Figure S69. Distribution of N3-N4-C7-C8 dihedral angles for conformers of **13'** optimized at the B3LYP/6-31G(d,p)/(CPCM=MeCN) level of theory. O = species with iminium in E configuration, X = species with iminium in Z configuration.

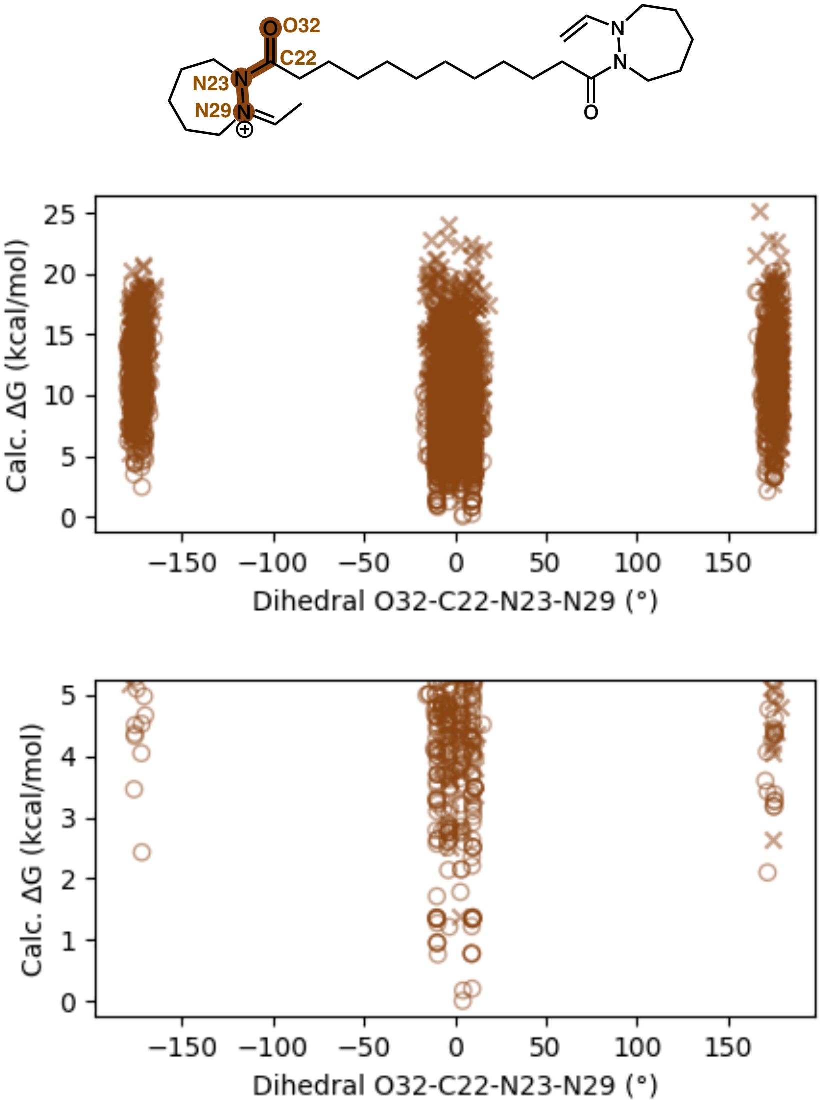

Figure S70. Distribution of O32-C22-N23-N29 dihedral angles for conformers of **13'** optimized at the B3LYP/6-31G(d,p)/(CPCM=MeCN) level of theory. O = species with iminium in E configuration, X = species with iminium in Z configuration.

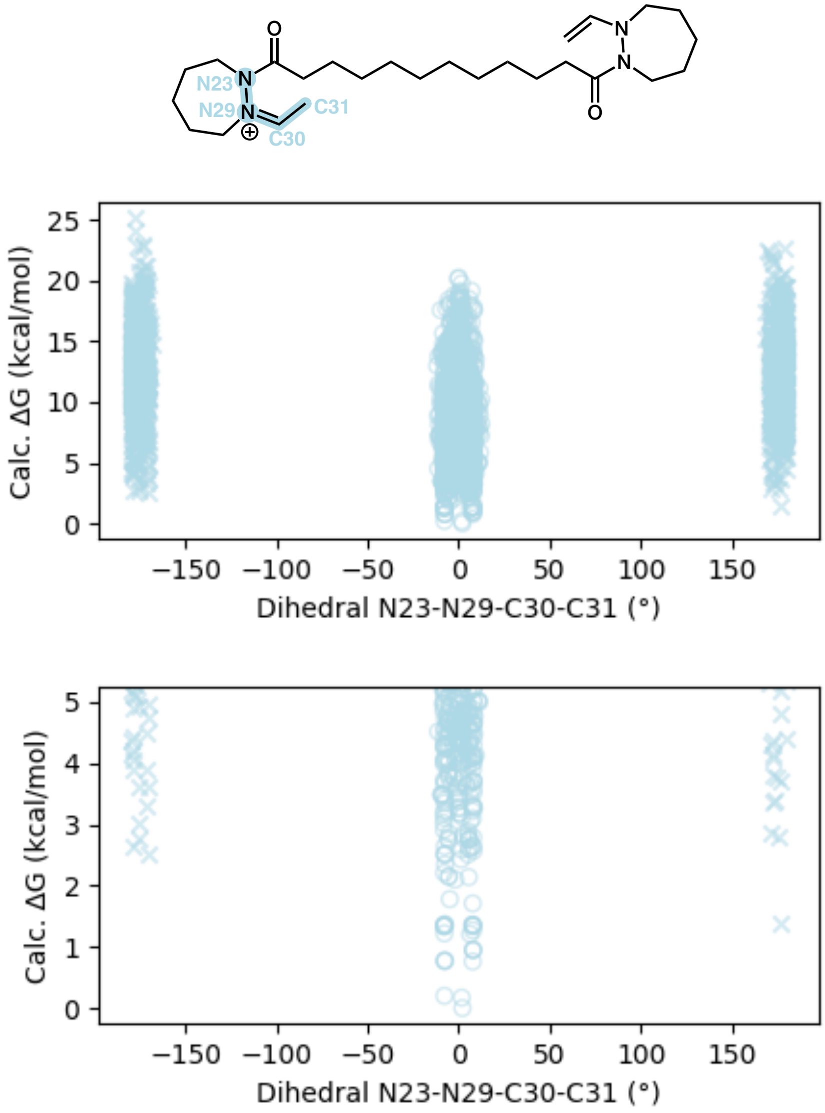

Figure S71. Distribution of N23-N29-C30-C31 dihedral angles for conformers of **13'** optimized at the B3LYP/6-31G(d,p)/(CPCM=MeCN) level of theory. O = species with iminium in E configuration, X = species with iminium in Z configuration.

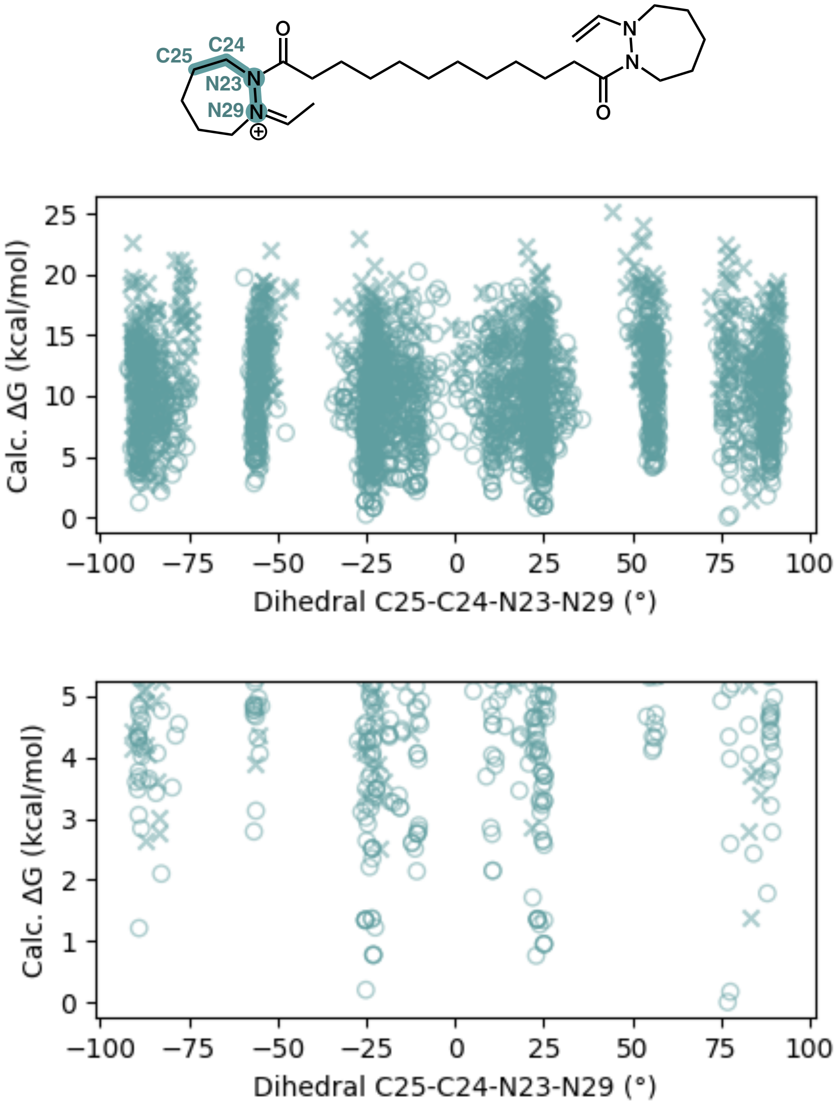

Figure S72. Distribution of C25-C24-N23-N29 dihedral angles for conformers of **13’** optimized at the B3LYP/6-31G(d,p)/(CPCM=MeCN) level of theory. O = species with iminium in E configuration, X = species with iminium in Z configuration.

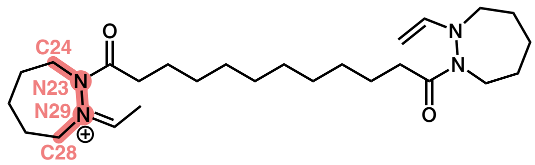

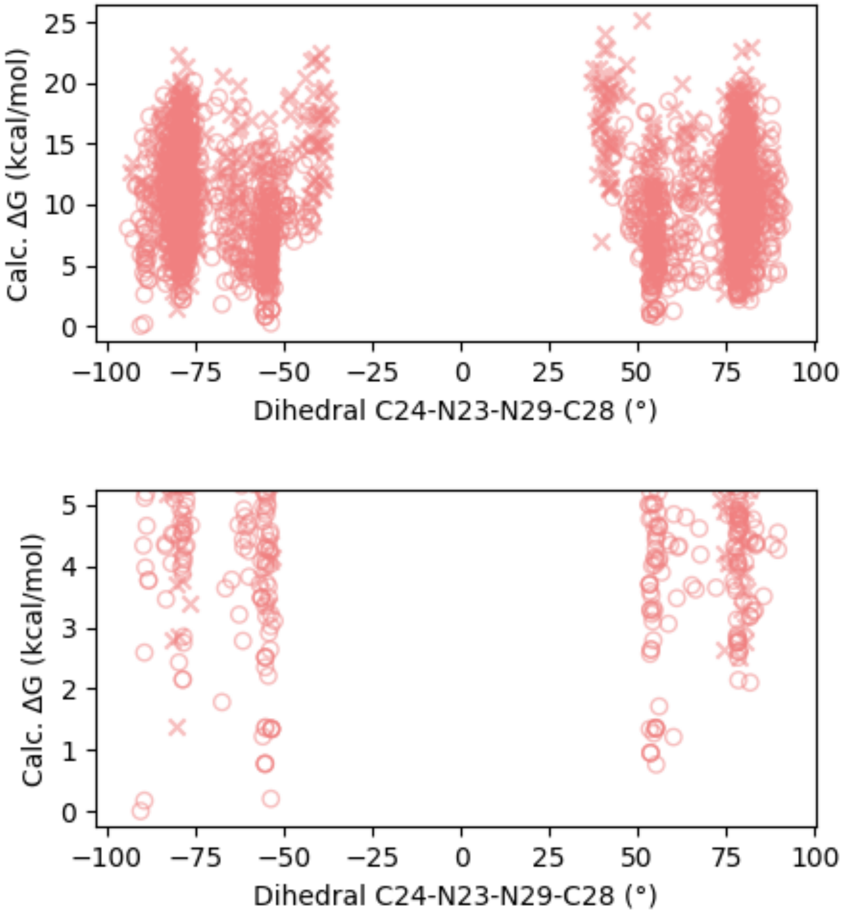

Figure S73. Distribution of C24-N23-N29-C28 dihedral angles for conformers of **13'** optimized at the B3LYP/6-31G(d,p)/(CPCM=MeCN) level of theory. O = species with iminium in E configuration, X = species with iminium in Z configuration.

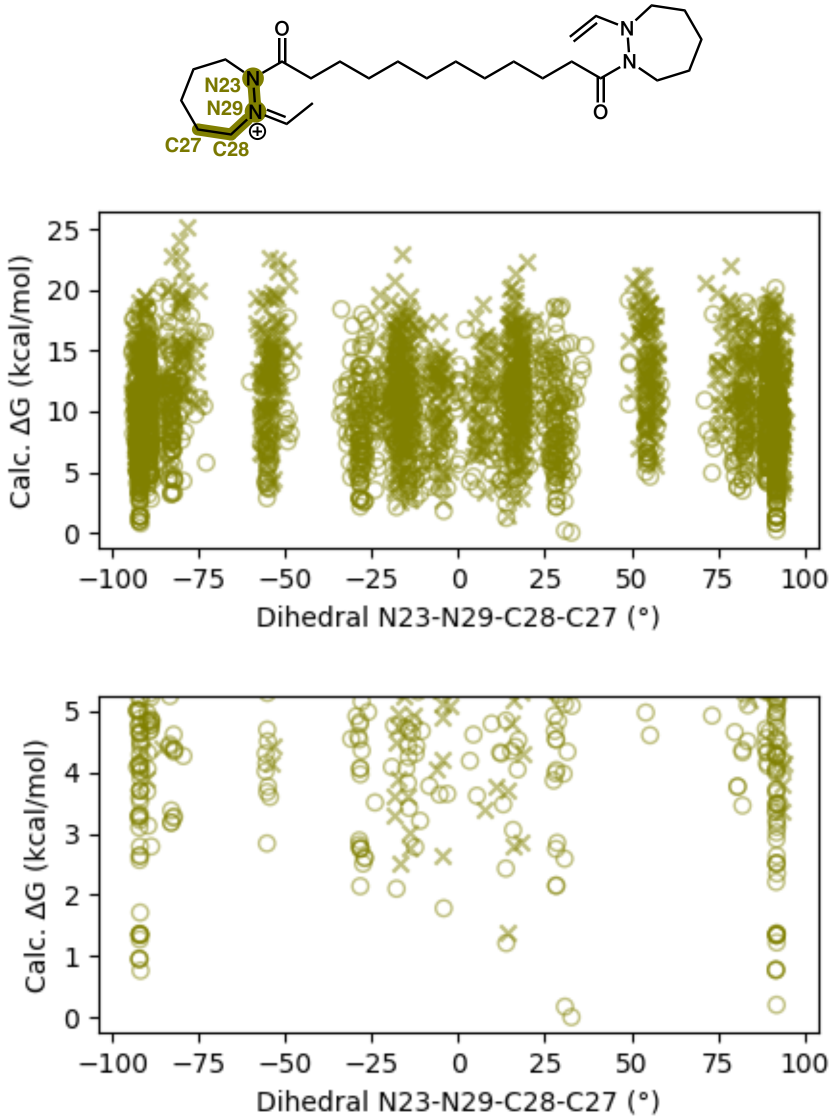

Figure S74. Distribution of N23-N29-C28-C27 dihedral angles for conformers of **13'** optimized at the B3LYP/6-31G(d,p)/(CPCM=MeCN) level of theory. O = species with iminium in E configuration, X = species with iminium in Z configuration.

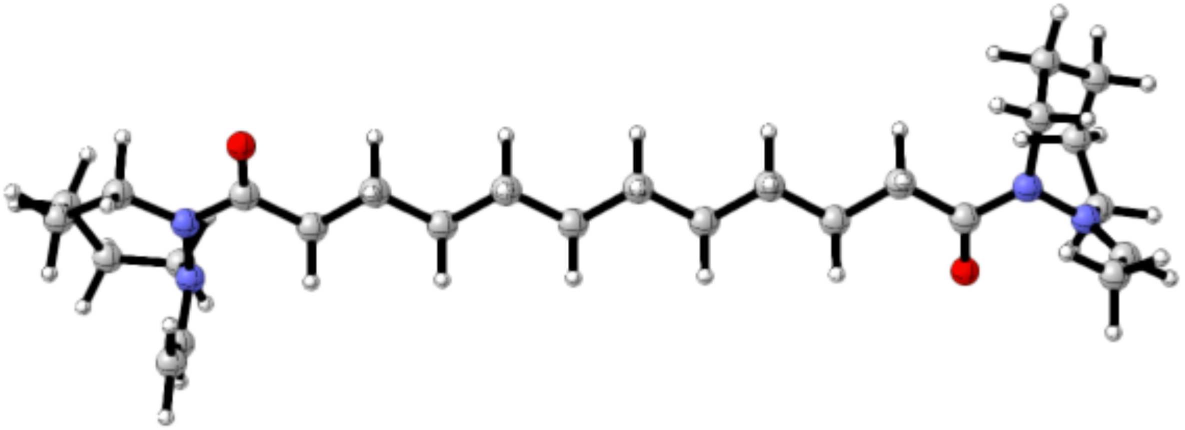

Figure S75. Lowest free energy conformer for **13’** identified at the B3LYP/6-31G(d,p)/(CPCM=MeCN) level of theory.

6.2 DFT-derived Geometric Parameters for **13'** – M06-2X/def2-TZVP

~750 conformers of **13'** with the iminium in the ***E*** configuration previously evaluated at the B3LYP/6-31G(d,p)/(CPCM=MeCN) level of theory were fully reevaluated (geometry reoptimized and vibrational frequencies calculated) at the M06-2X/def2-TZVP/(CPCM=MecN) level. Again, an energetic preference towards extended polymethylene tether conformations was observed, suggesting close preorganization of iminium and enamine moieties on **13'** is not energetically favorable.

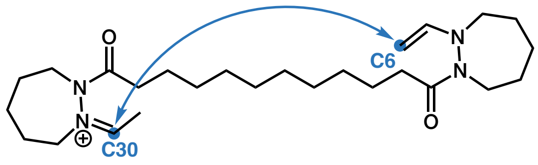

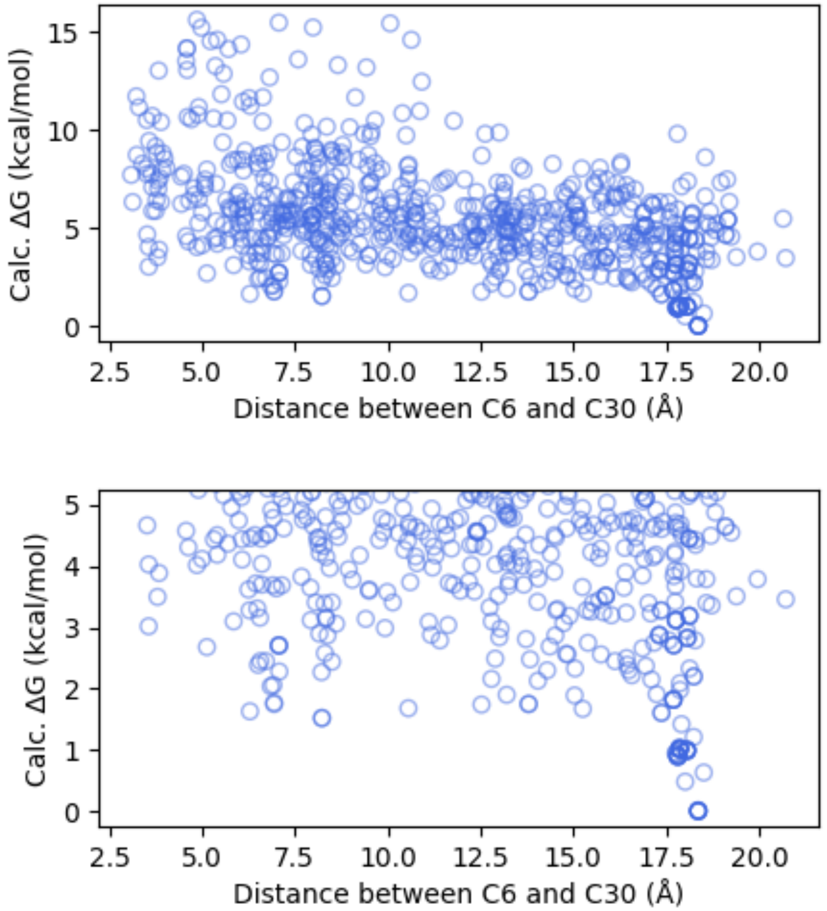

Figure S76. Distribution of the distance between C6 and C30 for conformers of **13'** optimized at the M06-2X/def2-TZVP/(CPCM=MeCN) level of theory.

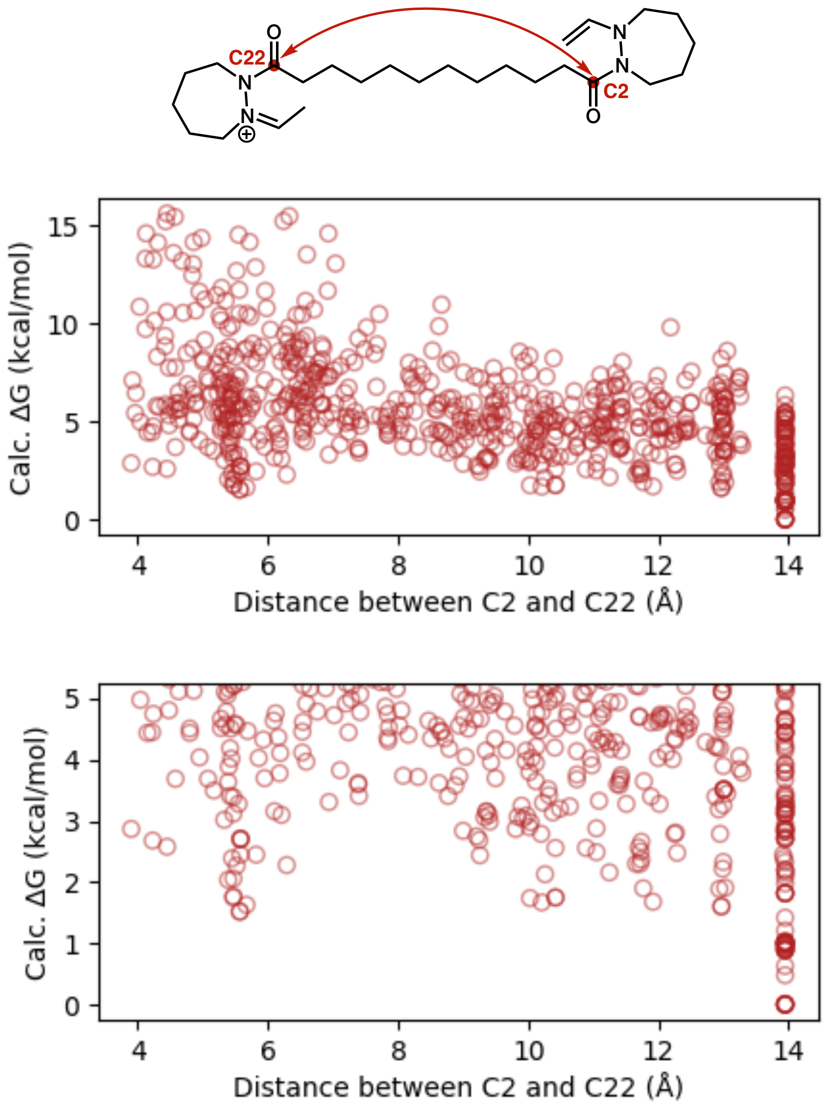

Figure S77. Distribution of the distance between C2 and C22 for conformers of **13'** optimized at the M06-2X/def2-TZVP/(CPCM=MeCN) level of theory.

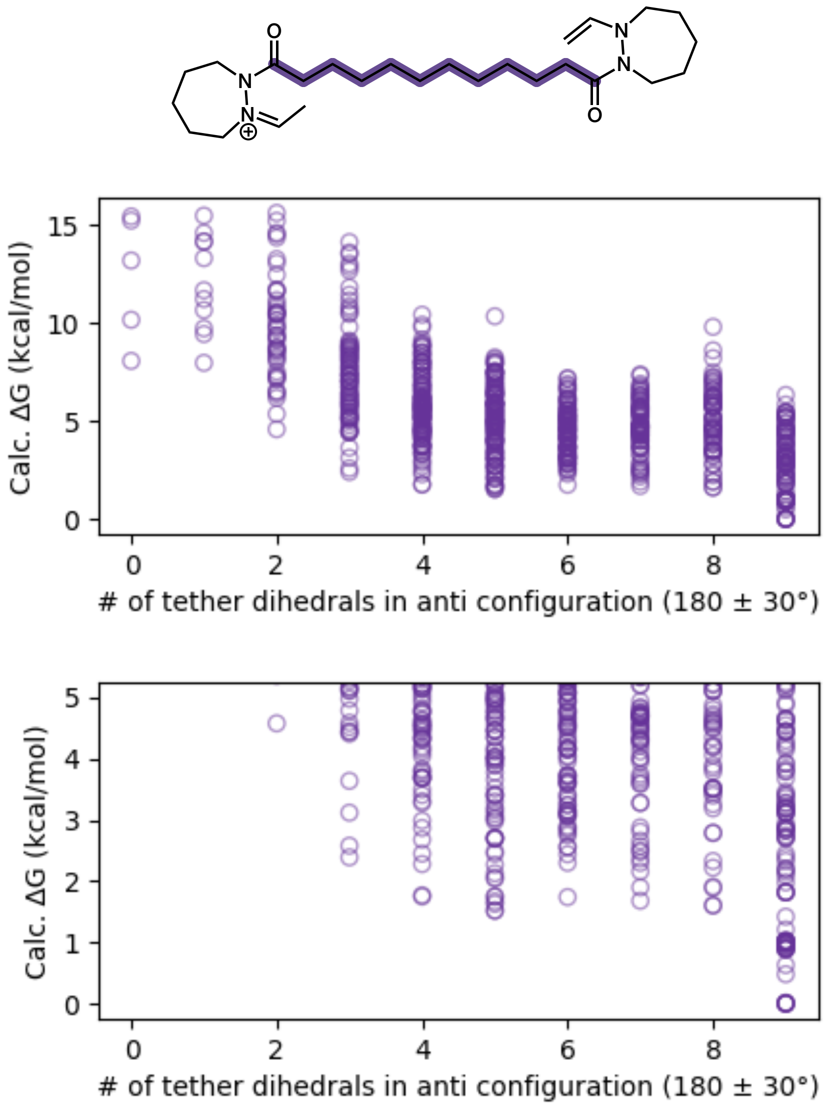

Figure S78. Distribution of the number of anti dihedral angles in the polymethylene tether for conformers of **13’** optimized at the M06-2X/def2-TZVP/(CPCM=MeCN) level of theory.

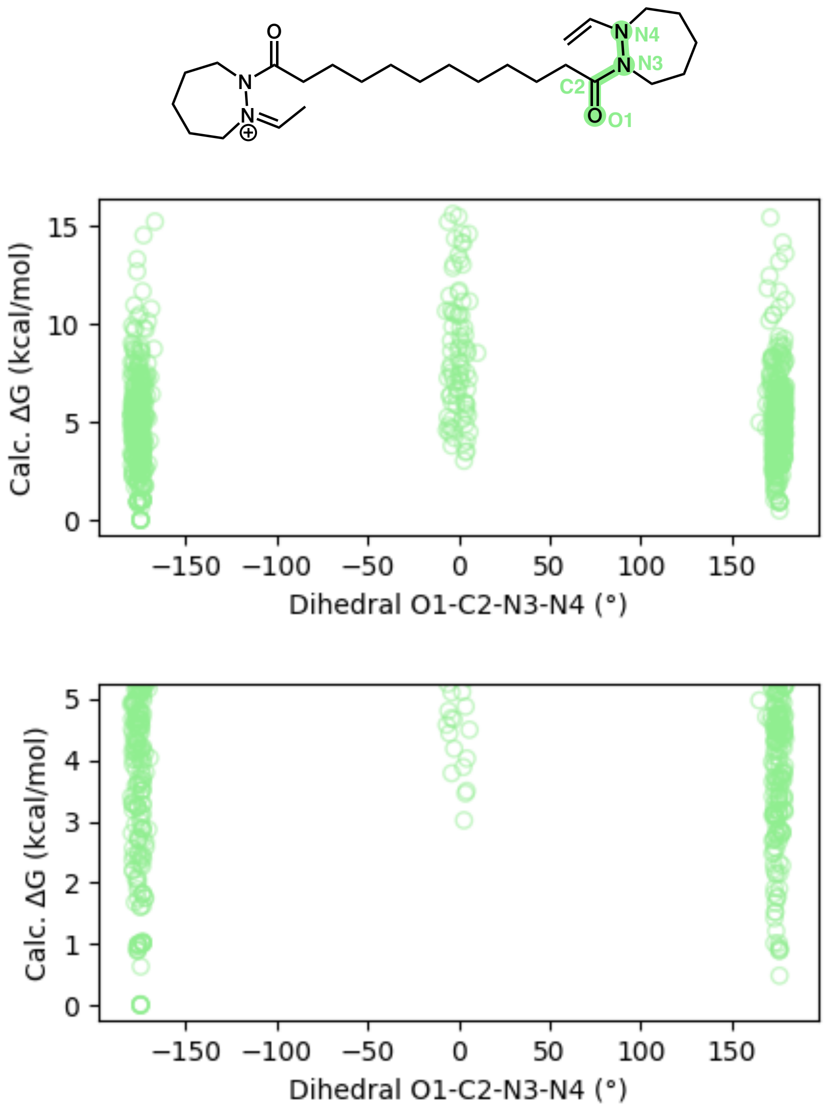

Figure S79. Distribution of O1-C2-N3-N4 dihedral angles for conformers of **13'** optimized at the M06-2X/def2-TZVP/(CPCM=MeCN) level of theory.

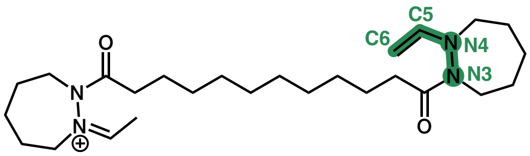

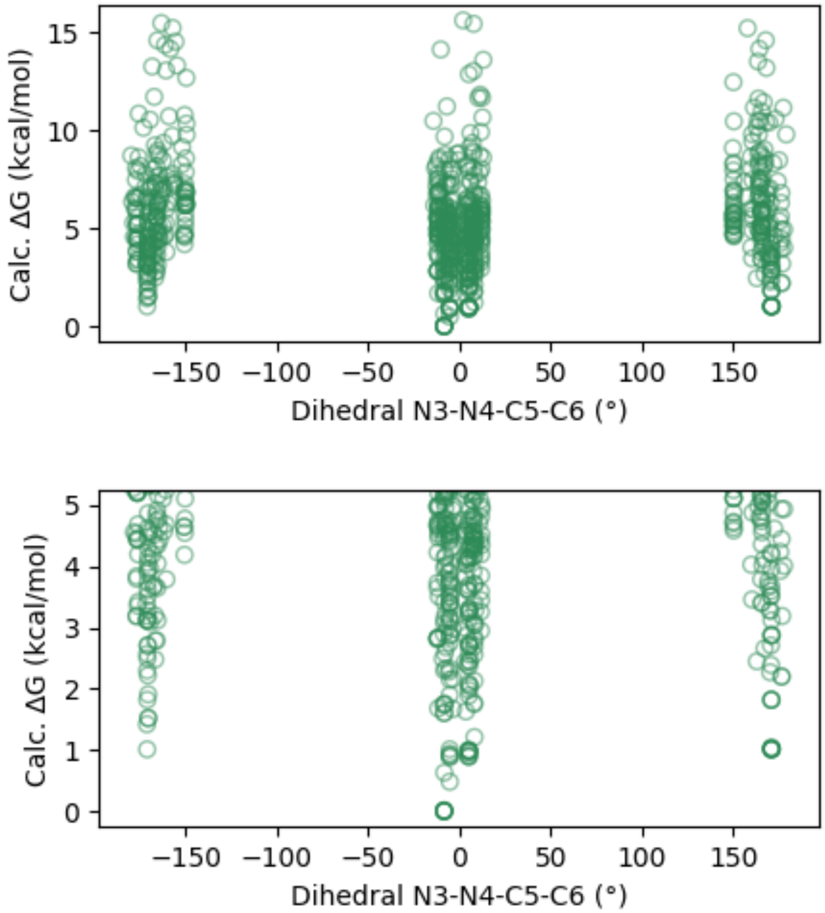

Figure S80. Distribution of N3-N4-C5-C6 dihedral angles for conformers of **13'** optimized at the M06-2X/def2-TZVP/(CPCM=MeCN) level of theory.

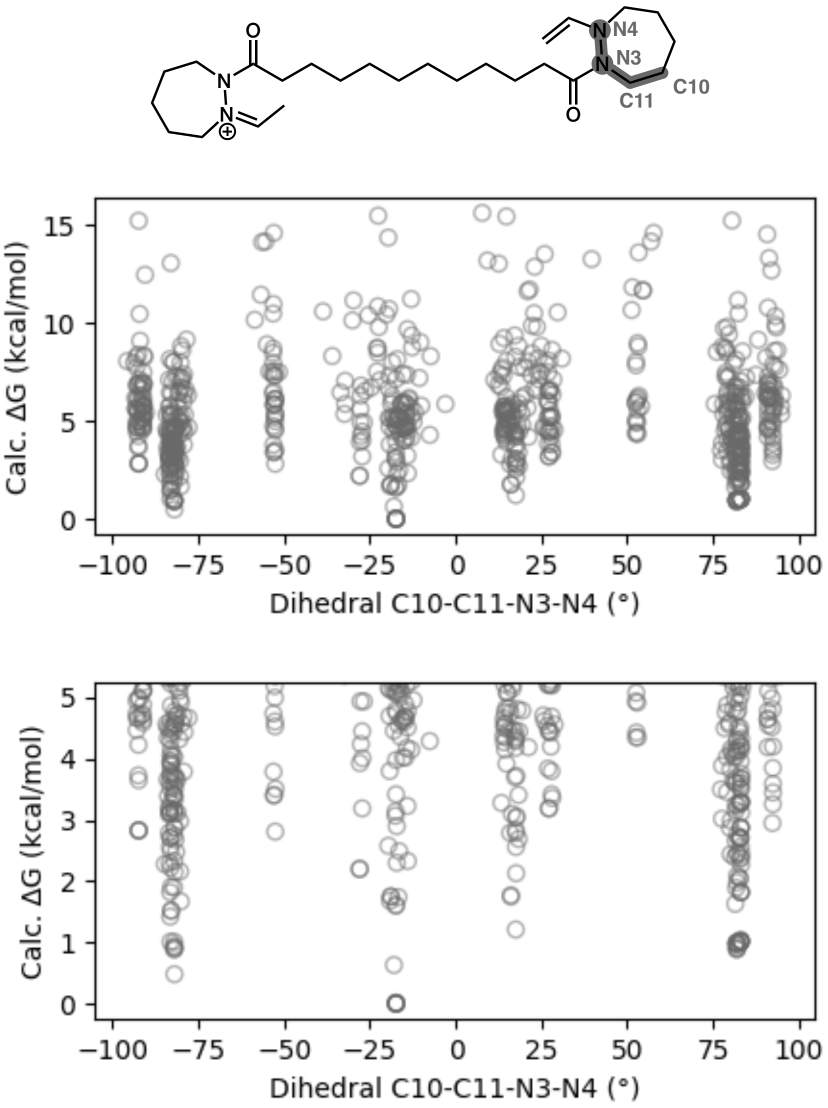

Figure S81. Distribution of C10-C11-N3-N4 dihedral angles for conformers of **13’** optimized at the M06-2X/def2-TZVP/(CPCM=MeCN) level of theory.

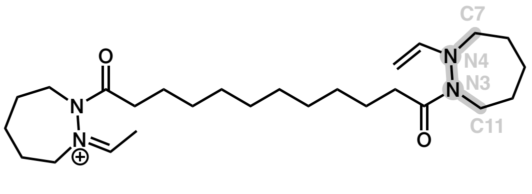

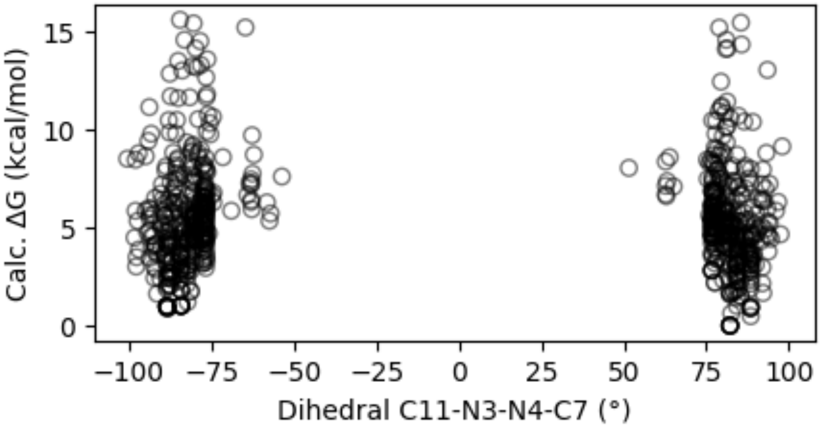

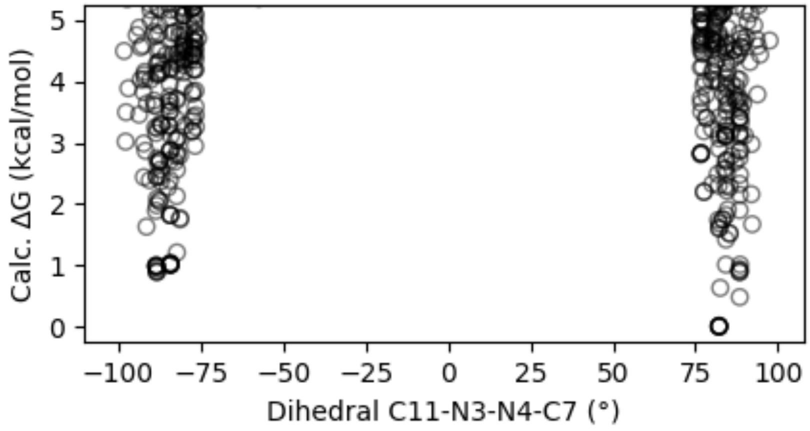

Figure S82. Distribution of C11-N3-N4-C7 dihedral angles for conformers of **13'** optimized at the M06-2X/def2-TZVP/(CPCM=MeCN) level of theory.

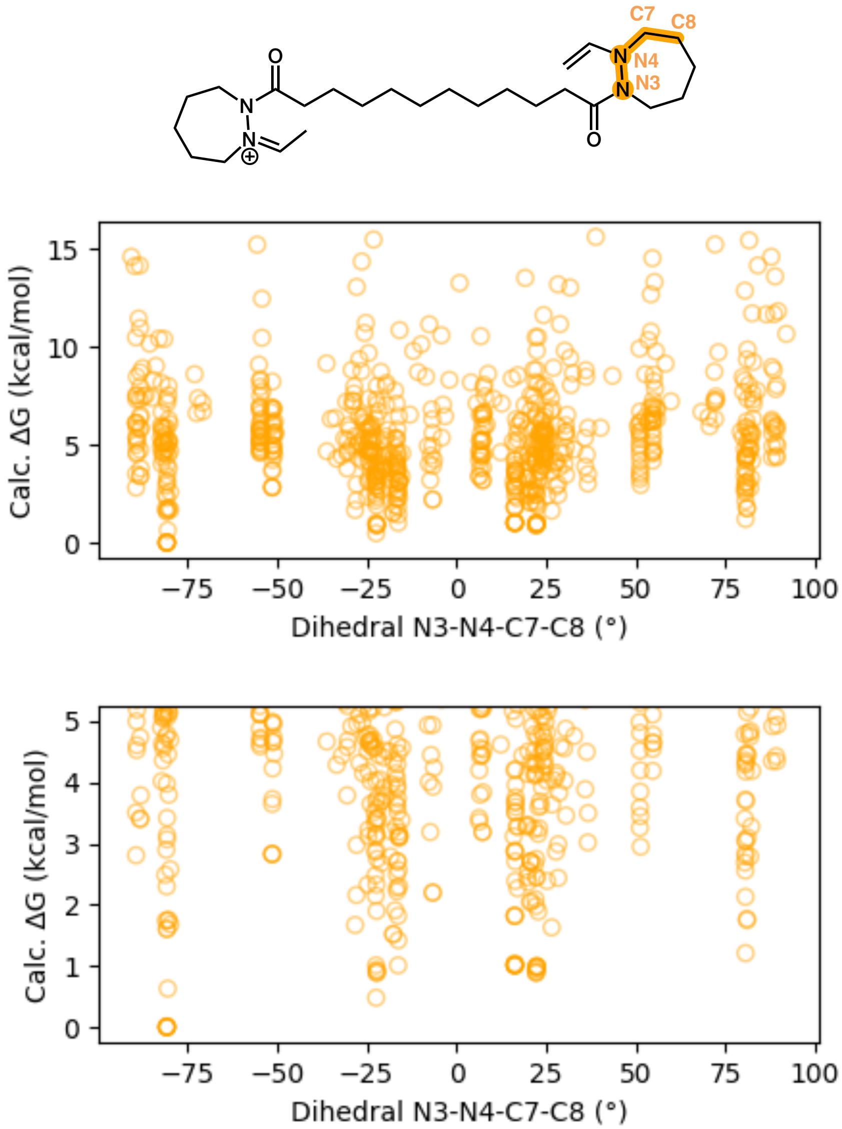

Figure S83. Distribution of N3-N4-C7-C8 dihedral angles for conformers of **13'** optimized at the M06-2X/def2-TZVP/(CPCM=MeCN) level of theory.

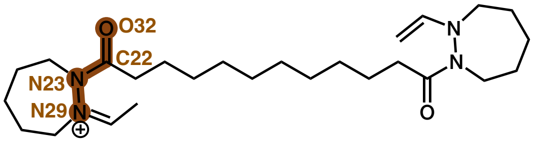

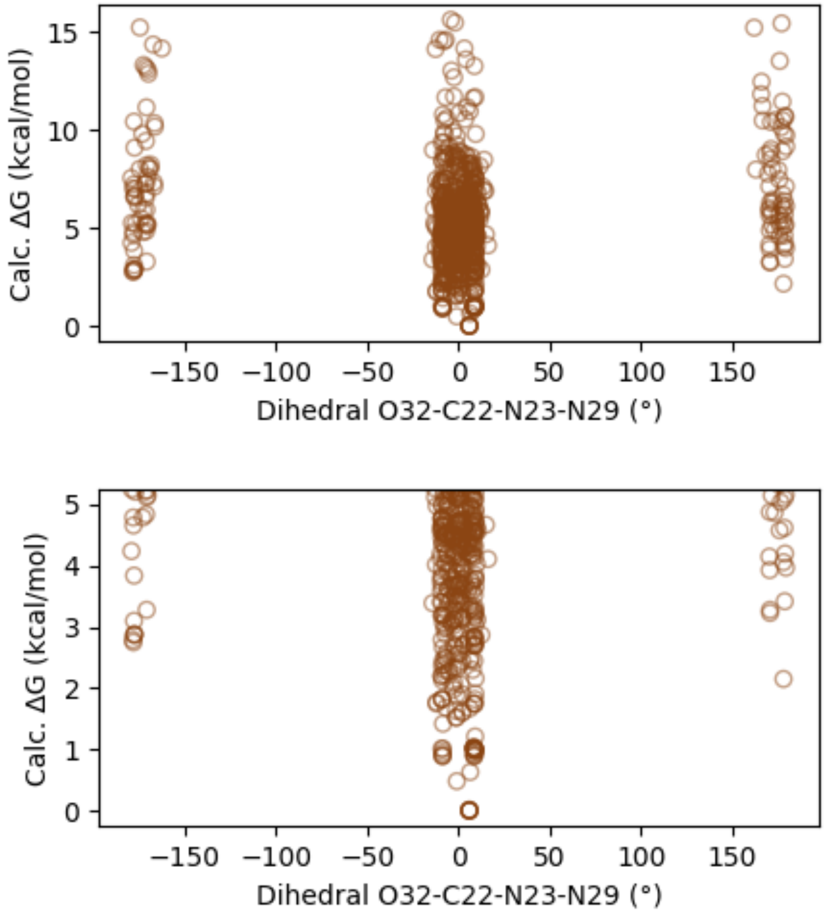

Figure S84. Distribution of O32-C22-N23-N29 dihedral angles for conformers of **13'** optimized at the M06-2X/def2-TZVP/(CPCM=MeCN) level of theory.

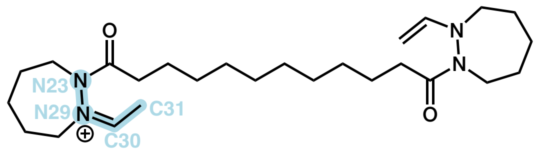

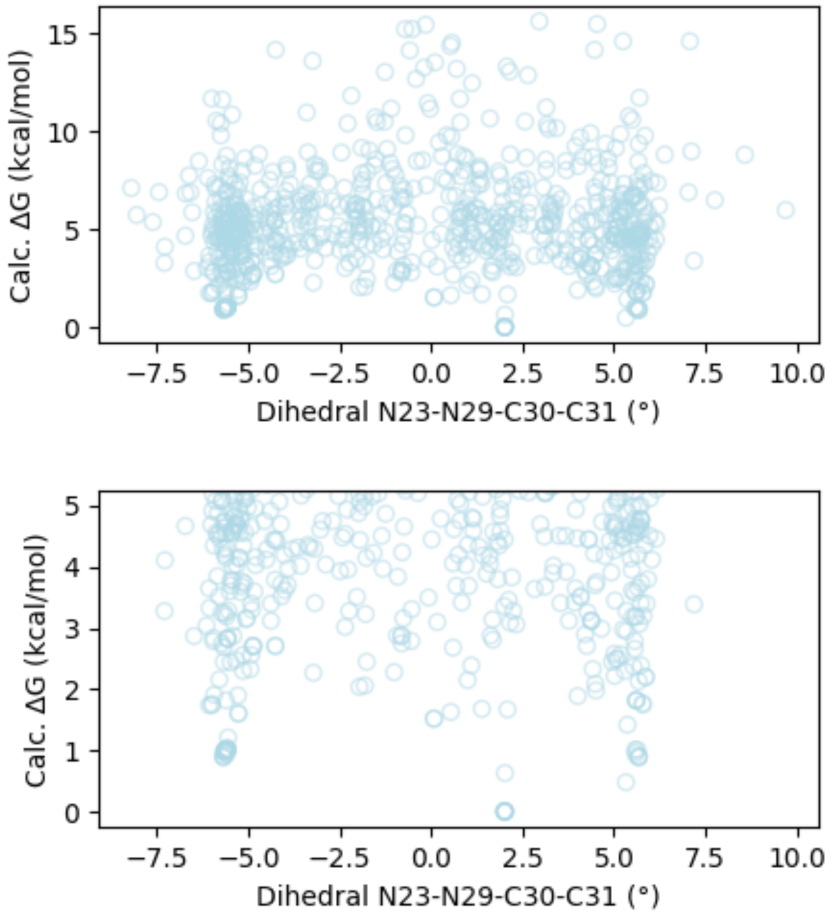

Figure S85. Distribution of N23-N29-C30-C31 dihedral angles for conformers of **13’** optimized at the M06-2X/def2-TZVP/(CPCM=MeCN) level of theory.

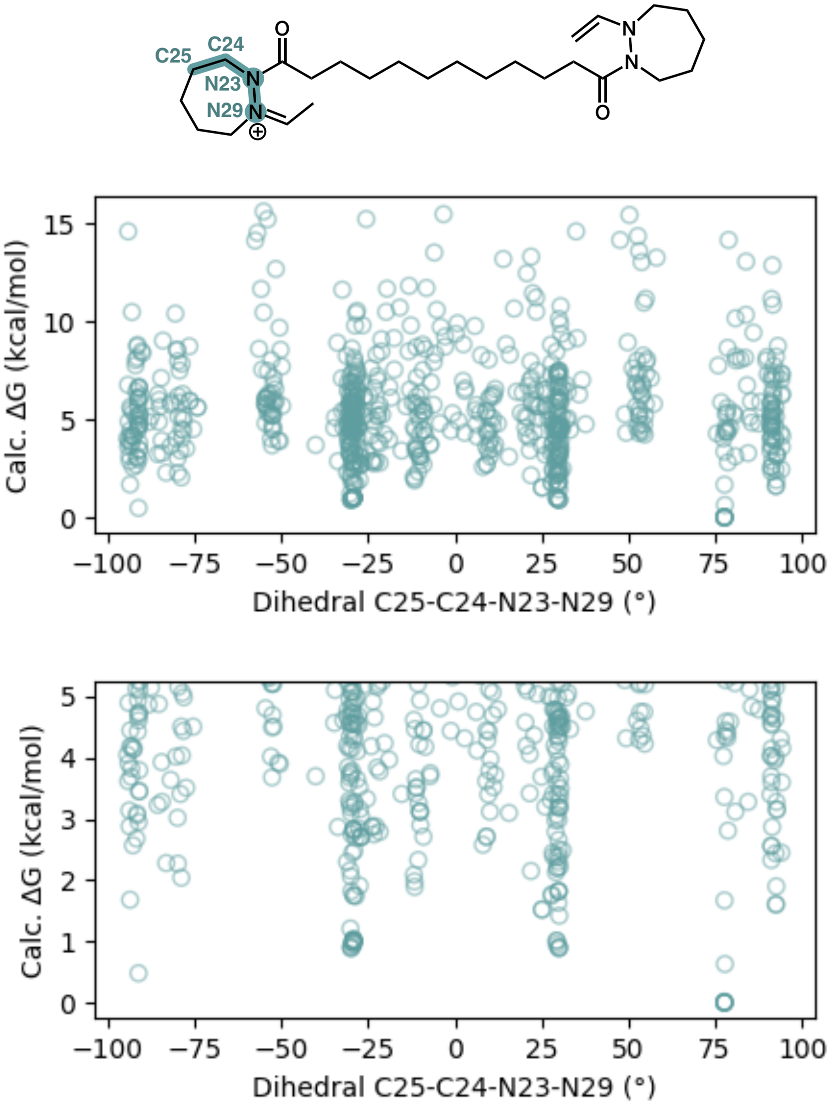

Figure S86. Distribution of C25-C24-N23-N29 dihedral angles for conformers of **13'** optimized at the M06-2X/def2-TZVP/(CPCM=MeCN) level of theory.

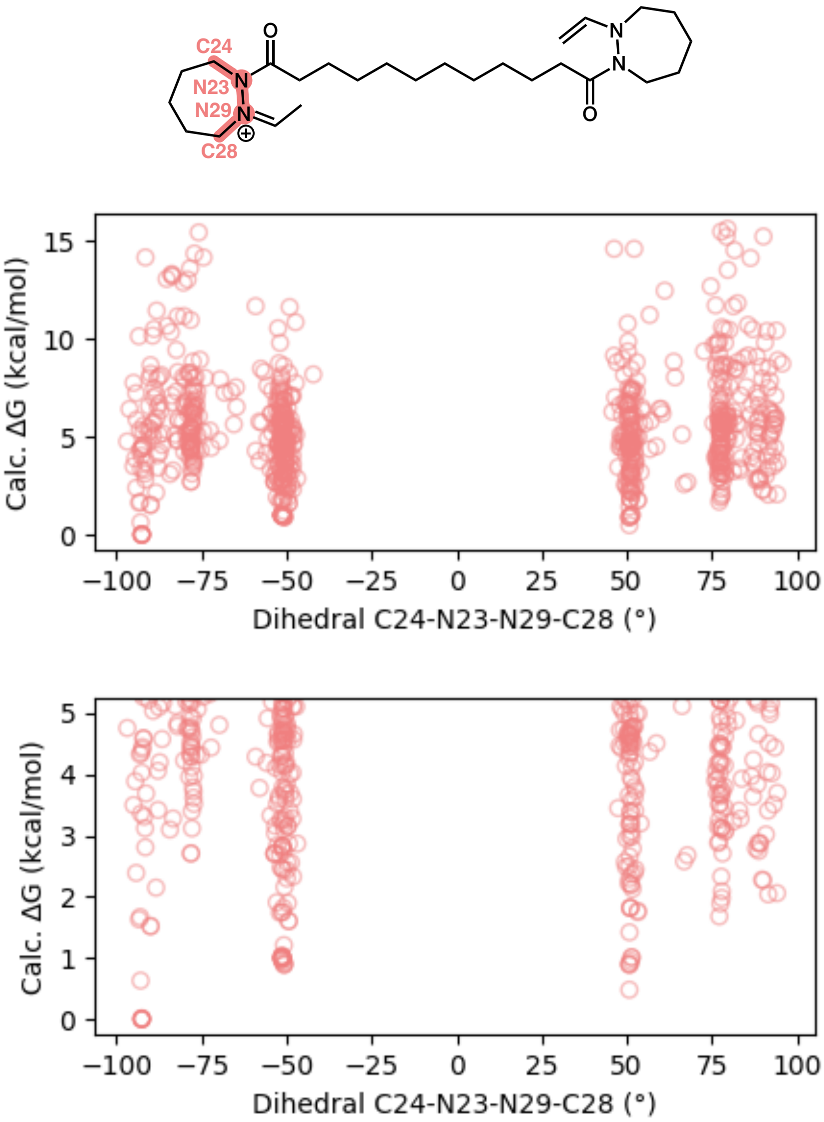

Figure S87. Distribution of C24-N23-N29-C28 dihedral angles for conformers of **13’** optimized at the M06-2X/def2-TZVP/(CPCM=MeCN) level of theory.

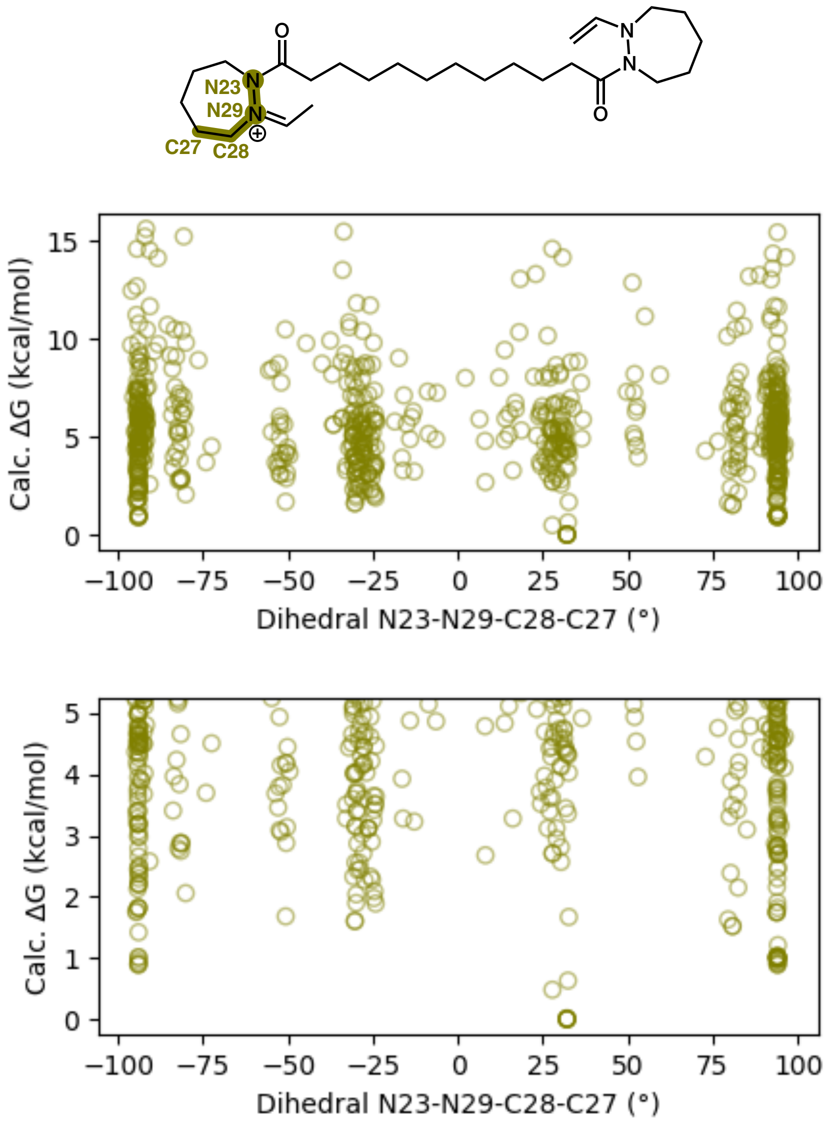

Figure S88. Distribution of N23-N29-C28-C27 dihedral angles for conformers of **13’** optimized at the M06-2X/def2-TZVP/(CPCM=MeCN) level of theory.

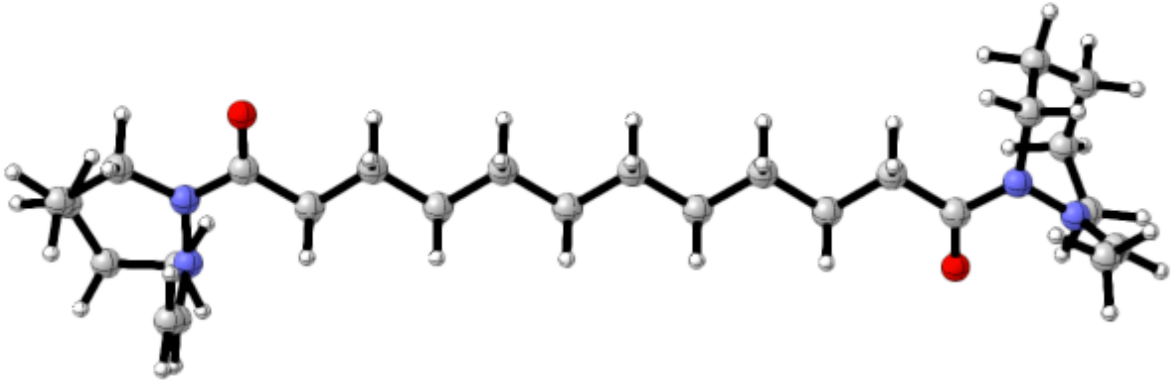

Figure S89. Lowest free energy conformer for **13’** identified at the M06-2X/def2-TZVP/(CPCM=MeCN) level of theory.

7. NMR, UPLC and MS Data for New Compounds

Fmoc-L-Glu(HyBoc)-OH (S5):

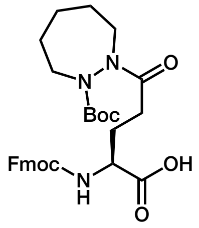

S5

^{1}H NMR (500 MHz, $CDCl_3$, mixture of rotamers):

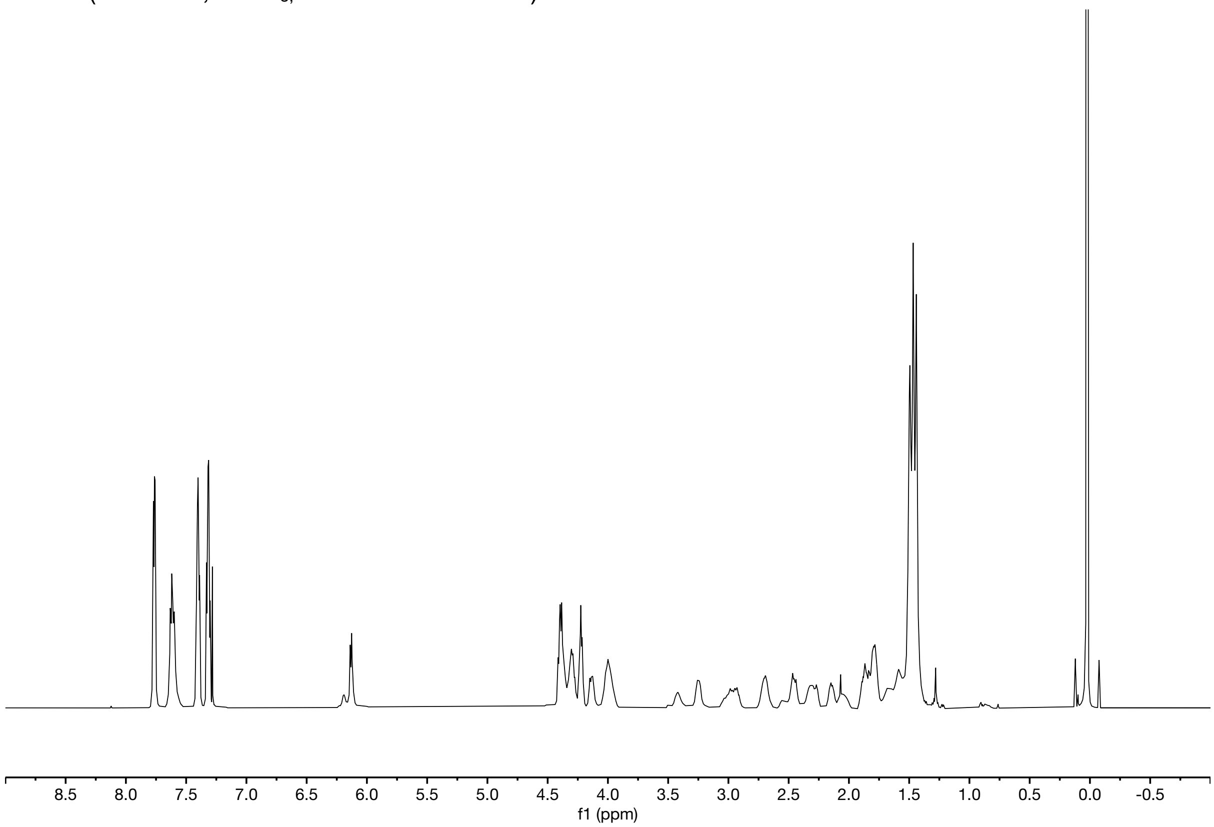

^{13}C NMR (125.725 MHz, $CDCl_3$, mixture of rotamers):

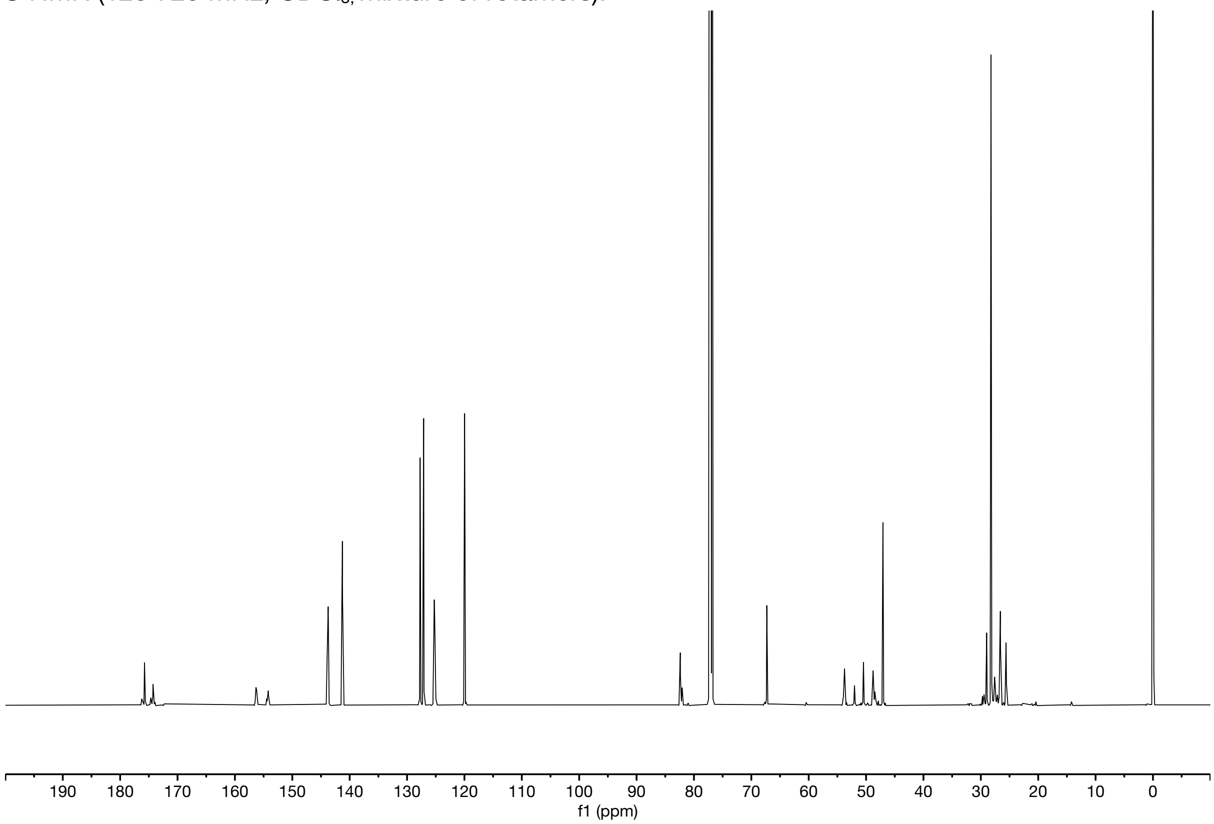

HR-ESI-MS:

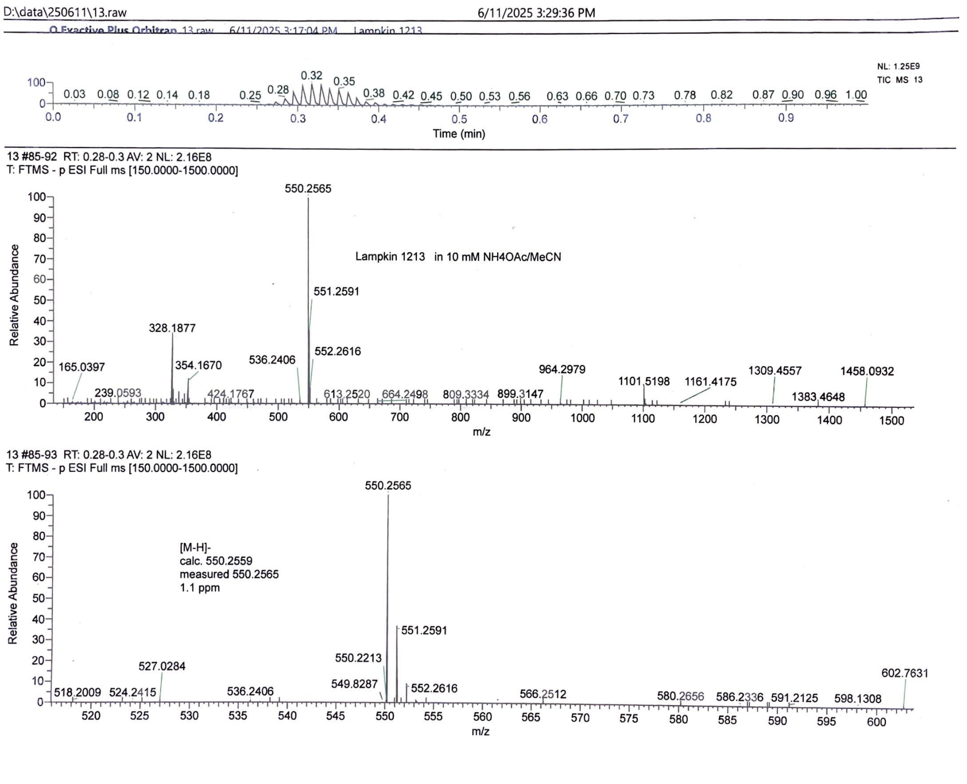

Fmoc-L-Asp(HyBoc)-OH (S7):

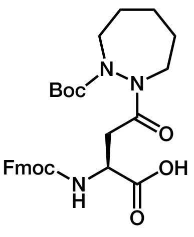

S7

^{1}H NMR (500 MHz, $CDCl_3$, mixture of rotamers):

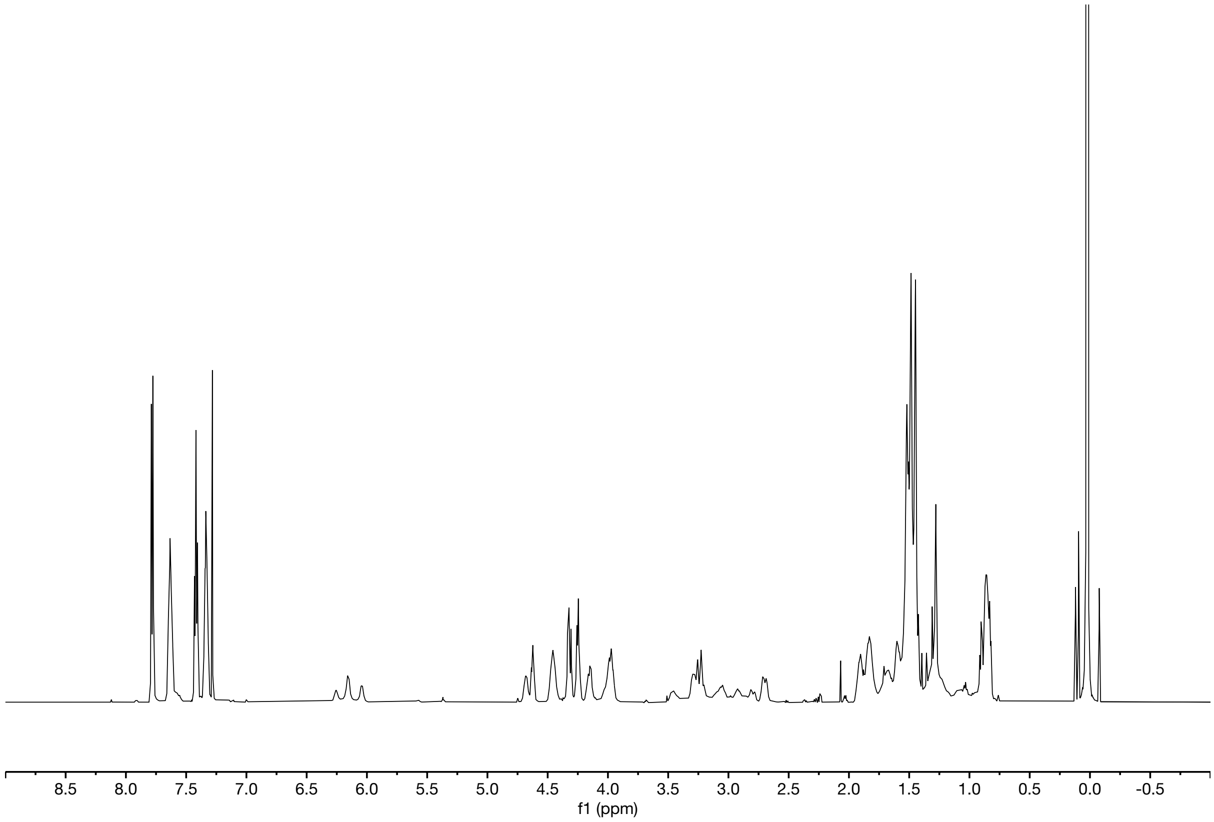

^{13}C NMR (125.725 MHz, $CDCl_3$, mixture of rotamers):

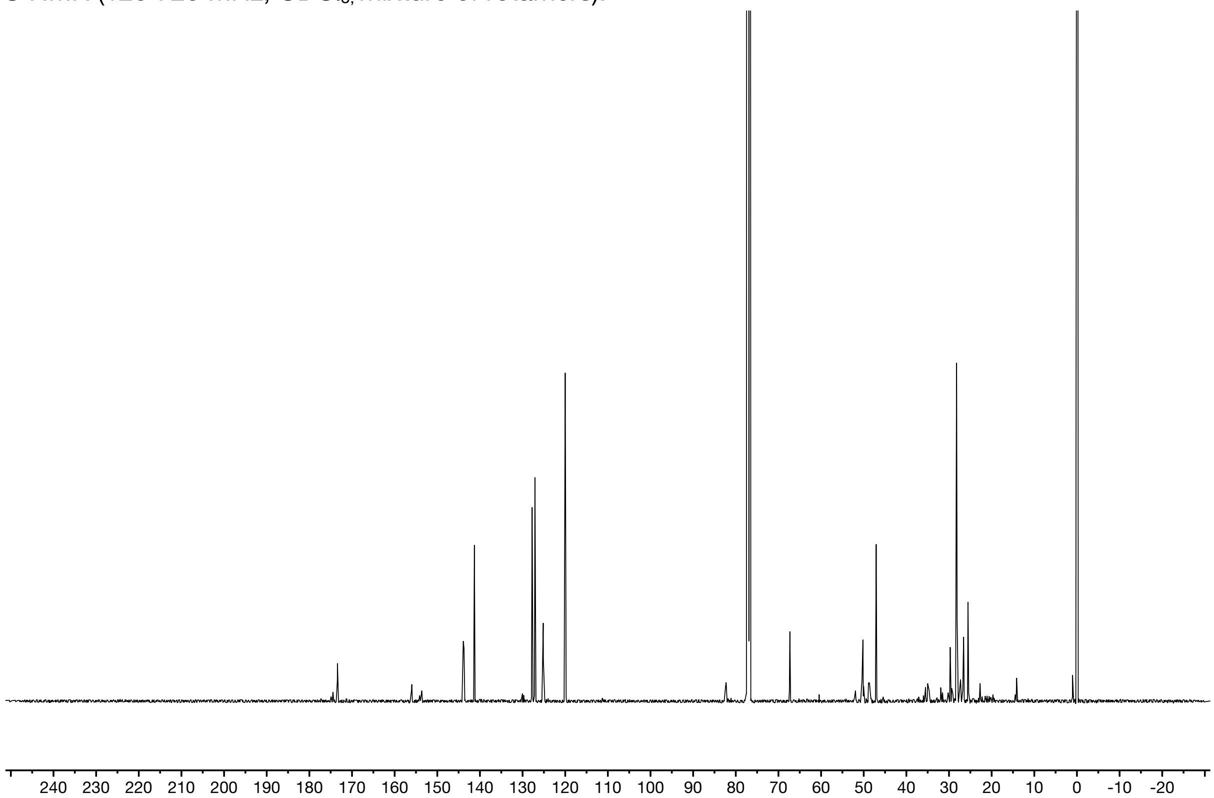

HR-ESI-MS:

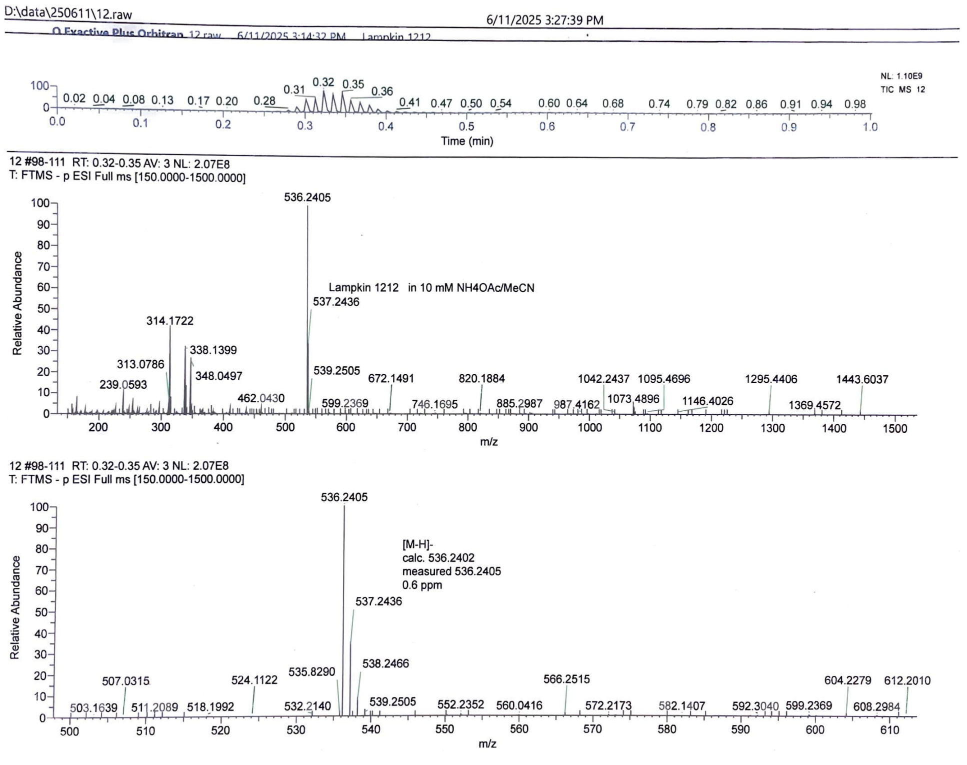

HO-Succ(HyBoc) (S10):

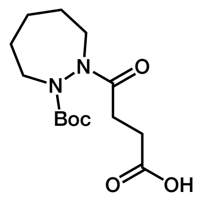

S10

^{1}H NMR (500 MHz, $CDCl_3$, mixture of rotamers):

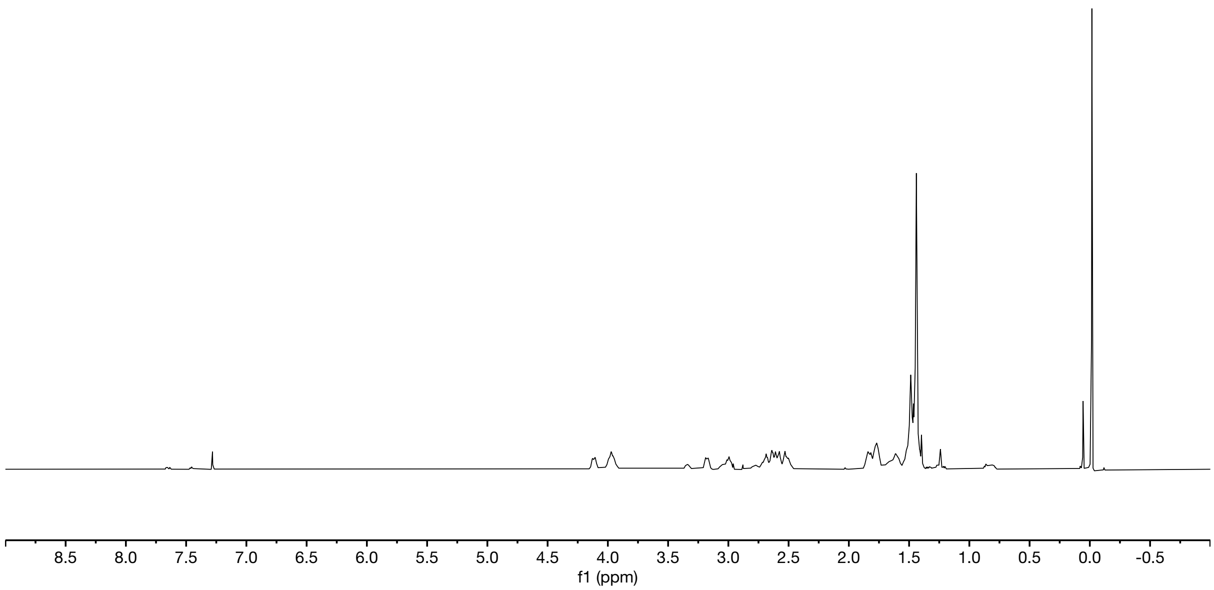

^{13}C NMR (125.725 MHz, $CDCl_3$, mixture of rotamers):

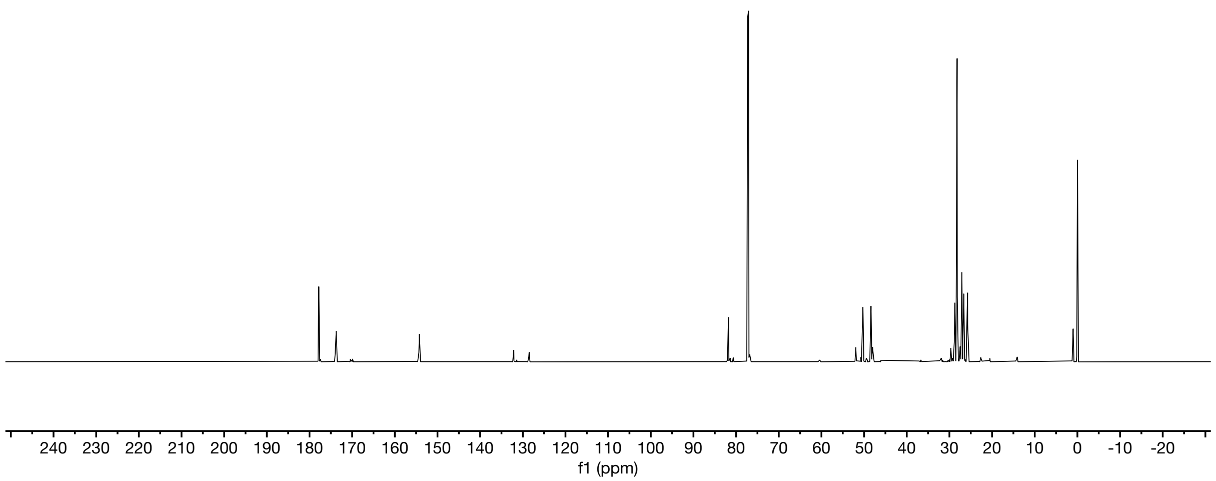

HR-ESI-MS:

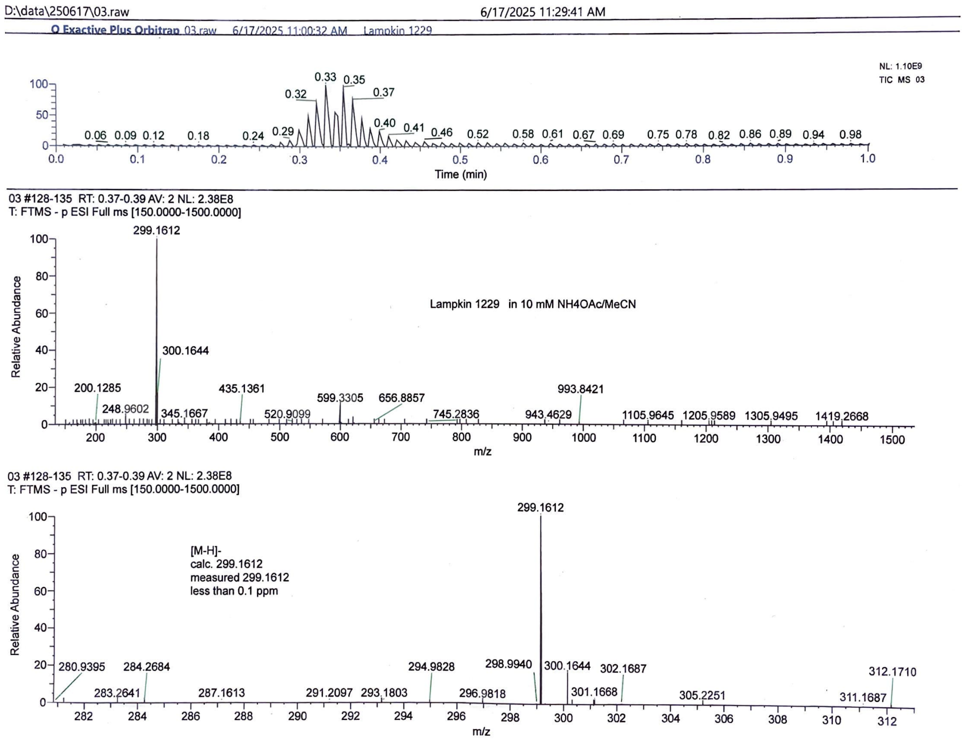

TPP-ACPC-ACPC-Glu(Hy)-ACPC-ACPC-Ala-B^{3}HTyr-C(O)NHMe (2)

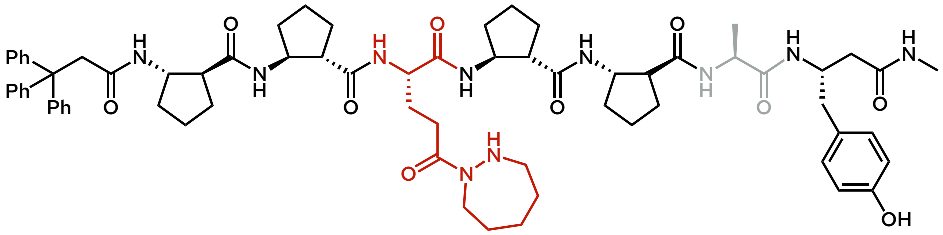

UPLC (220 nm channel, Purity = 98%):

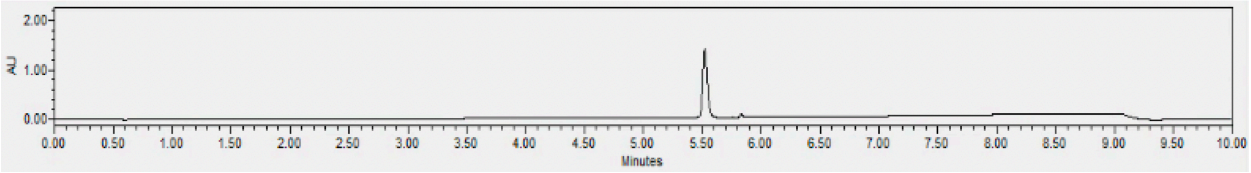

HR-ESI-MS:

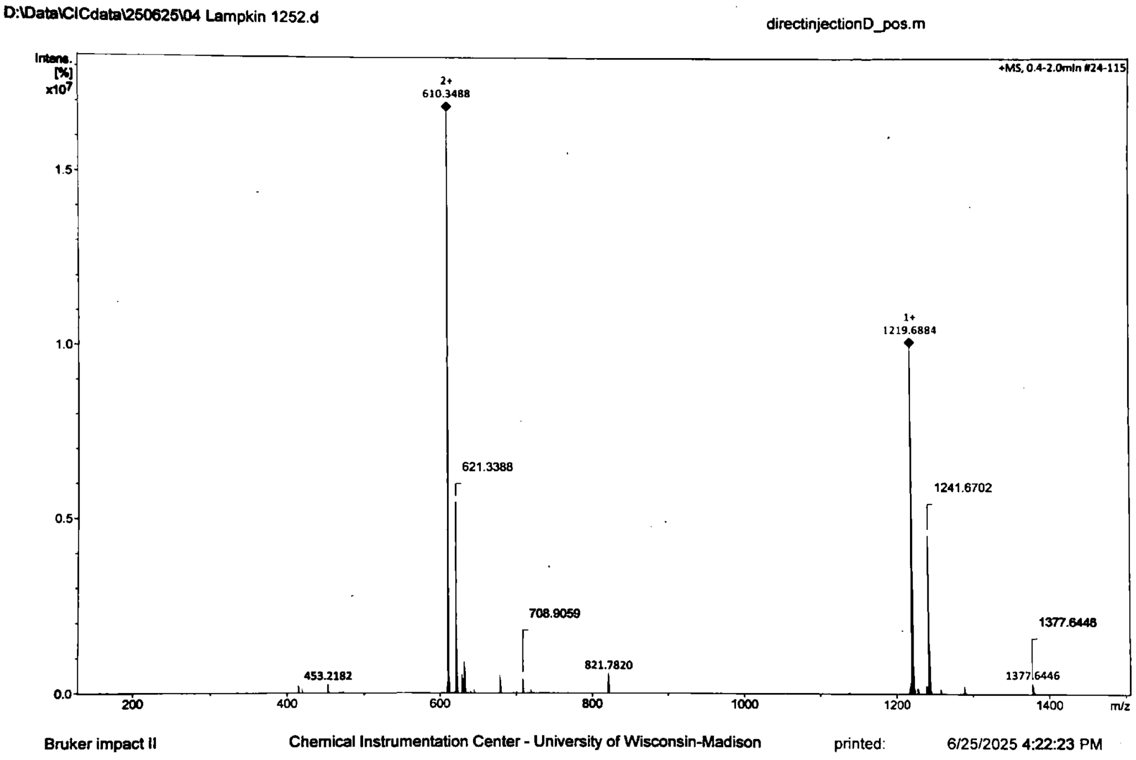

m/z observed: 1219.6884 ([M+H]$^{+}$) and 610.3488 ([M+2H]$^{2+}$)

m/z calculated: $C_{69}H_{91}N_{10}O_{10}^{+}$ = 1219.6914 ([M+H]$^{+}$) and $C_{69}H_{92}N_{10}O_{10}^{2+}$ = 610.3493 ([M+2H]$^{2+}$)

TPP-ACPC-ACPC-Ala-ACPC-ACPC-Glu(Hy)-B³HTyr-C(O)NHMe (3)

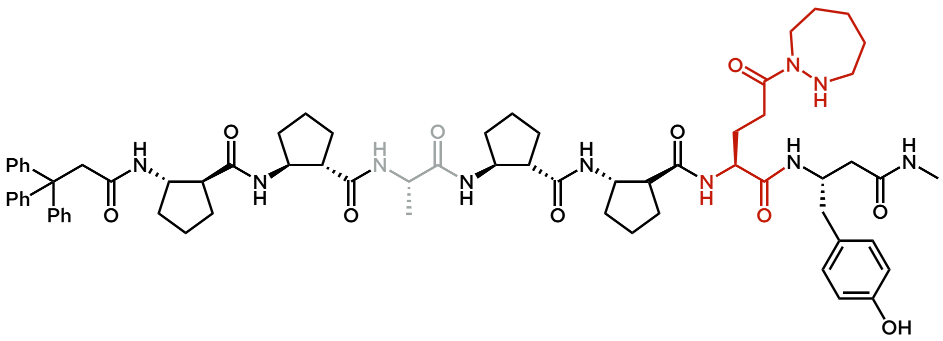

UPLC (220 nm channel, Purity = 98%):

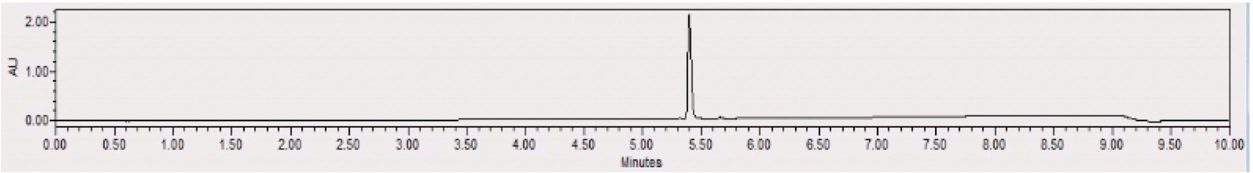

HR-ESI-MS:

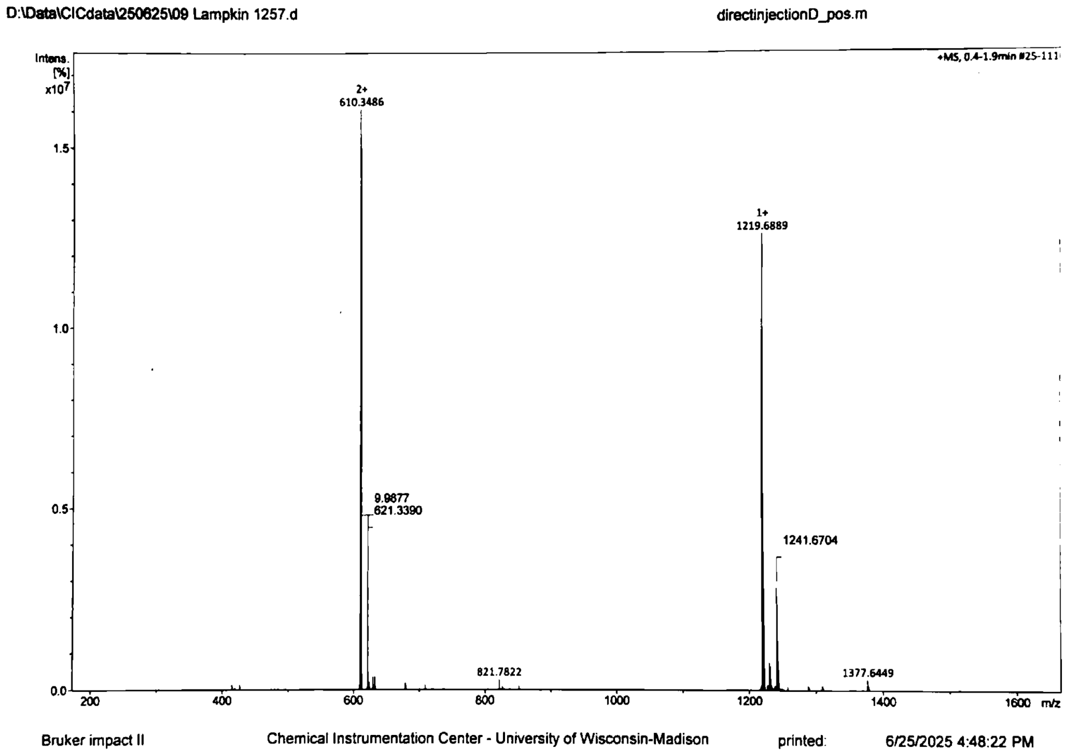

m/z observed: 1219.6889 ([M+H]$^{+}$) and 610.3486 ([M+2H]$^{2+}$)
m/z calculated: $C_{69}H_{91}N_{10}O_{10}^{+}$ = 1219.6914 ([M+H]$^{+}$) and $C_{69}H_{92}N_{10}O_{10}^{2+}$ = 610.3493 ([M+2H]$^{2+}$)

TPP-ACPC-ACPC-Ala-ACPC-ACPC-Asp(Hy)-β³HTyr-C(O)NHMe (4)

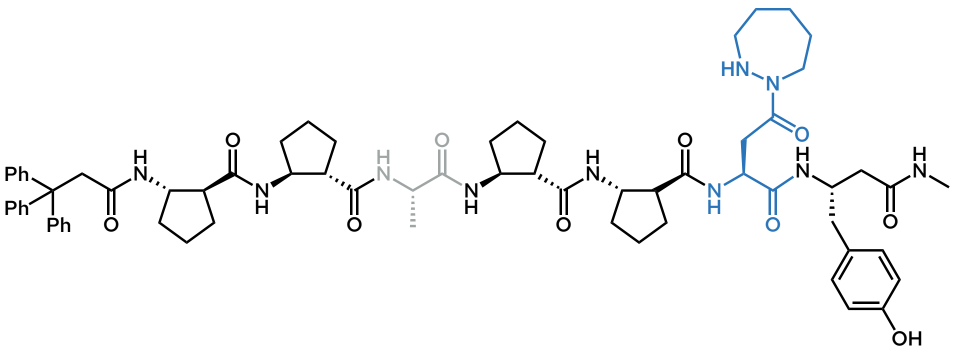

UPLC (220 nm channel, Purity = 97%):

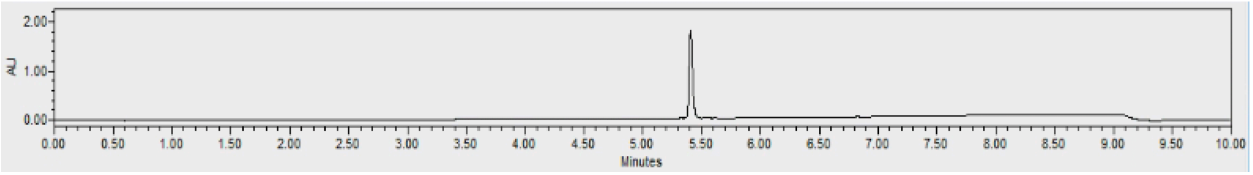

HR-ESI-MS:

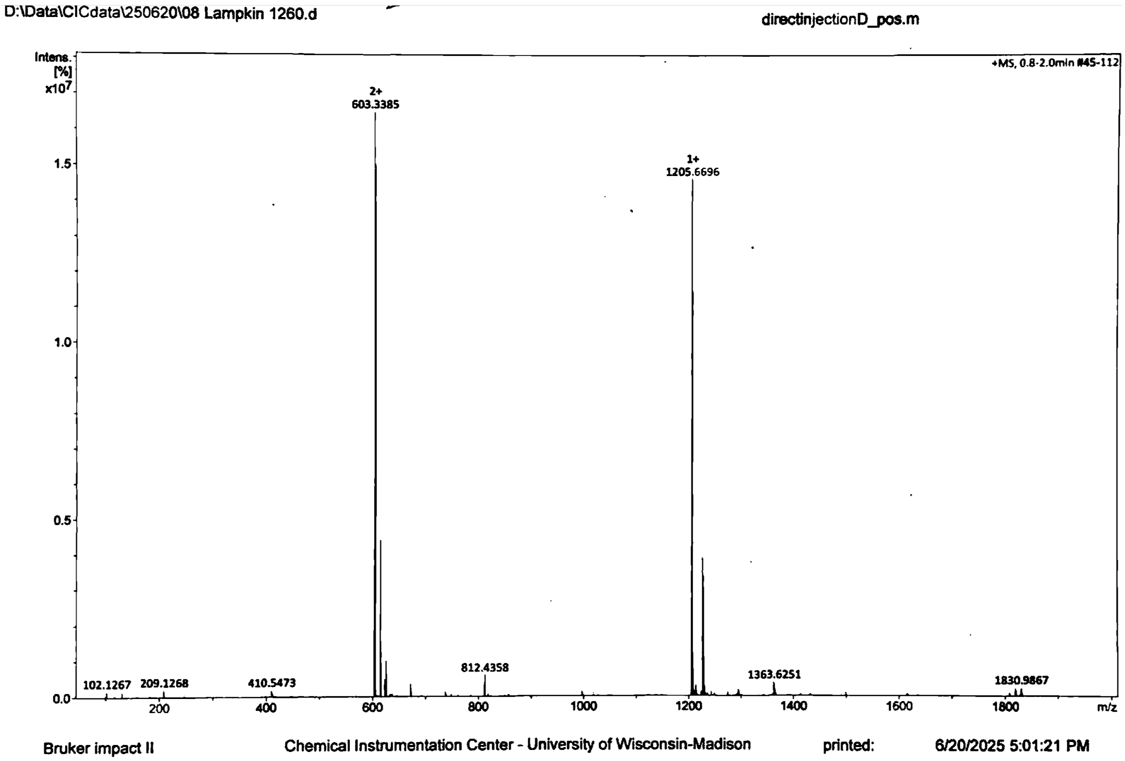

m/z observed: 1205.6696 ([M+H]$^{+}$) and 603.3385 ([M+2H]$^{2+}$)

m/z calculated: $C_{68}H_{89}N_{10}O_{10}^{+}$ = 1205.6758 ([M+H]$^{+}$) and $C_{68}H_{90}N_{10}O_{10}^{2+}$ = 603.3415 ([M+2H]$^{2+}$)

TPP-ACPC-ACPC-Glu(Hy)-ACPC-ACPC-Glu(Hy)-B^{3}HTyr-C(O)NHMe (5)

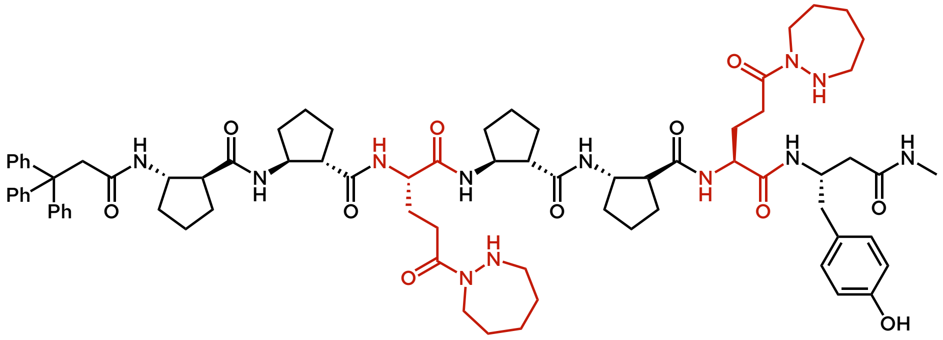

UPLC (220 nm channel, Purity = 98%):

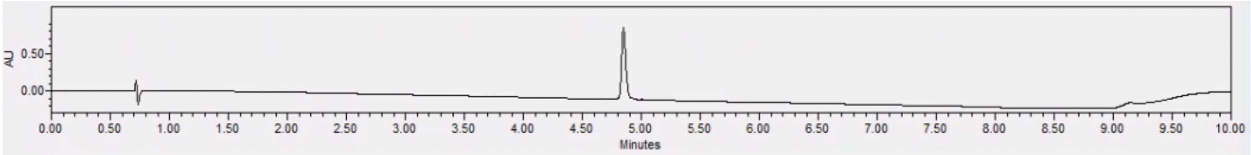

HR-ESI-MS:

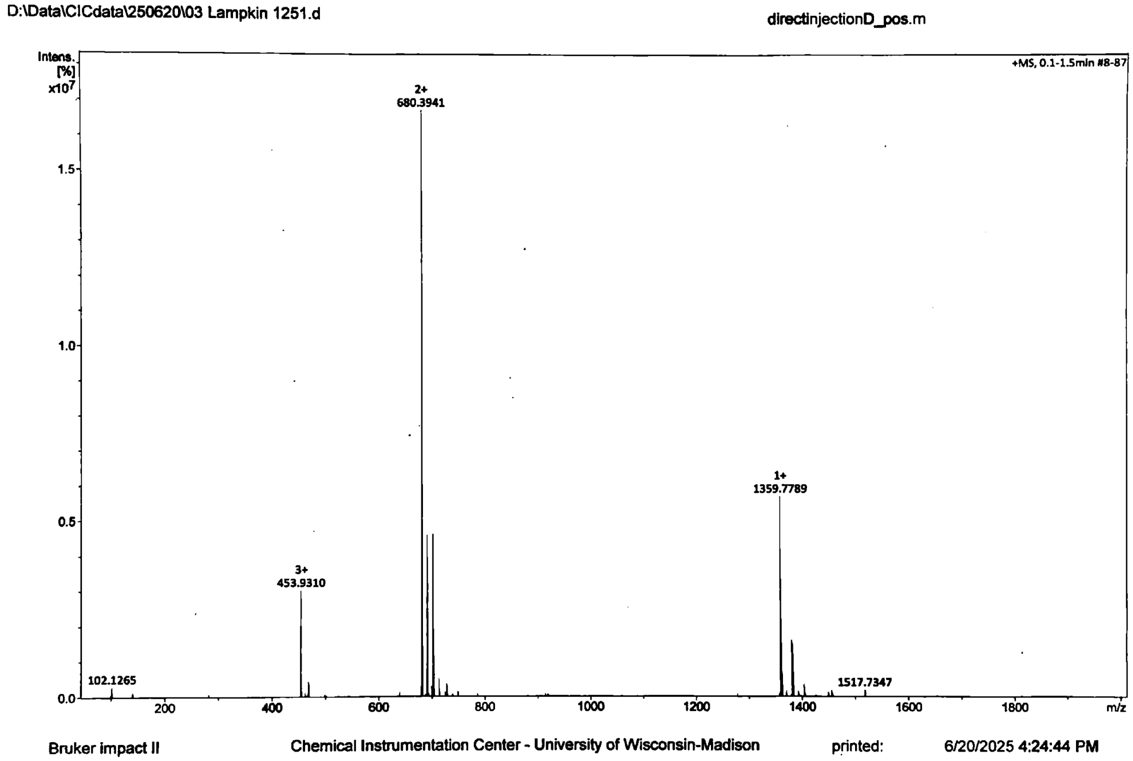

m/z observed: 1359.7789 ([M+H]$^{+}$) and 680.3941 ([M+2H]$^{2+}$)

m/z calculated: $C_{76}H_{103}N_{12}O_{11}^{+}$= 1359.7864 ([M+H]$^{+}$) and $C_{76}H_{104}N_{12}O_{11}^{2+}$= 680.3968 ([M+2H]$^{2+}$)

TPP-ACPC-ACPC-Asp(Hy)-ACPC-ACPC-Asp(Hy)-B^{3}HTyr-C(O)NHMe (6)

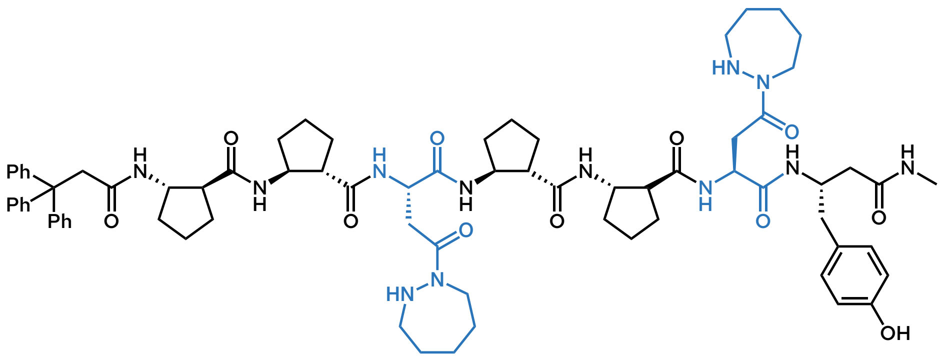

UPLC (220 nm channel, Purity = 96%):

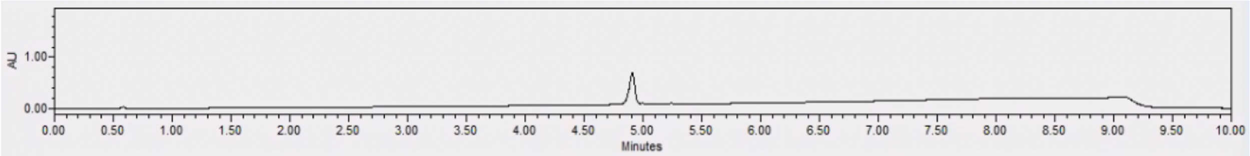

HR-ESI-MS:

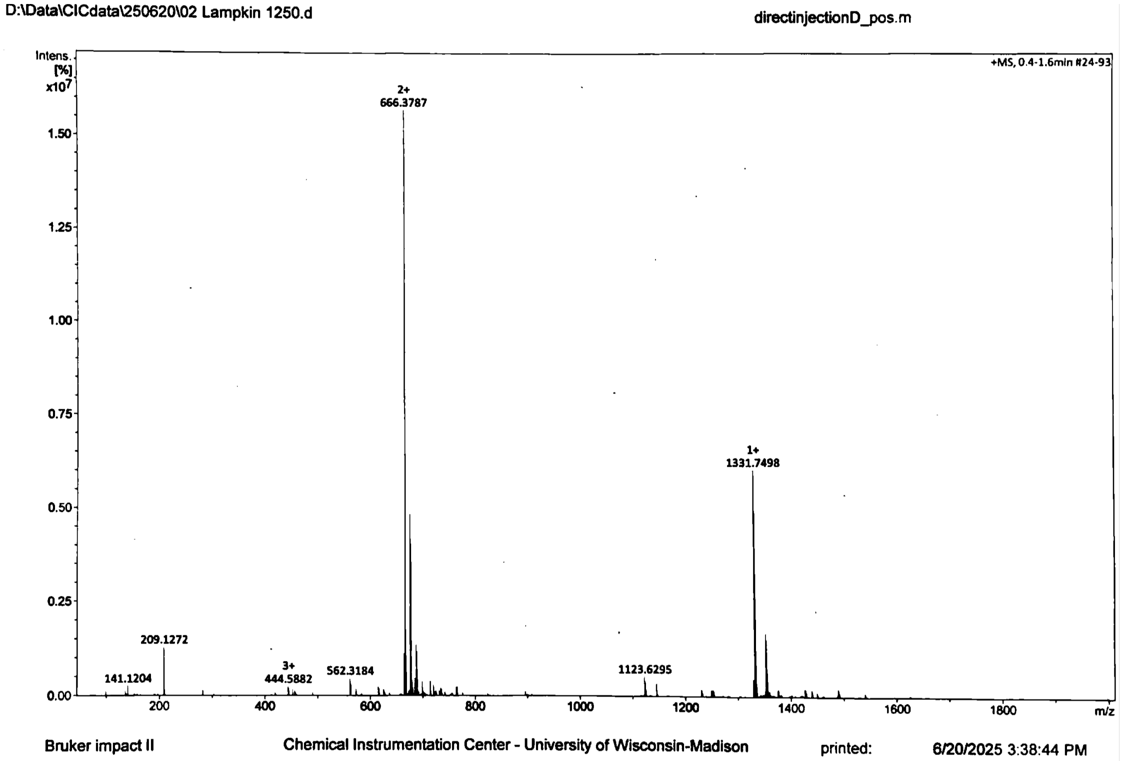

m/z observed: 1331.7498 ([M+H]$^+$) and 666.3787 ([M+2H]$^{2+}$)

m/z calculated: $C_{74}H_{99}N_{12}O_{11}^+$= 1331.7551 ([M+H]$^+$) and $C_{74}H_{100}N_{12}O_{11}^{2+}$= 666.3812 ([M+2H]$^{2+}$)

TPP-ACPC-ACPC-Glu(Hy)-ACPC-ACPC-Asp(Hy)-β^{3}HTyr-C(O)NHMe (7)

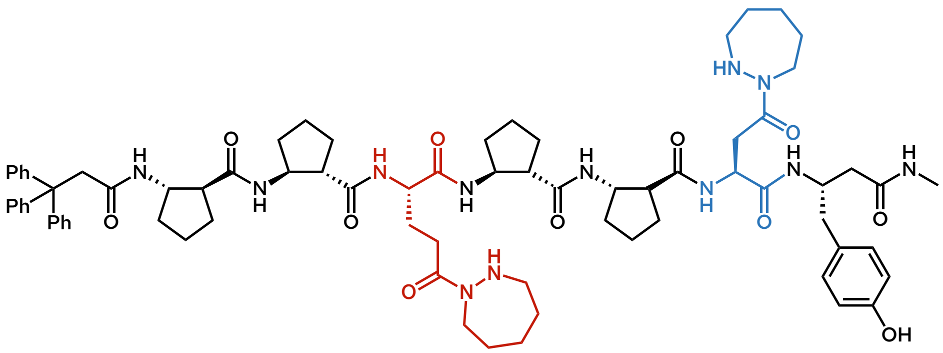

UPLC (220 nm channel, Purity = 89%):

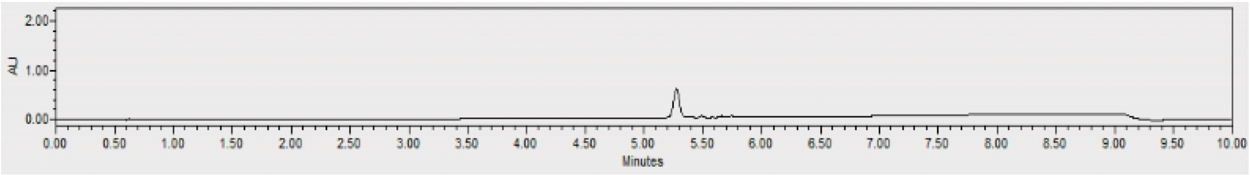

HR-ESI-MS:

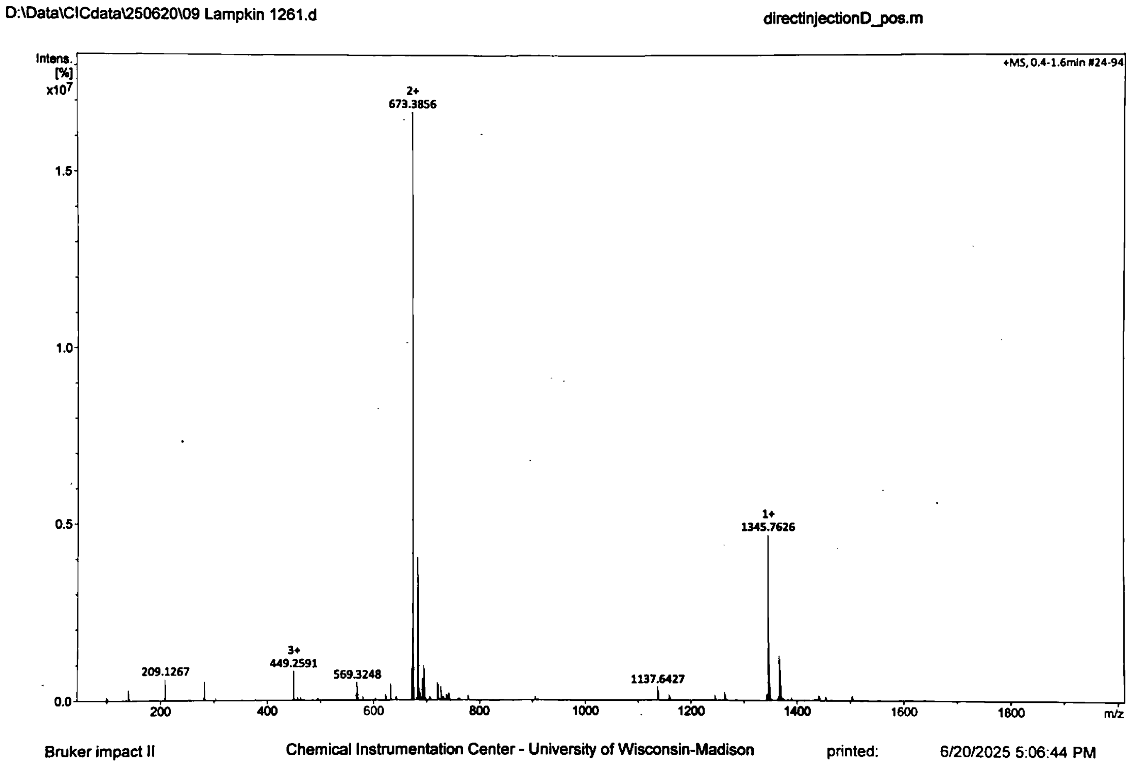

m/z observed: 1345.7626 ([M+H]$^+$) and 673.3856 ([M+2H]$^{2+}$)

m/z calculated: $C_{75}H_{101}N_{12}O_{11}{}^+$= 1345.7707 ([M+H]$^+$) and $C_{75}H_{102}N_{12}O_{11}{}^{2+}$= 673.3890 ([M+2H]$^{2+}$)

TPP-ACPC-ACPC-Asp(Hy)-ACPC-ACPC-Glu(Hy)-β³HTyr-C(O)NHMe (8)

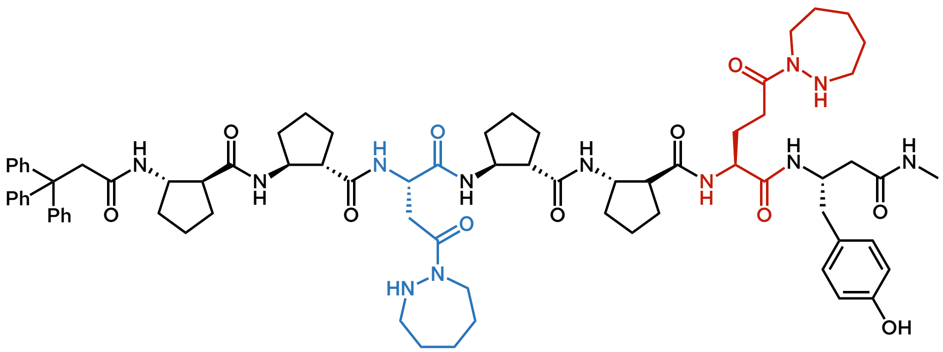

UPLC (220 nm channel, Purity = 87%):

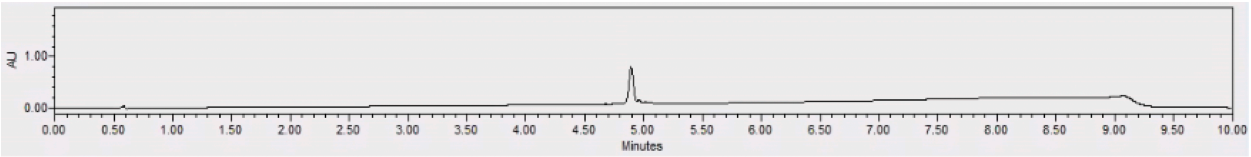

HR-ESI-MS:

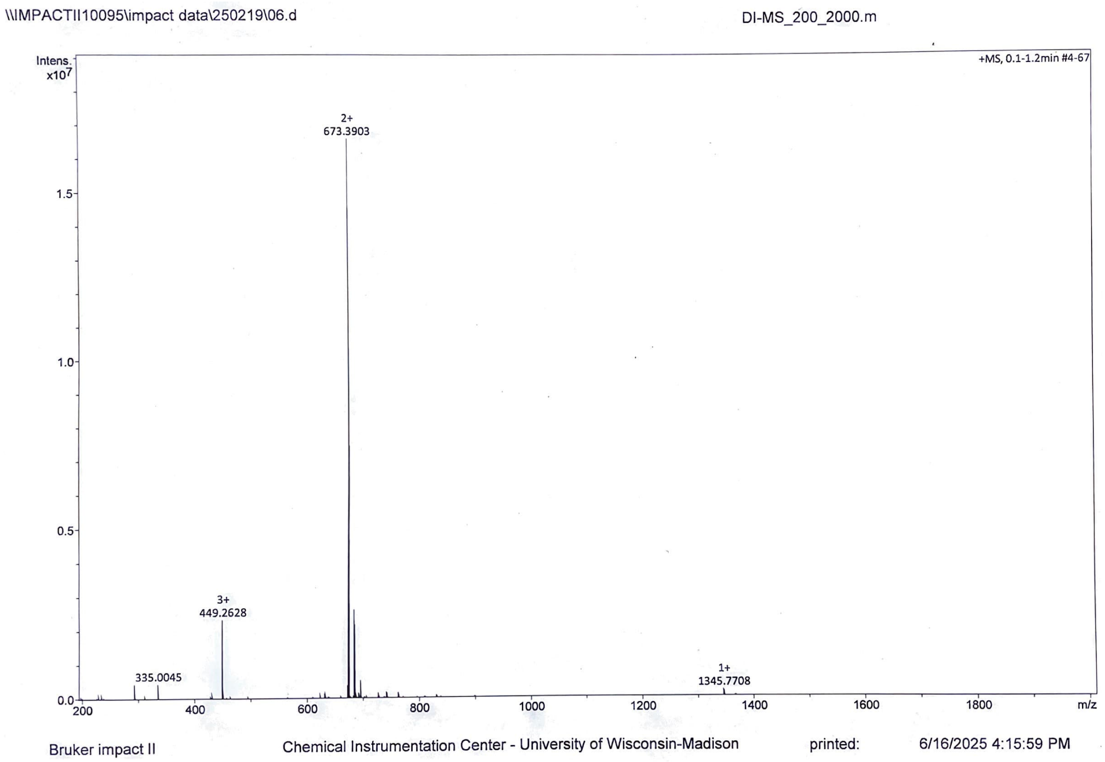

m/z observed: 1345.7708 ($[M+H]^+$) and 673.3903 ($[M+2H]^{2+}$)

m/z calculated: $C_{75}H_{101}N_{12}O_{11}^+$= 1345.7707 ($[M+H]^+$) and $C_{75}H_{102}N_{12}O_{11}^{2+}$= 673.3890 ($[M+2H]^{2+}$)

TPP-ACPC-ACPC-Glu(Hy)-ACPC-ACPC-Dap(SuccHy)-β^{3}HTyr-C(O)NHMe (9)

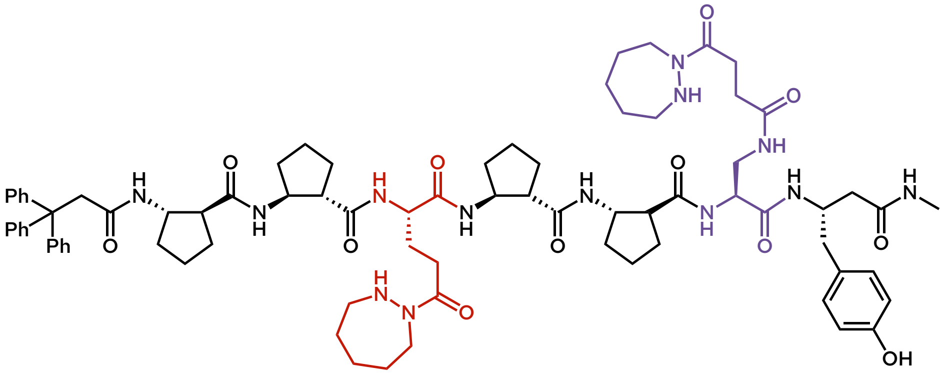

UPLC (220 nm channel, Purity = 97%):

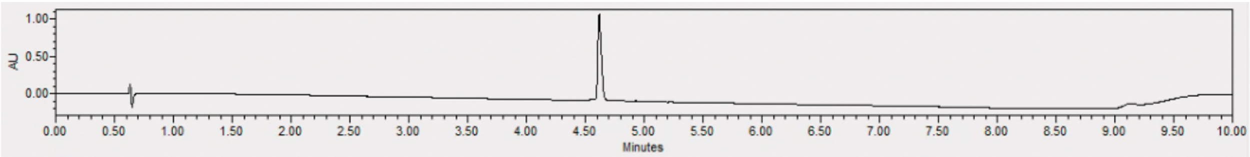

HR-ESI-MS:

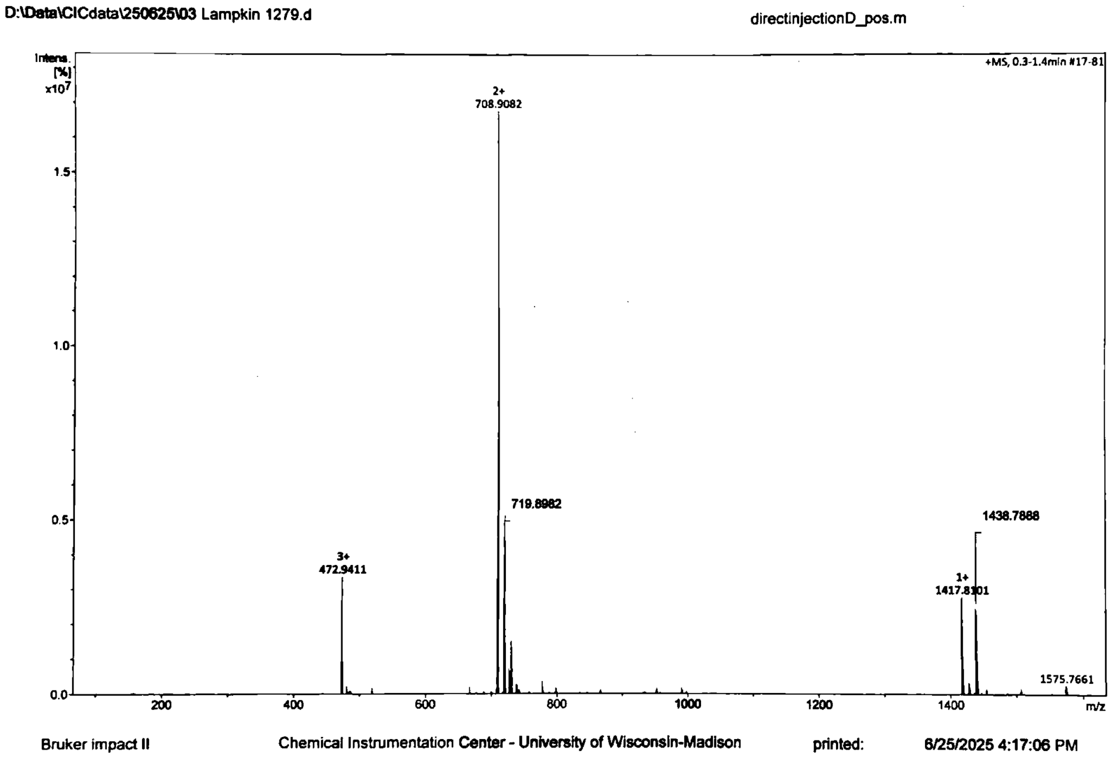

m/z observed: 1417.8101 ([M+H]$^+$) and 708.9082 ([M+2H]$^{2+}$)

m/z calculated: $C_{78}H_{106}N_{13}O_{12}^+$= 1416.8079 ([M+H]$^+$) and $C_{78}H_{107}N_{13}O_{12}^{2+}$= 708.9076 ([M+2H]$^{2+}$)

TPA-ACPC-ACPC-Dap(SuccHy)-ACPC-ACPC-Dap(SuccHy)-B^{3}HTyr-C(O)NHMe (10)

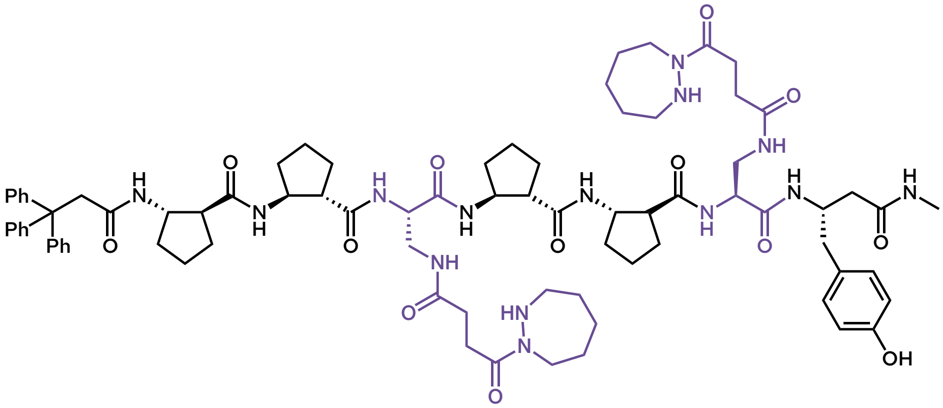

UPLC (220 nm channel, Purity = 95%):

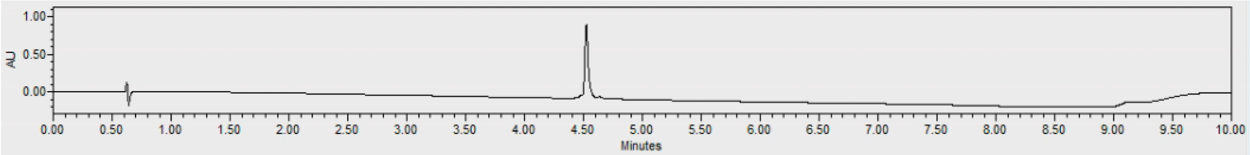

HR-ESI-MS:

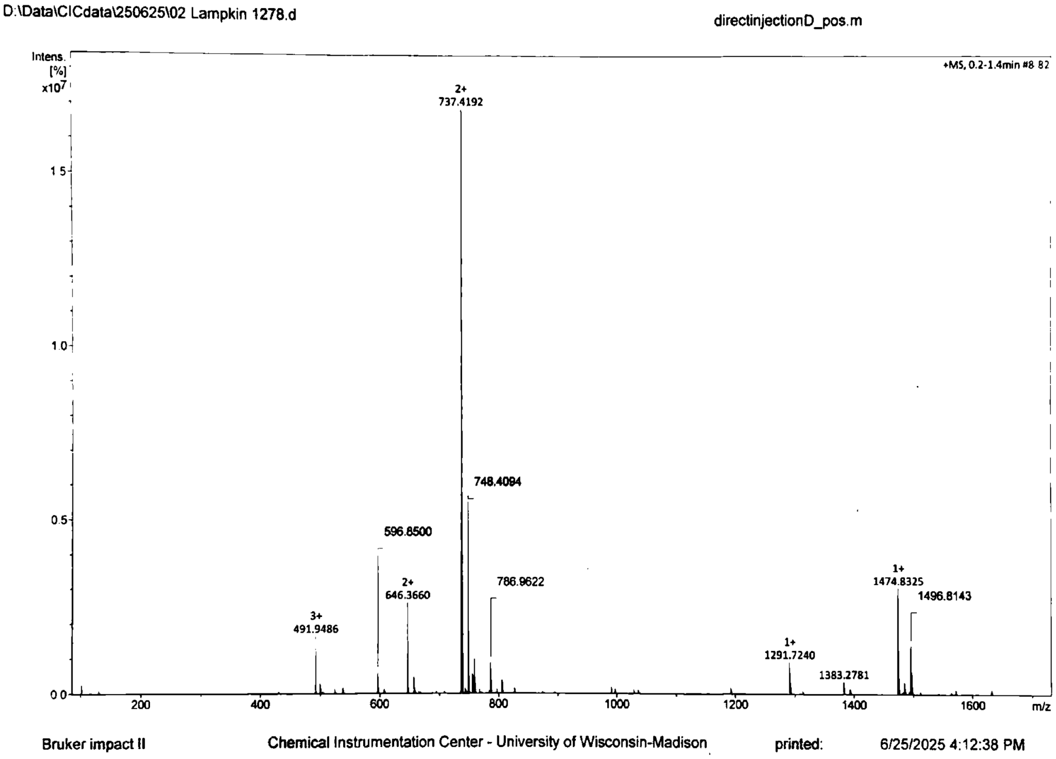

m/z observed: 1474.8325 ([M+H]$^{+}$) and 737.4192 ([M+2H]$^{2+}$)

m/z calculated: $C_{80}H_{109}N_{14}O_{13}^{+}$= 1473.8294 ([M+H]$^{+}$) and $C_{80}H_{110}N_{14}O_{13}^{2+}$= 737.4183 ([M+2H]$^{2+}$)

TPA-Glu(Hy)-ACPC-ACPC-Glu(Hy)-B³HTyr-C(O)NHMe (11)

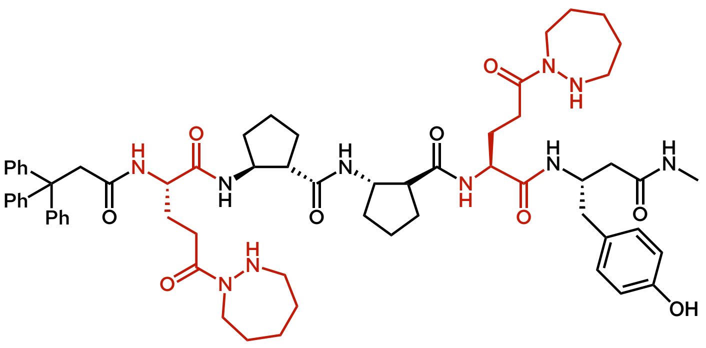

UPLC (220 nm channel, Purity = 92%):

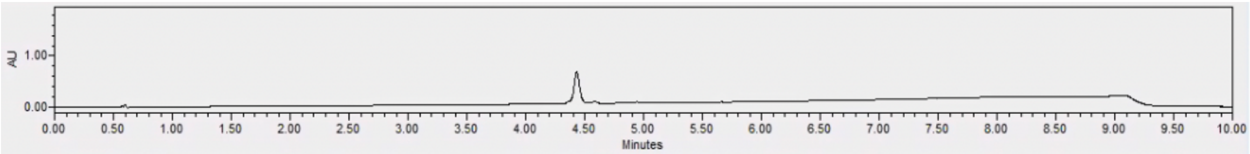

HR-ESI-MS:

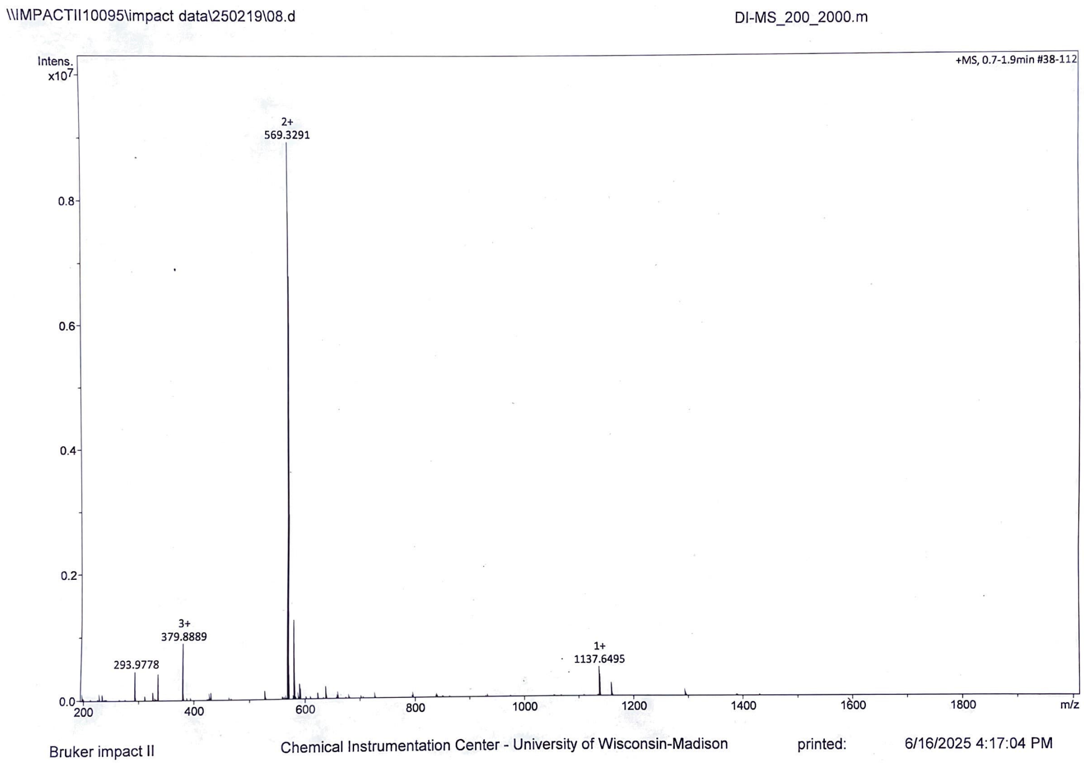

m/z observed: 1137.6495 ([M+H]$^+$) and 569.3291 ([M+2H]$^{2+}$)
m/z calculated: $C_{64}H_{85}N_{10}O_9^+$= 1137.6496 ([M+H]$^+$) and $C_{64}H_{86}N_{10}O_9^{2+}$= 569.3284 ([M+2H]$^{2+}$)

TPA-ACPC-ACPC-Glu(Hy)-ACPC-ACPC-Glu(Hy)-ACPC-ACPC-Tyr-C(O)NHMe (12)

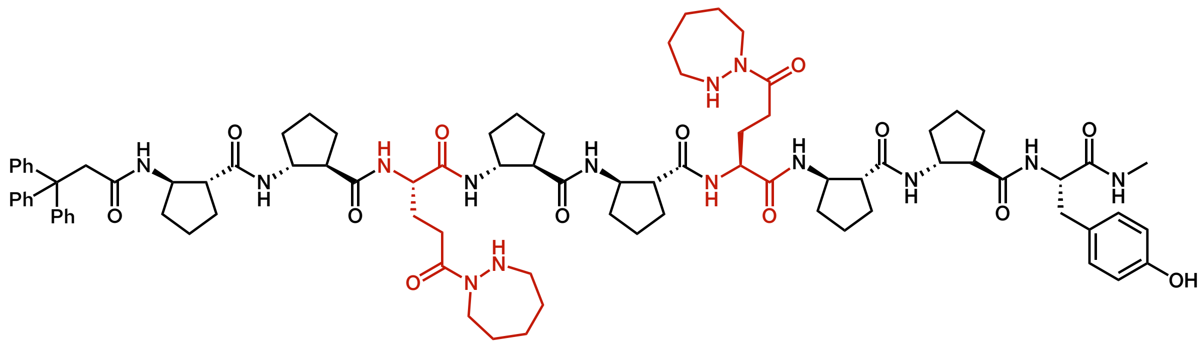

UPLC (220 nm channel, Purity = 93%):

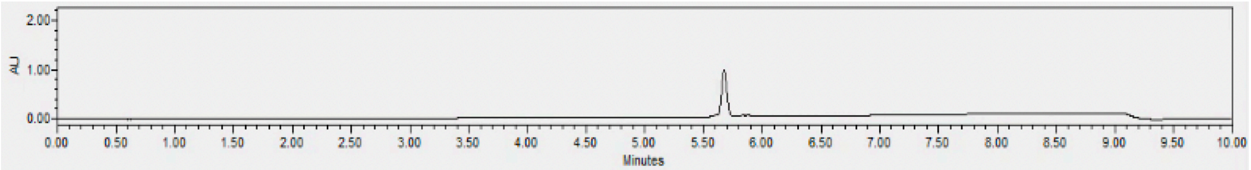

HR-ESI-MS:

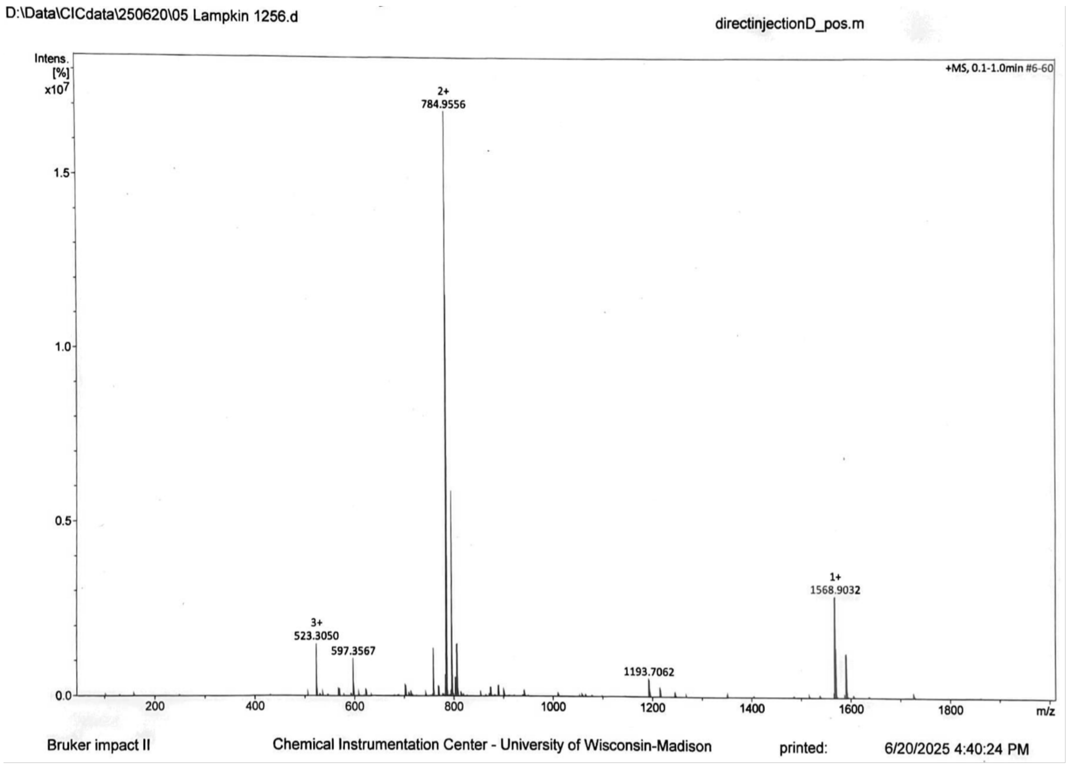

m/z observed: 1568.9032 ([M+H]$^{+}$) and 784.9556 ([M+2H]$^{2+}$)
m/z calculated: $C_{87}H_{119}N_{14}O_{13}^{+}$= 1567.9076 ([M+H]$^{+}$) and $C_{87}H_{120}N_{14}O_{13}^{2+}$= 784.4574 ([M+2H]$^{2+}$)

TPP-ACPC-Glu(Hy)-ACPC-ACPC-Glu(Hy)-ACPC-β^{3}HTyr-C(O)NHMe (15)

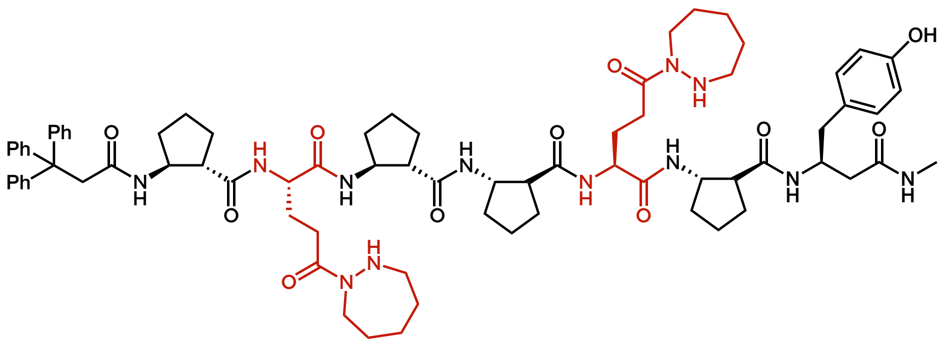

UPLC (220 nm channel, Purity = 91%):

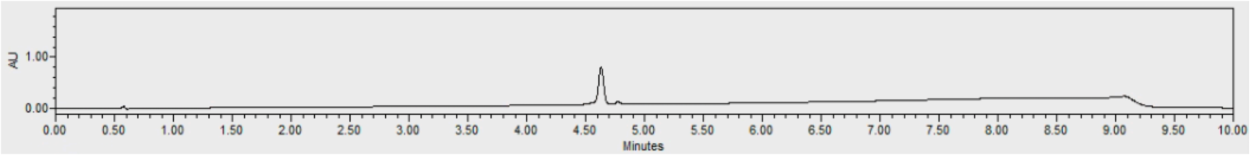

HR-ESI-MS:

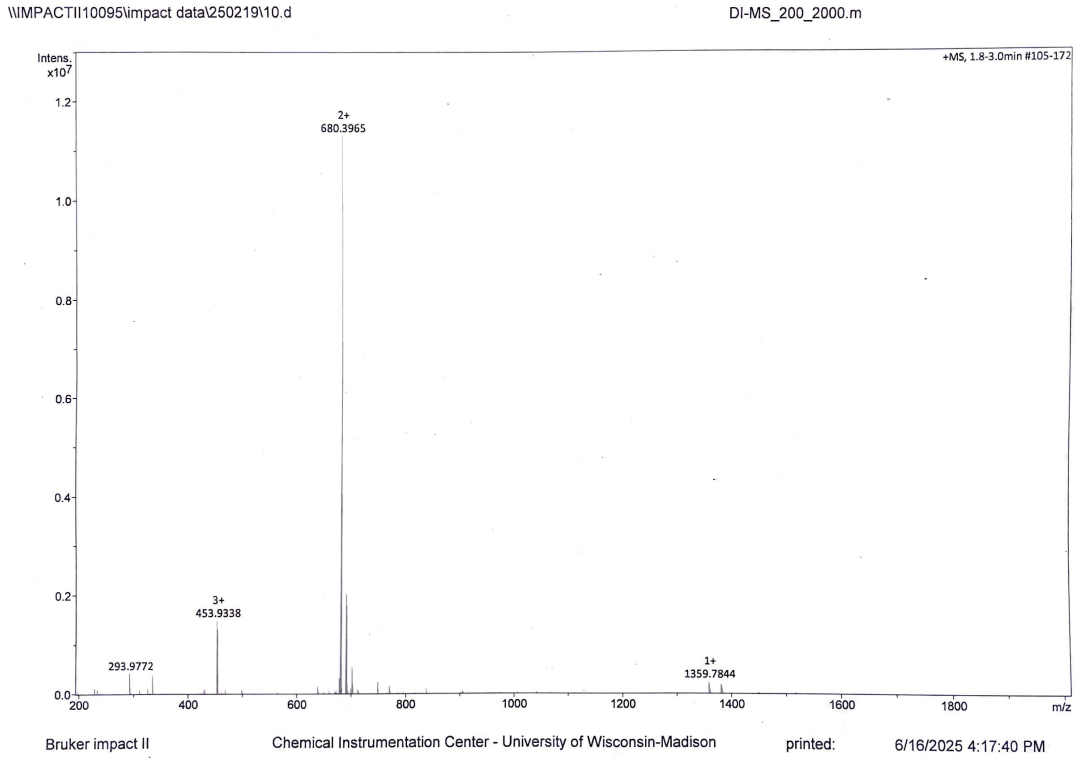

m/z observed: 1359.7844 ($[M+H]^+$) and 680.3965 ($[M+2H]^{2+}$)
m/z calculated: $C_{76}H_{103}N_{12}O_{11}^+$= 1359.7864 ($[M+H]^+$) and $C_{76}H_{104}N_{12}O_{11}^{2+}$= 680.3968 ($[M+2H]^{2+}$)

TPP-ACPC-ACPC-Glu(Hy)-ACPC-ACPC-Aze-B[3]HTyr-C(O)NHMe (16)

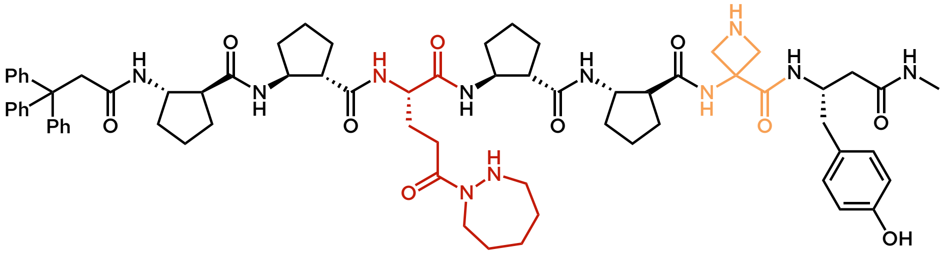

UPLC (220 nm channel, Purity = 97%):

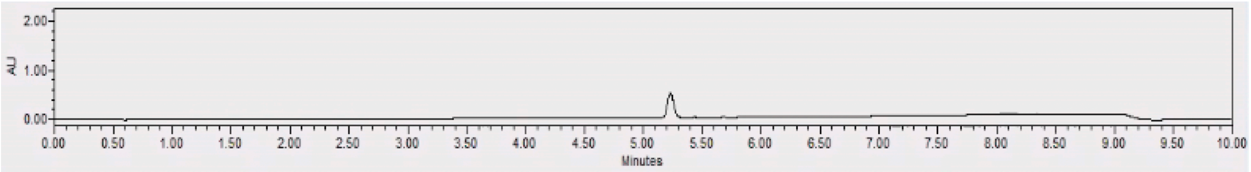

HR-ESI-MS:

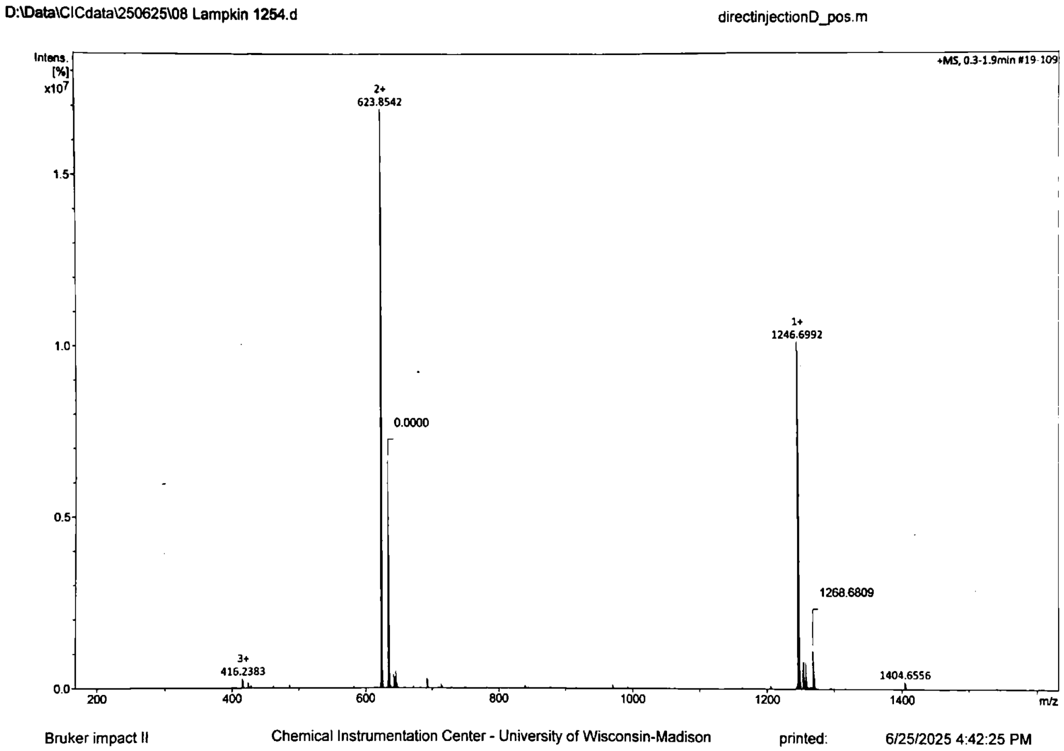

m/z observed: 1246.6992 ($[M+H]^+$) and 623.8542 ($[M+2H]^{2+}$)
m/z calculated: $C_{70}H_{92}N_{11}O_{10}^{+}$= 1246.7024 ($[M+H]^+$) and $C_{70}H_{93}N_{11}O_{10}^{2+}$= 623.8548 ($[M+2H]^{2+}$)

TPP-ACPC-ACPC-Glu(Hy)-ACPC-ACPC-Dab-β^{3}HTyr-C(O)NHMe (17)

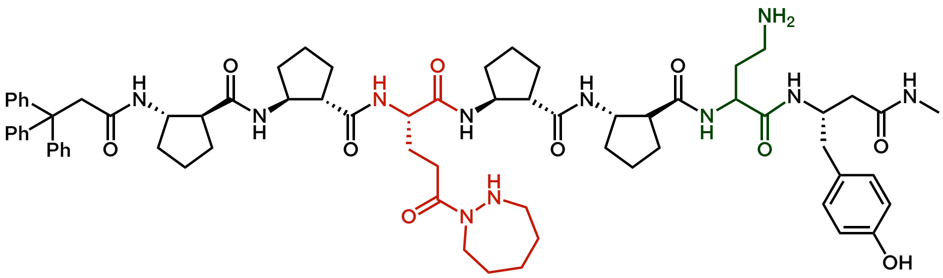

UPLC (220 nm channel, Purity = 98%):

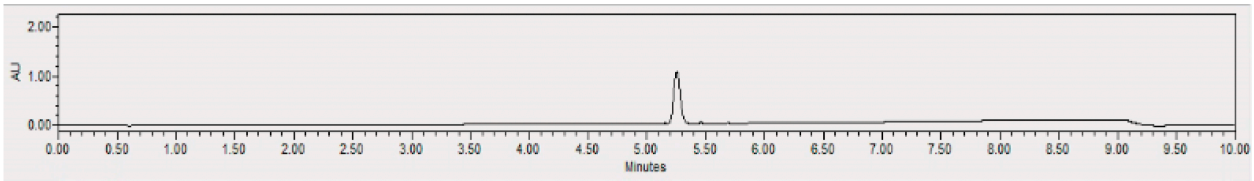

HR-ESI-MS:

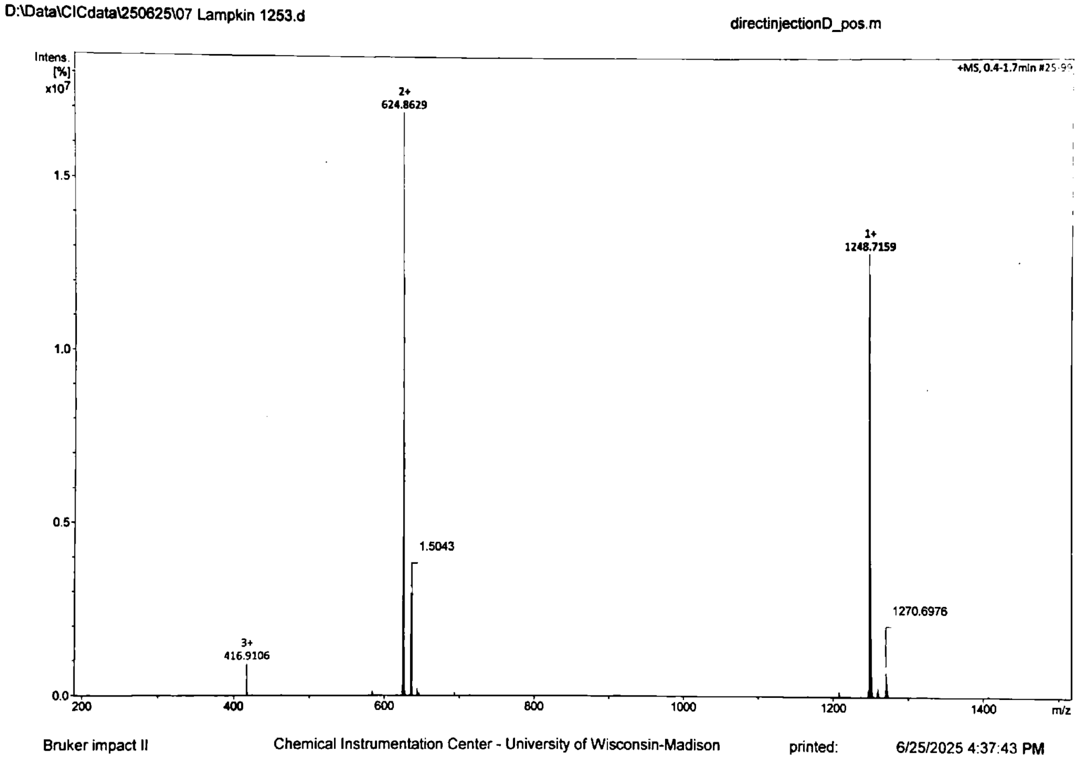

m/z observed: 1248.7159 ($[M+H]^+$) and 624.8629 ($[M+2H]^{2+}$)

m/z calculated: $C_{70}H_{94}N_{11}O_{10}^{+}$= 1248.7180 ($[M+H]^+$) and $C_{70}H_{95}N_{11}O_{10}^{2+}$= 624.8626 ($[M+2H]^{2+}$)

TPP-ACPC-ACPC-Glu(Hy)-ACPC-ACPC-Lys-β[3]HTyr-C(O)NHMe (18)

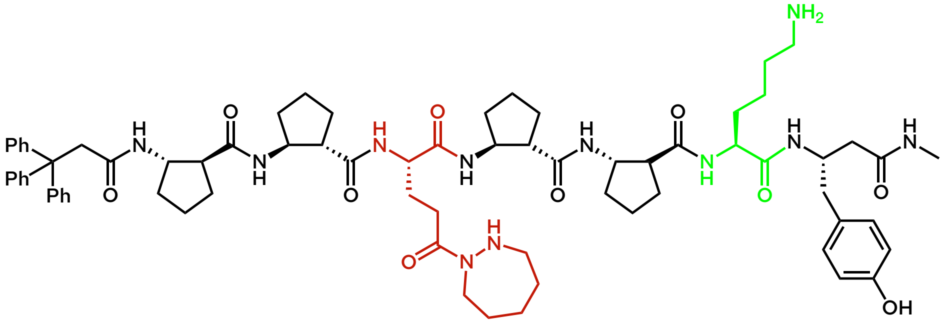

UPLC (220 nm channel, Purity = 93%):

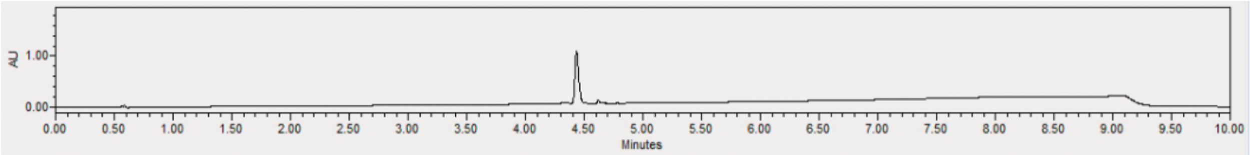

HR-ESI-MS:

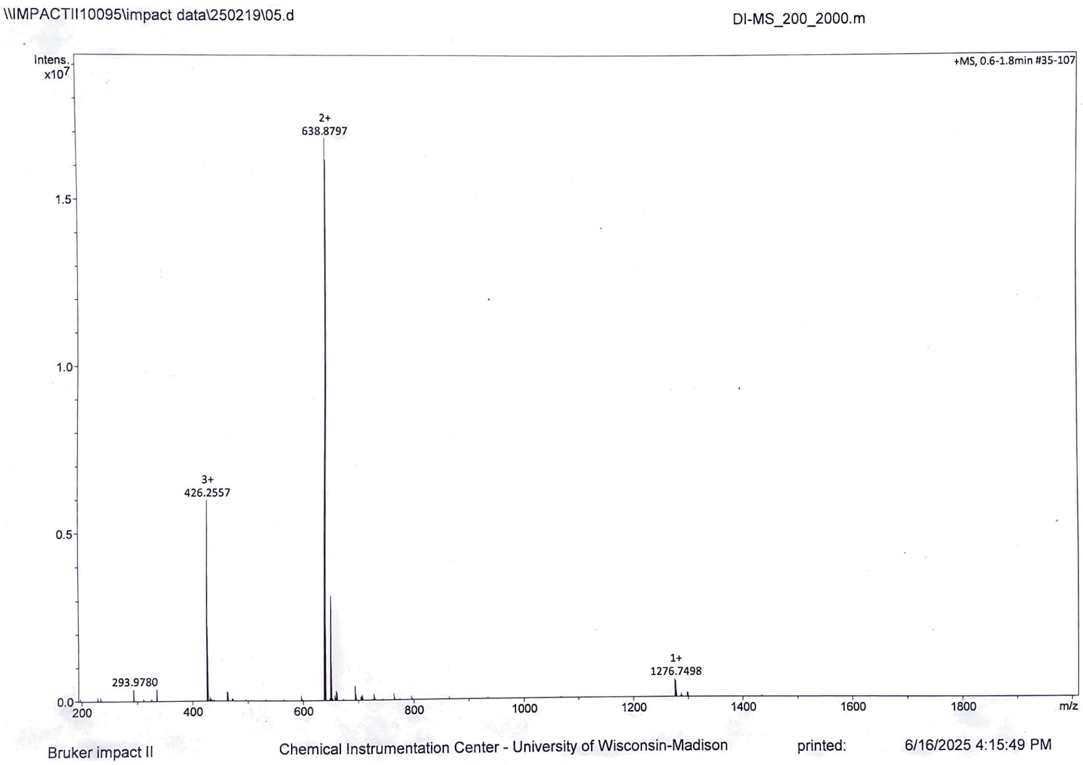

m/z observed: 1276.7498 ($[M+H]^+$) and 638.8797 ($[M+2H]^{2+}$)
m/z calculated: $C_{72}H_{98}N_{11}O_{10}^{+}$= 1276.7493 ($[M+H]^+$) and $C_{72}H_{99}N_{11}O_{10}^{2+}$= 638.8783 ($[M+2H]^{2+}$)

TPP-C(O)NH-ACPC-ACPC-Aze-ACPC-ACPC-Aze-B^{3}HTyr-C(O)NHMe (19)

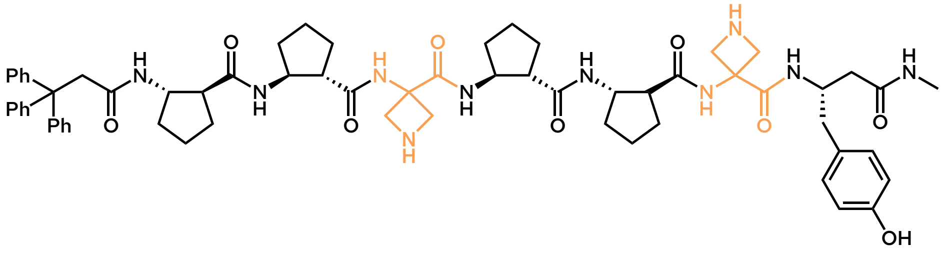

UPLC (220 nm channel, Purity = 97%):

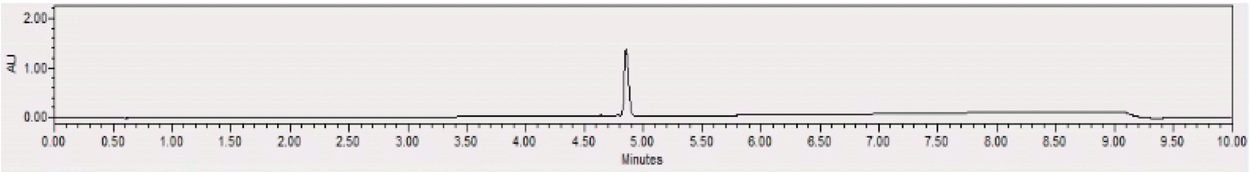

HR-ESI-MS:

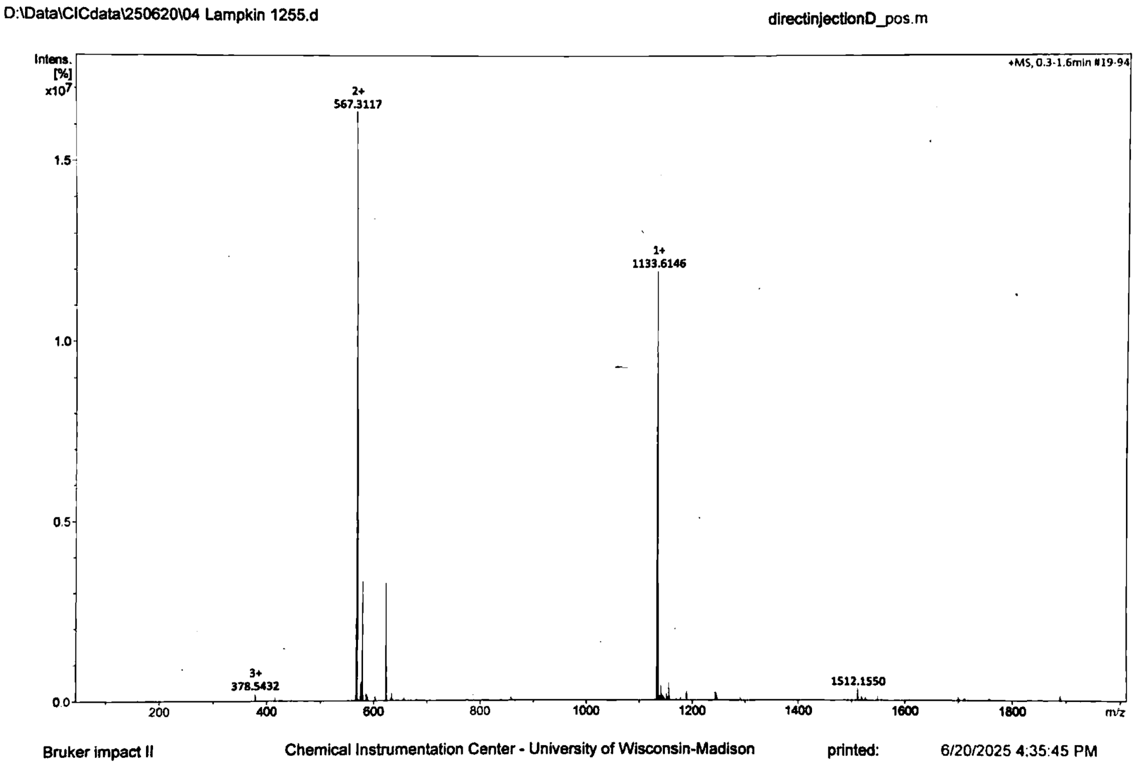

m/z observed: 1133.6146 ([M+H]$^+$) and 567.3117 ([M+2H]$^{2+}$)

m/z calculated: $C_{64}H_{81}N_{10}O_9^+$= 1133.6183 ([M+H]$^+$) and $C_{64}H_{82}N_{10}O_9^{2+}$= 567.3128 ([M+2H]$^{2+}$)

TPP-ACPC-ACPC-Dab-ACPC-ACPC-Dap-B³HTyr-C(O)NHMe (20)

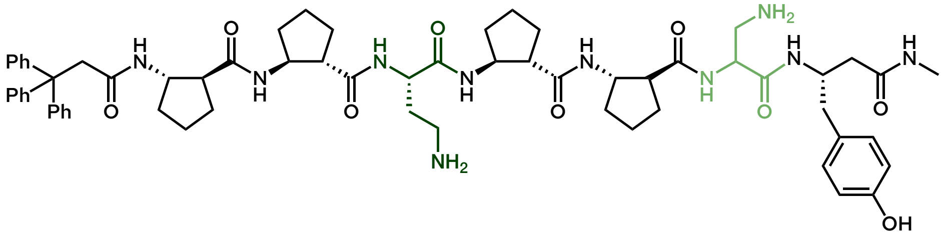

UPLC (220 nm channel, Purity = 100%):

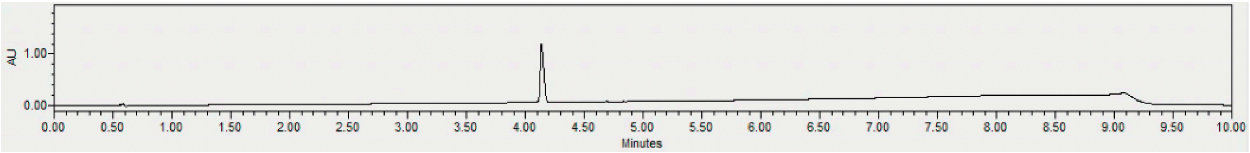

HR-ESI-MS:

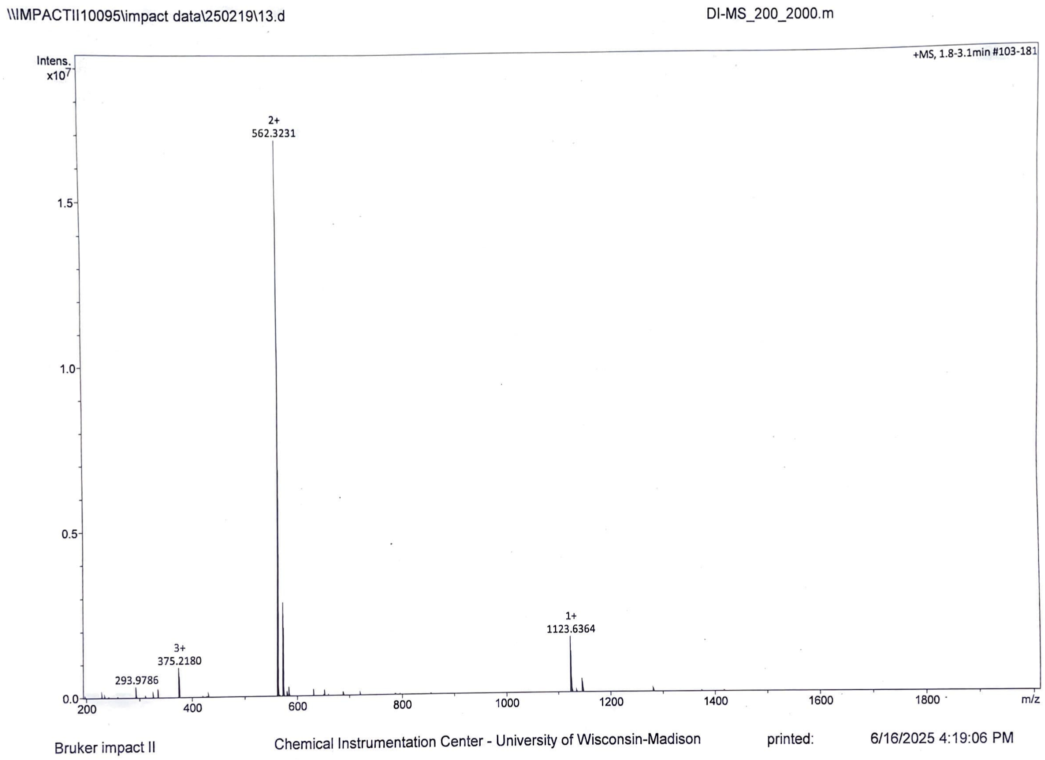

m/z observed: 1123.6364 ([M+H]$^{+}$) and 562.3231 ([M+2H]$^{2+}$)
m/z calculated: $C_{63}H_{83}N_{10}O_9^{+}$= 1123.6339 ([M+H]$^{+}$) and $C_{63}H_{84}N_{10}O_9^{2+}$= 562.3206 ([M+2H]$^{2+}$)

TPP-ACPC-ACPC-Lys-ACPC-ACPC-Lys-β3HTyr-C(O)NHMe (21)

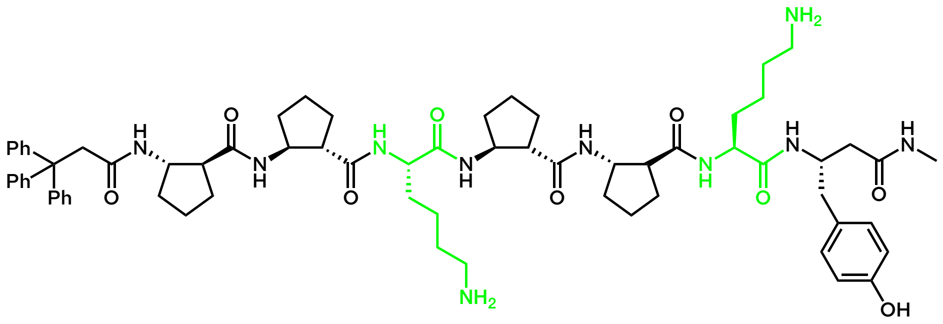

UPLC (220 nm channel, Purity = 97%):

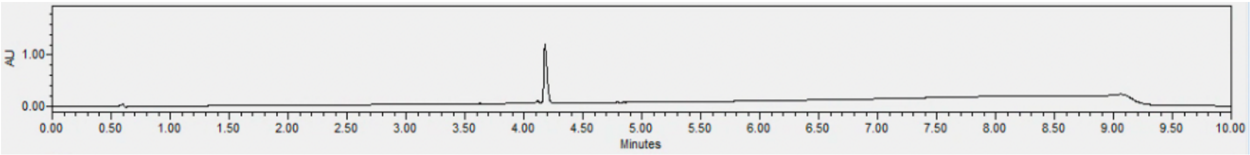

HR-ESI-MS:

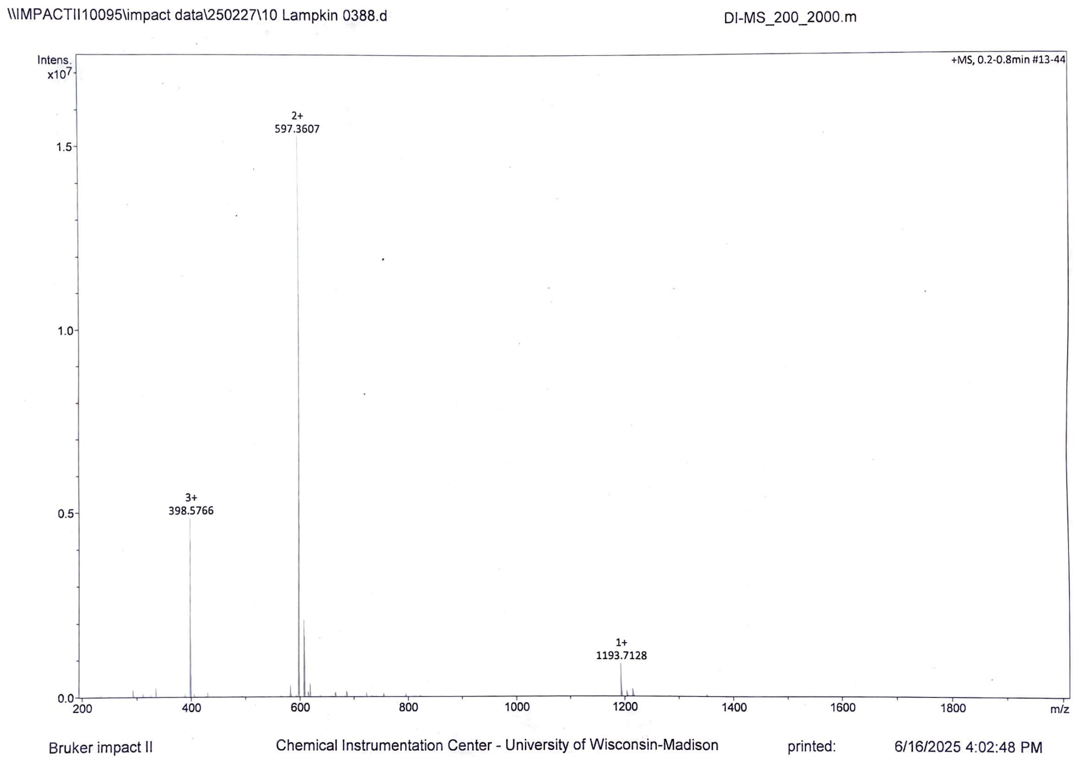

m/z observed: 1193.7128 ($[M+H]^+$) and 597.3607 ($[M+2H]^{2+}$)
m/z calculated: $C_{68}H_{93}N_{10}O_9^+$= 1193.7122 ($[M+H]^+$) and $C_{68}H_{94}N_{10}O_9^{2+}$= 597.3597 ($[M+2H]^{2+}$)

8. References

(1) Lampkin, P. P.; Gellman, S. H. Dual Activation Modes Enable Bifunctional Catalysis of Aldol Reactions by Flexible Dihydrazides *J. Am. Chem. Soc.* **2025**, *147*, 4, 3731–3739. https://doi.org/10.1021/jacs.4c16143.

(2) Yang, J.; Yu, Y.; Rebek, J.; Selective Macrocycle Formation in Cavitands. *J. Am. Chem. Soc.* **2021**, *143*, 5, 2190–2193. https://doi.org/10.1021/jacs.0c12302.

(3) Cheng, Y. A.; Chen, T.; Tan, C. K.; Heng, J. J.; Yeung, Y. Efficient Medium Ring Size Bromolactonization Using a Sulfur-Based Zwitterionic Organocatalyst *J. Am. Chem. Soc.* **2012**, *134*, 40, 16492–16495. https://doi.org/10.1021/ja307210n.

(4) *APEX – Version 2023.9-RC115.* Bruker AXS LLC. Madison, Wisconsin, USA. 2023.

(5) Krause, L.; Herbst-Irmer, R.; Sheldrick, G. M.; Stalke, D. Comparison of silver and molybdenum microfocus X-ray sources for single-crystal structure determination. *J. Appl. Cryst.* **2015***,* 48, 3-10. https://doi.org/10.1107/S1600576714022985.

(6) *XPREP – Version 2013/1.* Sheldrick, G. M. Georg-August-Universität Göttingen, Göttingen, Germany. 2013.

(7) *The SHELX homepage*, Sheldrick, G. M. 2013. http://shelx.uni-ac.gwdg.de/SHELX/.

(8) Sheldrick, G. M. *SHELXT* – Integrated space-group and crystal-structure determination. *Acta Cryst. A*, **2015**, 71, 3-8. https://doi.org/10.1107/S2053273314026370.

(9) Sheldrick, G. M. Crystal structure refinement with *SHELXL*. *Acta Cryst. C*, **2015**, 71, 3-8. https://doi.org/10.1107/S2053229614024218.

(10) Dolomanov, O. V.; Bourhis, L. J.; Gildea, R. J.; Howard, J. A. K.; Puschmann, H. *OLEX2*: a complete structure solution, refinement and analysis program. *J. Appl. Crystallogr.* **2009***,* 42, 339-341. https://doi.org/10.1107/S0021889808042726.

(11) Guzei, I. A. Programs *Gn*. University of Wisconsin-Madison, Madison, Wisconsin, USA. 2007-2022.

(12) Kleemiss, F.; Dolomanov, O. V.; Bodensteiner, M.; Peyerimhoff, N.; Midgley, L.; Bourhis, L. J.; Genoni, A.; Malaspina, L. A.; Jayatilaka, D.; Spencer, J. L.; White, F.; Grundkötter-Stock, B.; Steinhauer, S.; Lentz, D.; Puschmann, H.; Grabowsky, S. Accurate crystal structures and chemical properties from NoSpherA2. *Chem. Sci*. **2021**, 12, 1675-1692. https://doi.org/10.1039/D0SC05526C.

(13) Bourhis, L. J.; Dolomanov, O. V.; Gildea, R. J.; Howard, J. A. K.; Puschmann, H. The anatomy of a comprehensive constrained, restrained refinement program for the modern computing environment – Olex2 dissected. *Acta Cryst. A*. **2015**, 71, 59-75. https://dx.doi.org/10.1107/S2053273314022207.

(14) Neese, F. Software update: the ORCA program system, version 4.0. *Wiley Interdiscip. Rev.: Comput. Mol. Sci*. **2018**, 8, 1, e1327. https://doi.org/10.1002/wcms.1327.

(15) Neese, F. Software Update: The ORCA Program System – Version 6.0. *Wiley Interdiscip. Rev.: Comput. Mol. Sci*. **2025**, 15, 2, e70019. https://doi.org/10.1002/wcms.70019.

(16) *RDKit: Open-source cheminformatics – Release 2025.03.1*, Landrum, G., ETH Zurich, Switzerland. 2025. (https://doi.org/10.5281/zenodo.15115844).

(17) *Gaussian 16, Revision C.01* Frisch, M. J.; Trucks, G. W.; Schlegel, H. B.; Scuseria, G. E.; Robb, M. A.; Cheeseman, J. R.; Scalmani, G.; Barone, V.; Petersson, G. A.; Nakatsuji, H.; Li, X.; Caricato, M.; Marenich, A. V.; Bloino, J.; Janesko, B. G.; Gomperts, R.; Mennucci, B.; Hratchian, H. P.; Ortiz, J. V.; Izmaylov, A. F.; Sonnenberg, J. L.; Williams-Young, D.; Ding, F.; Lipparini, F.; Egidi, F.; Goings, J.; Peng, B.; Petrone, A.; Henderson, T.; Ranasinghe, D.; Zakrzewski, V. G.; Gao, J.; Rega, N.; Zheng, G.; Liang, W.; Hada, M.; Ehara, M.; Toyota, K.; Fukuda, R.; Hasegawa, J.; Ishida, M.; Nakajima, T.; Honda, Y.; Kitao, O.; Nakai, H.; Vreven, T.; Throssell, K.; Montgomery, J. A., Jr.; Peralta, J. E.; Ogliaro, F.; Bearpark, M. J.; Heyd, J. J.; Brothers, E. N.; Kudin, K. N.; Staroverov, V. N.; Keith, T. A.; Kobayashi, R.; Normand, J.; Raghavachari, K.; Rendell, A. P.; Burant, J. C.; Iyengar, S. S.; Tomasi, J.; Cossi, M.; Millam, J. M.; Klene, M.; Adamo, C.; Cammi, R.; Ochterski, J. W.; Martin, R. L.; Morokuma, K.; Farkas, O.; Foresman, J. B.; Fox, D. J. Gaussian, Inc., Wallingford CT, 2016.

(18) Dunning, T. H. Gaussian basis sets for use in correlated molecular calculations. I. The atoms boron through neon and hydrogen. *J. Chem. Phys.* **1989**, *90*, 1007–1023. https://doi.org/10.1063/1.456153.

(19) Kendall, R. A.; Dunning, T. H.; Harrison, R. J. Electron Affinities of the First-Row Atoms Revisited. Systematic Basis Sets and Wave Functions *J. Chem. Phys.* **1992**, 96, 6796-6806. http://doi.org/10.1063/1.462569.

(20) Zhao, Y.; Truhlar, D.G. The M06 suite of density functionals for main group thermochemistry, thermochemical kinetics, noncovalent interactions, excited states, and transition elements: two new functionals and systematic testing of four M06-class functionals and 12 other functionals. *Theor. Chem. Account*, **2008**, *120*, 215–241. https://doi.org/10.1007/s00214-007-0310-x.

(21) Schäfer, A.; Horn, H.; Ahlrichs, R. Fully optimized contracted Gaussian basis sets for atoms Li to Kr. *J. Chem. Phys.,* **1992**, 97, 2571-2577. https://doi.org/10.1063/1.463096.

(22) Cossi, M.; Rega, N.; Scalmani, G.; Barone, V. Energies, structures, and electronic properties of molecules in solution with the C-PCM solvation model. *J. Comput. Chem.,* **2003**, 24, 669-681. https://doi.org/10.1002/jcc.10189.

(23) Bursch, M.; Mewes, J.; Hansen, A.; Grimme, S.; Best-Practice DFT Protocols for Basic Molecular Computational Chemistry. *Angew. Chemie. Int. Ed.* **2022**, 61 (42), e202205735. https://doi.org/10.1002/anie.202205735.

(24) *CYLview20.* Legault, C. Y., Université de Sherbrooke, 2020 (http://www.cylview.org).

(25) Byrd, J. N.; Bartlett, R. J.; Montgomery, J. A. At What Chain Length Do Unbranched Alkanes Prefer Folded Conformations? *Journal of Physical Chemistry A* **2014**, *118* (9), 1706–1712. https://doi.org/10.1021/jp4121854.

(26) Liakos, D. G.; Neese, F. Domain Based Pair Natural Orbital Coupled Cluster Studies on Linear and Folded Alkane Chains. *J Chem Theory Comput* **2015**, *11* (5), 2137–2143. https://doi.org/10.1021/acs.jctc.5b00265.

(27) Ehlert, S.; Grimme, S. Hansen, A. Conformational Energy Benchmark for Longer n-Alkane Chains *J. Phys. Chem. A* **2022**, *126*, 22, 3521–3535. https://doi.org/10.1021/acs.jpca.2c02439.

9. Instrumentation Funding Acknowledgements

- Bruker D8 VENTURE Photon III Cu IμS 3.0 X-ray diffractometer – NSF grant CHE-1919350.
- The Bruker NEO-500 NMR spectrometer – NSF grant CHE-2017891.
- Bruker Impact II – Bender gift to the UW–Madison department of chemistry.
